# Biotransformation of Flavonoids with -NO_2_, -CH_3_ Groups and -Br, -Cl Atoms by Entomopathogenic Filamentous Fungi

**DOI:** 10.3390/ijms24119500

**Published:** 2023-05-30

**Authors:** Martyna Perz, Agnieszka Krawczyk-Łebek, Monika Dymarska, Tomasz Janeczko, Edyta Kostrzewa-Susłow

**Affiliations:** Department of Food Chemistry and Biocatalysis, Faculty of Biotechnology and Food Science, Wrocław University of Environmental and Life Sciences, 50-375 Wrocław, Poland; agnieszka.krawczyk-lebek@upwr.edu.pl (A.K.-Ł.); monika.dymarska@upwr.edu.pl (M.D.); tomasz.janeczko@upwr.edu.pl (T.J.)

**Keywords:** biotransformations, methyl group, nitro group, chlorine, bromine, *Beauveria bassiana*, *Isaria fumosorosea*, *Isaria farinosa*

## Abstract

Combining chemical and microbiological methods using entomopathogenic filamentous fungi makes obtaining flavonoid glycosides possible. In the presented study, biotransformations were carried out in cultures of *Beauveria bassiana* KCH J1.5, *Isaria fumosorosea* KCH J2, and *Isaria farinosa* KCH J2.6 strains on six flavonoid compounds obtained in chemical synthesis. As a result of the biotransformation of 6-methyl-8-nitroflavanone using the strain *I. fumosorosea* KCH J2, two products were obtained: 6-methyl-8-nitro-2-phenylchromane 4-*O-β*-D-(4″-*O*-methyl)-glucopyranoside and 8-nitroflavan-4-ol 6-methylene-*O-β*-D-(4″-*O*-methyl)-glucopyranoside. 8-Bromo-6-chloroflavanone was transformed by this strain to 8-bromo-6-chloroflavan-4-ol 4′-*O-β*-D-(4″-*O*-methyl)-glucopyranoside. As a result of microbial transformation by *I. farinosa* KCH J2.6 effectively biotransformed only 8-bromo-6-chloroflavone into 8-bromo-6-chloroflavone 4′-*O-β*-D-(4″-*O*-methyl)-glucopyranoside. *B. bassiana* KCH J1.5 was able to transform 6-methyl-8-nitroflavone to 6-methyl-8-nitroflavone 4′-*O-β*-D-(4″-*O*-methyl)-glucopyranoside, and 3′-bromo-5′-chloro-2′-hydroxychalcone to 8-bromo-6-chloroflavanone 3′-*O-β*-D-(4″-*O*-methyl)-glucopyranoside. None of the filamentous fungi used transformed 2′-hydroxy-5′-methyl-3′-nitrochalcone effectively. Obtained flavonoid derivatives could be used to fight against antibiotic-resistant bacteria. To the best of our knowledge, all the substrates and products presented in this work are new compounds and are described for the first time.

## 1. Introduction

The microbial transformation process is a well-known method of obtaining modified organic compounds like glycosides or hydroxylic derivatives using enzymes and whole cells [1,2,3]. This eco-friendly process allows obtaining compounds with high stereo-, chemo-, and regioselectivity. Other benefits, like rapid growth of the microorganisms, improvement of selectivity, and easy upscaling, make biotransformations good alternatives for traditional chemical synthesis [4,5]. 

There are many ways to obtain different flavonoid modifications using various microorganisms [5]. For example, bacteria strains like *Stenotrophomonas maltophilia* KB2 can carry out demethylation of 7-methoxyflavanones, and open the heterocyclic C ring in various methoxyflavanones, 7-methoxyflavanone, 3′-methoxyflavanone, and 5,7-dimethoxyflavanone [6]. Moreover, Park and coworkers showed that Lactic Acid Bacteria (LAB) can convert hesperidin to hesperetin [7].

With very high efficiency, yeast cultures can also carry out the biotransformation process. Unconventional yeast strains like *Rhodotorula rubra* KCH 4 and KCH 82, *Rhodotorula marina* KCH 77, *Rhodotorula glutinis* KCH 242, *Candida viswanathii* KCH 120, *Candida parapsilosis* KCH 909, *Saccharomyces cerevisiae* KCH 464, and *Yarrowia lipolytica* KCH 71 were used to carry out the biotransformation of 2′-hydroxychalcones with bromine atom at C-2, C-3, C-4, C-5′ positions. All substrates used were converted to the corresponding dihydrochalcones [8]. Other studies have shown that *S. cerevisiae* KCH 464 and *Y. lipolytica* KCH 71 were also able to hydrogenate 3-(2″-furyl)-1-(2′-hydroxyphenyl)-prop-2-en-1-one with >99% yield [9].

Other research showed that fungus *Mucor hiemalis* can transform 6,2′-dimethoxy-flavonol into 6,2′-dimethoxyflavonol-3-*O-β*-D-glucopyranoside [10].

Compared to the hydroxylation, methylation, or hydrogenation of flavonoids, glycosylation is much more challenging to carry out because of the larger glucose molecule and its higher spherical hindrance [11,12,13]. Moreover, obtaining flavonoid glycosides by chemical methods is very difficult. Chemical synthesis requires multiple repetitive steps of reaction, coupling, and toxic catalysts [14,15,16]. Comparing it with the microbial transformation process, it is much simpler and does not require multi-stage reactions and toxic reaction catalysts [17,18,19,20,21]. Filamentous fungi carry out the biotransformation process of polyphenolic compounds very efficiently [18,22,23]. Moreover, the glycosidic molecule improves the solubility and bioavailability of flavonoids and makes them more attractive to use in the pharmacological industry [14,21,24]. The flavonoid glycosides are present in nature and can be isolated from plants, some fungi, etc. [25,26].

Krawczyk-Łebek and coworkers have shown the glycosylation ability of entomopathogenic filamentous fungi. 4′-Methylflavone was transformed via *Isaria fumosorosea* KCH J2 into four products: 4′-hydroxymethylflavone, flavone 4′-methylene-*O-β*-D-(4″-*O*-methyl)-glucopyranoside, flavone 4′-carboxylic acid, and 4′-methylflavone 3-*O-β*-D-(4″-*O*-methyl)-glucopyranoside [22]. Moreover, the same strain was able to transform 6-methylfavone into 6-methylflavone 8-*O-β*-D-(4″-*O*-methyl)-glucopyranoside and 6-methylflavone 4′-*O-β*-D-(4″-*O*-methyl)-glucopyranoside [23]. *Beauveria bassiana* KCH J1.5 transformed 2′-methyflavanone into three glycosylated flavanones at the positions C-6′, C-6, and C-4 [18].

Substances with new properties obtained using environmentally friendly methods are constantly sought after in the pharmaceutical, cosmetic, and food industries [27,28]. It is known that flavonoid compounds have valuable properties: antioxidant [29,30], anti-carcinogenic [30,31], modulating intestinal immune responses [32], hepatoprotective [32,33], antimicrobial [34,35,36], and others. 

Bacteria that are resistant to antibiotics are a significant global problem nowadays. Flavonoid compounds with bromine and chlorine atoms and methyl and nitro groups may be the solution, as some of them show bacteriostatic/bactericidal properties [36,37]. The antibiotic potentiating activity test demonstrated that the (2*E*)-1-(3′-methoxy-4′-hydroxyphenyl)-3-(3-nitrophenyl)prop-2-en-1-one chalcone (called AVMNB) associated with the antibiotics has activity against multi-resistant strains of bacteria. Combining AVMNB chalcone with antibiotic ciprofloxacin showed synergistic action against multidrug-resistant *Staphylococcus aureus* 10 strains. Moreover, a similar effect was observed for AVMNB chalcone combined with antibiotic gentamycin against *Escherichia coli* 06 strain [38]. Other studies showed that many methylated flavonoids have antibacterial properties [39], for example, 3-hydroxy-5-methoxy-4,6,6-trimethyl-2-[(E)-3-phenylprop-2-enoyl]cyclohexa-2,4-dien-1-one (Desmosdumotin C) has anti-*Helicobacter pylori* activity [40], 2′,6′-dihydroxy-4′-methoxy-3′,5′-dimethyldihydrochalcone and 2′,4′ -dihydroxy-6′-methoxy-5′-methyl-chalcone (aurentiacin A) were active against *Cladosporium cucumerinum*, *Bacillus subtills*, and *E. coli* [41]. Chloroflavonoids have also documented antimicrobial activity [36]. Minimal inhibitory concentration (MIC) of 2′-chloroflavone and 4′-chloroflavone were tested against *B. cereus*, *S. aureus*, *E. coli*, *Vibrio cholerae*, *Pseudomonas aeruginosa*, *Salmonella typhi* in comparison of ampicillin. The significance inhibition for both of compounds was for *B. cereus*, *S. aureus*, and *S. typhi* [36]. The bromine derivatives of flavonoids can potentially be used to limit the growth of undesirable microorganisms. Quercetin, 8-bromoquercetin, and 6,8-dibromoquercetin were tested against biofilm formation of *P. aeruginosa* and *S. aureus*. In the case of quercetin, there was no biofilm inhibition, but all bromine derivatives inhibited bacteria biofilm formation with an IC_50_ value of less than 40 µM in both cases [42].

In this study, we biotransformed using entomopathogenic filamentous fungi flavonoid compounds with chlorine, bromine, methyl, or nitro substituents. The presented research aimed to check the catalytic potential of the strains *B. bassiana* KCH J1.5, *I. fumosorosea* KCH J2, and *I. farinosa* KCH J2.6 towards flavonoid compounds with chlorine, bromine, nitro, and methyl groups. The substrates were selected because of their potential bactericidal/bacteriostatic properties. Our main point was to investigate the ability of the enzymatic systems of entomopathogenic filamentous fungi to biotransform flavonoids with various substituents rather absent in natural flavonoids. We aim at further studies to assess the biological properties and bioavailability of obtained flavonoids.

## 2. Results

As a result of the biotransformation of substrates (**5**), (**6**), (**7**), (**8**), and (**9**) in cultures of *B. bassiana* KCH J1.5, *I. fumosorosea* KCH J2, and *I. farinosa* KCH J2.6, glycosidic derivatives of flavonoids were obtained. Only 2′-hydroxy-5′-methyl-3′-nitrochalcone (**4**) in the cultures of the chosen filamentous fungi was not biotransformed. The products were extracted from fungal cultures and purified using preparative thin-layer chromatography (pTLC). Obtained products were analyzed structurally with the use of Nuclear Magnetic Resonance (NMR), and masses were confirmed by Liquid Chromatography-Mass Spectrometry (LC-MS). 

6-Methyl-8-nitro-2-phenylchromane 4-*O-β*-D-(4″-*O*-methyl)-glucopyranoside (**5a**). C_23_H_27_NO_9_, mp: 98–101 °C; HPLC Rt = 6.6 min; [α]_D_ = −32.311 (0.38 *w*/*v* % in acetone); **^1^H NMR** (600 MHz, acetone-d_6_) δ (ppm): 7.69 (1H, d, *J* = 1.6 Hz, H-7), 7.59 (1H, d, *J* = 2.1 Hz, H-5), 7.56–7.54 (2H, m, H-2′, H-6′), 7.43 (2H, dd, *J* = 10.4, 4.8 Hz, H-3′, H-5′), 7.37–7.33 (1H, m, H-4′), 5.55 (1H, dd, *J* = 12.1, 2.0 Hz, H-2), 5.06 (1H, t, *J* = 2.9 Hz, H-4), 4.50 (1H, d, *J* = 7.7 Hz, H-1″), 4.43 (1H, d, *J* = 3.9 Hz, 2″-OH), 4.25 (1H, d, *J* = 3.9 Hz, 3″-OH), 3.91 (1H, ddd, *J* = 11.4, 5.6, 2.1 Hz, H-6″), 3.86 (1H, m, 6″-OH), 3.72 (1H, dt, *J* = 11.7, 6.0 Hz, H-6″), 3.54 (3H, s, 4″-O-CH_3_), 3.50 (1H, dd, *J* = 8.9, 3.8 Hz, H-3″), 3.36 (1H, ddd, *J* = 9.8, 5.6, 2.1 Hz, H-5″), 3.30 (1H, ddd, *J* = 9.0, 8.0, 3.8 Hz, H-2″), 3.12 (1H, dd, *J* = 9.6, 9.0 Hz, H-4″), 2.60 (1H, dt, *J* = 14.4, 2.5 Hz, H-3ax), 2.37 (3H, s, -CH_3_), 2.19–2.13 (1H, m, H-3eq); **^13^C NMR** (acetone-d_6_) δ (ppm): 147.27 (C-8a), 141.48 (C-1′), 137.48 (C-5), 130.03 (C-6), 129.33 (C-3′, C-5′), 128.78 (C-4′), 126.83 (C-2′, C-6′), 126.53 (C-7), 125.43 (C-8), 123.87 (C-4a), 101.49 (C-1″), 80.59 (C-4″), 78.11 (C-3″), 77.10 (C-5″), 75.29 (C-2″), 75.12 (C-2), 69.42 (C-4), 62.54 (C-6″), 60.52 (C4″-O-CH_3_), 36.72 (C-3), 20.09 (-CH_3_).

8-Nitroflavan-4-ol 6-methylene-*O-β*-D-(4″-*O*-methyl)-glucopyranoside (**5b**). C_23_H_27_NO_10_, mp: 177 °C; HPLC Rt = 11.6 min; [α]_D_ = −37.348 (1.1 *w*/*v* % in acetone); **^1^H NMR** (600 MHz, acetone-d_6_) δ (ppm): 7.87 (1H, d, *J* = 2.1 Hz, H-7), 7.71 (1H, d, *J* = 2.0 Hz, H-5), 7.54 (2H, d, *J* = 7.5 Hz, H-2′, H-6′), 7.45–7.42 (2H, m, H-3′, H-5′), 7.36 (1H, t, *J* = 7.5 Hz, H-4′), 5.49 (1H, dd, *J* = 11.8, 2.1 Hz, H-2), 4.90 (1H, s, H-4), 4.90 (1H, d, *J* = 12.1 Hz, 6-CH_2_-O), 4.68 (1H, d, *J* = 12.1 Hz, 6-CH_2_-O), 4.41 (1H, d, *J* = 7.8 Hz, H-1″), 4.29 (1H, s, 2″-OH), 3.83 (1H, d, *J* = 11.6 Hz, H-6″), 3.76–3.70 (1H, m, 6″-OH), 3.65 (1H, dd, *J* = 14.1, 8.8 Hz, H-6″), 3.53 (3H, s, 4″-O-CH_3_), 3.51 (1H, d, *J* = 9.0 Hz, H-3″), 3.31–3.25 (2H, m, H-5″, H-2″), 3.13–3.08 (1H, m, H-4″), 2.39–2.36 (1H, m, H-3ax), 2.23–2.18 (1H, m, H-3eq); **^13^C NMR** (acetone-d_6_) δ (ppm): 148.21 (C-8a), 141.39 (C-1′), 139.96 (C-8), 137.40 (C-4a), 135.41 (C-5), 131.11 (C-6), 129.40 (C-3′, C-5′), 128.84 (C-4′), 126.78 (C-2′, C-6′), 124.95 (C-7), 103.29 (C-1″), 80.50 (C-4″), 78.09 (C-3″), 76.96 (C-5″), 75.24 (C-2), 75.15 (C-2″), 69.79 (6-CH_2_-O-), 63.10 (C-4), 62.44 (C-6″), 60.49 (4″-O-CH_3_), 38.83 (C-3).

6-Methyl-8-nitroflavone 4′-*O-β*-D-(4″-*O*-methyl)-glucopyranoside (**6a**). C_23_H_23_NO_10_, mp: 140–143 °C; HPLC Rt = 6.7 min; [α]_D_ = −12.707 (0.35 *w*/*v* % in acetone); **^1^H NMR** (600 MHz, acetone-d_6_) δ (ppm): 8.34 (1H, dd, *J* = 2.3, 0.6 Hz, H-7), 8.24 (1H, dd, *J* = 2.2, 0.8 Hz, H-5), 8.11–8.08 (2H, m, H-2′, H-6′), 7.29–7.26 (2H, m, H-3′, H-5′), 6.93 (1H, s, H-3), 5.12 (1H, d, *J* = 7.8 Hz, H-1″), 4.75 (1H, d, *J* = 4.2 Hz, 2″-OH), 4.46 (1H, d, *J* = 3.9 Hz, 3″-OH), 4.16–4.13 (1H, m, 6″-OH), 3.87 (1H, ddd, *J* = 11.8, 5.1, 2.3 Hz, H-6″), 3.74–3.70 (1H, m, H-6″), 3.68–3.66 (1H, m, H-3″), 3.58–3.57 (4H, m, 4″-O-CH_3_, H-5″), 3.54–3.50 (1H, m, H-2″), 3.27–3.23 (1H, m, H-4″), 2.59 (3H, s, -CH_3_); **^13^C NMR** (acetone-d_6_) δ (ppm): 179.09 (C=O), 163.80 (C-2), 161.81 (C-4′), 153.12 (C-8a), 139.88 (C-8), 136.11 (C-6), 131.58 (C-5), 131.36 (C-7), 129.17 (C-2′, C-6′), 125.75 (C-4a), 124.92 (C-1′), 117.77 (C-3′, C-5′), 106.73 (C-3), 101.14 (C-1″), 80.02 (C-4″), 77.97 (C-3″), 77.20 (C-5″), 74.86 (C-2″), 62.02 (C-6″), 60.58 (4″-O-CH_3_), 20.58 (-CH_3_).

8-Bromo-6-chloroflavanone 3′-*O-β*-D-(4″-*O*-methyl)-glucopyranoside (**7a**). C_22_H_22_BrClO_8_, mp: 97 °C; HPLC Rt = 13.6 min; [α]_D_ = −60.024 (0.41 *w*/*v* % in acetone) **^1^H NMR** (600 MHz, acetone-d_6_) δ (ppm): 7.91 (1H, d, *J* = 2.6 Hz, H-7), 7.77 (1H, d, *J* = 2.5 Hz, H-5), 7.38 (1H, td, *J* = 7.9, 3.0 Hz, H-5′), 7.32–7.29 (1H, m, H-2′), 7.25 (1H, d, *J* = 7.0 Hz, H-6′), 7.09 (1H, dd, *J* = 7.9, 2.1 Hz, H-4′), 5.81 (1H, dd, *J* = 12.6, 3.0 Hz, H-2), 5.01 (1H, d, *J* = 7.8 Hz, H-1″), 4.64 (1H, d, *J* = 4.2 Hz, 2″-OH), 4.39 (1H, d, *J* = 4.1 Hz, 3″-OH), 3.83 (1H, ddd, *J* = 11.6, 5.2, 2.2 Hz, H-6″), 3.73 (1H, dd, *J* = 7.9, 2.8 Hz, 6″-OH), 3.70–3.66 (1H, m, H-6″), 3.65–3.60 (1H, m, H-3″), 3.56 (3H, s, 4″-O-CH_3_), 3.50–3.46 (2H, m, H-2″, H-5″), 3.26 (2H, dd, *J* = 16.9, 12.5 Hz, H-3ax), 3.24–3.20 (1H, m, H-4″), 3.03 (1H, dd, *J* = 17.0, 3.1 Hz, H-3eq); **^13^C NMR** (acetone-d_6_) δ (ppm): 190.26 (C=O), 158.93 (C-3′), 157.58 (C-8a), 140.87 (C-1′), 138.83 (C-7), 130.68 (C-5′), 127.35 (C-6), 126.12 (C-5), 123.68 (C-4a), 120.72 (C-6′), 117.43 (C-4′), 115.23 (C-2′), 113.60 (C-8), 101.35 (C-1″), 80.84 (C-2), 80.07 (C-4″), 78.08 (C-3″), 77.06 (C-2″), 74.94 (C-5″), 62.05 (C-6″), 60.55 (4″-O-CH_3_), 43.85 (C-3). 

8-Bromo-6-chloroflavan-4-ol 4′-*O-β*-D-(4″-*O*-methyl)-glucopyranoside (**8a**). C_22_H_24_BrClO_8_, mp: 117 °C; HPLC Rt = 11.0 min; [α]_D_ = −8.725 (0.4 *w*/*v* % in acetone); **^1^H NMR** (600 MHz, acetone-d_6_) δ (ppm): 7.54 (1H, d, *J* = 2.5 Hz, H-7), 7.45 (2H, d, *J* = 8.6 Hz, H-2′, H-6′), 7.40 (1H, d, *J* = 2.5 Hz, H-5), 7.11 (2H, d, *J* = 8.7 Hz, H-3′, H-5′), 5.37 (1H, dd, *J* = 11.7, 2.2 Hz, H-2), 4.98 (1H, d, *J* = 7.8 Hz, H-1″), 4.85–4.81 (1H, m, H-4), 4.63 (1H, d, *J* = 4.2 Hz, 2″-OH), 4.38 (1H, d, *J* = 4.1 Hz, 3″-OH), 3.85 (1H, ddd, *J* = 11.7, 5.4, 2.2 Hz, H-6″), 3.76 (1H, dd, *J* = 7.1, 5.4 Hz, 6″-OH), 3.69 (1H, ddd, *J* = 7.1, 6.0, 4.3 Hz, H-6″), 3.66–3.61 (1H, m, H-3″), 3.57 (3H, s, 4″-O-CH_3_), 3.51–3.45 (2H, m, H-5″, H-2″), 3.24–3.20 (1H, m, H-4″), 2.29–2.25 (1H, m, H-3ax), 2.19–2.13 (1H, m, H-3eq); **^13^C NMR** (acetone-d_6_) δ (ppm): 158.63 (C-4′), 151.36 (C-8a), 135.03 (C-1′), 132.58 (C-7), 130.45 (C-5), 128.78 (C-4a), 128.25 (C-2′, C-6′), 125.45 (C-6), 117.36 (C-3′, C-5′), 111.84 (C-8), 101.64 (C-1″), 80.12 (C-4″), 78.04 (C-3″), 77.06 (C-5″), 74.98 (C-2″), 74.91 (C-2), 63.44 (C-4), 62.11 (C-6″), 60.55 (4″-O-CH_3_), 38.57 (C-3).

8-Bromo-6-chloroflavone 4′-*O-β*-D-(4″-*O*-methyl)-glucopyranoside (**9a**). C_22_H_20_BrClO_8_, mp: 160 °C; HPLC Rt = 11.1 min; [α]_D_ = −17.181 (1.39 *w*/*v* % in acetone) **^1^H NMR** (600 MHz, acetone-d_6_) δ (ppm): 8.12 (3H, dd, *J* = 5.7, 3.1 Hz, H-2′, H-6′, H-7), 8.01 (1H, d, *J* = 2.5 Hz, H-5), 7.27 (2H, d, *J* = 8.9 Hz, H-3, H-5′), 6.90 (1H, s, H-3), 5.12 (1H, d, *J* = 7.7 Hz, H-1″), 4.75 (1H, d, *J* = 0.7 Hz, 2″-OH), 4.47 (1H, d, *J* = 1.5 Hz, 3″-OH), 3.87 (1H, d, *J* = 11.5 Hz, H-6″), 3.84–3.82 (1H, m, 6″-OH), 3.73–3.70 (1H, m, H-6″), 3.66 (1H, t, *J* = 8.0 Hz, H-3″), 3.58 (3H, s, 4″-O-CH_3_), 3.56 (1H, d, *J* = 1.7 Hz, H-5″), 3.54–3.50 (1H, m, H-2″), 3.24 (1H, t, *J* = 9.3 Hz, H-4″); **^13^C NMR** (acetone-d_6_) δ (ppm): 176.14 (C=O), 164.06 (C-2), 161.82 (C-4′), 152.40 (C-8a), 137.19 (C-7), 131.44 (C-6), 129.11 (C-2′, C-6′), 126.65 (C-4a), 125.38 (C-1′), 124.88 (C-5), 117.81 (C-3′, C-5′), 113.75 (C-8), 106.48 (C-3), 101.16 (C-1″), 80.05 (C-4″), 77.96 (C-3″), 77.22 (C-5″), 74.86 (C-2″), 62.05 (C-6″), 60.59 (4″-O-CH_3_).

### 2.1. Microbial Transformation of 2′-Hydroxy-5′-Methyl-3′-Nitrochalcone (4) in the Culture of Entomopathogenic Filamentous Fungi

The compound 2′-hydroxy-5′-methyl-3′-nitrochalcone (**4**) was subjected to small-scale biotransformation by *B. bassiana* KCH J1.5, *I. fumosorosea* KCH J2, and *I. farinosa* KCH J2.6. A High-Pressure Liquid Chromatography (HPLC) analysis showed no flavonoid derivative products in the obtained extracts. 

### 2.2. Microbial Transformation of 6-Methyl-8-Nitroflavanone (5) in the Culture of I. fumosorosea KCH J2

**Figure 1:**
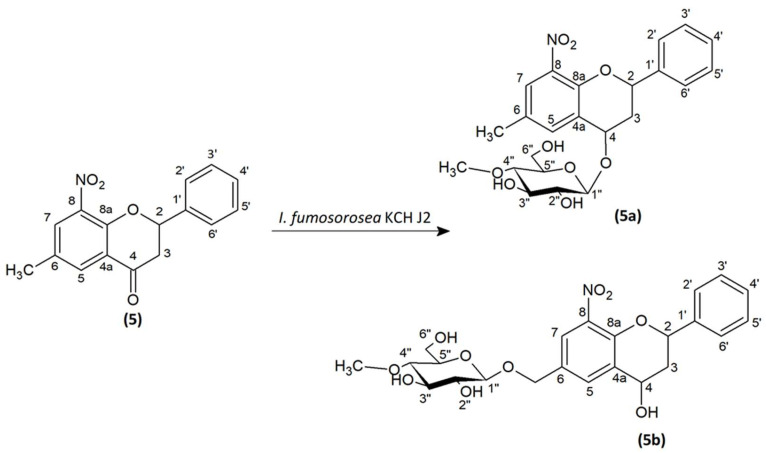
Biotransformation of 6-methyl-8-nitroflavanone (5) in the culture of I. fumosorosea KCH J2.

6-Methyl-8-nitroflavanone (**5**) was transformed microbiologically in the culture of *I. fumosorosea* KCH J2 (Figure 1). The process yielded two products 6-methyl-8-nitro-2-phenylchromane 4-*O-β*-D-(4″-*O*-methyl)-glucopyranoside (**5a**) with 5.3% yield (4.3 mg) and 8-nitroflavan-4-ol 6-methylene-*O-β*-D-(4″-*O*-methyl)-glucopyranoside (**5b**) with 14.8% yield (12.5 mg). The biotransformation took 10 days.

**Figure 2:**
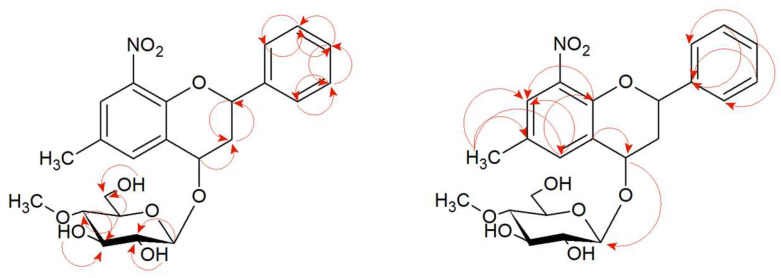
Key COSY (on the **left**) and HMBC (on the **right**) correlations of product (**5a**).

The structures of products (**5a**) and (**5b**) were determined by NMR spectroscopy using ^1^H NMR, ^13^C NMR, HMBC, and HSQC spectra and mass confirmation by LC-MS analysis (Supplementary Materials Figures S34–S80). In product 6-methyl-8-nitro-2-phenylchromane 4-*O-β*-D-(4″-*O*-methyl)-glucopyranoside (**5a**)**,** the presence of glucose in position C-4 is confirmed by the lack of a characteristic signal from carbonyl carbon in the ^13^C NMR spectrum as it was in a substrate (**5**). The NMR spectrum shows the signal of one proton at C-4, which proves the reduction of the carbonyl group at this position (δ = 5.06 ppm ^1^H NMR) and carbon δ = 69.42 ppm (^13^C NMR) (Supplementary Materials Figures S37 and S41). There is also a correlation between H-4 and C-1″ in the HMBC spectrum, which gives evidence that glucose is attached at the C-4 position (Supplementary Materials Figure S55). The characteristic single proton doublet H-1″ present in the ^1^H NMR at δ = 4.50 ppm with the coupling constant *J* = 7.7 Hz is evidence of *β*-configuration of the glucose. What is more, the singlet from three protons at δ = 3.54 ppm (^1^H NMR) and the presence of one signal from carbon at δ = 60.52 ppm (^13^C NMR) shows that glucose is *O*-methylated at the C-4″ position (Supplementary Materials Figures S37, S38 and S41). There are two signals at δ = 7.69 ppm and δ = 7.59 ppm, which come from protons at carbons C-7 and C-5 in A ring of the flavonoid part. It evidences the substitution of additional groups in positions C-6 and C-8. The position of -CH_3_ at C-6 confirms the correlation of the protons of this group with the C-5, C-6, and C-7 carbon in the HMBC spectrum (Supplementary Materials Figure S53) Moreover, a doublet of doublets at δ = 5.55 ppm corresponds to one proton in C-2 position and a doublet of triplets at δ = 2.6 ppm and a multiplet at δ = 2.15 ppm are from 3ax and 3eq protons in C-3 position which is characteristic of flavanone structure (Supplementary Materials Figures S49 and S51). Proton correlations through one bond (COSY) and multiple bonds (HMBC) are shown in Figure 2 and Supplementary Materials Figures S42–S46 and S52–S55.

**Figure 3:**
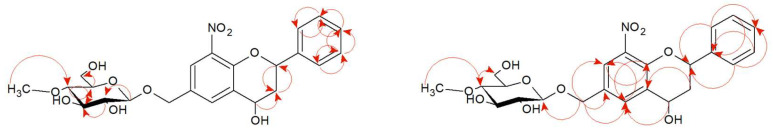
Key COSY (on the **left**) and HMBC (on the **right**) correlations of product (**5b**).

In the case of product (**5b**)**,** the presence of the one proton attached to carbon C-2 at δ = 5.49 ppm in the ^1^H NMR spectrum (carbon at δ = 75.24 ppm ^13^C NMR) and the two protons: H-3ax at δ = 2.37 ppm and H-3eq at δ = 2.20 ppm (carbon C-3 at δ = 38.83 ppm ^13^C NMR) indicates the structure of the flavanone. Moreover, the correlation of these protons is visible in the COSY spectrum (marked with an arrow—Supplementary Materials Figure S66). At δ = 4.90 ppm (^1^H NMR), there is a signal from a single proton suggesting a reduction of the carbonyl group at the C-4 position. There is also a dehydrogenation of the -CH_3_ group at the C-6 position to a -CH_2_- group (two characteristic proton doublets at δ = 4.90 ppm and 4.68 ppm) (Supplementary Materials Figure S71). In the HMBC spectrum is a correlation between these two protons with the carbons C-5, C-6, and C-7. Moreover, protons from the -CH_2_- group correlate with the carbon C-1″ from the glucose molecule confirming its attachment in that position (Supplementary Materials Figure S74). In the ^1^H NMR spectrum at δ = 4.41 ppm, *J* = 7.8 Hz is a one-proton doublet H-1″ from the anomeric carbon atom C-1″. It evidences the presence of glucose in *β*-configuration. At δ = 3.53 ppm in the ^1^H NMR is a signal from three protons from -CH_3_ group, which is attached to the oxygen in the C-4″ position at the glucose molecule (*O*-methylation) (Supplementary Materials Figure S71). Proton correlations through one bond (COSY), and multiple bonds (HMBC) are shown in Figure 3 and Supplementary Materials Figures S64–S68 and S73–S80.

### 2.3. Microbial Transformation of 6-Methyl-8-Nitroflavone (6) in the Culture of B. bassiana KCH J1.5

**Figure 4:**
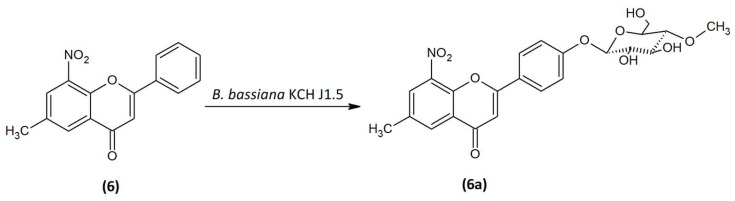
Biotransformation of 6-methyl-8-nitroflavone (**6**) in the culture of *B. bassiana* KCH J1.5.

As a result of the biotransformation of the 6-methyl-8-nitroflavone (**6**) in the culture of *B. bassiana* KCH J1.5, the 6-methyl-8-nitroflavone 4′-*O-β*-D-(4″-*O*-methyl)-glucopyranoside (**6a**) was formed (Figure 4). After seven days of biotransformation, 4.2 mg of the product (**6a**) was obtained (5%).

**Figure 5:**
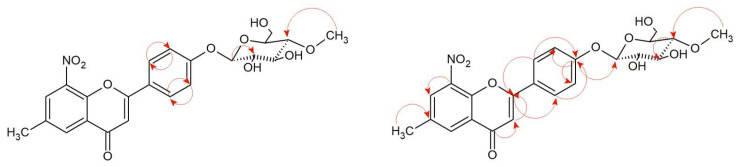
Key COSY (on the **left**) and HMBC (on the **right**) correlations of product (**6a**).

The structure of 6-methyl-8-nitroflavone 4′-*O-β*-D-(4″-*O*-methyl)-glucopyranoside (**6a**) was determined based on NMR spectroscopy and LC-MS analysis (Supplementary Materials Figures S98–S114). The carbon signal from C-1″ at δ = 101.14 ppm in ^13^C NMR spectrum with the characteristic attached proton doublet H-1” in the ^1^H NMR spectrum at δ = 5.12 ppm having the coupling constant *J* = 7.8 Hz indicates the *β*-configuration of the attached glucose molecule (Supplementary Materials Figures S101 and S104). In the HMBC spectrum, a correlation between carbon C-4′ from the ring B of flavonoid and the proton at the C-1″ indicates the substitution of sugar molecule at the C-4′ position (Supplementary Materials Figure S113). There is also a presence of the characteristic AA’BB’ coupling system with the signals from protons at C-2′ and C-6′ and signals from protons at C-3′ and C-5′, which confirms the para substitution. Moreover, the glucose is *O*-methylated in position C-4″ (Supplementary Materials Figure S100). A signal from the three protons from -O-CH_3_ group located at δ = 3.57 ppm (^1^H NMR) correlates with the carbon C-4″ (δ = 60.58 ppm, ^13^C NMR) in the HMBC spectrum (Supplementary Materials Figure S114). The carbonyl group remained intact, evidencing the characteristic signal at the ^13^C NMR at δ = 176.09 ppm, additionally, a signal from this carbon (C-4) is correlated with the one proton at C-3 carbon in the HMBC spectrum (Supplementary Materials Figures S104 and S113). The double bond between carbons C-2 and C-3 was not reduced, which confirms the correlation of C-2 with a proton at C-3 in the HMBC spectrum (Supplementary Materials: Figure S113). In Figure 5, the key COSY and HMBC correlations are shown.

### 2.4. Microbial Transformation of 3′-Bromo-5′-Chloro-2′-Hydroxychalcone (7) in the Culture of B. bassiana KCH J1.5 

**Figure 6:**
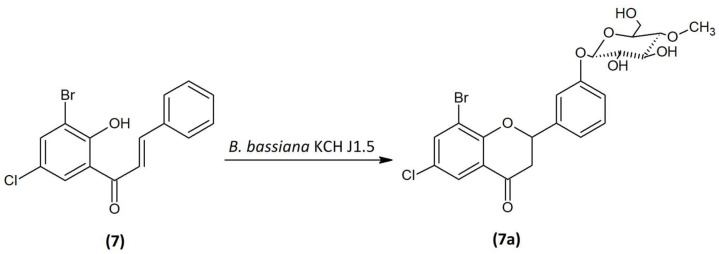
Biotransformation of 3′-bromo-5′-chloro-2′-hydroxychalcone (**7**) in the culture of *B. bassiana* KCH J1.5.

3′-Bromo-5′-chloro-2′-hydroxychalcone (**7**) was biotransformed by entomopathogenic fungi *B. bassiana* KCH J1.5 into 8-bromo-6-chloroflavanone 3′-*O-β*-D-(4″-*O*-methyl)-glucopyranoside (**7a**) with 6.24% yield (4.9 mg) (Figure 6). The process took 10 days. 

**Figure 7:**
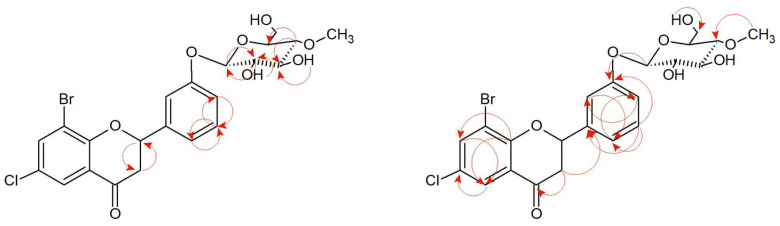
Key COSY (on the **left**) and HMBC (on the **right**) correlations of product (**7a**).

3′-Bromo-5′-chloro-2′-hydroxychalcone (**7**) was converted to the flavanone structure by the cyclization of the C ring and glycosylated at C-3′ position. The structure of the product was confirmed by NMR spectroscopy and LC-MS analysis (Supplementary Materials Figures S130–S150). The arrangement of signals from the protons belonging to the B ring of the flavonoid and their coupling in the COSY spectrum (Figure 7 and Supplementary Materials Figure S139) indicate the substitution of glucose at the C-3′ position. Another proof is also the correlation between the proton at C-1″ δ = 5.01 ppm (^1^H NMR) and the carbon C-3′ δ = 158.93 ppm (^13^C NMR) at the HMBC contour map (Supplementary Materials Figure S147). Two doublets in A ring of the flavonoid H-7 (δ = 7.91 ppm, *J* = 2.6 Hz) and H-5 (δ = 7.77 ppm, *J* = 2.5 Hz) evidence the presence of substitution in positions C-6 and C-8 (Supplementary Materials Figures S147–S149). In addition, there is a correlation between the carbonyl group (δ = 190.26 ppm, ^13^C NMR) and protons 3-axial and 3-equatorial (δ = 3.26 ppm and δ = 3.03, ^1^H NMR) at HMCB (Supplementary Materials Figure S147). The presence of the one-proton doublet H-1” at the anomeric carbon C-1″at δ = 5.01 ppm in the ^1^H NMR spectrum with the coupling constant *J* =7.8 Hz is characteristic of the *β*-configuration of glucose molecule (Supplementary Materials Figures S133 and S137). The attached glucose is *O*-methylated as evidenced by the presence of 4″-O-CH_3_ group in the ^1^H NMR δ = 3.56 ppm, and in the HMBC contour map, there is a correlation between the attached -CH_3_ group in the C-4″ position and the carbon 4″ (δ = 80.07 ppm, ^13^C NMR) (Figure 7 and Supplementary Materials Figure S150). Other proton–proton correlations COSY and proton–carbon correlations HMBC are shown in Figure 7.

### 2.5. Microbial Transformation of 8-Bromo-6-Chloroflavanone (8) in the Culture of I. fumosorosea KCH J2

**Figure 8:**
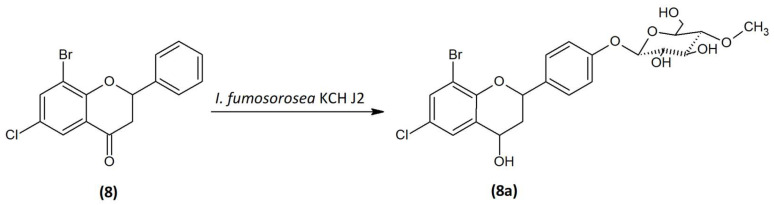
Biotransformation of 8-bromo-6-chloroflavanone (**8**) in the culture of *I. fumosorosea* KCH J2.

Microbial transformation of 8-bromo-6-chloroflavanone (**8**) in the culture of *I. fumosorosea* KCH J2 allowed receiving 8-bromo-6-chloroflavan-4-ol 4′-*O-β*-D-(4″-*O*-methyl)-glucopyranoside (**8a**) (Figure 8) with 2.67% yield (2.1mg). The process took 10 days.

**Figure 9:**
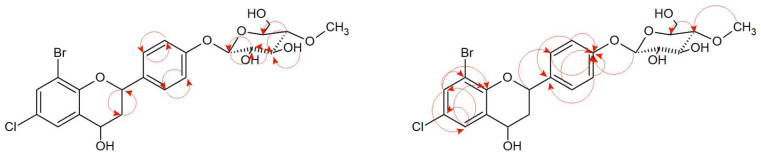
Key COSY (on the **left**) and HMBC (on the **right**) correlations of product (**8a**).

The analysis of NMR spectra confirmed the creation of the product (**8a**). The ^1^H NMR spectrum shows two doublets coming from protons H-7 (δ = 7.54 ppm, *J* = 2.5 Hz) and H-5 (δ = 7.40 ppm, *J* = 2.5 Hz), evidencing the substitution at C-8 and C-6 positions, as in the case of the substrate (**8**). The correlation is visible in the HMBC spectrum (Figure 9 and Supplementary Materials Figure S183). The characteristic AA′BB′ coupling system between signals from protons at C-2′ and C-6′ (δ = 7.45 ppm, *J* = 8.6 Hz), and protons from C-3′ and C-5′ (δ = 7.11 ppm, *J* = 8.7 Hz) suggesting additional substitution at the C-4′ position (Supplementary Materials Figure S169). In the ^13^C NMR, there is no characteristic signal from the carbonyl group at the C-4 position, but on ^1^H NMR, there is a signal from the proton in the C-4 position δ = 4.83 ppm (δ = 63.44 ppm, ^13^C NMR) which proves the reduction of the carbonyl group (Supplementary Materials Figure S170). According to HMBC spectra, there is a correlation between the proton in C-1″ and carbon C-4′, evidencing attachment of the glucose molecule in that position (Figure 9, Supplementary Materials Figure S183). The proton in anomeric carbon C-1″ δ = 4.98 ppm with the characteristic coupling constant *J* = 7.8 Hz visualized in the ^1^H NMR spectrum evidencing *β*-configuration of glucose. Additionally, the singlet coming from three protons at δ = 3.57 ppm (^1^H NMR) and the presence of one carbon at δ = 60.55 ppm (^13^C NMR) shows that glucose is *O*-methylated at C-4″ position (Supplementary Materials Figures S170, S180 and S181). In Figure 9, the key COSY and HMBC correlations are shown.

### 2.6. Microbial Transformation of 8-Bromo-6-Chloroflavone (9) in the Culture of I. farinosa KCH J2.6

**Figure 10:**
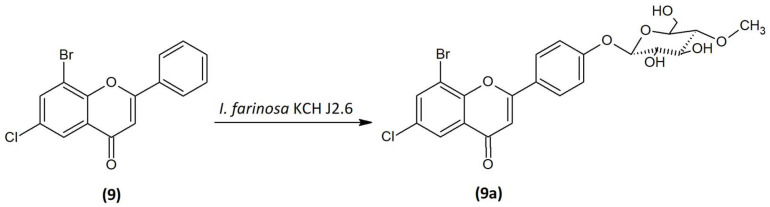
Biotransformation of 8-bromo-6-chloroflavone (**9**) in the culture of *I. farinosa* KCH J2.6.

The 8-bromo-6-chloroflavone (**9**) was biotransformed by enthomopathogenic fungi *I. farinosa* KCH J2.6 into 8-bromo-6-chloroflavone 4′-*O-β*-D-(4″-*O*-methyl)-glucopyranoside (**9a**) (Figure 10) with 19.59% yield (15.4 mg). The process took 10 days. 

**Figure 11:**
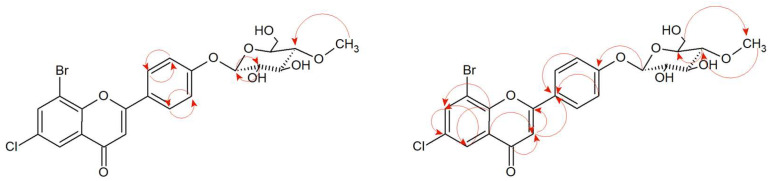
Key COSY (on the **left**) and HMBC (on the **right**) correlations of product (**9a**).

The obtained product (**9a**) was an *O*-methylglycosylated 8-bromo-6-chloroflavone derivative. The accurate NMR and LC-MS analysis confirming the structure and mass of the obtained product is presented in Supplementary Materials Figures S197–S213. The characteristic signals from the attached glucose molecule were shown in the ^1^H NMR and ^13^C NMR spectra (Supplementary Materials Figures S200 and S201 and Figure S204). The attachment of glucose to the 8-bromo-6-chloroflavone (**9**) was confirmed by the presence of a proton doublet from the proton H-1″ at the anomeric carbon C-1″ at δ = 5.12 ppm in the ^1^H NMR spectrum with the characteristic coupling constant *J* = 7.7 Hz (Supplementary Materials Figures S200). This coupling constant corresponds to the *β*-configuration of glucose. A singlet coming from three protons at δ = 3.58 ppm in the ^1^H NMR and the carbon at δ = 60.59 ppm in the ^13^C NMR spectrum gives proof of appearance of a -O-CH_3_ group. What’s more, this signal is correlated in the HMBC spectrum with the C-4″ signal at δ = 80.05 ppm, which proves the *O*-methylation at the C-4″ hydroxyl group of the glucose (Supplementary Materials Figures S201 and S213). The presence of the characteristic AA′BB′ coupling system in the flavonoid B ring from protons at C-2′ and C6′ and from the C-3′ and C-5′ confirmed the substitution at C-4′ (Figure 11, Supplementary Materials Figure S206). In the HMBC spectrum, a proton at the carbon C-1″ (δ = 5.12 ppm) derived from sugar moiety corresponding with the carbon C-4′ (δ = 161.82 ppm) coming from flavonoid part, proving substitution with the glucose molecule at this position (Figure 11, Supplementary Materials Figure S212). The presence of one-proton signals from C-5 and C-7 in the ^1^H NMR spectrum (Supplementary materials Figures S199 and S212) confirms the preservation of the arrangement of substituents at the C-8 and C-6 position as in the substrate (**9**). Other proton–proton correlations COSY and proton–carbon correlations HMBC are shown in Figure 11.

## 3. Discussion

Various modifications of compounds arising during the biotransformation process using entomopathogenic filamentous fungi are widely known [43,44,45].

In this study, in all cases where the strain *I. fumosorosea* KCH J2 was able to biotransform selected flavonoids, there has been a reduction of the carbonyl group in C-4 position. A similar reduction of the carbonyl group with glycosylation in C-4 position, like in the product (**5a**)**,** was observed for 6-methylflavanone [46]. The presence of hydroxyl group in C-4 position in the product (**5b**) was shown for 4′-methylflavanone in the biotransformation using *I. fumosorosea* KCH J2 [47]. Moreover, in the biotransformation of compound (**8**), there was also a reduction of the carbonyl group to -OH and glycosylation in C-4′ position. The reduction of the carbonyl group to -OH group was observed for 7-hydroxyflavanone, which was biotransformed using *Aspergillus niger* KB [48]. To conclude, a repeated tendency of this strain to reduce the carbonyl group can be seen in the case of flavanones. 

In the case of biotransformation of flavones, for the product (**6a**) obtained during biotransformation by *B. bassiana* KCH J1.5 and (**9a**) by *I. farinosa* KCH J2.6, there was only glycosylation in C-4′ position. *B. bassiana* is known for its ability to biotranform flavonoids [49]. The most common transformation is *O*-glycosylation [19,50]. In our case, we can also observe the *O*-glycosylation. According to the formation product (**9a**) by *I. farinosa* KCH J2.6, similar glycosylation in C-4′ position was also obtained via different strains of *I. farinosa* as a result of biotransformation of 3-methoxyflavone [51]. It confirms the ability for biotransformation of *I. farinosa* strains flavonoids with various substituents, which is important in searching for compounds with new properties. 

Only chalcone with bromine and chlorine atoms (**7**) was biotransformed by *B. bassiana* KCH J1.5. There was observed a formation of C ring creating flavanone with a glycosylation in C-3′ position. Interestingly, other studies showed glycosylation of chalcone at C-3′ position in a 2′-hydroxy-5′-methylchalcone [46]. It can suggest that in the first step of the process, a glycosylation in C-3′ occurred, and then, a cyclization of a ring, creating flavanone.

The 2′-hydroxy-5′-methyl-3′-nitrochalcone (**4**) was not biotransformed by any previously selected entomopathogenic filamentous fungi. Probably, the presence of a polar nitro group in the structure of a flavonoid compound makes it difficult to match this compound to the catalytic center of the enzyme. It is possible that the place of group substitution also affects this. Often, a slight change in the position of the substituent can result in a significant improvement in process yield or, on the contrary, no product formation.

## 4. Materials and Methods

### 4.1. Substrates

**Figure 12:**
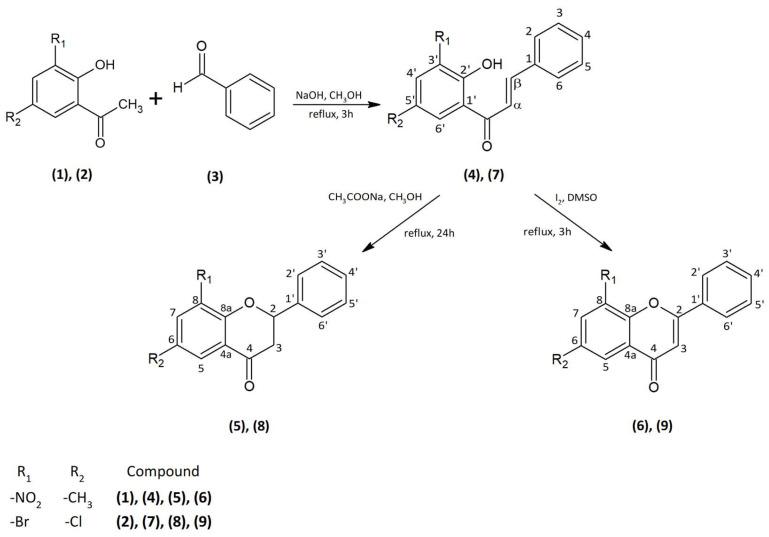
Synthesis of 2′-hydroxy-5′-methyl-3′-nitrochalcone (**4**), 6-methyl-8-nitroflavanone (**5**), 6-methyl-8-nitroflavone (**6**), 3′-bromo-5′-chloro-2′-hydroxychalcone (**7**), 8-bromo-6-chloroflavanone (**8**), and 8-bromo-6-chloroflavone (**9**).

The substrates 2′-hydroxy-5′-methyl-3′-nitrochalcone (**4**), 6-methyl-8-nitroflavanone (**5**), 6-methyl-8-nitroflavone (**6**), 3′-bromo-5′-chloro-2′-hydroxychalcone (**7**), 8-bromo-6-chloroflavanone (**8**), and 8-bromo-6-chloroflavone (**9**) were synthesized according to the reaction presented in Figure 12. The 2′-hydroxy-5′-methyl-3′-nitroacetophenone (**1**) and 3′-bromo-5′-chloro-2′-hydroxyacetophenone (**2**), and the benzaldehyde (**3**) were purchased from Sigma-Aldrich (St. Louis, MO, USA).

The first step was the synthesis of chalcones (**4**) and (**7**) carried out by the Claisen–Schmidt condensation (Figure 12). The reaction of the appropriately substituted acetophenone (**1**) and (**2**) with benzaldehyde (**3**) allowed to obtain 2′-hydroxy-5′-methyl-3′-nitrochalcone (**4**) (75% yield) and 3′-bromo-5′-chloro-2′-hydroxychalcone (**7**) (60% yield). Substrates to the synthesis were dissolved in methanol under alkaline conditions (NaOH) with the addition of water. The reaction was carried out for 3 h at the boiling point of the reactants under reflux. The second step was the cyclization of chalcones (**4**) and (**7**) into flavanones (**5**) (42% yield) and (**8**) (77% yield) using sodium acetate dissolved in methanol under reflux via 24 h (Figure 12). The last step was to obtain flavones (**6**) (90% yield) and (**9**) (94% yield) by reacting appropriate chalcones with iodine dissolved in dimethyl sulfoxide (DMSO) for 3 h under reflux at 125 °C in oil bath (Figure 12).

2′-Hydroxy-5′-methyl-3′-nitrochalcone (**4**). C_16_H_13_NO_4_, mp: 158 °C; HPLC Rt = 18.2 min; **^1^H NMR** (600 MHz, acetone-d_6_) δ (ppm): 13.50 (1H, s, -OH), 8.44 (1H, d, *J* = 1.6 Hz, H-6′), 8.05 (2H, dd, *J* = 8.6, 6.9 Hz, H-4′, H-α), 7.98 (1H, d, *J* = 15.5 Hz, H-β), 7.90–7.87 (2H, m, H-2, H-6), 7.53–7.48 (5H, m, H-3, H-4, H-5), 2.44 (3H, s, -CH_3_); **^13^C NMR** (acetone-d_6_) δ (ppm): 194.60 (C=O), 155.23 (C-1′), 147.59 (C-β), 139.16 (C-3′), 136.98 (C-6′), 135.50 (C-1), 132.23 (C-4), 132.14 (C-4′), 130.12 (C-2, C-6), 129.97 (C-3, C-5), 129.35 (C-5′), 123.82 (C-2′), 121.82 (C-α), 20.08 (-CH_3_)

6-Methyl-8-nitroflavanone (**5**). C_16_H_13_NO_4_, mp: 179–180 °C; HPLC Rt = 18.1 min; [α]_D_ = 0.108 (0.5 *w*/*v* % in acetone); **^1^H NMR** (600 MHz, acetone-d_6_) δ (ppm): 8.02 (1H, d, *J* = 1.9 Hz, H-7), 7.96–7.95 (1H, m, H-5), 7.61 (2H, d, *J* = 7.4 Hz, H-2′, H-6′), 7.47 (2H, t, *J* = 7.5 Hz, H-3′, H-5′), 7.41 (1H t, *J* = 7.4 Hz, H-4′), 5.87 (1H, dd, *J* = 12.7, 2.9 Hz, H-2), 3.30 (1H, dd, *J* = 16.9, 12.7 Hz, H-3_ax_), 3.07 (1H, dd, *J* = 16.9, 3.0 Hz, H-3_eq_), 2.44 (3H, s, -CH_3_); ^**13**^**C NMR** (acetone-d_6_) δ (ppm): 190.29 (C=O), 152.95 (C-6), 140.62 (C-8), 139.39 (C-1′), 132.30 (C-5), 132.09 (C-7), 131.87 (C-8a), 129.57 (C-3′, C-5′), 129.53 (C-4′), 127.10 (C-2′, C-6′), 123.97 (4a), 81.36 (C-2), 44.35 (C-3), 20.06 (-CH_3_)

6-Methyl-8-nitroflavone (**6**). C_16_H_11_NO_4_, mp: 219 °C; HPLC Rt = 17.1 min; **^1^H NMR** (600 MHz; acetone-d_6_) δ (ppm): 8.35 (1H, dd, *J* = 2.2, 0.5 Hz, H-7), 8.24 (1H, dd, *J* = 2.2, 0.8 Hz, H-5), 8.15 (2H, ddd, *J* = 5.7, 4.3, 2.5 Hz, H-2′, H-6′), 7.65–7.61 (3H, m, H-3′, H-4′, H-5′), 7.01 (1H, s, H-3), 2.59 (3H, s, -CH_3_); **^13^C NMR** (acetone-d_6_) δ (ppm): 176.23 (C=O), 163.90 (C-2), 147.56 (C-8a), 139.61 (C-8), 136.24 (C-6), 133.00 (C-4′), 131.92 (C-1′), 131.57 (C-5), 131.53 (C-7), 130.11 (C-3′, C-5′), 127.45 (C-2′, C-6′), 126.31 (C-4a), 107.97 (C-3), 20.60 (-CH_3_)

3′-Bromo-5′-chloro-2′-hydroxychalcone (**7**). C_15_H_10_BrClO_2_, mp: 133–135 °C; HPLC Rt = 19.9 min; **^1^H NMR** (600 MHz, acetone-d_6_) δ (ppm): 13.63 (1H, s, -OH), 8.40 (1H, d, *J* = 2.4 Hz, H-6′), 8.14 (1H, d, *J* = 15.4 Hz, H-α), 8.03 (1H, d, *J* = 15.4 Hz, H-β), 7.94 (2H, dd, *J* = 7.6, 1.7 Hz, H-2, H-6), 7.90 (1H, d, *J* = 2.5 Hz, H-4′), 7.54–7.48 (3H, m, H-3, H-4, H-5); **^13^C NMR** (acetone-d_6_) δ (ppm): 194.21 (C=O), 159.79 (C-1′), 148.25 (C-β), 139.40 (C-4′), 135.44 (C-1), 132.33 (C-4), 130.34 (C-2, C-6), 130.13 (C-6′), 129.94 (C-3, C-5), 124.46 (C-5′), 122.09 (C-2′), 120.75 (C-α), 113.07 (C-3′)

8-Bromo-6-chloroflavanone (**8**). C_15_H_10_BrClO_2_, mp: 117–119 °C; HPLC Rt = 18.6 min; [α]_D_ = -5.331 (0.325 w/v % in acetone); **^1^H NMR** (600 MHz, acetone-d_6_) δ (ppm): 7.91 (1H, d, *J* = 2.6 Hz, H-7), 7.77 (1H, d, *J* = 2.6 Hz, H-5), 7.64–7.61 (2H, m, H-2′, H-6′), 7.50–7.46 (2H, m, H-3′, H-5′), 7.44–7.40 (1H, m, H-4′), 5.84 (1H, dd, *J* = 12.7, 3.0 Hz, H-2), 3.27 (1H, dd, *J* = 16.9, 12.7 Hz, H-3_ax_), 3.03 (1H, dd, *J* = 16.9, 3.0 Hz, H-3_eq_); **^13^C NMR** (acetone-d_6_) δ (ppm): 190.27 (C=O), 157.66 (C-8a), 139.43 (C-1′), 138.81 (C-7), 129.61 (C-3′, C-4′, C-5′), 127.32 (C-6), 127.22 (C-2′, C-6′), 126.14 (C-5), 123.70 (C-4a), 113.58 (C-8),81.05 (C-2), 43.87 (C-3)

8-Bromo-6-chloroflavone (**9**). C_15_H_8_BrClO_2_, mp: 186–189 °C; HPLC Rt = 19.2 min; **^1^H NMR** (600 MHz, acetone-d_6_) δ (ppm): 8.19–8.17 (2H, m, H-2′, H-6′), 8.14 (1H, d, *J* = 2.5 Hz, H-7), 8.03 (1H, d, *J* = 2.5 Hz, H-5), 7.66–7.62 (3H, m, H-4′, H-3′, H-5′), 7.00 (1H, s, H-3); ^**13**^**C NMR** (acetone-d_6_) δ (ppm): 176.30 (C=O), 164.21 (C-2), 152.51 (C-8a), 137.38 (C-7), 133.05 (C-4′), 132.06 (C-1′), 131.56 (C-6), 130.16 (C-3′, C-5′), 127.43 (C-2′, C-6′), 126.69 (C-4a), 124.90 (C-5), 113.90 (C-8), 107.72 (C-3) 

### 4.2. Microorganisms

In the biotransformation process, three strains of entomopathogenic filamentous fungi *B. bassiana* KCH J1.5, *I. fumosorosea* KCH J2, and *I. farinosa* KCH J2.6 were used. The microorganisms belong to the Department of Food Chemistry and Biocatalysis of the Wrocław University of Environmental and Life Sciences in Poland. The methods of isolation of entomopathogenic filamentous fungi, reproduction, and genetic identification were described in our previous papers [23,52,53].

### 4.3. Analysis

Analytical and preparative TLC (Thin Layer Chromatography) was used to assess the course of synthesis as well as biotransformation and product isolation. Analytical TLC was used to monitor the course of chemical syntheses and the progress of biotransformation. For this purpose, TLC Silica gel 60/Kieselguhr F254 (0.2 mm thick) aluminum sheets 20 cm × 20 cm (Merck, Darmstadt, Germany) were used. The eluent consisted of cyclohexane (Chempur, Piekary Śląskie, Poland): Ethyl acetate (Chempur, Piekary Śląskie, Poland) in the ratio 9:1 and 4:1 *v*/*v* was used for the analysis of chemical syntheses. On the other hand, the mixture of chloroform (Chempur, Piekary Śląskie, Poland): Methanol (Chempur, Piekary Śląskie, Poland) in a ratio of 9:1 *v*/*v* was used to monitor the course of biotransformation. In both cases, the plates were observed under a UV lamp using a wavelength of λ = 254 nm and λ = 365 nm. Preparative TLC was used to separate the product mixture on scale-up biotransformation. For this purpose, preparative TLC Silica plates (Analtech, Gehrden, Germany) (0.5, 1, and 2 mm thick) were used with the eluent consisting of chloroform (Chempur, Piekary Śląskie, Poland) and methanol (Chempur, Piekary Śląskie, Poland) in the ratio (9:1 *v*/*v*). The products were observed under a UV lamp using a wavelength of λ = 254 nm and λ = 365 nm. Then, products were extracted thrice with 15 mL of ethyl acetate (Chempur, Piekary Śląskie, Poland). The extracts were filtered and evaporated in a vacuum evaporator.

HPLC chromatography was performed to check the biotransformation’s progress and determine the retention times of their substrates and products. Dionex Ultimate 3000 instrument (Thermo Fisher Scientific, Waltham, MA, USA) with a DAD-3000 diode array detector using an analytical octadecylsilica (ODS) 2 column (4.6 mm × 250 mm, Waters, Milford, MA, USA) and pre-column. The eluent was a mixture of 0.1% aqueous acid formic acid *v*/*v* (A) and acetonitrile (B). The gradient program was as follows: initial conditions—32.5% B in A, 4 min—40% B in A, 8 min—40% B in A, 10 min—45% B in A, 15 min—95% B in A, 18 min—95% B in A, 19 min—32.5% B in A, and 23 min—32.5% B in A. The flow rate was 1 mL/min, the injection volume was 5 µL, and the detection wavelength was 280 nm [22]. Data were collected using Chromeleon software version 7.2 (Thermo Fisher Scientific, Waltham, MA, USA).

For the NMR analysis of substrates and biotransformation products, ^1^H NMR, ^13^C NMR, COSY, HSQC, and HMBC spectra were performed using the DRX Avance^TM^ 600 MHz NMR spectrometer (Bruker, Billerica, MA, USA). All samples were dissolved in 0.7 mL of deuterated acetone.

The mass of the obtained biotransformation substrates and products was confirmed using LC-MS analysis, LC-MS 8045 SHIMADZU Triple Quadrupole Liquid Chromatograph Mass Spectrometer with electrospray ionization (ESI) source (Shimadzu, Kyoto, Japan). Analyses were conducted using method “product ion scan”. Only a specific ion with a known molecular mass (determined by previous NMR analysis) was searched in each sample with a pure compound. The separation was achieved on the Kinetex column (2.6 µm C18 100 Å, 100 mm × 3 mm, Phenomenex, Torrance, CA, USA) operated at 30 °C. The mobile phase was a mixture of 0.1% aqueous formic acid *v*/*v* (A) and acetonitrile (B). The flow rate was 0.4 mL/min, and the injection volume was 5 µL. The gradient program was as follows: Initial conditions—80% B in A, 6.5 min—100% B, 7 min—80% B in A. The principal operating parameters for the LC-MS were set as follows: nebulizing gas flow: 3 L/min, heating gas flow: 10 L/min, interface temperature: 300 °C, drying gas flow: 10 L/min, data acquisition range, *m*/*z* 100–1000 Da, positive ionization mode. Data were collected with LabSolutions version 5.97 (Shimadzu, Kyoto, Japan) software.

Optical rotation was measured using digital polarimeter P-2000-Na (ABL&E-JASCO, Kraków, Poland).

### 4.4. Small-Scale Biotransformation

Three entomopathogenic filamentous fungi *B. bassiana* KCH J1.5, *I. fumosorosea* KCH J2, and *I. farinosa* KCH J2.6 were used for small-scale biotransformation. Each of the six substrates underwent biotransformation by all the microorganisms above-mentioned. These fungi have been chosen for their ability to biotransform flavonoid compounds (production of glycoside derivatives) based on previous screening studies conducted by our team in the Department of Food Chemistry and Biocatalysis [47,54]. The purpose of this part of the research was to select the appropriate microorganism and biotransformation time of substrates (**4**), (**5**), (**6**), (**7**), (**8**), and (**9**) for further scale-up studies.

Small-scale biotransformation was carried out in 300 mL flat-bottomed conical flasks (Erlenmeyer flasks) containing 100 mL of modified Sabouraud medium (1% aminobac, 3% sucrose per 1 L of water). The flasks were inoculated with about 1 mL of a culture of entomopathogenic filamentous fungi and then shaken for 72 h at 140 rpm and 25 °C. Next, 10 mg of the substrate was added to each flask and shaken again at 140 rpm at 25 °C. Samples were collected after 3, 7, and 10 days of the experiment. After that, samples were extracted with ethyl acetate in the ratio of 1:1 ethyl acetate: medium, and collected in a separate flask. Extracts were dried with magnesium sulfate (MgSO_4_), filtered, and concentrated in a vacuum evaporator. Samples for HPLC analysis from obtained extracts were prepared. The dried extracts were suspended in 1 mL of acetonitrile and analyzed by HPLC. The results of this analysis made it possible to select the appropriate biotransformation time and strain for the increased scale of the experiment so that the process would run as efficiently as possible.

### 4.5. Scale-Up Biotransformation

The scale-up biotransformation was carried out in a 2-L flask containing 500 mL of modified Sabouraud’s medium (same as for small scale). The scale-up biotransformation was used to obtain more products for further analysis. 

To the flask with a prepared sterile medium 1 mL of preincubation culture of entomopathogenic filamentous fungi was added. Then, the flask was incubated for 72 h at 25 °C with shaking at 145 rpm. After this time, 50 mg of substrate dissolved in 2 mL of DMSO was added to the flask. The time of scale-up biotransformation was based on previous small-scale studies for a given microorganism and substrate. The next step was to extract the obtained products three times using 350 mL of ethyl acetate. The combined extracts were then dried with MgSO_4_, filtered, and evaporated to dryness. The obtained samples were separated using preparative TLC plates. For this purpose, the extract was dissolved in approx. two mL of THF and applied to the plate. In a chromatography chamber, the products were separated using chloroform: methanol mixture (9:1, *v*/*v*) as the eluent. TLC plates were visualized under a UV lamp (254 nm and 365 nm), and separated fractions were removed from the plate. Fractions were extracted three times with 15 mL ethyl acetate for 30 min. The extracts were filtered and evaporated to dryness in a vacuum evaporator. Then, the fractions prepared this way were dissolved in 0.7 mL of deuterated acetone and subjected to NMR analysis.

NMR analyses are presented in Supplementary Materials.

## 5. Conclusions

In this paper, we present the microbial transformations of chalcones (**4**), (**7**), flavanones (**5**), (**8**), and flavones (**6**), (**9**) using entomopathogenic filamentous fungi. 

*B. bassiana* KCH J1.5, *I. fumosorosea* KCH J2, and *I. farinosa* KCH J2.6 strains did not transform the 2′-hydroxy-5′-methyl-3′-nitrochalcone (**4**). 

*B. bassiana* KCH J1.5 strain was able to transform 3′-bromo-5′-chloro-2′-hydroxychalcone (**7**). In this case, the chalcone was cyclized to flavanone, and the glycosidic unit was attached at the C-3’ position creating an 8-bromo-6-chloroflavanone 3′-*O-β*-D-(4″-*O*-methyl)-glucopyranoside (**7a**). 

Moreover, flavanones (**5**) and (**8**) were only effectively biotransformed by entomopathogenic filamentous fungi *I. fumosorosea* KCH J2. The carbonyl groups of both substrates were reduced. During the biotransformation of 6-methyl-8-nitroflavanone (**5**), the carbonyl group was reduced with the attachment of a glycosidic unit at the C-4 position (6-methyl-8-nitro-2-phenylchromane 4-*O-β*-D-(4″-*O*-methyl)-glucopyranoside (**5a**)), and in the product (5b) the sugar unit has become attached to the -CH_3_ group at the C-6 position (8-nitroflavan-4-ol 6-methylene-*O-β*-D-(4″-*O*-methyl)-glucopyranoside (**5b**)). 

In the case of the transformation of flavanones, glucose was attached at the C-4′ position. For compound (**6**), the most efficient reaction was carried out by *B. bassiana* KCH J1.5, and for compound (**9**) by *I. farinosa* KCH J2.6.

The presented research results show the glycosylation capacity of flavonoids with various substituents by entomopathogenic filamentous fungi. All biotransformation products have not been previously described in the scientific literature. We aim at further studies to assess their biological properties and bioavailability.

## Data Availability

Samples of the compounds **1–9** and **5a**, **5b**, **6a**, **7a**, **8a**, and **9a** are available from the authors.

## Supplementary Materials

The following supporting information can be downloaded at: https://www.mdpi.com/article/10.3390/ijms24119500/s1.
