# Supplementary material for: Biotransformation of Flavonoids with -NO2, -CH3 Groups and -Br, -Cl Atoms by Entomopathogenic Filamentous Fungi
Source: Int J Mol Sci. 2023 May 30;24(11):9500. doi: 10.3390/ijms24119500 (PMC10254066; doi:10.3390/ijms24119500)
Supplement: Supplementary file 1 [file ijms-24-09500-s001.zip › ijms-2403650-supplementary.pdf]

Supplementary Materials

Biotransformation of Flavonoids with-NO_2,-CH_3 Groups and-Br,-Cl Atoms by Entomopathogenic Filamentous Fungi

Martyna Perz*, Agnieszka Krawczyk-Łebek, Monika Dymarska, Tomasz Janeczko, Edyta Kostrzewa-Susłow*

Department of Food Chemistry and Biocatalysis, Faculty of Biotechnology and Food Science, Wrocław University of Environmental and Life Sciences, 50-375 Wrocław, Poland.
*Correspondence: martyna.perz@upwr.edu.pl; edyta.kostrzewa-suslow@upwr.edu.pl

Content:

Figure S1. MS analysis of 2'-hydroxy-5'-methyl-3'-nitrochalcone (4)
Figure S2. ^{1}H NMR spectrum of 2'-hydroxy-5'-methyl-3'-nitrochalcone (4)
Figure S3. ^{1}H NMR spectrum expansion of 2'-hydroxy-5'-methyl-3'-nitrochalcone (4)
Figure S4. ^{1}H NMR spectrum expansion of 2'-hydroxy-5'-methyl-3'-nitrochalcone (4)
Figure S5. ^{13}C NMR spectrum of 2'-hydroxy-5'-methyl-3'-nitrochalcone (4)
Figure S6. ^{13}C NMR spectrum expansion of 2'-hydroxy-5'-methyl-3'-nitrochalcone (4)
Figure S7. ^{13}C NMR spectrum expansion of 2'-hydroxy-5'-methyl-3'-nitrochalcone (4)
Figure S8. COSY contour map – ^{1}H x ^{1}H of 2'-hydroxy-5'-methyl-3'-nitrochalcone (4)
Figure S9. COSY contour map – ^{1}H x ^{1}H expansion of 2'-hydroxy-5'-methyl-3'-nitrochalcone (4)
Figure S10. COSY contour map – ^{1}H x ^{1}H of 2'-hydroxy-5'-methyl-3'-nitrochalcone (4)
Figure S11. HSQC contour map – ^{1}H x ^{13}C of 2'-hydroxy-5'-methyl-3'-nitrochalcone (4)
Figure S12. HSQC contour map – ^{1}H x ^{13}C expansion of 2'-hydroxy-5'-methyl-3'-nitrochalcone (4)
Figure S13. HSQC contour map – ^{1}H x ^{13}C expansion of 2'-hydroxy-5'-methyl-3'-nitrochalcone (4)
Figure S14. HMBC contour map – ^{1}H x ^{13}C of 2'-hydroxy-5'-methyl-3'-nitrochalcone (4)
Figure S15. HMBC contour map – ^{1}H x ^{13}C expansion of 2'-hydroxy-5'-methyl-3'-nitrochalcone (4)
Figure S16. HMBC contour map – ^{1}H x ^{13}C expansion of 2'-hydroxy-5'-methyl-3'-nitrochalcone (4)
Figure S17. HMBC contour map – ^{1}H x ^{13}C expansion of 2'-hydroxy-5'-methyl-3'-nitrochalcone (4)
Figure S18. MS analysis of 6-methyl-8-nitroflavanone (5)
Figure S19. ^{1}H NMR spectrum of 6-methyl-8-nitroflavanone (5)
Figure S20. ^{1}H NMR spectrum expansion of 6-methyl-8-nitroflavanone (5)
Figure S21. ^{1}H NMR spectrum expansion of 6-methyl-8-nitroflavanone (5)
Figure S22. ^{13}C NMR spectrum of 6-methyl-8-nitroflavanone (5)
Figure S23. ^{13}C NMR spectrum expansion of 6-methyl-8-nitroflavanone (5)
Figure S24. ^{13}C NMR spectrum expansion of 6-methyl-8-nitroflavanone (5)
Figure S25. COSY contour map – ^{1}H x ^{1}H of 6-methyl-8-nitroflavanone (5)
Figure S26. COSY contour map – ^{1}H x ^{1}H expansion of 6-methyl-8-nitroflavanone (5)
Figure S27. COSY contour map – ^{1}H x ^{1}H expansion of 6-methyl-8-nitroflavanone (5)
Figure S28. HSQC contour map – ^{1}H x ^{13}C of 6-methyl-8-nitroflavanone (5)
Figure S29. HSQC contour map – ^{1}H x ^{13}C expansion of 6-methyl-8-nitroflavanone (5)
Figure S30. HSQC contour map – ^{1}H x ^{13}C expansion of 6-methyl-8-nitroflavanone (5)

Figure S31. HMBC contour map – ^{1}H x ^{13}C of 6-methyl-8-nitroflavanone (5)
Figure S32. HMBC contour map – ^{1}H x ^{13}C expansion of 6-methyl-8-nitroflavanone (5)
Figure S33. HMBC contour map – ^{1}H x ^{13}C expansion of 6-methyl-8-nitroflavanone (5)
Figure S34. MS analysis of 6-methyl-8-nitro-2-phenylchromane 4-*O*-*β*-D-(4”-*O*-methyl)-glucopyranoside (5a)
Figure S35. ^{1}H NMR spectrum of 6-methyl-8-nitro-2-phenylchromane 4-*O*-*β*-D-(4”-*O*-methyl)-glucopyranoside (5a)
Figure S36. ^{1}H NMR spectrum expansion of 6-methyl-8-nitro-2-phenylchromane 4-*O*-*β*-D-(4”-*O*-methyl)-glucopyranoside (5a)
Figure S37. ^{1}H NMR spectrum expansion of 6-methyl-8-nitro-2-phenylchromane 4-*O*-*β*-D-(4”-*O*-methyl)-glucopyranoside (5a)
Figure S38. ^{1}H NMR spectrum expansion of 6-methyl-8-nitro-2-phenylchromane 4-*O*-*β*-D-(4”-*O*-methyl)-glucopyranoside (5a)
Figure S39. ^{13}C NMR spectrum of 6-methyl-8-nitro-2-phenylchromane 4-*O*-*β*-D-(4”-*O*-methyl)-glucopyranoside (5a)
Figure S40. ^{13}C NMR spectrum expansion of 6-methyl-8-nitro-2-phenylchromane 4-*O*-*β*-D-(4”-*O*-methyl)-glucopyranoside (5a)
Figure S41. ^{13}C NMR spectrum expansion of 6-methyl-8-nitro-2-phenylchromane 4-*O*-*β*-D-(4”-*O*-methyl)-glucopyranoside (5a)
Figure S42. COSY contour map – ^{1}H x ^{13}C of 6-methyl-8-nitro-2-phenylchromane 4-*O*-*β*-D-(4”-*O*-methyl)-glucopyranoside (5a)
Figure S43. COSY contour map – ^{1}H x ^{13}C expansion of 6-methyl-8-nitro-2-phenylchromane 4-*O*-*β*-D-(4”-*O*-methyl)-glucopyranoside (5a)
Figure S44. COSY contour map – ^{1}H x ^{13}C expansion of 6-methyl-8-nitro-2-phenylchromane 4-*O*-*β*-D-(4”-*O*-methyl)-glucopyranoside (5a)
Figure S45. COSY contour map – ^{1}H x ^{13}C expansion of 6-methyl-8-nitro-2-phenylchromane 4-*O*-*β*-D-(4”-*O*-methyl)-glucopyranoside (5a)
Figure S46. COSY contour map – ^{1}H x ^{13}C expansion of 6-methyl-8-nitro-2-phenylchromane 4-*O*-*β*-D-(4”-*O*-methyl)-glucopyranoside (5a)
Figure S47. HSQC contour map – ^{1}H x ^{13}C of 6-methyl-8-nitro-2-phenylchromane 4-O-β-D-(4”-O-methyl)-glucopyranoside (5a)
Figure S48. HSQC contour map – ^{1}H x ^{13}C expansion of 6-methyl-8-nitro-2-phenylchromane 4-*O*-*β*-D-(4”-*O*-methyl)-glucopyranoside (5a)
Figure S49. HSQC contour map – ^{1}H x ^{13}C expansion of 6-methyl-8-nitro-2-phenylchromane 4-*O*-*β*-D-(4”-*O*-methyl)-glucopyranoside (5a)
Figure S50. HSQC contour map – ^{1}H x ^{13}C expansion of 6-methyl-8-nitro-2-phenylchromane 4-*O*-*β*-D-(4”-*O*-methyl)-glucopyranoside (5a)
Figure S51. HSQC contour map – ^{1}H x ^{13}C expansion of 6-methyl-8-nitro-2-phenylchromane 4-*O*-*β*-D-(4”-*O*-methyl)-glucopyranoside (5a)
Figure S52. HMBC contour map – ^{1}H x ^{13}C of 6-methyl-8-nitro-2-phenylchromane 4-*O*-*β*-D-(4”-*O*-methyl)-glucopyranoside (5a)
Figure S53. HMBC contour map – ^{1}H x ^{13}C expansion of 6-methyl-8-nitro-2-phenylchromane 4-*O*-*β*-D-(4”-*O*-methyl)-glucopyranoside (5a)
Figure S54. HMBC contour map – ^{1}H x ^{13}C expansion of 6-methyl-8-nitro-2-phenylchromane 4-*O*-*β*-D-(4”-*O*-methyl)-glucopyranoside (5a)
Figure S55. HMBC contour map – ^{1}H x ^{13}C expansion of 6-methyl-8-nitro-2-phenylchromane 4-O-β-D-(4”-O-methyl)-glucopyranoside (5a)
Figure S56. MS analysis of 8-nitroflavan-4-ol 6-methylene-*O*-*β*-D-(4”-*O*-methyl)-glucopyranoside (5b)

Figure S57. ^{1}H NMR spectrum of 8-nitroflavan-4-ol 6-methylene-*O*-*β*-D-(4”-*O*-methyl)-glucopyranoside (5b)
Figure S58. ^{1}H NMR spectrum expansion of 8-nitroflavan-4-ol 6-methylene-*O*-*β*-D-(4”-*O*-methyl)-glucopyranoside (5b)
Figure S59. ^{1}H NMR spectrum expansion of 8-nitroflavan-4-ol 6-methylene-*O*-*β*-D-(4”-*O*-methyl)-glucopyranoside (5b)
Figure S60. ^{1}H NMR spectrum expansion of 8-nitroflavan-4-ol 6-methylene-*O*-*β*-D-(4”-*O*-methyl)-glucopyranoside (5b)
Figure S61. ^{13}C NMR spectrum of 8-nitroflavan-4-ol 6-methylene-*O*-*β*-D-(4”-*O*-methyl)-glucopyranoside (5b)
Figure S62. ^{13}C NMR spectrum expansion of 8-nitroflavan-4-ol 6-methylene-*O*-*β*-D-(4”-*O*-methyl)-glucopyranoside (5b)
Figure S63. ^{13}C NMR spectrum expansion of 8-nitroflavan-4-ol 6-methylene-*O*-*β*-D-(4”-*O*-methyl)-glucopyranoside (5b)
Figure S64. COSY contour map – ^{1}H x ^{1}H of 8-nitroflavan-4-ol 6-methylene-*O*-*β*-D-(4”-*O*-methyl)-glucopyranoside (5b)
Figure S65. COSY contour map – ^{1}H x ^{1}H expansion of 8-nitroflavan-4-ol 6-methylene-*O*-*β*-D-(4”-*O*-methyl)-glucopyranoside (5b)
Figure S66. COSY contour map – ^{1}H x ^{1}H expansion of 8-nitroflavan-4-ol 6-methylene-*O*-*β*-D-(4”-*O*-methyl)-glucopyranoside (5b)
Figure S67. COSY contour map – ^{1}H x ^{1}H expansion of 8-nitroflavan-4-ol 6-methylene-*O*-*β*-D-(4”-*O*-methyl)-glucopyranoside (5b)
Figure S68. COSY contour map – ^{1}H x ^{1}H expansion of 8-nitroflavan-4-ol 6-methylene-*O*-*β*-D-(4”-*O*-methyl)-glucopyranoside (5b)
Figure S69. HSQC contour map – ^{1}H x ^{13}C of 8-nitroflavan-4-ol 6-methylene-*O*-*β*-D-(4”-*O*-methyl)-glucopyranoside (5b)
Figure S70. HSQC contour map – ^{1}H x ^{13}C expansion of 8-nitroflavan-4-ol 6-methylene-*O*-*β*-D-(4”-*O*-methyl)-glucopyranoside (5b)
Figure S71. HSQC contour map – ^{1}H x ^{13}C expansion of 8-nitroflavan-4-ol 6-methylene-*O*-*β*-D-(4”-*O*-methyl)-glucopyranoside (5b)
Figure S72. HSQC contour map – ^{1}H x ^{13}C expansion of 8-nitroflavan-4-ol 6-methylene-*O*-*β*-D-(4”-*O*-methyl)-glucopyranoside (5b)
Figure S73. HMBC contour map – ^{1}H x ^{13}C of 8-nitroflavan-4-ol 6-methylene-*O*-*β*-D-(4”-*O*-methyl)-glucopyranoside (5b)
Figure S74. HMBC contour map – ^{1}H x ^{13}C expansion of 8-nitroflavan-4-ol 6-methylene-*O*-*β*-D-(4”-*O*-methyl)-glucopyranoside (5b)
Figure S75. HMBC contour map – ^{1}H x ^{13}C expansion of 8-nitroflavan-4-ol 6-methylene-*O*-*β*-D-(4”-*O*-methyl)-glucopyranoside (5b)
Figure S76. HMBC contour map – ^{1}H x ^{13}C expansion of 8-nitroflavan-4-ol 6-methylene-*O*-*β*-D-(4”-*O*-methyl)-glucopyranoside (5b)
Figure S77. HMBC contour map – ^{1}H x ^{13}C expansion of 8-nitroflavan-4-ol 6-methylene-*O*-*β*-D-(4”-*O*-methyl)-glucopyranoside (5b)
Figure S78. HMBC contour map – ^{1}H x ^{13}C expansion of 8-nitroflavan-4-ol 6-methylene-*O*-*β*-D-(4”-*O*-methyl)-glucopyranoside (5b)
Figure S79. HMBC contour map – ^{1}H x ^{13}C expansion of 8-nitroflavan-4-ol 6-methylene-*O*-*β*-D-(4”-*O*-methyl)-glucopyranoside (5b)
Figure S80. HMBC contour map – ^{1}H x ^{13}C expansion of 8-nitroflavan-4-ol 6-methylene-*O*-*β*-D-(4”-*O*-methyl)-glucopyranoside (5b)

Figure S81. MS analysis of 6-methyl-8-nitroflavone (6)
Figure S82. ^{1}H NMR spectrum of 6-methyl-8-nitroflavone (6)
Figure S83. ^{1}H NMR spectrum expansion of 6-methyl-8-nitroflavone (6)
Figure S84. ^{1}H NMR spectrum expansion of 6-methyl-8-nitroflavone (6)
Figure S85. ^{13}C NMR spectrum of 6-methyl-8-nitroflavone (6)
Figure S86. ^{13}C NMR spectrum expansion of 6-methyl-8-nitroflavone (6)
Figure S87. ^{13}C NMR spectrum expansion of 6-methyl-8-nitroflavone (6)
Figure S88. COSY contour map – ^{1}H x ^{1}H of 6-methyl-8-nitroflavone (6)
Figure S89. COSY contour map – ^{1}H x ^{1}H expansion of 6-methyl-8-nitroflavone (6)
Figure S90. COSY contour map – ^{1}H x ^{1}H expansion of 6-methyl-8-nitroflavone (6)
Figure S91. HSQC contour map – ^{1}H x ^{13}C of 6-methyl-8-nitroflavone (6)
Figure S92. HSQC contour map – ^{1}H x ^{13}C expansion of 6-methyl-8-nitroflavone (6)
Figure S93. HSQC contour map – ^{1}H x ^{13}C expansion of 6-methyl-8-nitroflavone (6)
Figure S94. HMBC contour map – ^{1}H x ^{13}C of 6-methyl-8-nitroflavone (6)
Figure S95. HMBC contour map – ^{1}H x ^{13}C expansion of 6-methyl-8-nitroflavone (6)
Figure S96. HMBC contour map – ^{1}H x ^{13}C expansion of 6-methyl-8-nitroflavone (6)
Figure S97. HMBC contour map – ^{1}H x ^{13}C expansion of 6-methyl-8-nitroflavone (6)
Figure S98. MS analysis of 6-methyl-8-nitroflavone 4’-*O*-*β*-D-(4”-*O*-methyl)-glucopyranoside (6a)
Figure S99. ^{1}H NMR spectrum of 6-methyl-8-nitroflavone 4’-*O*-*β*-D-(4”-*O*-methyl)-glucopyranoside (6a)
Figure S100. ^{1}H NMR spectrum expansion of 6-methyl-8-nitroflavone 4’-*O*-*β*-D-(4”-*O*-methyl)-glucopyranoside (6a)
Figure S101. ^{1}H NMR spectrum expansion of 6-methyl-8-nitroflavone 4’-*O*-*β*-D-(4”-*O*-methyl)-glucopyranoside (6a)
Figure S102. ^{1}H NMR spectrum expansion of 6-methyl-8-nitroflavone 4’-*O*-*β*-D-(4”-*O*-methyl)-glucopyranoside (6a)
Figure S103. ^{13}C NMR spectrum of 6-methyl-8-nitroflavone 4’-*O*-*β*-D-(4”-*O*-methyl)-glucopyranoside (6a)
Figure S104. ^{13}C NMR spectrum expansion of 6-methyl-8-nitroflavone 4’-*O*-*β*-D-(4”-*O*-methyl)-glucopyranoside (6a)
Figure S105. ^{13}C NMR spectrum expansion of 6-methyl-8-nitroflavone 4’-*O*-*β*-D-(4”-*O*-methyl)-glucopyranoside (6a)
Figure S106. COSY contour map – ^{1}H x ^{1}H of 6-methyl-8-nitroflavone 4’-*O*-*β*-D-(4”-*O*-methyl)-glucopyranoside (6a)
Figure S107. COSY contour map – ^{1}H x ^{1}H expansion of 6-methyl-8-nitroflavone 4’-*O*-*β*-D-(4”-*O*-methyl)-glucopyranoside (6a)
Figure S108. COSY contour map – ^{1}H x ^{1}H expansion of 6-methyl-8-nitroflavone 4’-*O*-*β*-D-(4”-*O*-methyl)-glucopyranoside (6a)
Figure S109. HSQC contour map – ^{1}H x ^{13}C of 6-methyl-8-nitroflavone 4’-*O*-*β*-D-(4”-*O*-methyl)-glucopyranoside (6a)
Figure S110. HSQC contour map – ^{1}H x ^{13}C expansion of 6-methyl-8-nitroflavone 4’-*O*-*β*-D-(4”-*O*-methyl)-glucopyranoside (6a)
Figure S111. HSQC contour map – ^{1}H x ^{13}C expansion of 6-methyl-8-nitroflavone 4’-*O*-*β*-D-(4”-*O*-methyl)-glucopyranoside (6a)
Figure S112. HMBC contour map – ^{1}H x ^{13}C of 6-methyl-8-nitroflavone 4’-*O*-*β*-D-(4”-*O*-methyl)-glucopyranoside (6a)
Figure S113. HMBC contour map – ^{1}H x ^{13}C expansion of 6-methyl-8-nitroflavone 4’-*O*-*β*-D-(4”-*O*-methyl)-glucopyranoside (6a)

Figure S114. HMBC contour map – ^{1}H x ^{13}C expansion of 6-methyl-8-nitroflavone 4’-*O*-*β*-D-(4”-*O*-methyl)-glucopyranoside (6a)
Figure S115. MS analysis of 3’-bromo-5’-chloro-2’-hydroxychalcone (7)
Figure S116. ^{1}H NMR spectrum of 3’-bromo-5’-chloro-2’-hydroxychalcone (7)
Figure S117. ^{1}H NMR spectrum expansion of 3’-bromo-5’-chloro-2’-hydroxychalcone (7)
Figure S118. ^{1}H NMR spectrum expansion of 3’-bromo-5’-chloro-2’-hydroxychalcone (7)
Figure S119. ^{13}C NMR spectrum of 3’-bromo-5’-chloro-2’-hydroxychalcone (7)
Figure S120. ^{13}C NMR spectrum expansion of 3’-bromo-5’-chloro-2’-hydroxychalcone (7)
Figure S121. COSY contour map – ^{1}H x ^{1}H of 3’-bromo-5’-chloro-2’-hydroxychalcone (7)
Figure S122. COSY contour map – ^{1}H x ^{1}H expansion of 3’-bromo-5’-chloro-2’-hydroxychalcone (7)
Figure S123. COSY contour map – ^{1}H x ^{1}H expansion of 3’-bromo-5’-chloro-2’-hydroxychalcone (7)
Figure S 124. HSQC contour map – ^{1}H x ^{13}C of 3’-bromo-5’-chloro-2’-hydroxychalcone (7)
Figure S125. HSQC contour map – ^{1}H x ^{13}C expansion of 3’-bromo-5’-chloro-2’-hydroxychalcone (7)
Figure S126. HSQC contour map – ^{1}H x ^{13}C expansion of 3’-bromo-5’-chloro-2’-hydroxychalcone (7)
Figure S127. HMBC contour map – ^{1}H x ^{13}C of 3’-bromo-5’-chloro-2’-hydroxychalcone (7)
Figure S128. HMBC contour map – ^{1}H x ^{13}C expansion of 3’-bromo-5’-chloro-2’-hydroxychalcone (7)
Figure S129. HMBC contour map – ^{1}H x ^{13}C expansion of 3’-bromo-5’-chloro-2’-hydroxychalcone (7)
Figure S130. MS analysis of 8-bromo-6-chloroflavanone 3’-*O*-*β*-D-(4”-*O*-methyl)-glucopyranoside (7a)
Figure S131. ^{1}H NMR spectrum of 8-bromo-6-chloroflavanone 3’-*O*-*β*-D-(4”-*O*-methyl)-glucopyranoside (7a)
Figure S132. ^{1}H NMR spectrum expansion of 8-bromo-6-chloroflavanone 3’-*O*-*β*-D-(4”-*O*-methyl)-glucopyranoside (7a)
Figure S133. ^{1}H NMR spectrum expansion of 8-bromo-6-chloroflavanone 3’-*O*-*β*-D-(4”-*O*-methyl)-glucopyranoside (7a)
Figure S134. ^{1}H NMR spectrum expansion of 8-bromo-6-chloroflavanone 3’-*O*-*β*-D-(4”-*O*-methyl)-glucopyranoside (7a)
Figure S135. ^{13}C NMR spectrum of 8-bromo-6-chloroflavanone 3’-*O*-*β*-D-(4”-*O*-methyl)-glucopyranoside (7a)
Figure S136. ^{13}C NMR spectrum expansion of 8-bromo-6-chloroflavanone 3’-*O*-*β*-D-(4”-*O*-methyl)-glucopyranoside (7a)
Figure S137. ^{13}C NMR spectrum expansion of 8-bromo-6-chloroflavanone 3’-*O*-*β*-D-(4”-*O*-methyl)-glucopyranoside (7a)
Figure S138. COSY contour map – ^{1}H x ^{1}H of 8-bromo-6-chloroflavanone 3’-*O*-*β*-D-(4”-*O*-methyl)-glucopyranoside (7a)
Figure S139. COSY contour map – ^{1}H x ^{1}H expansion of 8-bromo-6-chloroflavanone 3’-*O*-*β*-D-(4”-*O*-methyl)-glucopyranoside (7a)
Figure S140. COSY contour map – ^{1}H x ^{1}H expansion of 8-bromo-6-chloroflavanone 3’-*O*-*β*-D-(4”-*O*-methyl)-glucopyranoside (7a)
Figure S141. COSY contour map – ^{1}H x ^{1}H expansion of 8-bromo-6-chloroflavanone 3’-*O*-*β*-D-(4”-*O*-methyl)-glucopyranoside (7a)
Figure S142. HSQC contour map – ^{1}H x ^{13}C of 8-bromo-6-chloroflavanone 3’-*O*-*β*-D-(4”-*O*-methyl)-glucopyranoside (7a)
Figure S143. HSQC contour map – ^{1}H x ^{13}C expansion of 8-bromo-6-chloroflavanone 3’-*O*-*β*-D-(4”-*O*-methyl)-glucopyranoside (7a)
Figure S144. HSQC contour map – ^{1}H x ^{13}C expansion of 8-bromo-6-chloroflavanone 3’-*O*-*β*-D-(4”-*O*-methyl)-glucopyranoside (7a)
Figure S145. HSQC contour map – ^{1}H x ^{13}C expansion of 8-bromo-6-chloroflavanone 3’-*O*-*β*-D-(4”-*O*-methyl)-glucopyranoside (7a)

Figure S146. HMBC contour map – ^{1}H x ^{13}C of 8-bromo-6-chloroflavanone 3’-*O*-*β*-D-(4”-*O*-methyl)-glucopyranoside (7a)
Figure S147. HMBC contour map – ^{1}H x ^{13}C expansion of 8-bromo-6-chloroflavanone 3’-*O*-*β*-D-(4”-*O*-methyl)-glucopyranoside (7a)
Figure S148. HMBC contour map – ^{1}H x ^{13}C expansion of 8-bromo-6-chloroflavanone 3’-*O*-*β*-D-(4”-*O*-methyl)-glucopyranoside (7a)
Figure S149. HMBC contour map – ^{1}H x ^{13}C expansion of 8-bromo-6-chloroflavanone 3’-*O*-*β*-D-(4”-*O*-methyl)-glucopyranoside (7a)
Figure S150. HMBC contour map – ^{1}H x ^{13}C expansion of 8-bromo-6-chloroflavanone 3’-*O*-*β*-D-(4”-*O*-methyl)-glucopyranoside (7a)
Figure S151. MS analysis of 8-bromo-6-chloroflavanone (8)
Figure S152. ^{1}H NMR spectrum of 8-bromo-6-chloroflavanone (8)
Figure S153. ^{1}H NMR spectrum expansion of 8-bromo-6-chloroflavanone (8)
Figure S154. ^{1}H NMR spectrum expansion of 8-bromo-6-chloroflavanone (8)
Figure S155. ^{13}C NMR spectrum of 8-bromo-6-chloroflavanone (8)
Figure S156. ^{13}C NMR spectrum expansion of 8-bromo-6-chloroflavanone (8)
Figure S157. ^{13}C NMR spectrum expansion of 8-bromo-6-chloroflavanone (8)
Figure S158. COSY contour map – ^{1}H x ^{1}H of 8-bromo-6-chloroflavanone (8)
Figure S159. COSY contour map – ^{1}H x ^{1}H expansion of 8-bromo-6-chloroflavanone (8)
Figure S160. COSY contour map – ^{1}H x ^{1}H expansion of 8-bromo-6-chloroflavanone (8)
Figure S161. HSQC contour map – ^{1}H x ^{13}C of 8-bromo-6-chloroflavanone (8)
Figure S162. HSQC contour map – ^{1}H x ^{13}C expansion of 8-bromo-6-chloroflavanone (8)
Figure S163. HSQC contour map – ^{1}H x ^{13}C expansion of 8-bromo-6-chloroflavanone (8)
Figure S164. HMBC contour map – ^{1}H x ^{13}C of 8-bromo-6-chloroflavanone (8)
Figure S165. HMBC contour map – ^{1}H x ^{13}C expansion of 8-bromo-6-chloroflavanone (8)
Figure S166. HMBC contour map – ^{1}H x ^{13}C expansion of 8-bromo-6-chloroflavanone (8)
Figure S167. MS analysis of 8-bromo-6-chloroflavan-4-ol 4’-*O*-*β*-D-(4”-*O*-methyl)-glucopyranoside (8a)
Figure S168. ^{1}H NMR spectrum of 8-bromo-6-chloroflavan-4-ol 4’-*O*-*β*-D-(4”-*O*-methyl)-glucopyranoside (8a)
Figure S169. ^{1}H NMR spectrum expansion of 8-bromo-6-chloroflavan-4-ol 4’-*O*-*β*-D-(4”-*O*-methyl)-glucopyranoside (8a)
Figure S170. ^{1}H NMR spectrum expansion of 8-bromo-6-chloroflavan-4-ol 4’-*O*-*β*-D-(4”-*O*-methyl)-glucopyranoside (8a)
Figure S171. ^{1}H NMR spectrum expansion of 8-bromo-6-chloroflavan-4-ol 4’-*O*-*β*-D-(4”-*O*-methyl)-glucopyranoside (8a)
Figure S172. ^{13}C NMR spectrum of 8-bromo-6-chloroflavan-4-ol 4’-*O*-*β*-D-(4”-*O*-methyl)-glucopyranoside (8a)
Figure S173. ^{13}C NMR spectrum expansion of 8-bromo-6-chloroflavan-4-ol 4’-*O*-*β*-D-(4”-*O*-methyl)-glucopyranoside (8a)
Figure S174. ^{13}C NMR spectrum expansion of 8-bromo-6-chloroflavan-4-ol 4’-*O*-*β*-D-(4”-*O*-methyl)-glucopyranoside (8a)
Figure S175. COSY contour map – ^{1}H x ^{1}H of 8-bromo-6-chloroflavan-4-ol 4’-*O*-*β*-D-(4”-*O*-methyl)-glucopyranoside (8a)
Figure S176. COSY contour map – ^{1}H x ^{1}H expansion of 8-bromo-6-chloroflavan-4-ol 4’-*O*-*β*-D-(4”-*O*-methyl)-glucopyranoside (8a)
Figure S177. COSY contour map – ^{1}H x ^{1}H expansion of 8-bromo-6-chloroflavan-4-ol 4’-*O*-*β*-D-(4”-*O*-methyl)-glucopyranoside (8a)

Figure S178. COSY contour map – ^{1}H x ^{1}H expansion of 8-bromo-6-chloroflavan-4-ol 4'-*O*-*β*-D-(4"-*O*-methyl)-glucopyranoside (8a)
Figure S179. HSQC contour map – ^{1}H x ^{13}C of 8-bromo-6-chloroflavan-4-ol 4'-*O*-*β*-D-(4"-*O*-methyl)-glucopyranoside (8a)
Figure S180. HSQC contour map – ^{1}H x ^{13}C expansion of 8-bromo-6-chloroflavan-4-ol 4'-*O*-*β*-D-(4"-*O*-methyl)-glucopyranoside (8a)
Figure S181. HSQC contour map – ^{1}H x ^{13}C expansion of 8-bromo-6-chloroflavan-4-ol 4'-*O*-*β*-D-(4"-*O*-methyl)-glucopyranoside (8a)
Figure S182. HMBC contour map – ^{1}H x ^{13}C of 8-bromo-6-chloroflavan-4-ol 4'-*O*-*β*-D-(4"-*O*-methyl)-glucopyranoside (8a)
Figure S183. HMBC contour map – ^{1}H x ^{13}C expansion of 8-bromo-6-chloroflavan-4-ol 4'-*O*-*β*-D-(4"-*O*-methyl)-glucopyranoside (8a)
Figure S184. HMBC contour map – ^{1}H x ^{13}C expansion of 8-bromo-6-chloroflavan-4-ol 4'-*O*-*β*-D-(4"-*O*-methyl)-glucopyranoside (8a)
Figure S185. MS analysis of 8-bromo-6-chloroflavone (9)
Figure S186. ^{1}H NMR spectrum of 8-bromo-6-chloroflavone (9)
Figure S187. ^{1}H NMR spectrum expansion of 8-bromo-6-chloroflavone (9)
Figure S188. ^{13}C NMR spectrum of 8-bromo-6-chloroflavone (9)
Figure S 189. ^{13}C NMR spectrum expansion of 8-bromo-6-chloroflavone (9)
Figure S190. COSY contour map – ^{1}H x ^{1}H of 8-bromo-6-chloroflavone (9)
Figure S191. COSY contour map – ^{1}H x ^{1}H expansion of 8-bromo-6-chloroflavone (9)
Figure S192. HSQC contour map – ^{1}H x ^{13}C of 8-bromo-6-chloroflavone (9)
Figure S193. HSQC contour map – ^{1}H x ^{13}C expansion of 8-bromo-6-chloroflavone (9)
Figure S194. HMBC contour map – ^{1}H x ^{13}C of 8-bromo-6-chloroflavone (9)
Figure S195. HMBC contour map – ^{1}H x ^{13}C expansion of 8-bromo-6-chloroflavone (9)
Figure S196. HMBC contour map – ^{1}H x ^{13}C expansion of 8-bromo-6-chloroflavone (9)
Figure S197. MS analysis of 8-bromo-6-chloroflavone 4'-*O*-*β*-D-(4"-*O*-methyl)-glucopyranoside (9a)
Figure S198. ^{1}H NMR spectrum of 8-bromo-6-chloroflavone 4'-*O*-*β*-D-(4"-*O*-methyl)-glucopyranoside (9a)
Figure S199. ^{1}H NMR spectrum expansion of 8-bromo-6-chloroflavone 4'-*O*-*β*-D-(4"-*O*-methyl)-glucopyranoside (9a)
Figure S200. ^{1}H NMR spectrum expansion of 8-bromo-6-chloroflavone 4'-*O*-*β*-D-(4"-*O*-methyl)-glucopyranoside (9a)
Figure S201. ^{1}H NMR spectrum expansion of 8-bromo-6-chloroflavone 4'-*O*-*β*-D-(4"-*O*-methyl)-glucopyranoside (9a)
Figure S202. ^{13}C NMR spectrum of 8-bromo-6-chloroflavone 4'-*O*-*β*-D-(4"-*O*-methyl)-glucopyranoside (9a)
Figure S203. ^{13}C NMR spectrum expansion of 8-bromo-6-chloroflavone 4'-*O*-*β*-D-(4"-*O*-methyl)-glucopyranoside (9a)
Figure S204. ^{13}C NMR spectrum expansion of 8-bromo-6-chloroflavone 4'-*O*-*β*-D-(4"-*O*-methyl)-glucopyranoside (9a)
Figure S205. COSY contour map – ^{1}H x ^{1}H of 8-bromo-6-chloroflavone 4'-*O*-*β*-D-(4"-*O*-methyl)-glucopyranoside (9a)
Figure S206. COSY contour map – ^{1}H x ^{1}H expansion of 8-bromo-6-chloroflavone 4'-*O*-*β*-D-(4"-*O*-methyl)-glucopyranoside (9a)
Figure S207. COSY contour map – ^{1}H x ^{1}H expansion of 8-bromo-6-chloroflavone 4'-*O*-*β*-D-(4"-*O*-methyl)-glucopyranoside (9a)

Figure S208. HSQC contour map – ^{1}H x ^{13}C of 8-bromo-6-chloroflavone 4’-*O*-*β*-D-(4”-*O*-methyl)-glucopyranoside (9a)
Figure S209. HSQC contour map – ^{1}H x ^{13}C expansion of 8-bromo-6-chloroflavone 4’-*O*-*β*-D-(4”-*O*-methyl)-glucopyranoside (9a)
Figure S210. HSQC contour map – ^{1}H x ^{13}C expansion of 8-bromo-6-chloroflavone 4’-*O*-*β*-D-(4”-*O*-methyl)-glucopyranoside (9a)
Figure S211. HMBC contour map – ^{1}H x ^{13}C of 8-bromo-6-chloroflavone 4’-*O*-*β*-D-(4”-*O*-methyl)-glucopyranoside (9a)
Figure S212. HMBC contour map – ^{1}H x ^{13}C expansion of 8-bromo-6-chloroflavone 4’-*O*-*β*-D-(4”-*O*-methyl)-glucopyranoside (9a)
Figure S213. HMBC contour map – ^{1}H x ^{13}C expansion of 8-bromo-6-chloroflavone 4’-*O*-*β*-D-(4”-*O*-methyl)-glucopyranoside (9a)

Molecular Formula: $C_{16}H_{13}NO_4$
Formula Weight: 283.279
Ionization mode: positive
Precursor $[M + H]^+$: 284.085
Collision energy (CE): -25.0

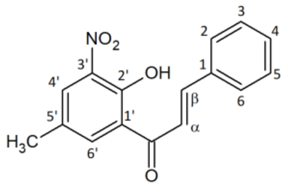

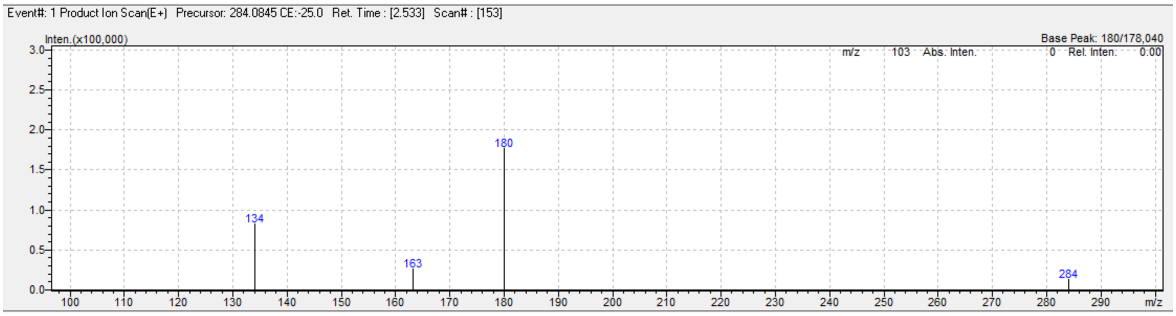

Figure S1. MS analysis of 2’-hydroxy-5’-methyl-3’-nitrochalcone **(4)**

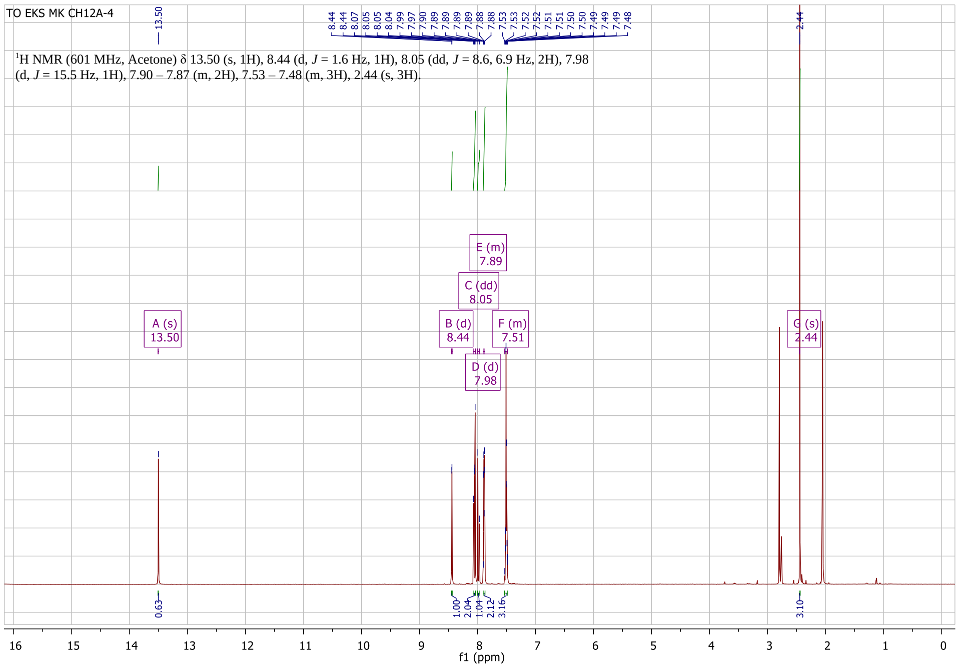

Figure S2. ^{1}H NMR spectrum of 2’-hydroxy-5’-methyl-3’-nitrochalcone **(4)**

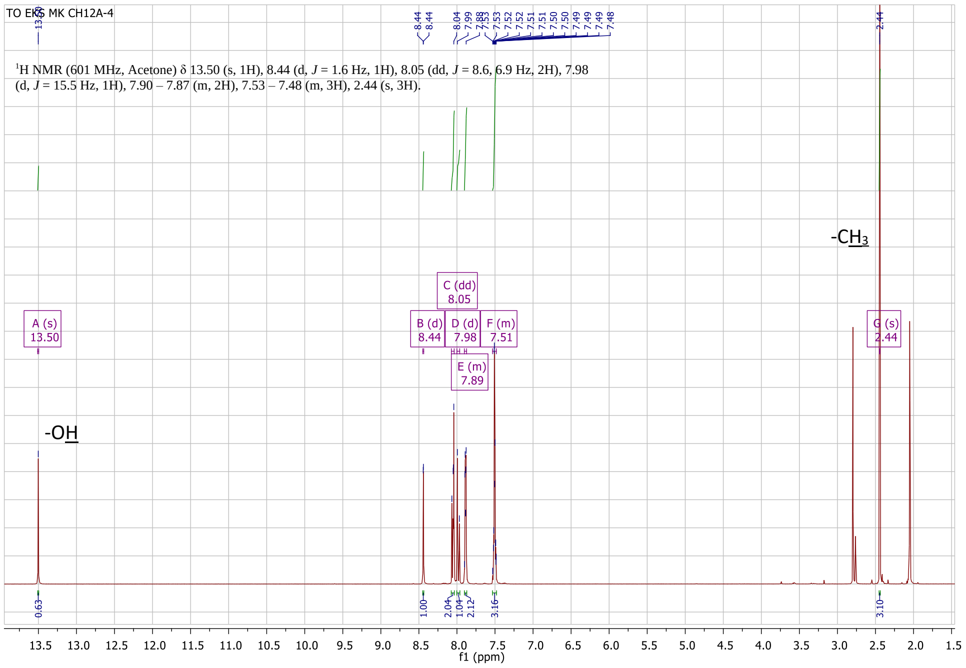

Figure S3. ^{1}H NMR spectrum expansion of 2’-hydroxy-5’-methyl-3’-nitrochalcone **(4)**

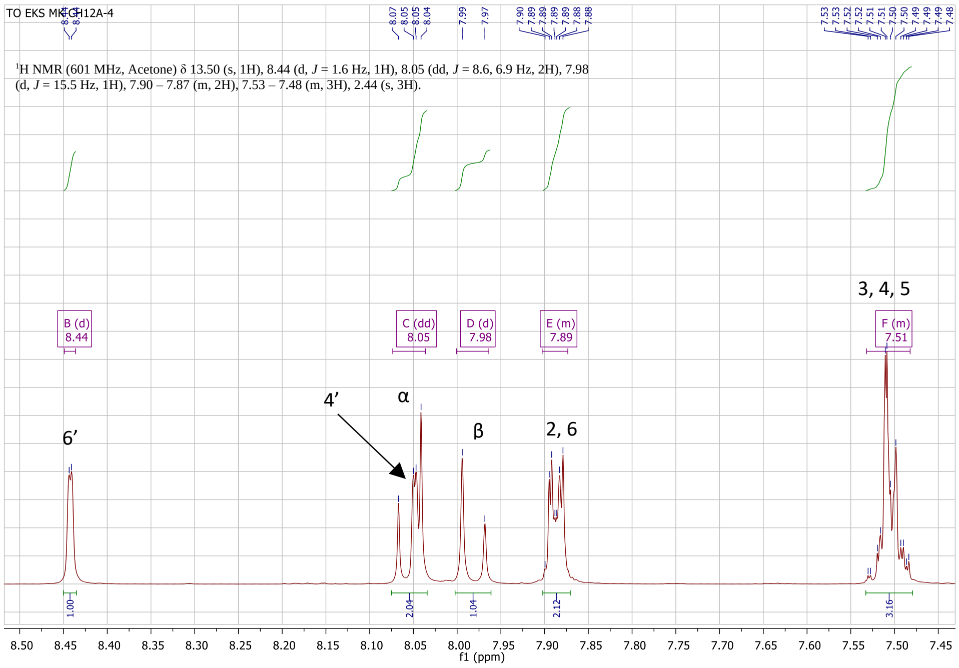

Figure S4. ^{1}H NMR spectrum expansion of 2’-hydroxy-5’-methyl-3’-nitrochalcone **(4)**

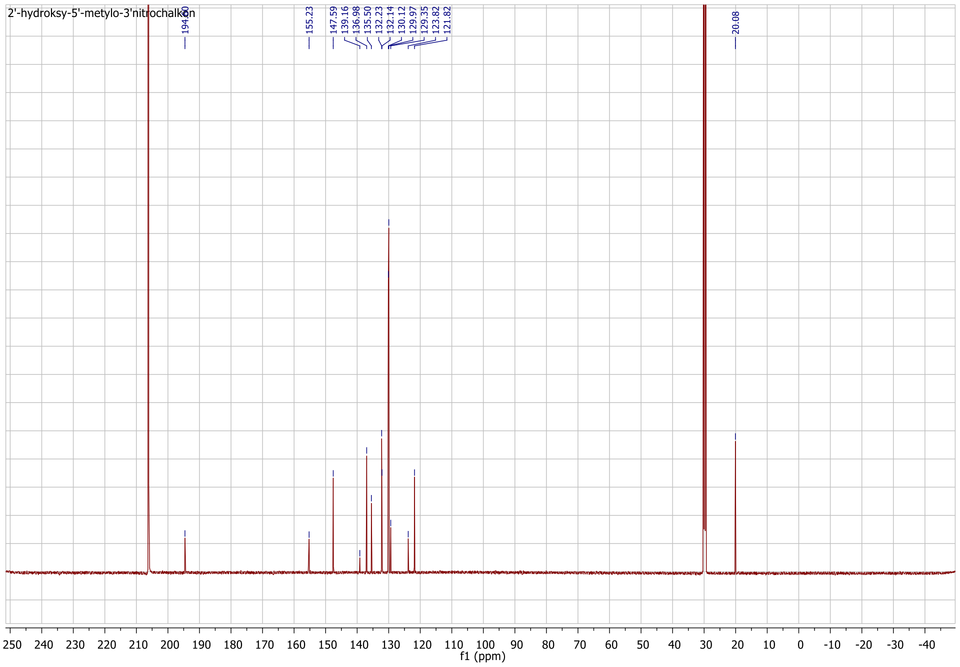

Figure S5. ^{13}C NMR spectrum of 2’-hydroxy-5’-methyl-3’-nitrochalcone **(4)**

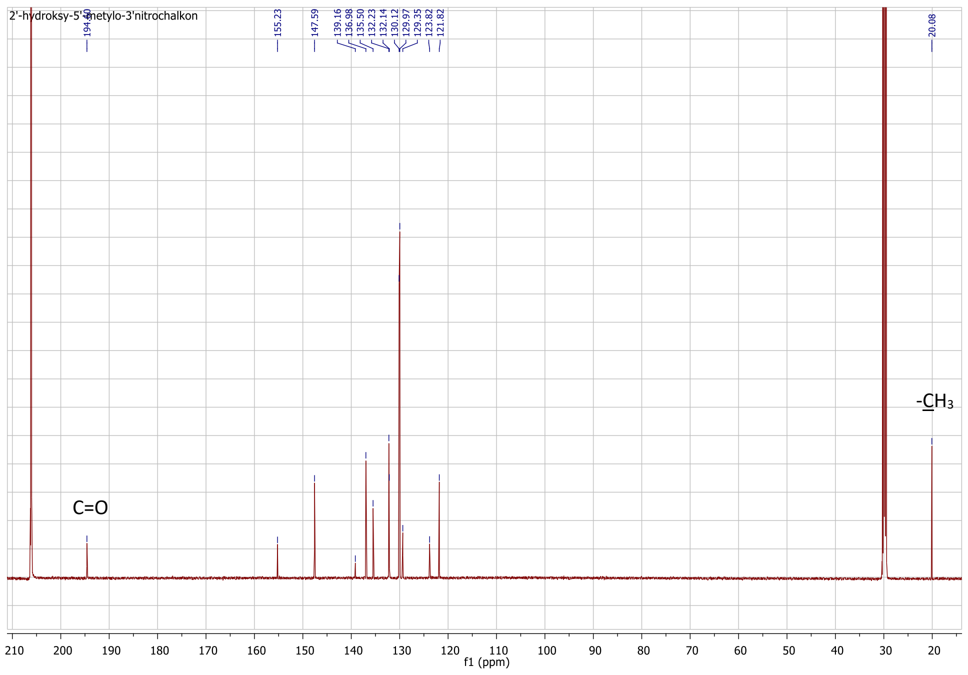

Figure S6. ^{13}C NMR spectrum expansion of 2’-hydroxy-5’-methyl-3’-nitrochalcone **(4)**

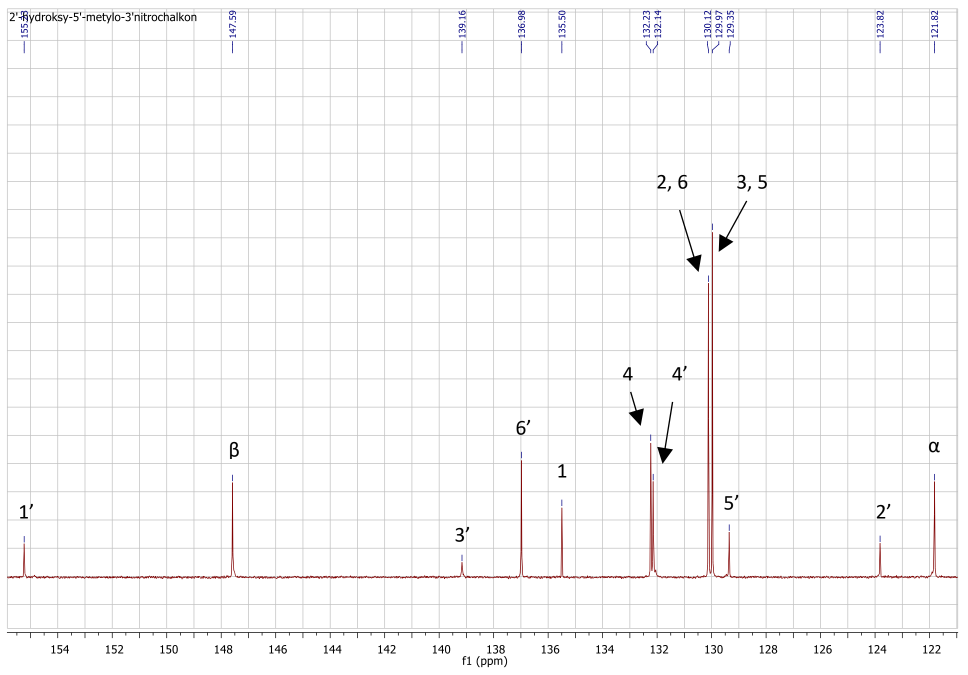

Figure S7. ^{13}C NMR spectrum expansion of 2’-hydroxy-5’-methyl-3’-nitrochalcone **(4)**

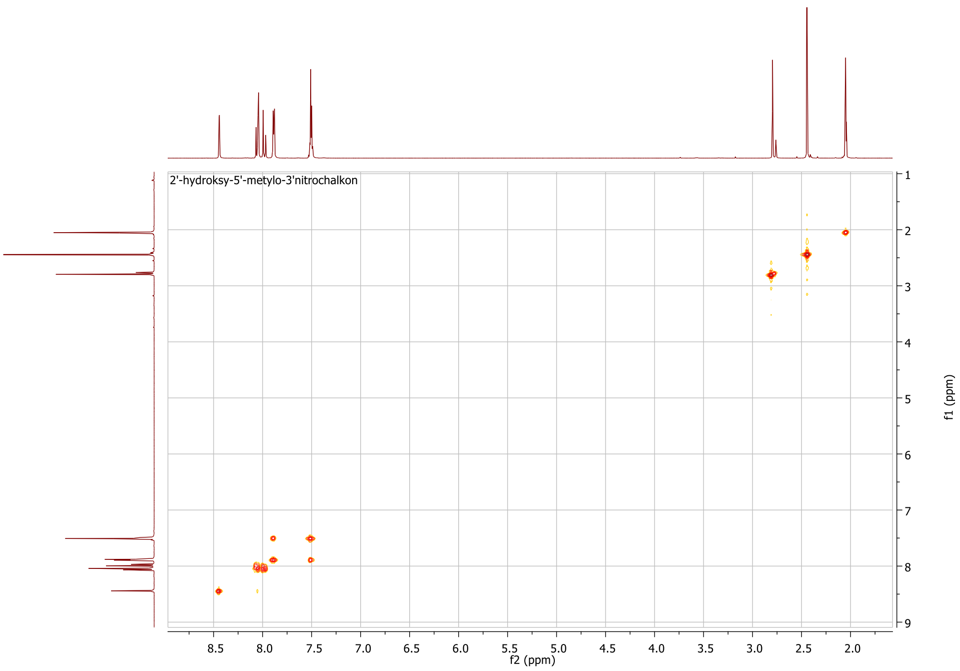

Figure S8. COSY contour map – ^1H x ^1H of 2’-hydroxy-5’-methyl-3’-nitrochalcone **(4)**

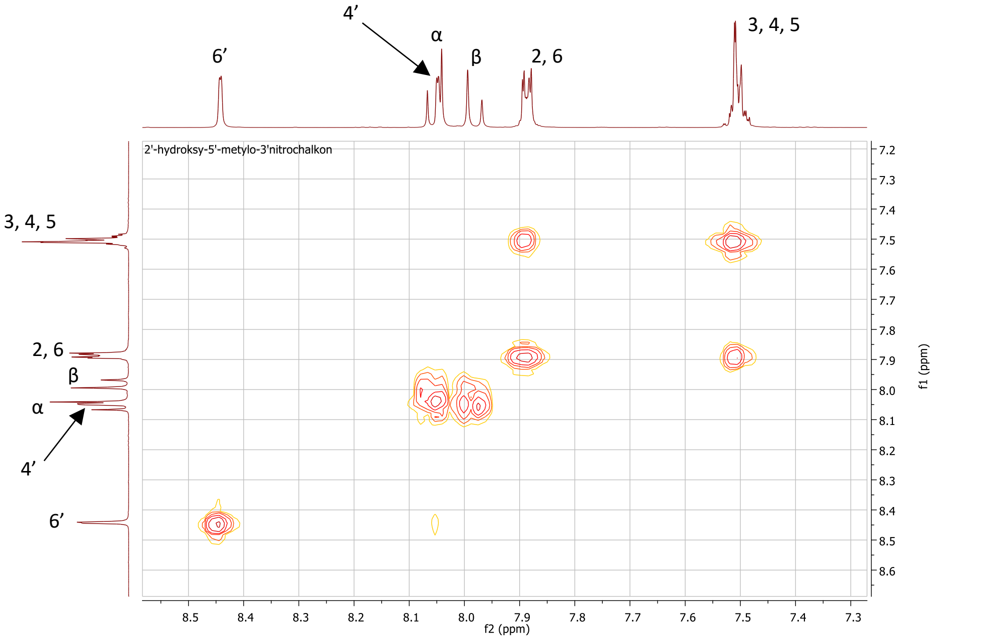

Figure S9. COSY contour map – ^1H x ^1H expansion of 2’-hydroxy-5’-methyl-3’-nitrochalcone **(4)**

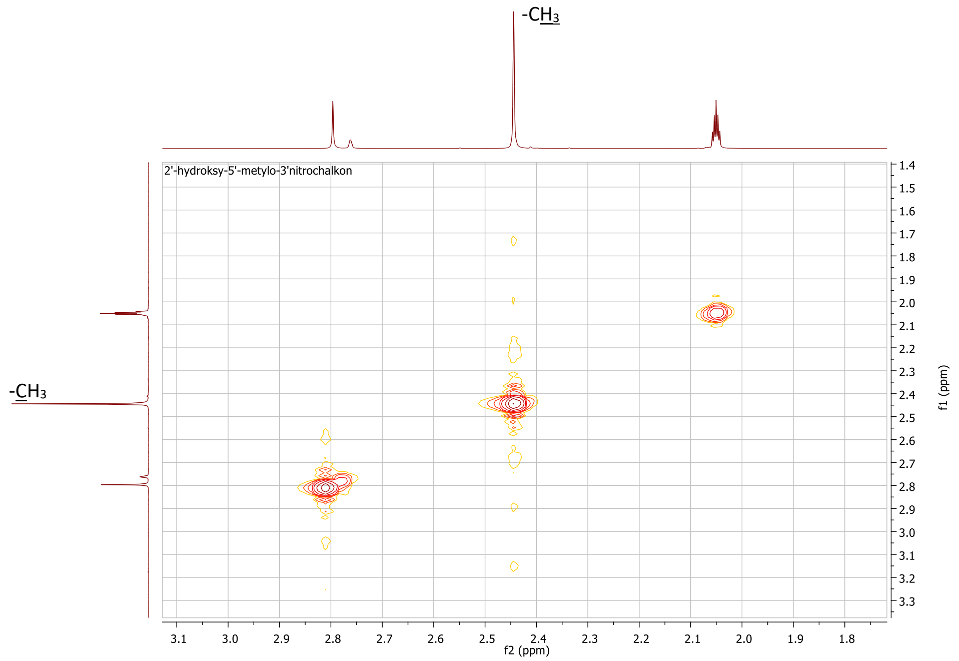

Figure S10. COSY contour map – ^{1}H x ^{1}H of 2’-hydroxy-5’-methyl-3’-nitrochalcone **(4)**

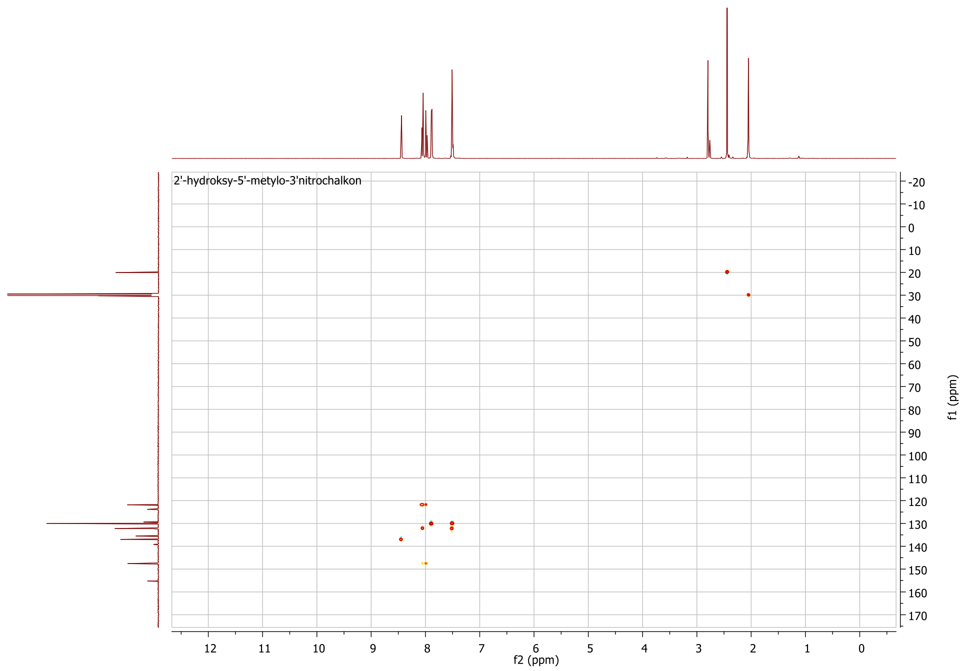

Figure S11. HSQC contour map – ^{1}H x ^{13}C of 2’-hydroxy-5’-methyl-3’-nitrochalcone **(4)**

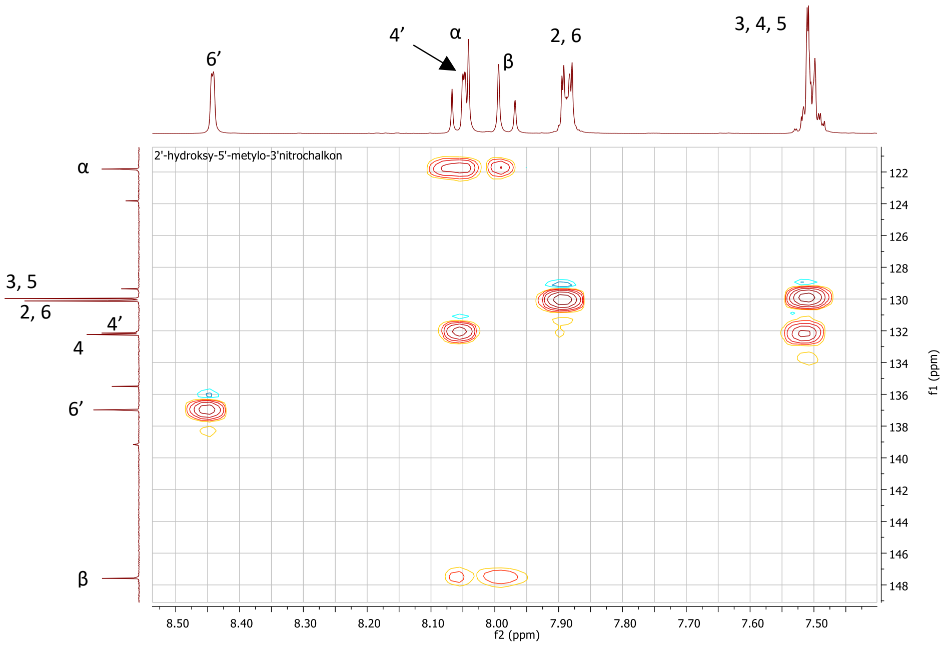

Figure S12. HSQC contour map – ^{1}H x ^{13}C expansion of 2’-hydroxy-5’-methyl-3’-nitrochalcone **(4)**

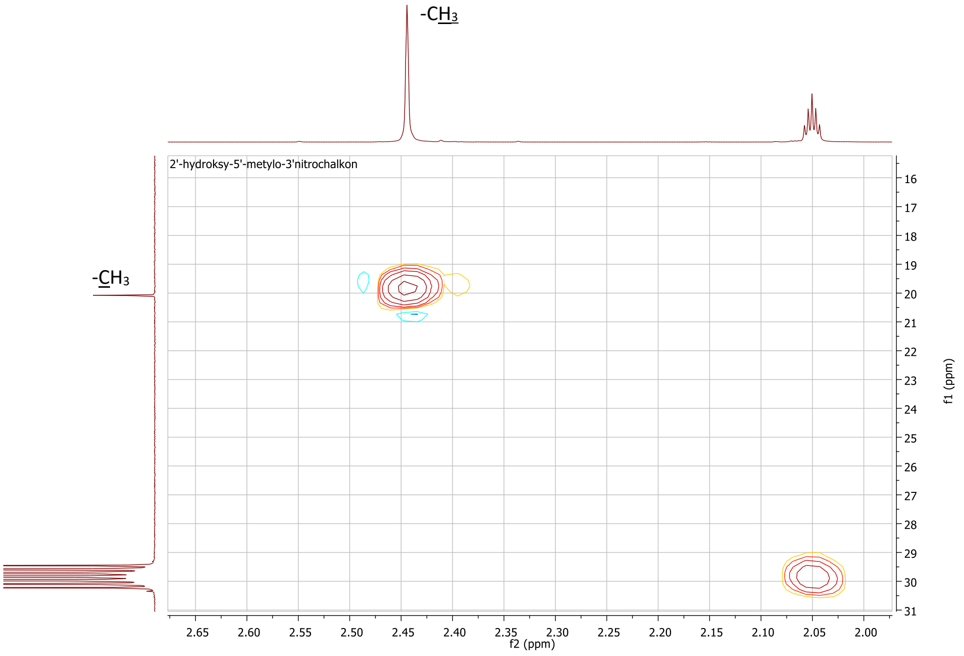

Figure S13. HSQC contour map – ^{1}H x ^{13}C expansion of 2’-hydroxy-5’-methyl-3’-nitrochalcone **(4)**

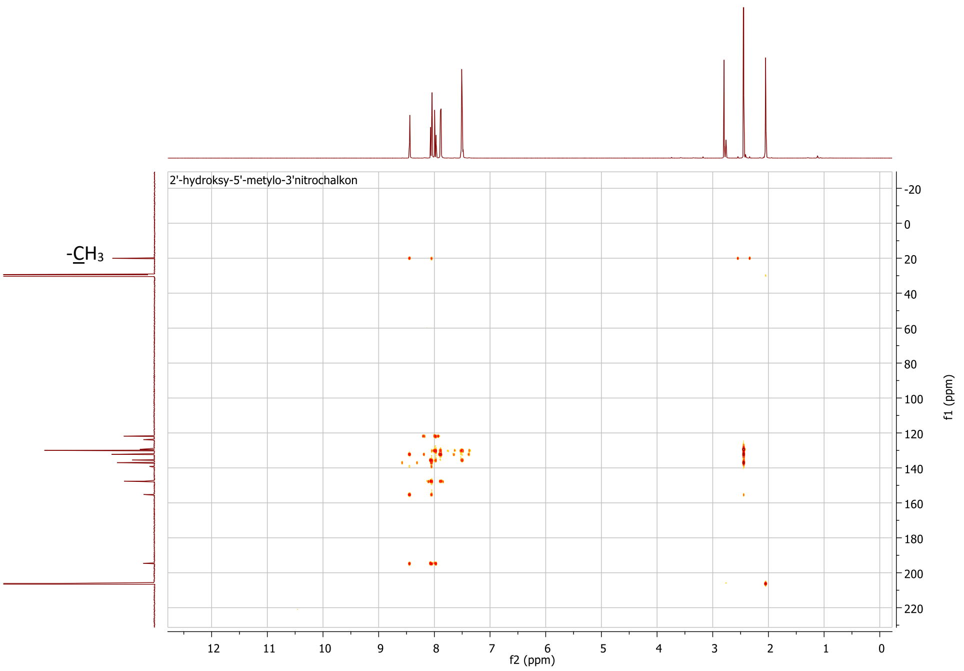

Figure S14. HMBC contour map – ^{1}H x ^{13}C of 2’-hydroxy-5’-methyl-3’-nitrochalcone **(4)**

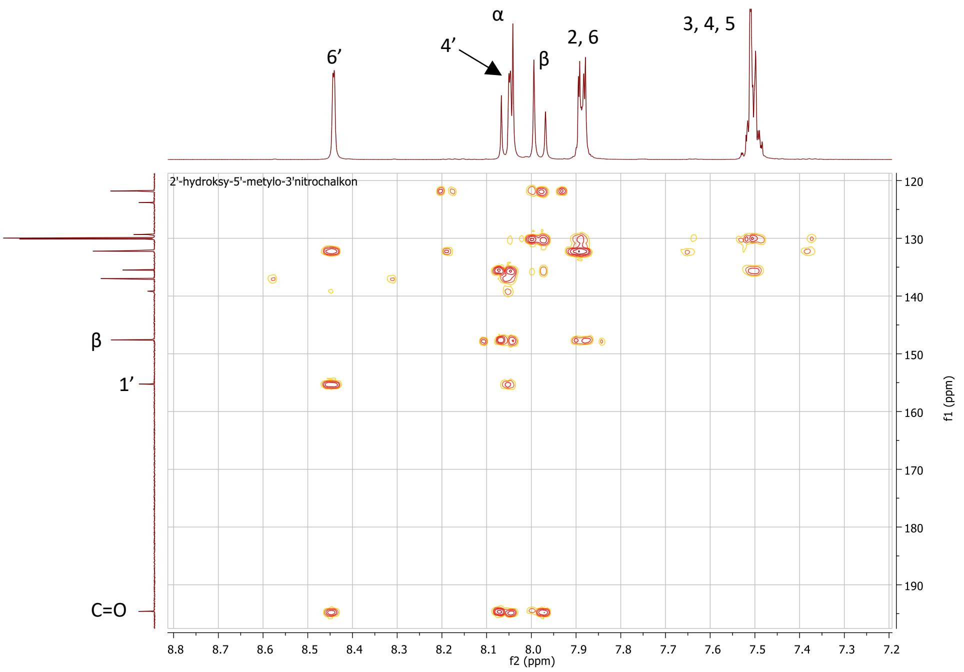

Figure S15. HMBC contour map – ^{1}H x ^{13}C expansion of 2’-hydroxy-5’-methyl-3’-nitrochalcone **(4)**

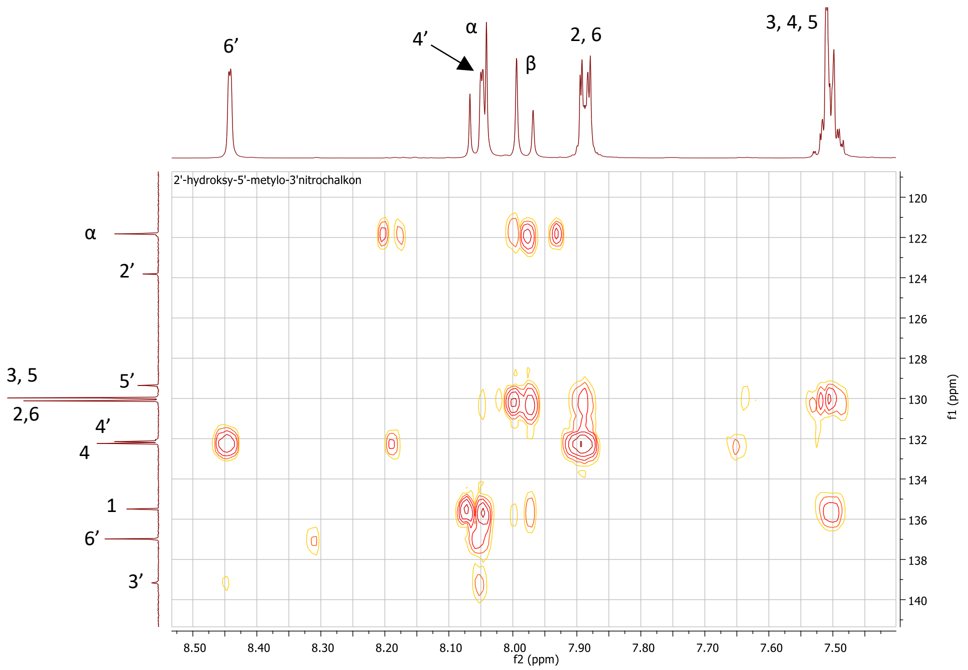

Figure S16. HMBC contour map – ^{1}H x ^{13}C expansion of 2’-hydroxy-5’-methyl-3’-nitrochalcone **(4)**

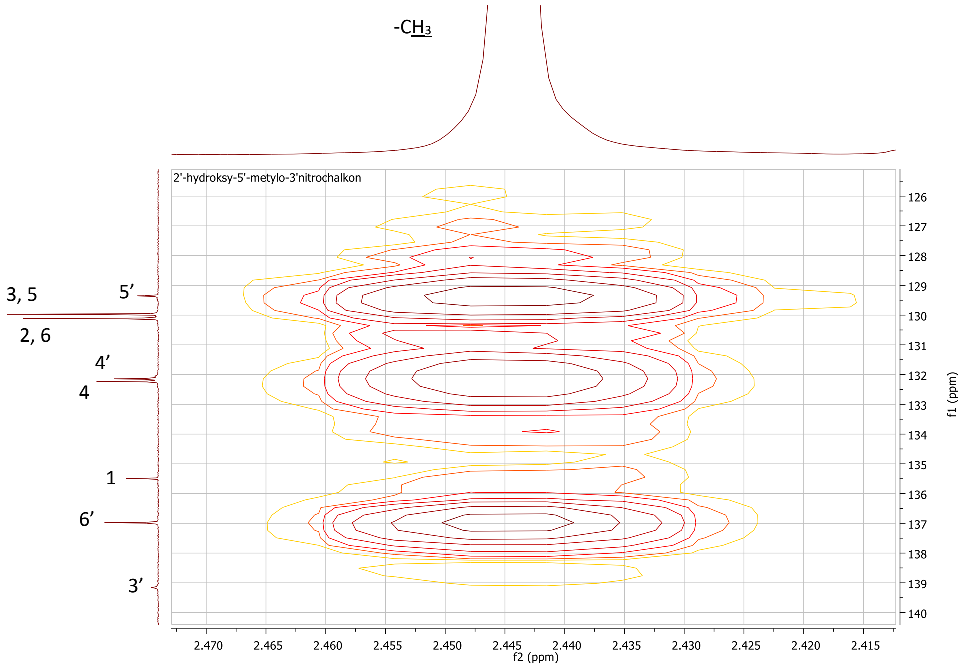

Figure S17. HMBC contour map – ^{1}H x ^{13}C expansion of 2’-hydroxy-5’-methyl-3’-nitrochalcone **(4)**

Molecular Formula: $C_{16}H_{13}NO_4$
Formula Weight: 283.279
Ionization mode: positive
Precursor $[M + H]^+$: 284.085
Collision energy (CE): -20.0

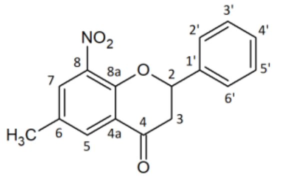

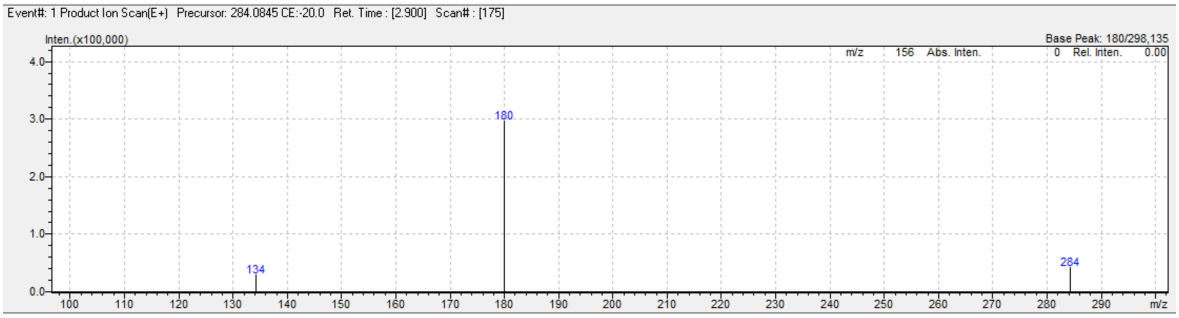

Figure S18. MS analysis of 6-methyl-8-nitroflavanone **(5)**

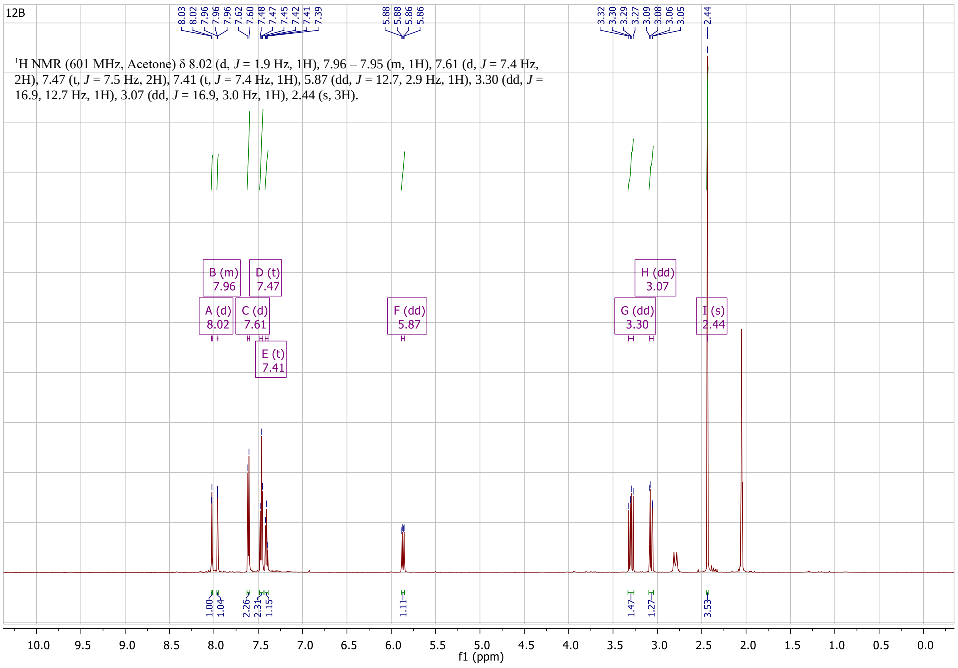

Figure S19. ^{1}H NMR spectrum of 6-methyl-8-nitroflavanone **(5)**

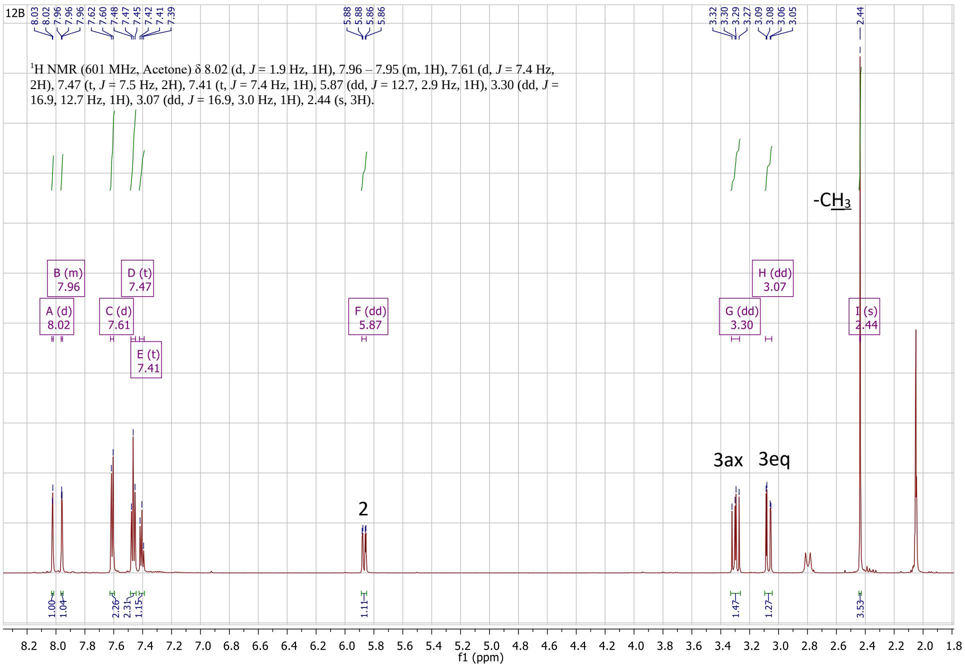

Figure S20. ^{1}H NMR spectrum expansion of 6-methyl-8-nitroflavanone **(5)**

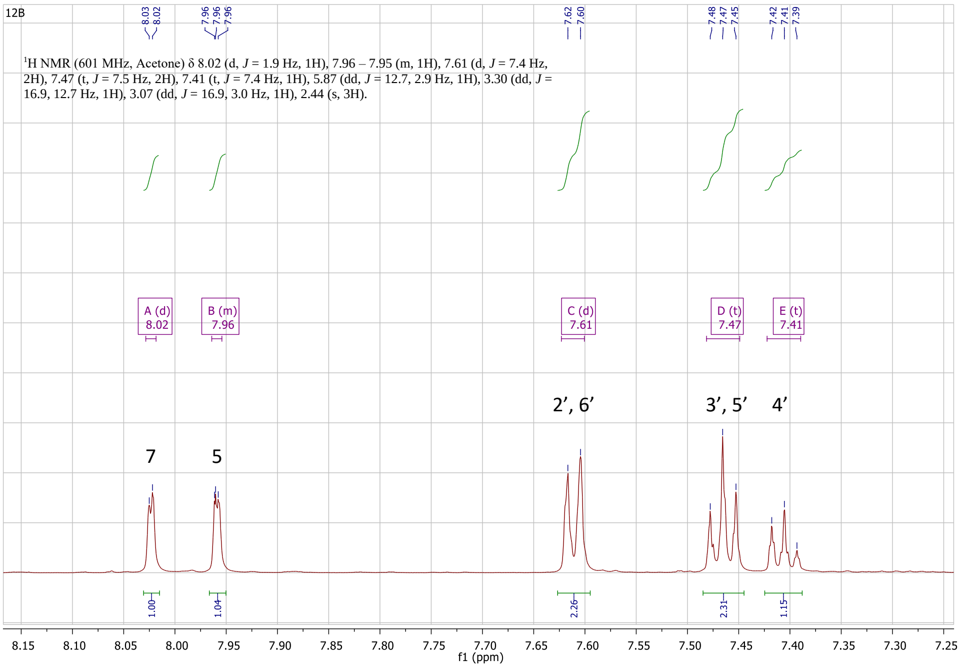

Figure S21. ^{1}H NMR spectrum expansion of 6-methyl-8-nitroflavanone **(5)**

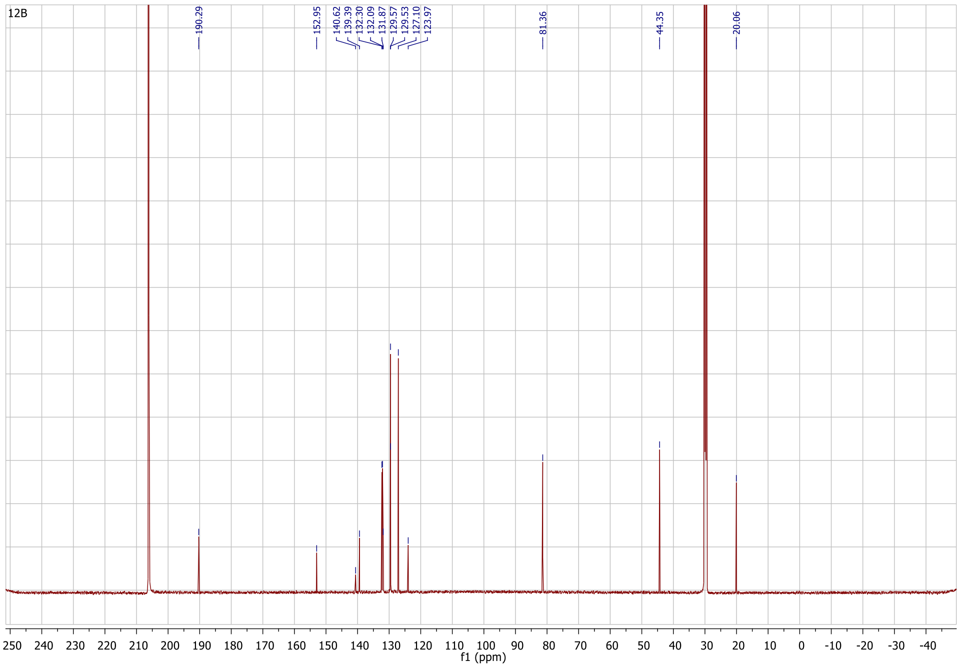

Figure S22. ^{13}C NMR spectrum of 6-methyl-8-nitroflavanone **(5)**

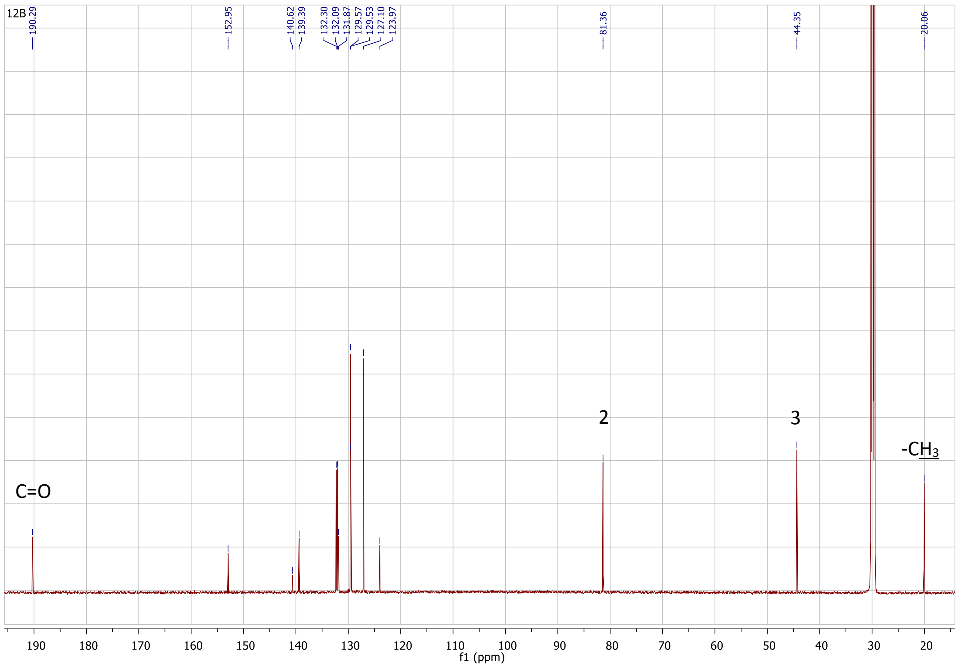

Figure S23. ^{13}C NMR spectrum expansion of 6-methyl-8-nitroflavanone **(5)**

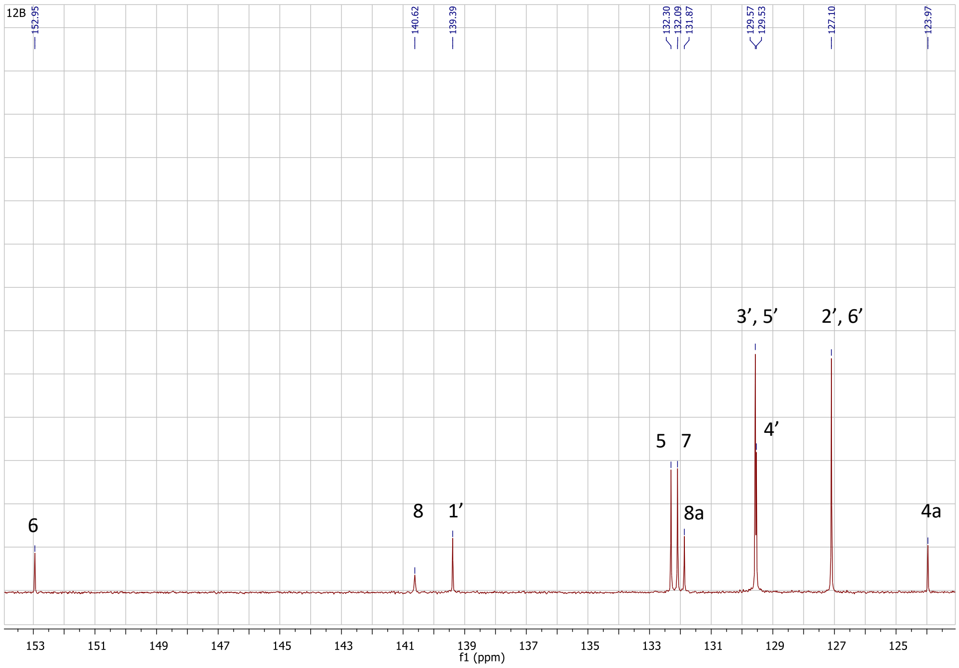

Figure S24. ^{13}C NMR spectrum expansion of 6-methyl-8-nitroflavanone **(5)**

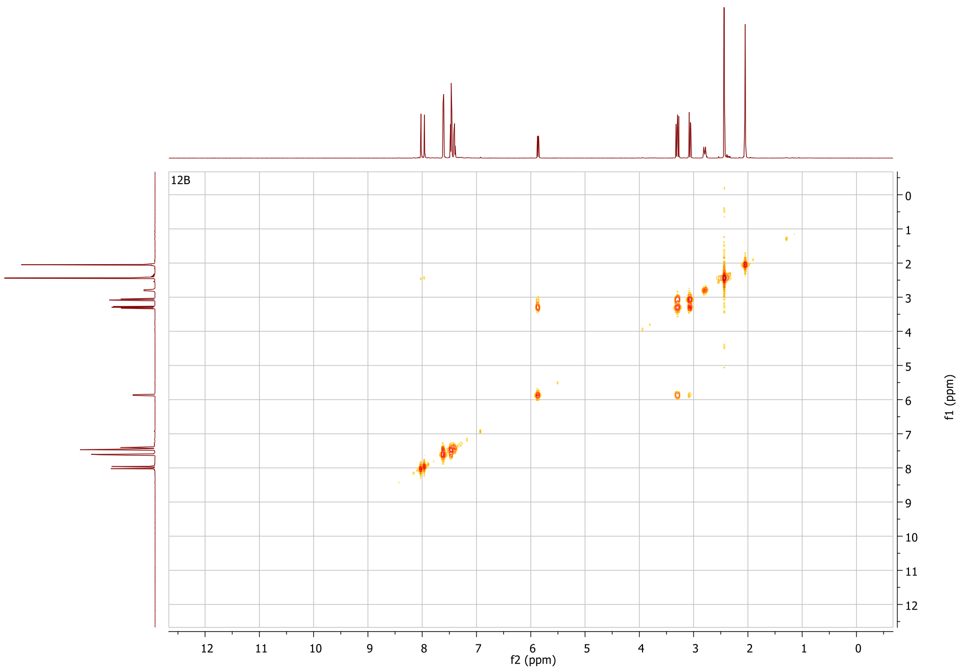

Figure S25. COSY contour map – ^{1}H x ^{1}H of 6-methyl-8-nitroflavanone **(5)**

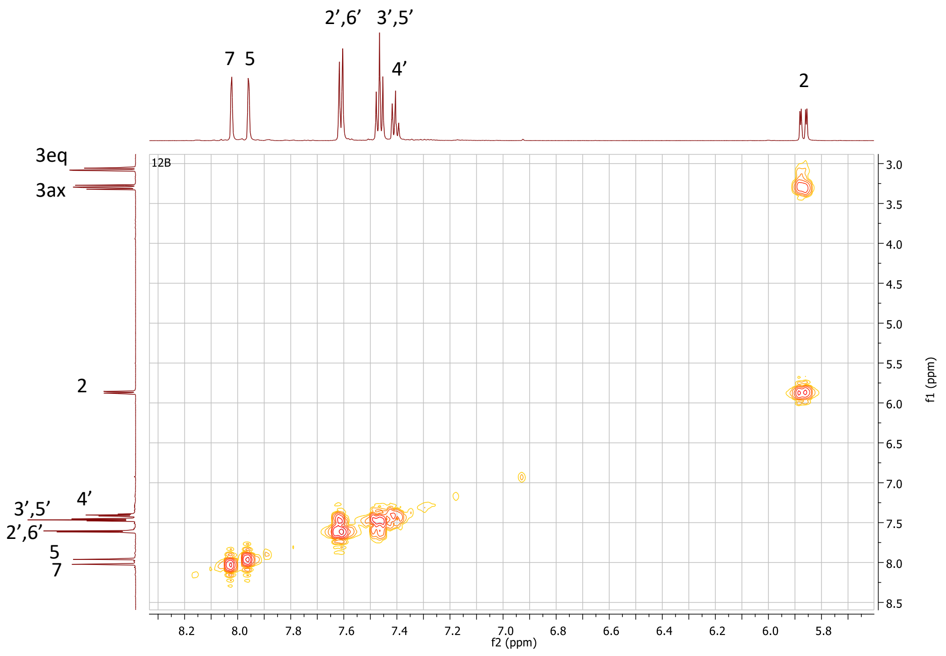

Figure S26. COSY contour map – ^{1}H x ^{1}H expansion of 6-methyl-8-nitroflavanone **(5)**

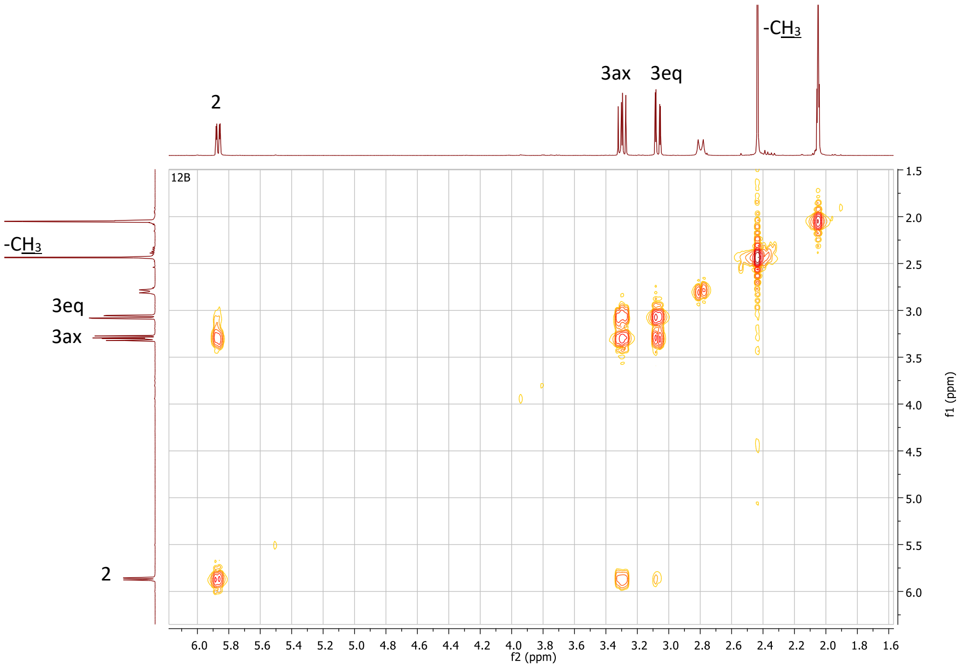

Figure S27. COSY contour map – ^{1}H x ^{1}H expansion of 6-methyl-8-nitroflavanone **(5)**

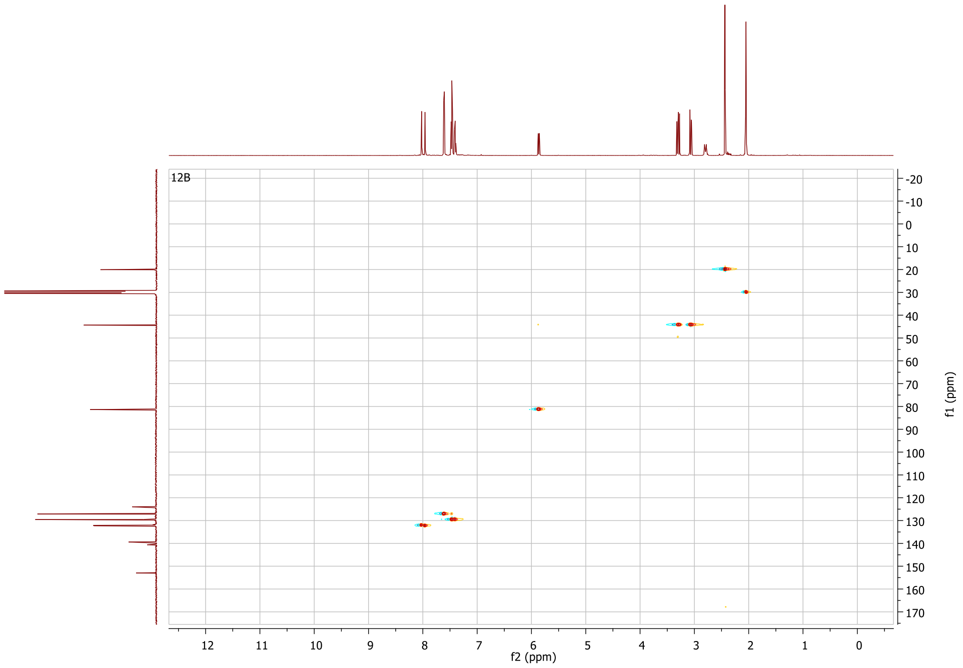

Figure S28. HSQC contour map – ^{1}H x ^{13}C of 6-methyl-8-nitroflavanone **(5)**

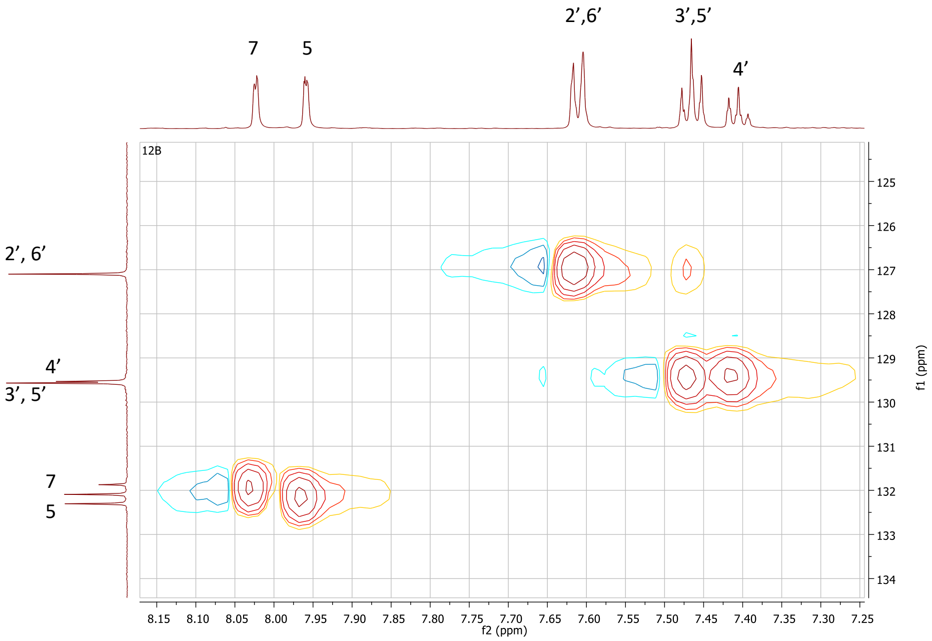

Figure S29. HSQC contour map – ^{1}H x ^{13}C expansion of 6-methyl-8-nitroflavanone **(5)**

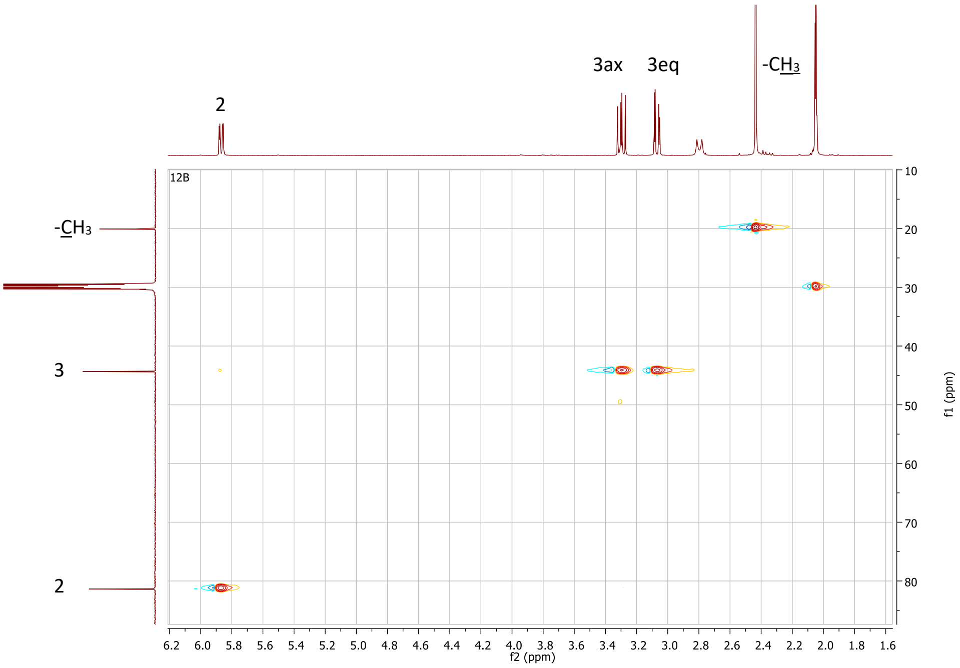

Figure S30. HSQC contour map – ^{1}H x ^{13}C expansion of 6-methyl-8-nitroflavanone **(5)**

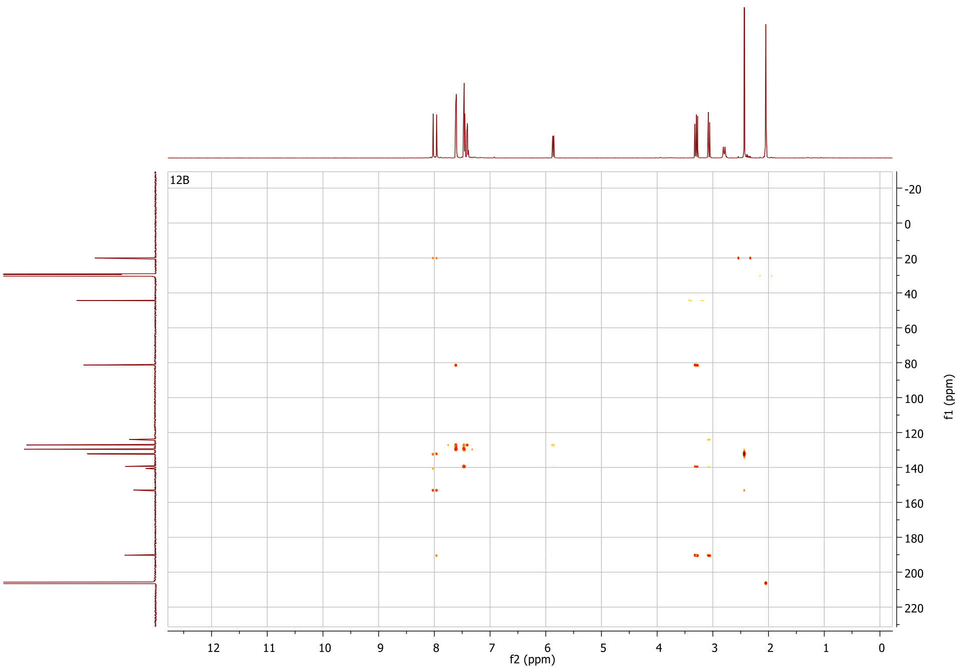

Figure S31. HMBC contour map – ^{1}H x ^{13}C of 6-methyl-8-nitroflavanone **(5)**

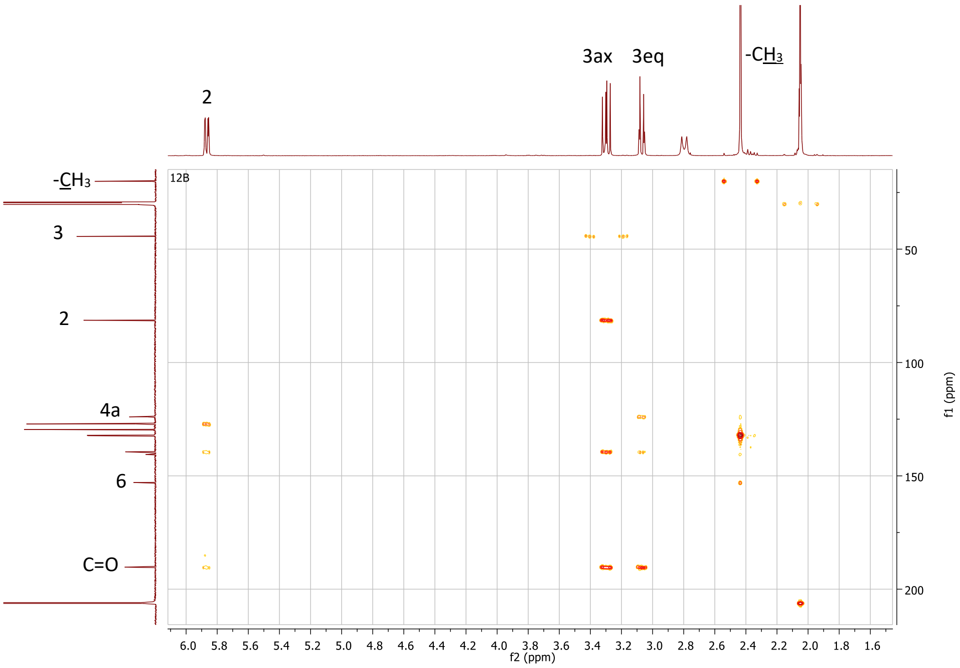

Figure S32. HMBC contour map – ^{1}H x ^{13}C expansion of 6-methyl-8-nitroflavanone **(5)**

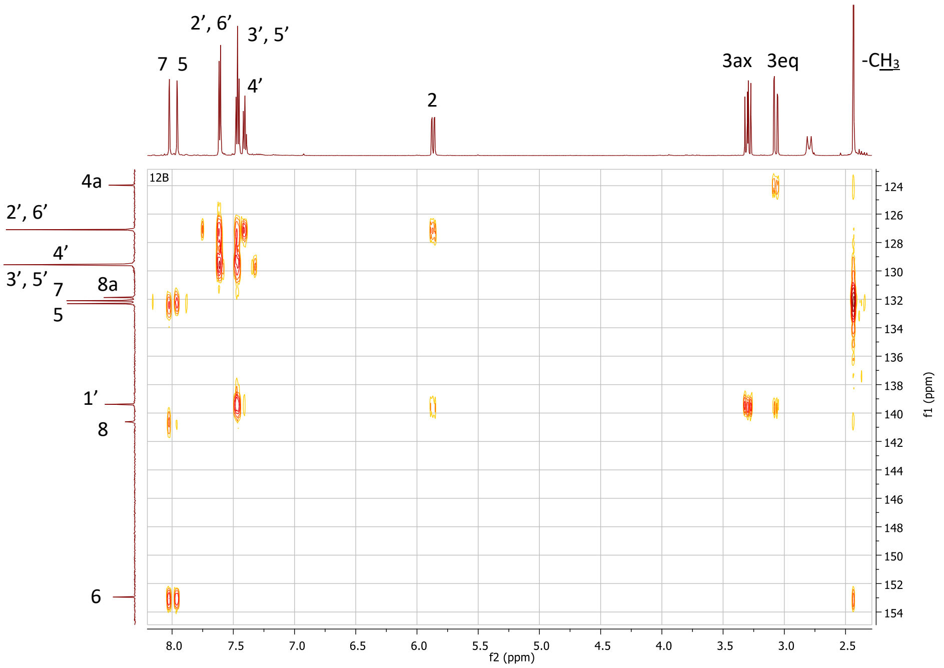

Figure S33. HMBC contour map – ^{1}H x ^{13}C expansion of 6-methyl-8-nitroflavanone **(5)**

Molecular Formula: $C_{23}H_{27}NO_9$
Formula Weight: 461.462
Ionization mode: positive
Precursor $[M + ACN]^+$: 501.166
Collision energy (CE): -25.0

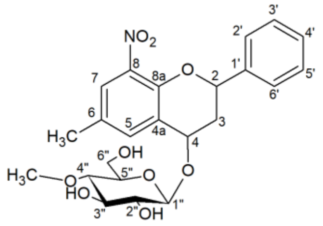

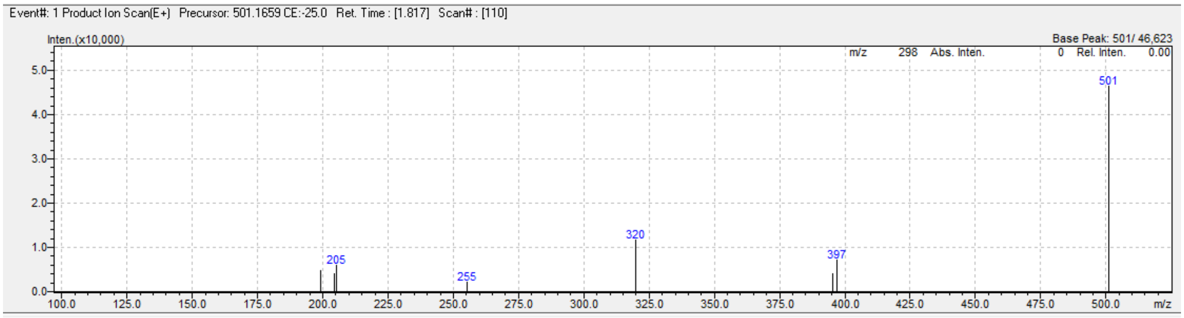

Figure S34. MS analysis of 6-methyl-8-nitro-2-phenylchromane 4-*O*-*β*-D-(4”-*O*-methyl)-glucopyranoside **(5a)**

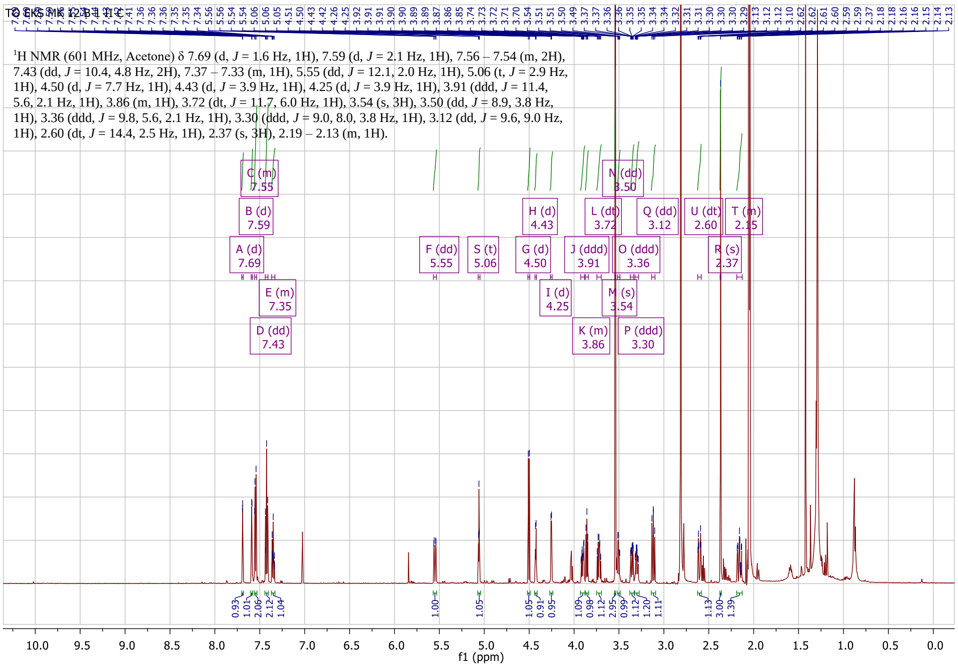

Figure S35. ^{1}H NMR spectrum of 6-methyl-8-nitro-2-phenylchromane 4-*O*-*β*-D-(4”-*O*-methyl)-glucopyranoside **(5a)**

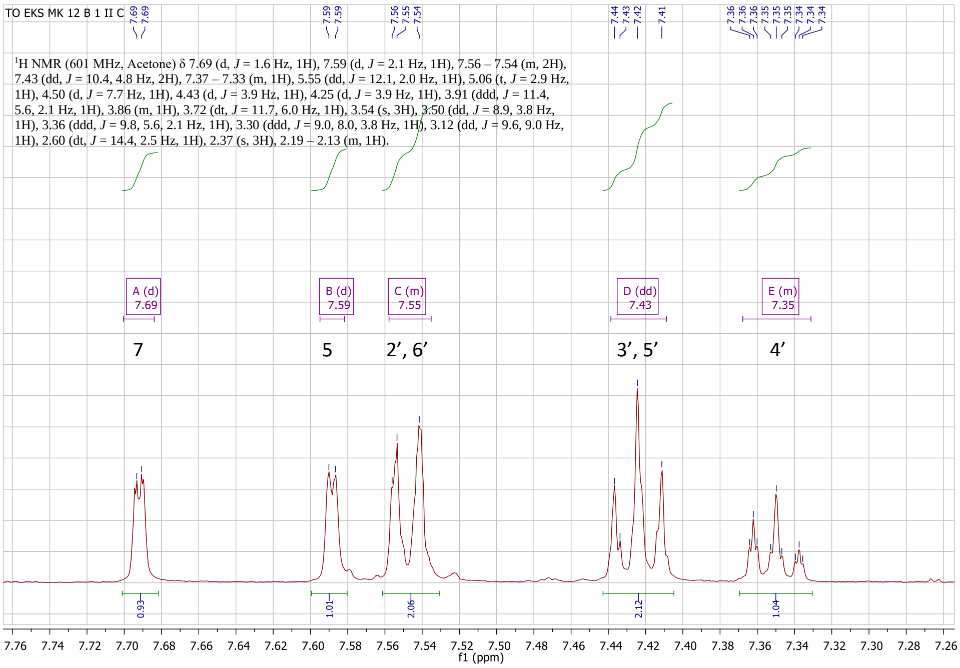

Figure S36. ^{1}H NMR spectrum expansion of 6-methyl-8-nitro-2-phenylchromane 4-*O*-*β*-D-(4”-*O*-methyl)-glucopyranoside **(5a)**

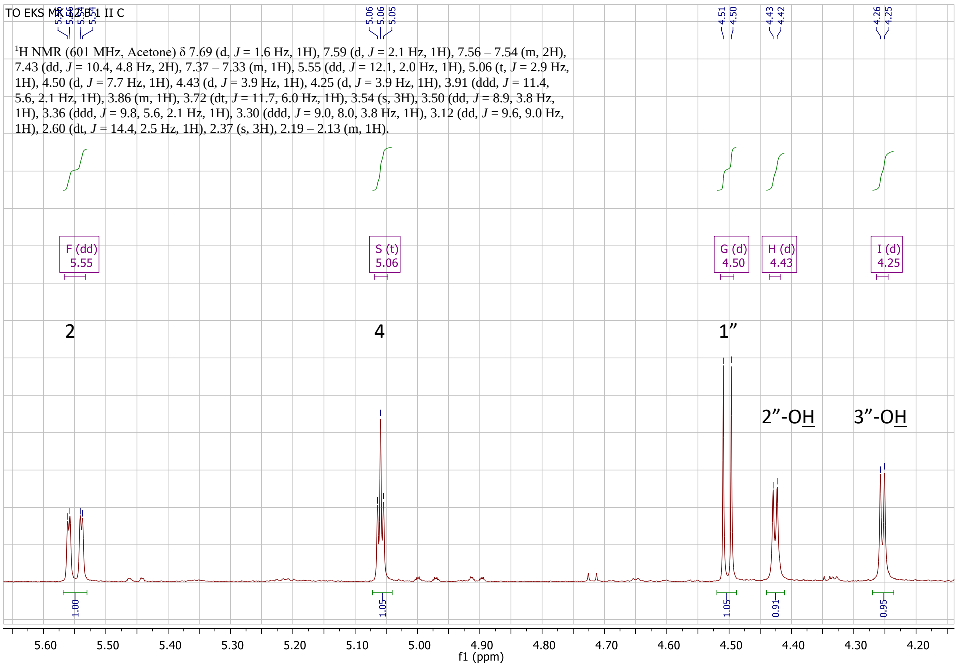

Figure S37. ^{1}H NMR spectrum expansion of 6-methyl-8-nitro-2-phenylchromane 4-*O*-*β*-D-(4”-*O*-methyl)-glucopyranoside **(5a)**

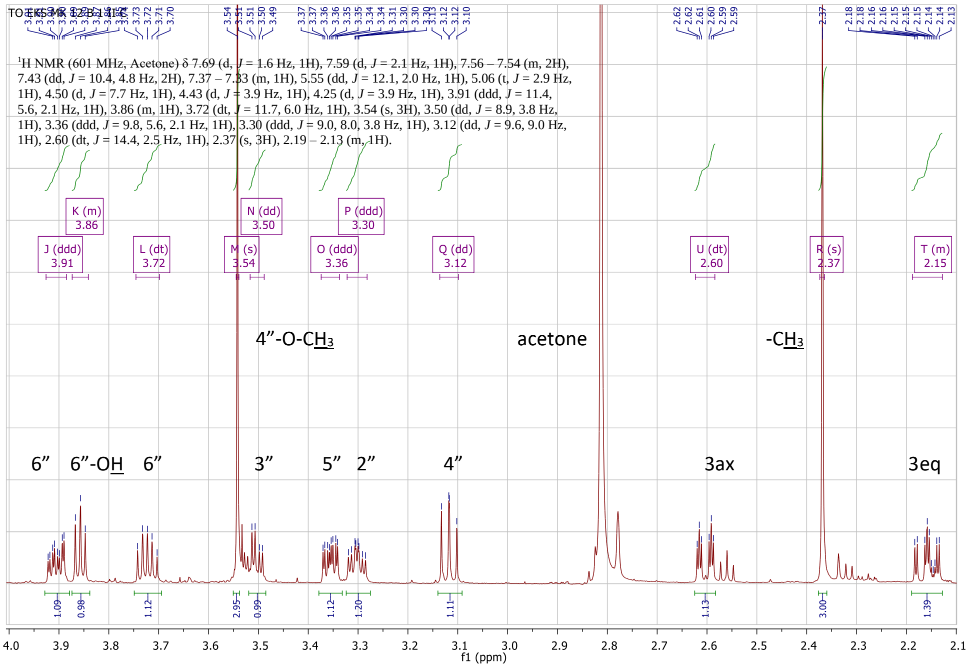

Figure S38. ^{1}H NMR spectrum expansion of 6-methyl-8-nitro-2-phenylchromane 4-*O*-*β*-D-(4”-*O*-methyl)-glucopyranoside **(5a)**

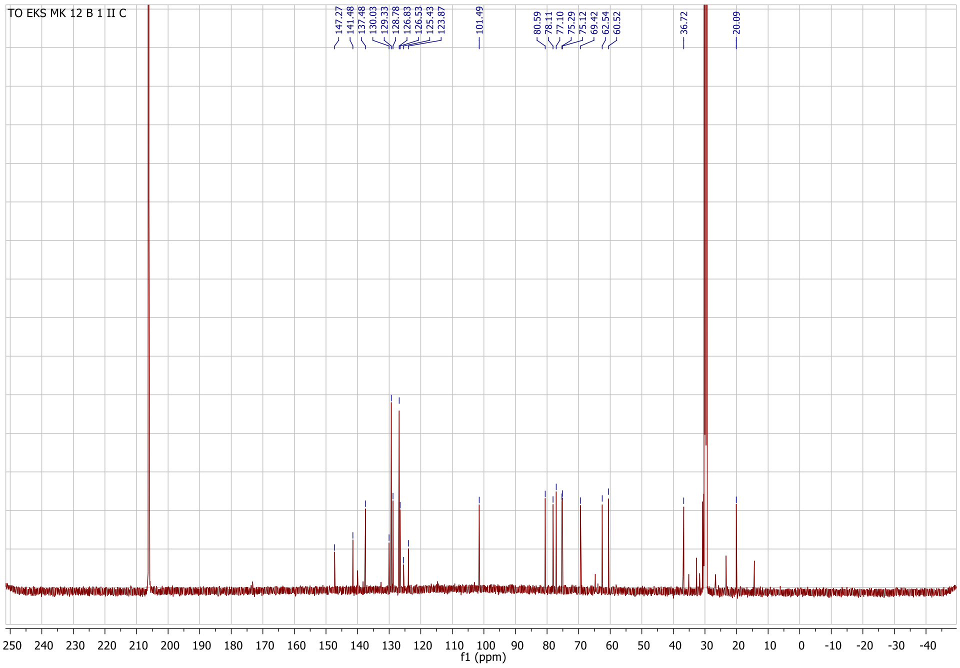

Figure S39. ^{13}C NMR spectrum of 6-methyl-8-nitro-2-phenylchromane 4-*O*-*β*-D-(4”-*O*-methyl)-glucopyranoside **(5a)**

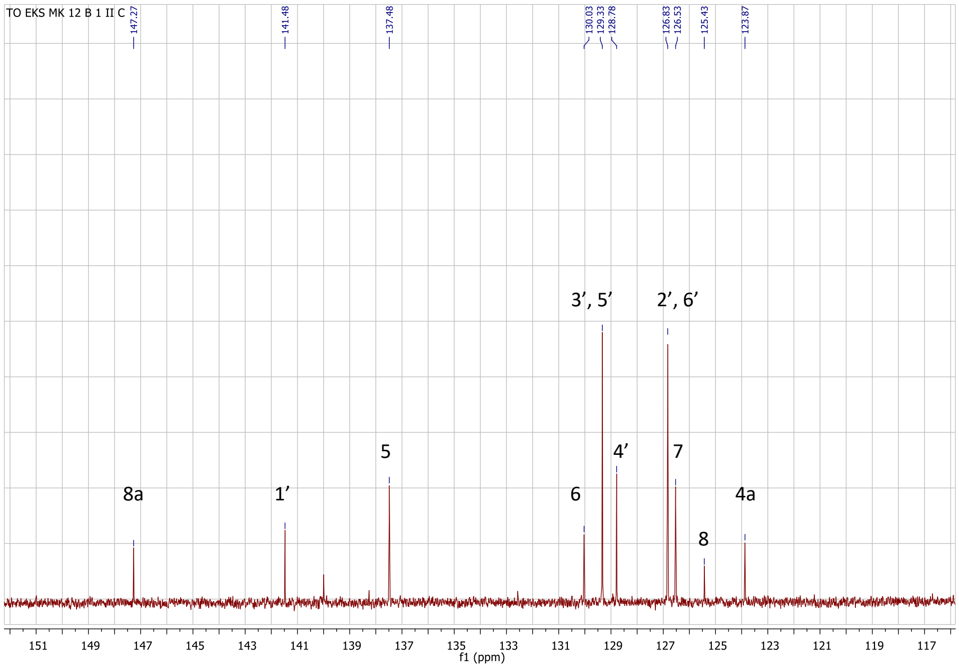

Figure S40. ^{13}C NMR spectrum expansion of 6-methyl-8-nitro-2-phenylchromane 4-*O*-*β*-D-(4”-*O*-methyl)-glucopyranoside **(5a)**

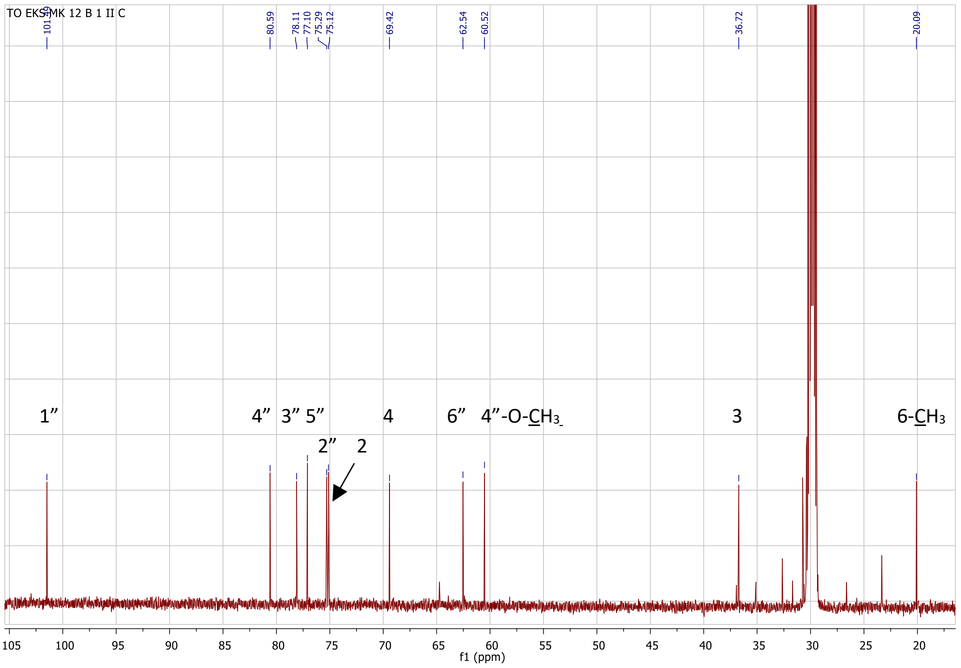

Figure S41. ^{13}C NMR spectrum expansion of 6-methyl-8-nitro-2-phenylchromane 4-*O*-*β*-D-(4"-*O*-methyl)-glucopyranoside **(5a)**

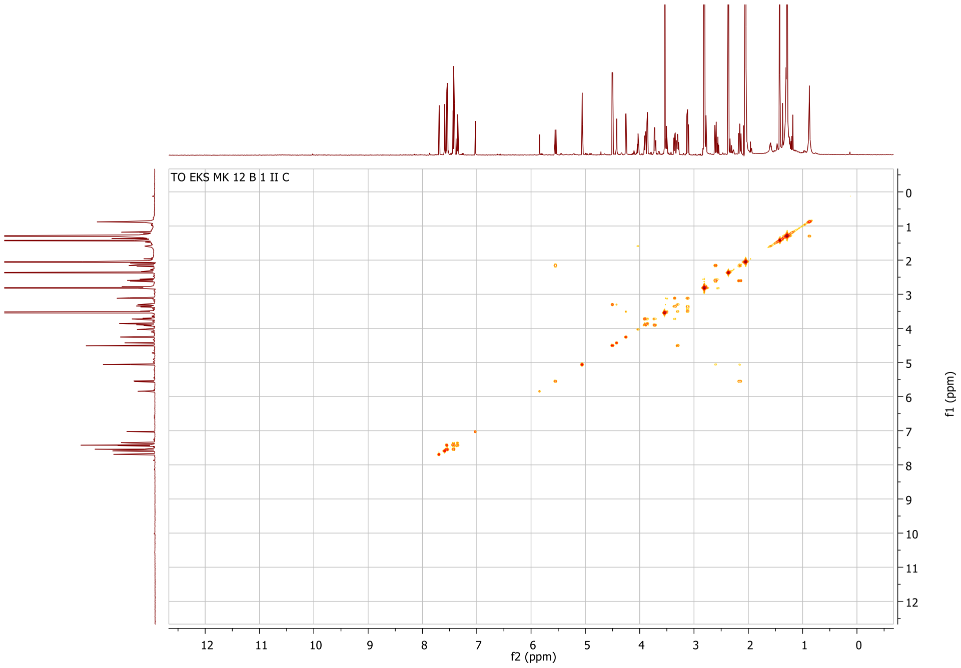

Figure S42. COSY contour map – ^{1}H x ^{13}C of 6-methyl-8-nitro-2-phenylchromane 4-*O*-*β*-D-(4"-*O*-methyl)-glucopyranoside **(5a)**

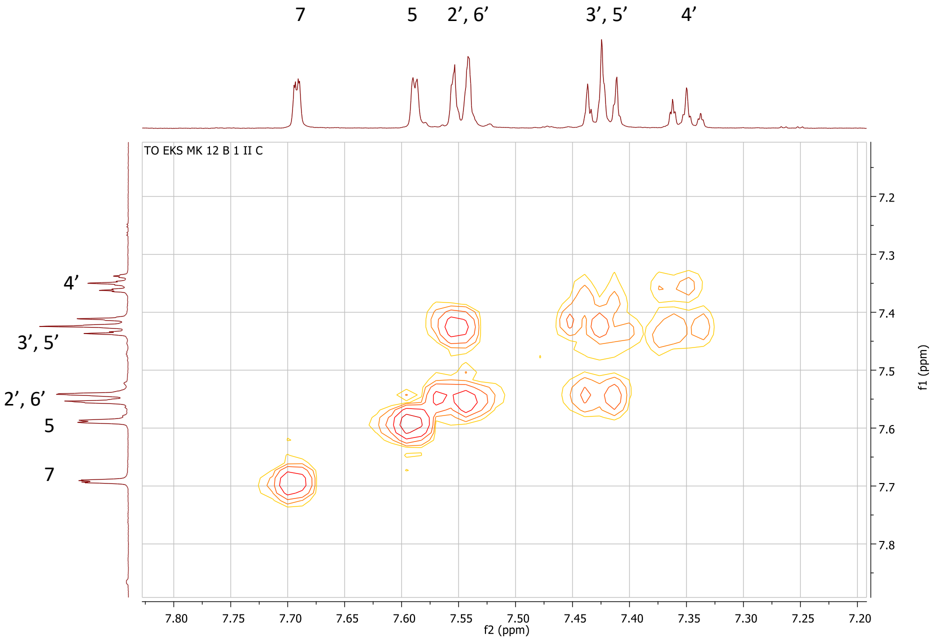

Figure S43. COSY contour map – ^{1}H x ^{13}C expansion of 6-methyl-8-nitro-2-phenylchromane 4-*O*-*β*-D-(4"-*O*-methyl)-glucopyranoside **(5a)**

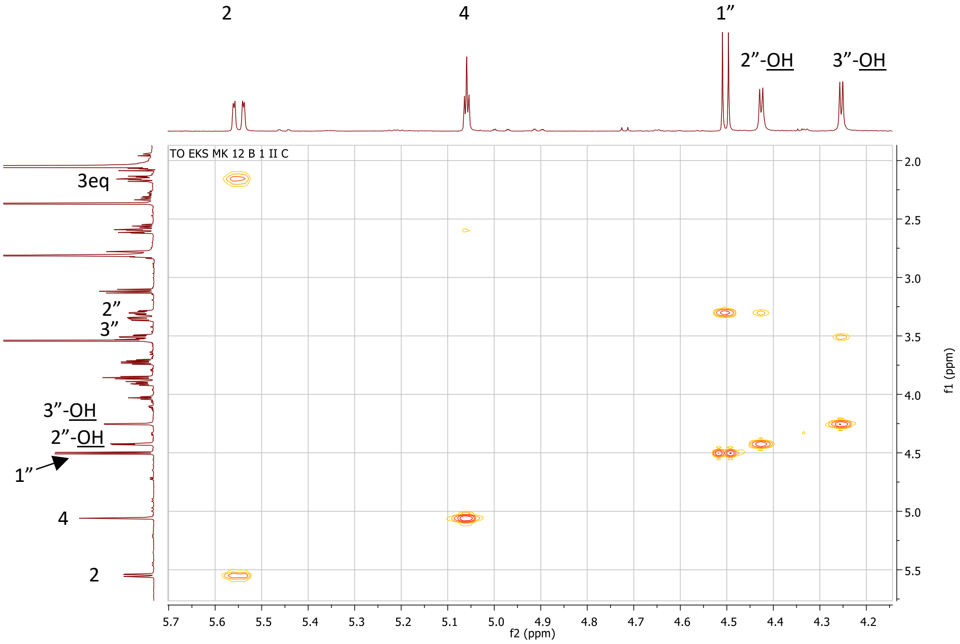

Figure S44. COSY contour map – ^{1}H x ^{13}C expansion of 6-methyl-8-nitro-2-phenylchromane 4-*O*-*β*-D-(4"-*O*-methyl)-glucopyranoside **(5a)**

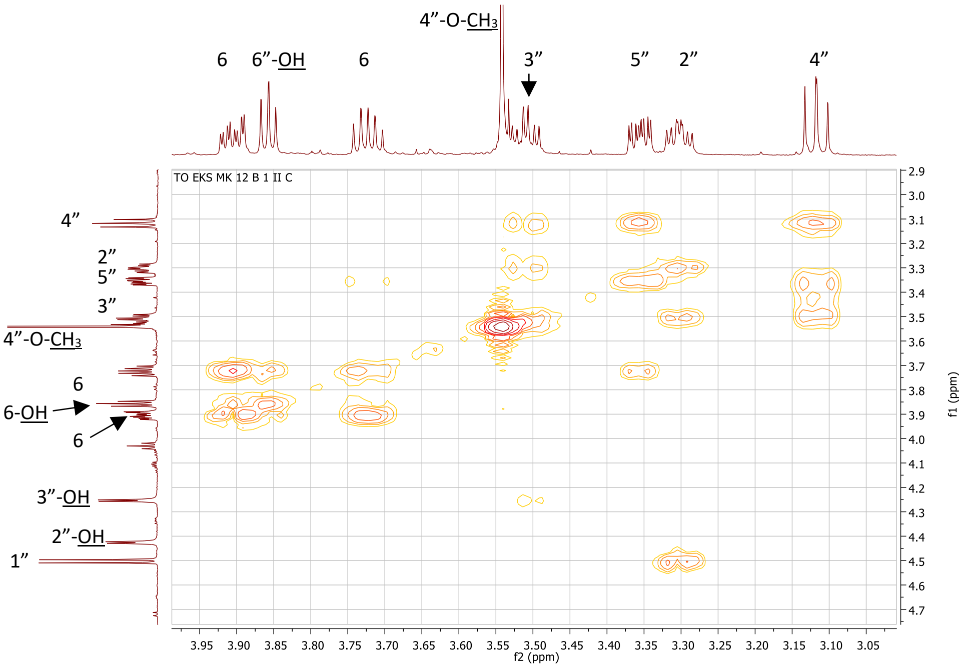

Figure S45. COSY contour map – ^{1}H x ^{13}C expansion of 6-methyl-8-nitro-2-phenylchromane 4-*O*-*β*-D-(4”-*O*-methyl)-glucopyranoside **(5a)**

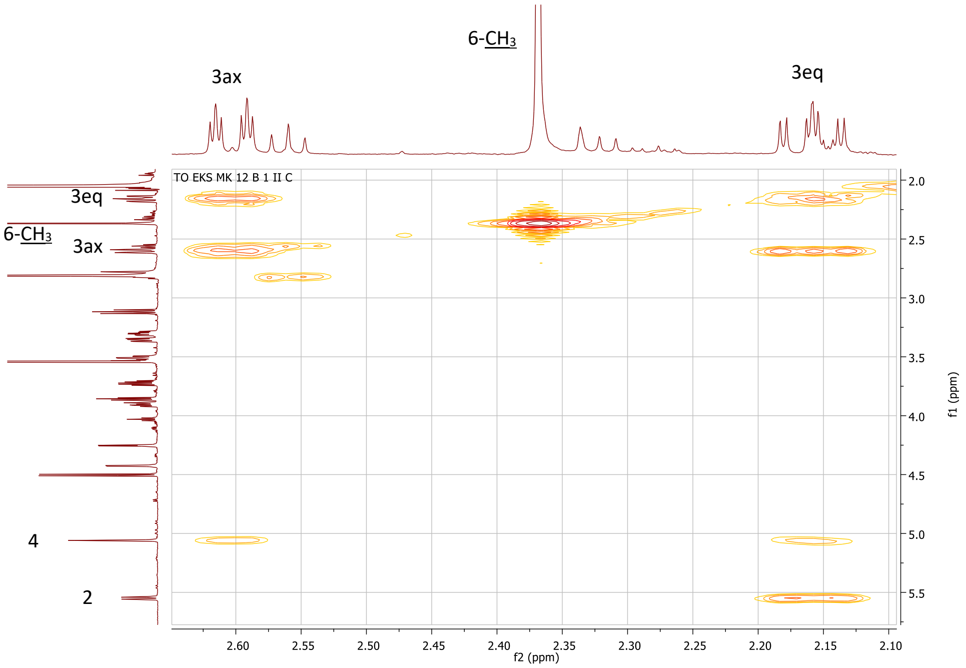

Figure S46. COSY contour map – ^{1}H x ^{13}C expansion of 6-methyl-8-nitro-2-phenylchromane 4-*O*-*β*-D-(4”-*O*-methyl)-glucopyranoside **(5a)**

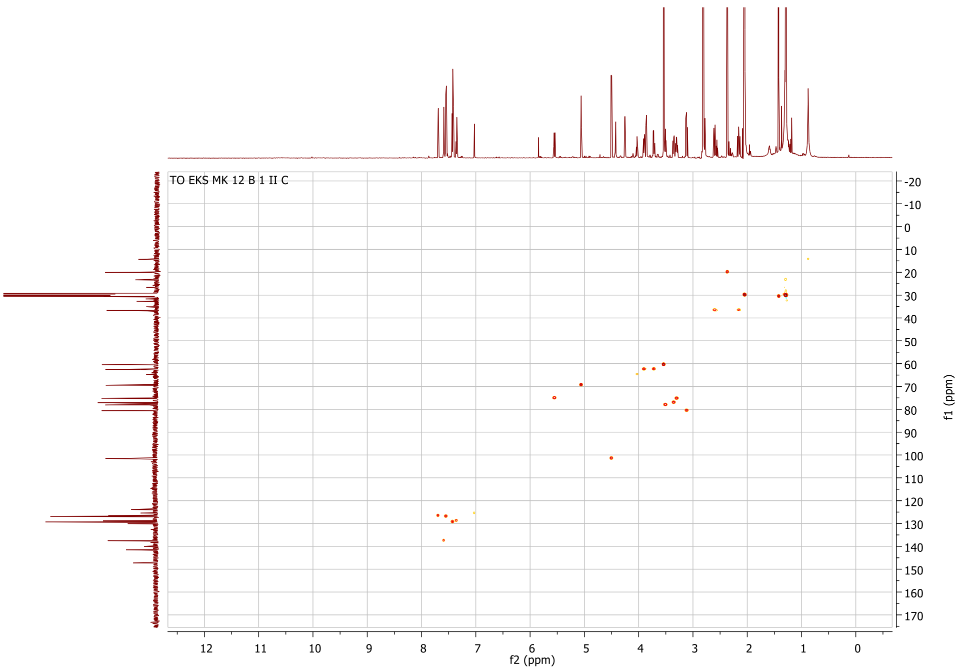

Figure S47. HSQC contour map – ^{1}H x ^{13}C of 6-methyl-8-nitro-2-phenylchromane 4-O-β-D-(4”-O-methyl)-glucopyranoside **(5a)**

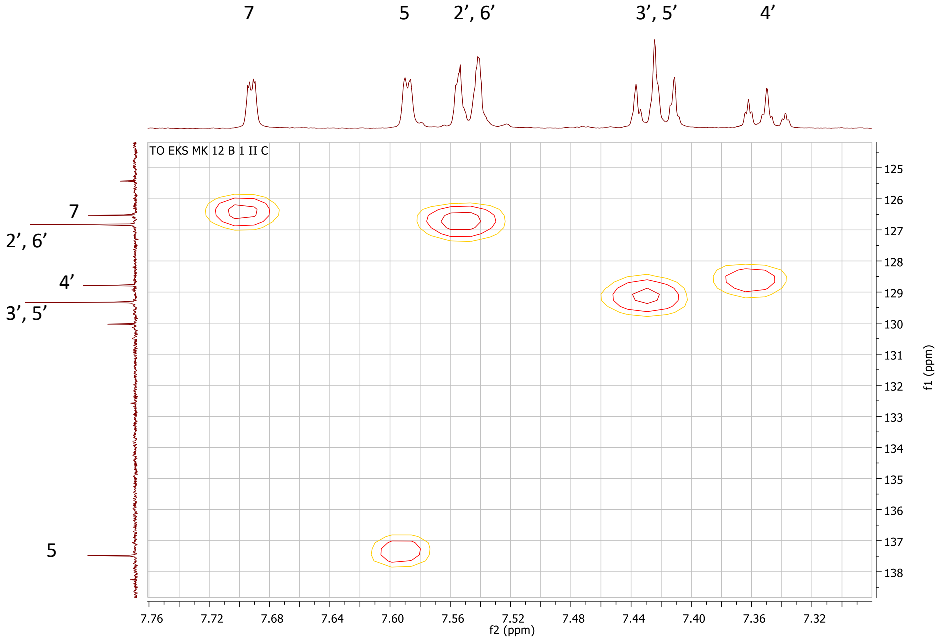

Figure S48. HSQC contour map – ^{1}H x ^{13}C expansion of 6-methyl-8-nitro-2-phenylchromane 4-*O*-*β*-D-(4”-*O*-methyl)-glucopyranoside **(5a)**

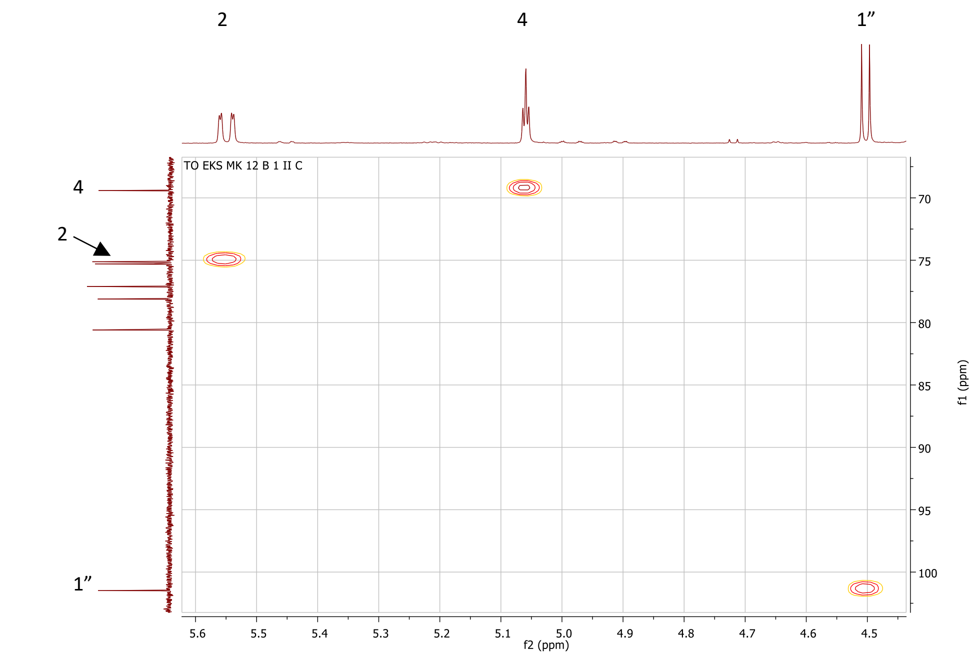

Figure S49. HSQC contour map – ^{1}H x ^{13}C expansion of 6-methyl-8-nitro-2-phenylchromane 4-*O*-*β*-D-(4”-*O*-methyl)-glucopyranoside **(5a)**

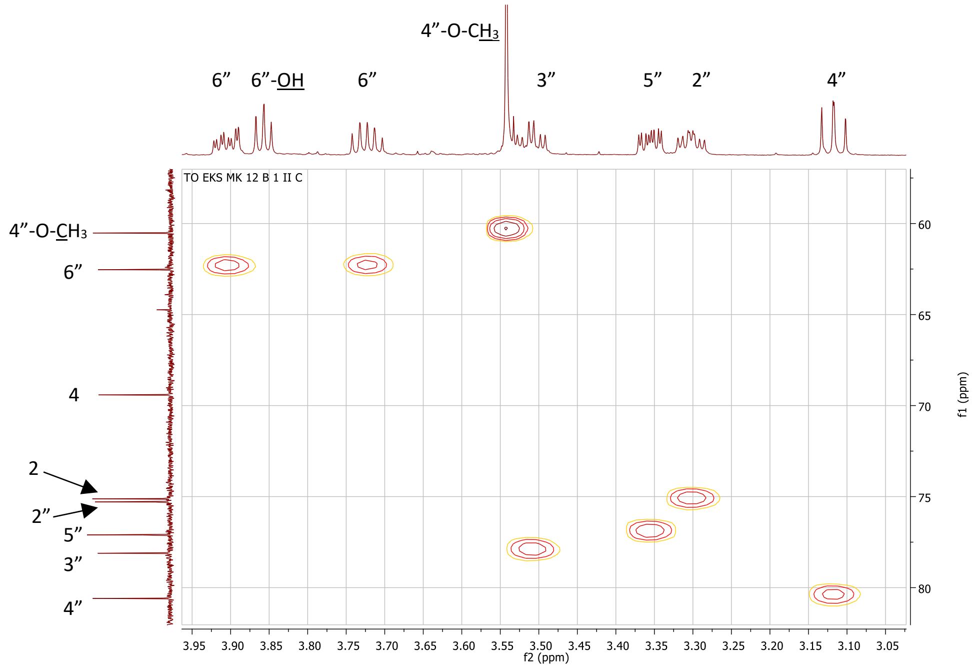

Figure S50. HSQC contour map – ^{1}H x ^{13}C expansion of 6-methyl-8-nitro-2-phenylchromane 4-*O*-*β*-D-(4”-*O*-methyl)-glucopyranoside **(5a)**

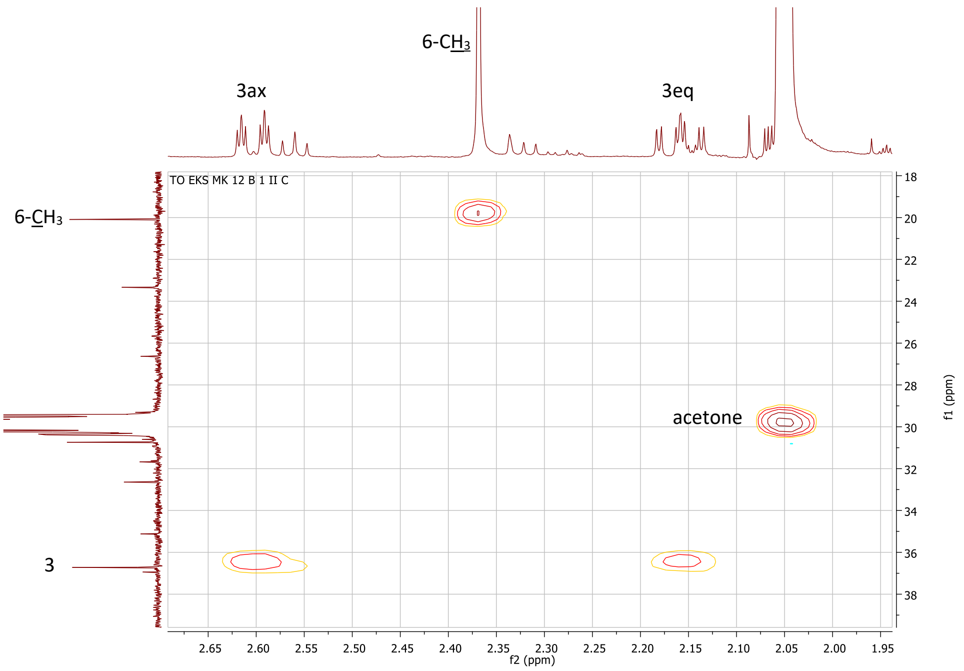

Figure S51. HSQC contour map – ^{1}H x ^{13}C expansion of 6-methyl-8-nitro-2-phenylchromane 4-*O*-*β*-D-(4”-*O*-methyl)-glucopyranoside **(5a)**

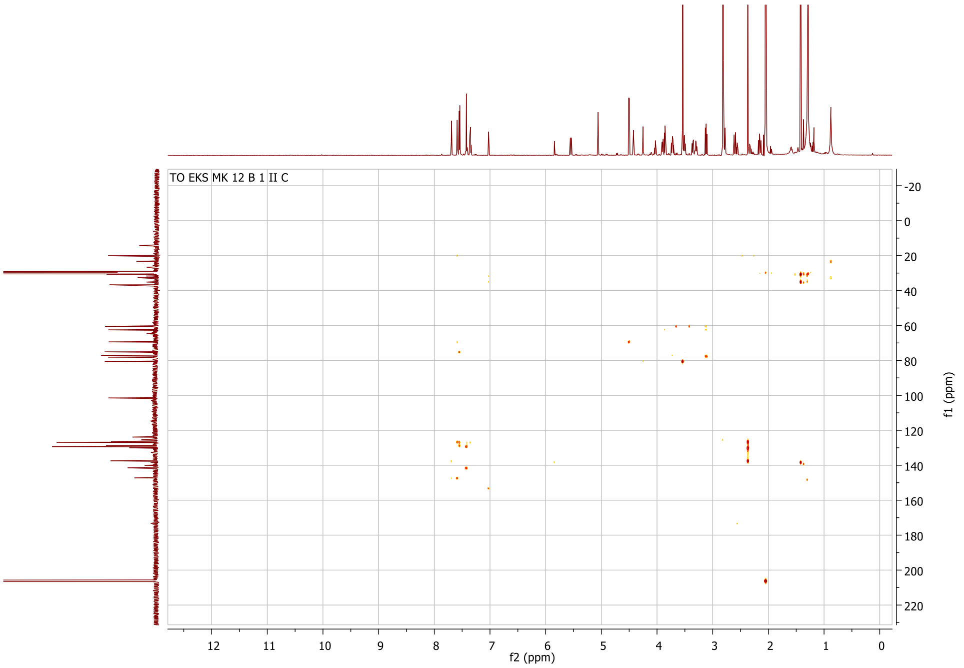

Figure S52. HMBC contour map – ^{1}H x ^{13}C of 6-methyl-8-nitro-2-phenylchromane 4-*O*-*β*-D-(4”-*O*-methyl)-glucopyranoside **(5a)**

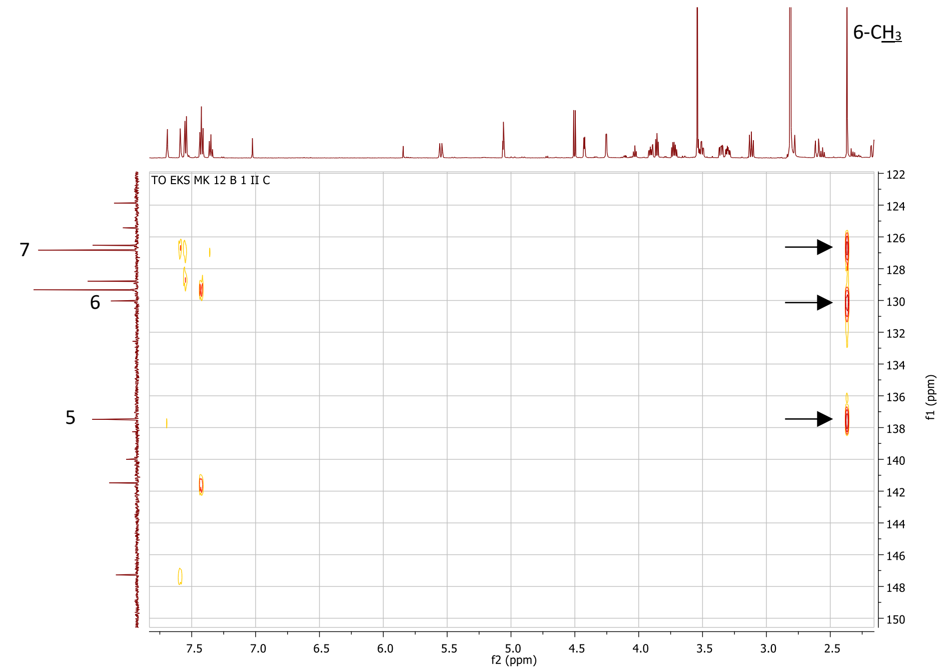

Figure S53. HMBC contour map – ^{1}H x ^{13}C expansion of 6-methyl-8-nitro-2-phenylchromane 4-*O*-*β*-D-(4”-*O*-methyl)-glucopyranoside **(5a)**

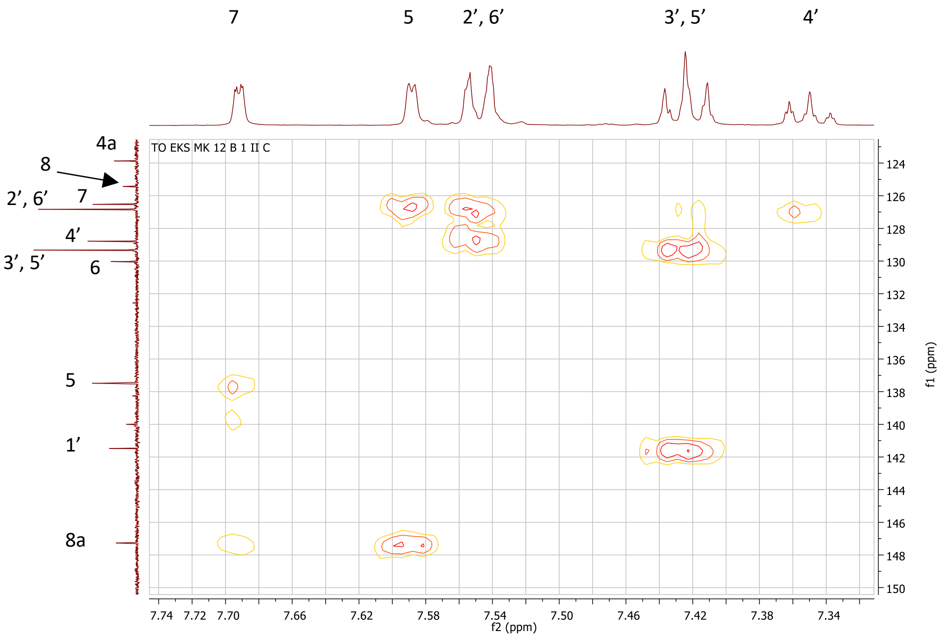

Figure S54. HMBC contour map – ^{1}H x ^{13}C expansion of 6-methyl-8-nitro-2-phenylchromane 4-*O*-*β*-D-(4”-*O*-methyl)-glucopyranoside **(5a)**

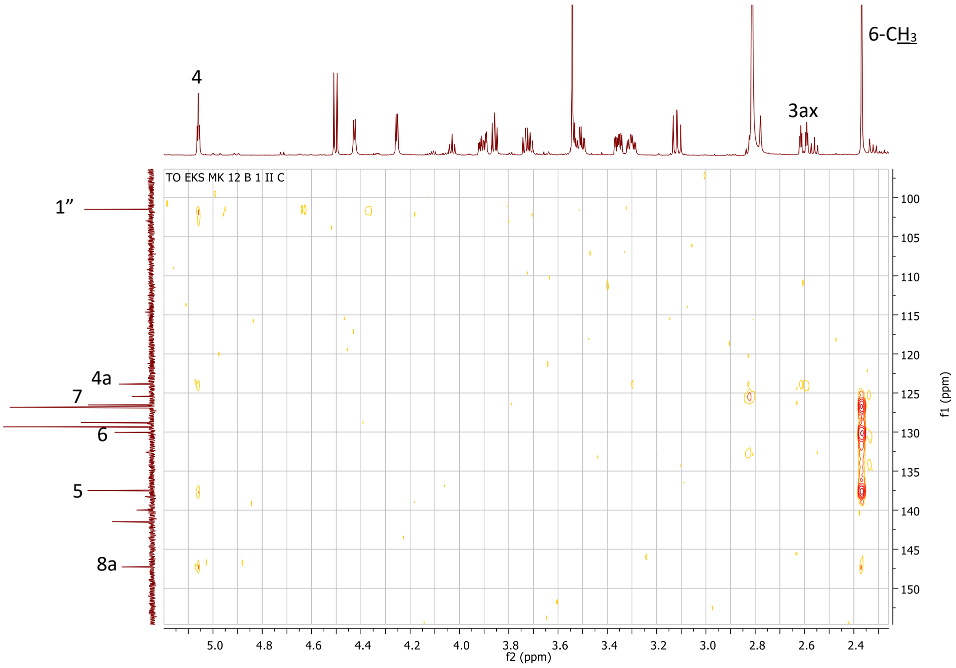

Figure S55. HMBC contour map – ^{1}H x ^{13}C expansion of 6-methyl-8-nitro-2-phenylchromane 4-O-β-D-(4”-O-methyl)-glucopyranoside **(5a)**

Molecular Formula: $C_{23}H_{27}NO_{10}$
Formula Weight: 477.461
Ionization mode: positive
Precursor $[M + H]^{+}$: 478.164
Collision energy (CE): -10.0

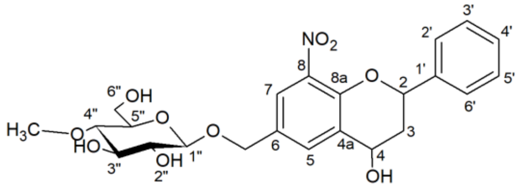

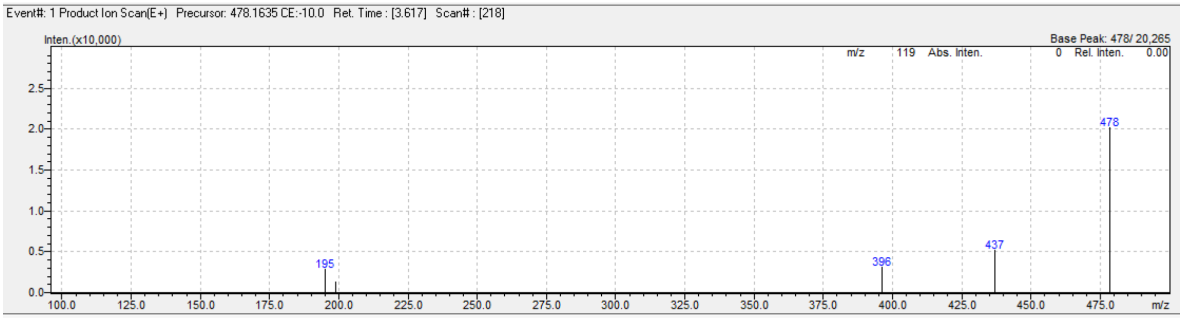

Figure S56. MS analysis of 8-nitroflavan-4-ol 6-methylene-*O*-*β*-D-(4”-*O*-methyl)-glucopyranoside **(5b)**

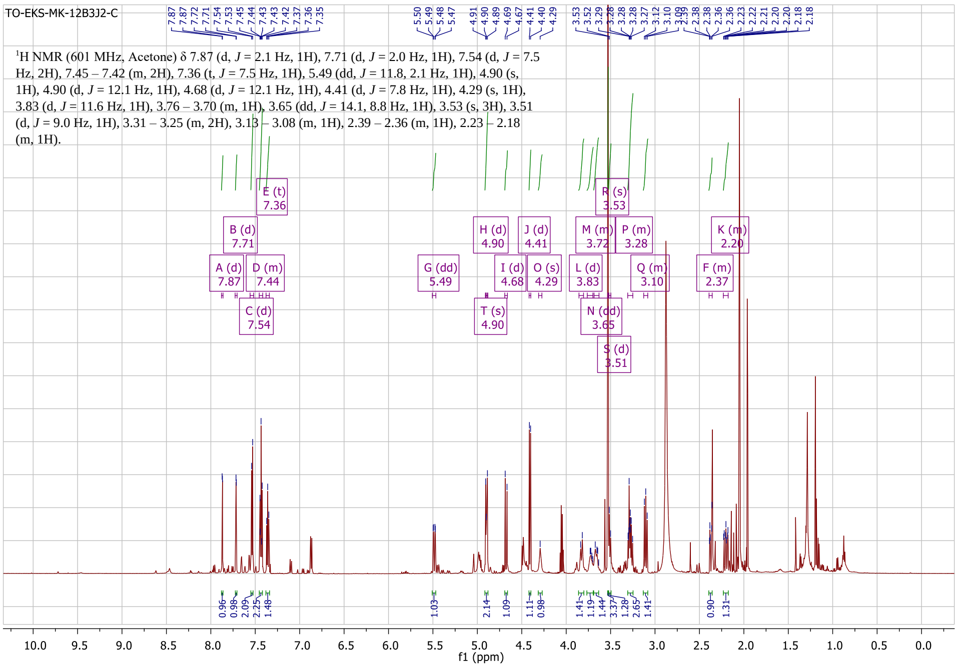

Figure S57. ^{1}H NMR spectrum of 8-nitroflavan-4-ol 6-methylene-*O*-*β*-D-(4”-*O*-methyl)-glucopyranoside **(5b)**

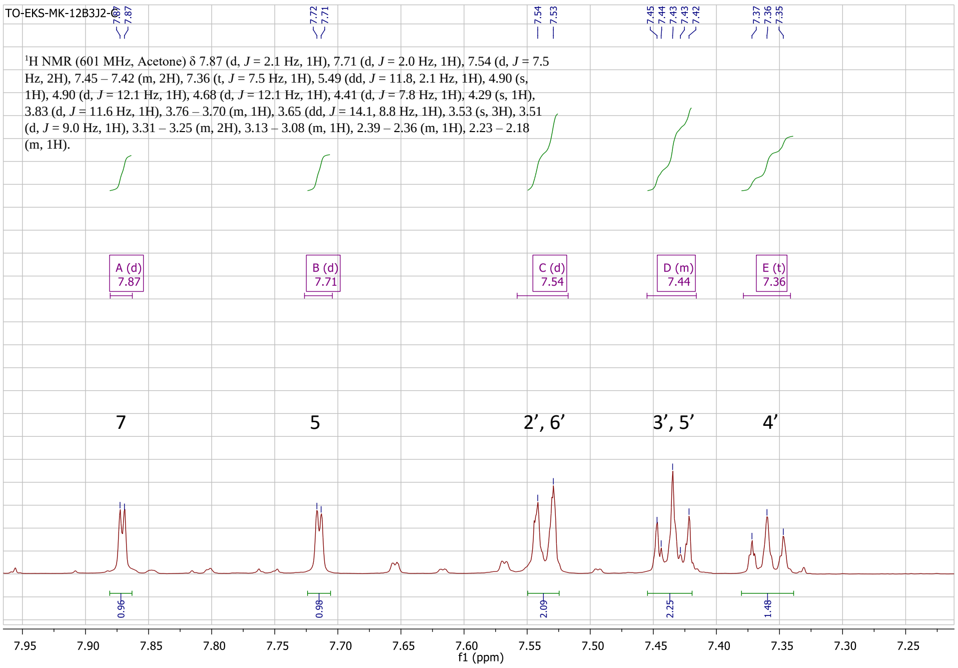

Figure S58. ^{1}H NMR spectrum expansion of 8-nitroflavan-4-ol 6-methylene-*O*-*β*-D-(4”-*O*-methyl)-glucopyranoside **(5b)**

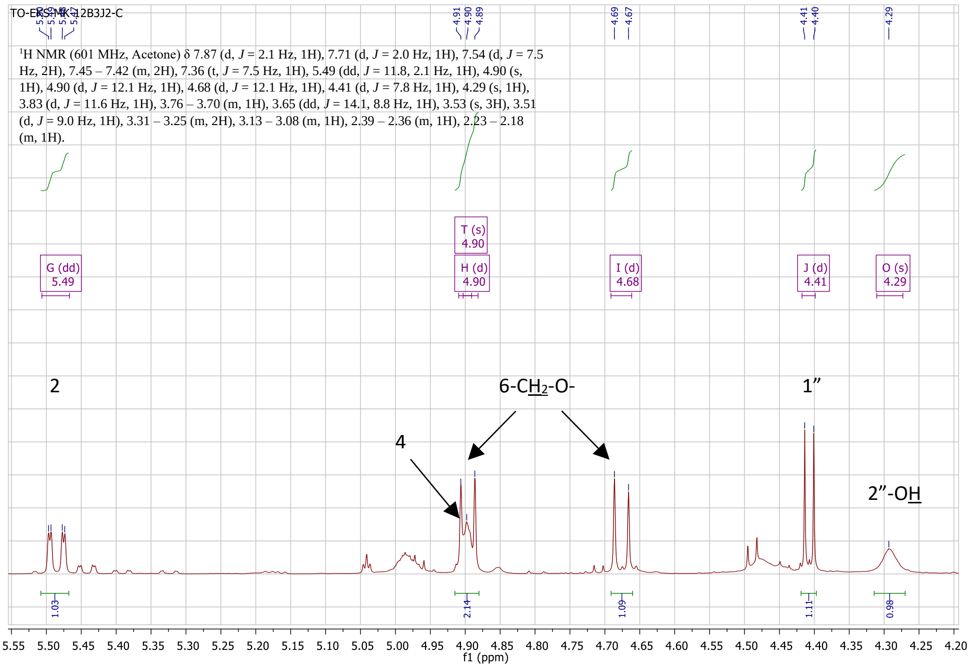

Figure S59. ¹H NMR spectrum expansion of 8-nitroflavan-4-ol 6-methylene-*O*-*β*-D-(4”-*O*-methyl)-glucopyranoside **(5b)**

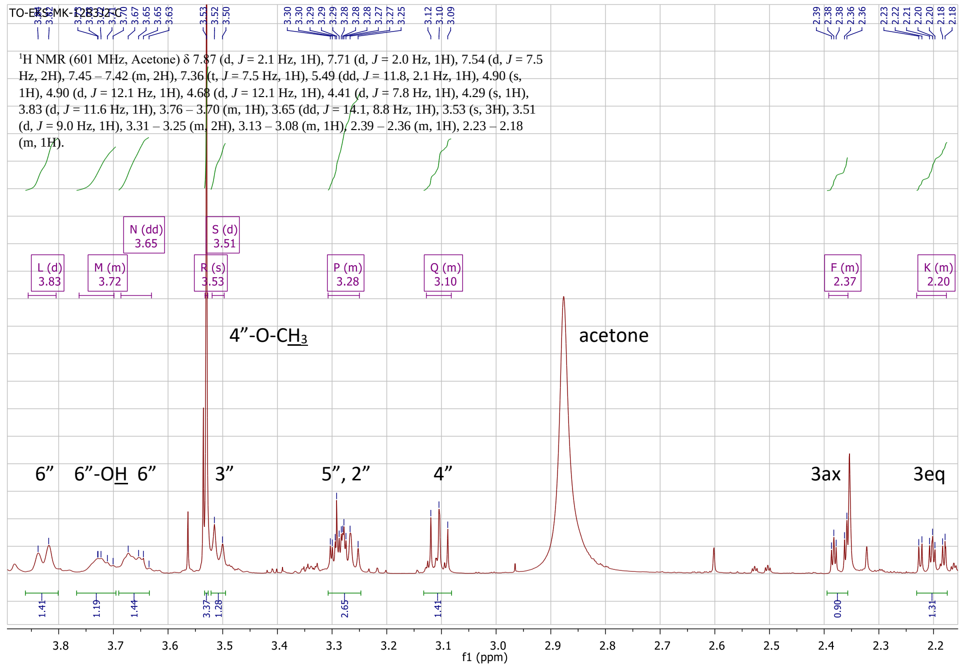

Figure S60. ¹H NMR spectrum expansion of 8-nitroflavan-4-ol 6-methylene-*O*-*β*-D-(4”-*O*-methyl)-glucopyranoside **(5b)**

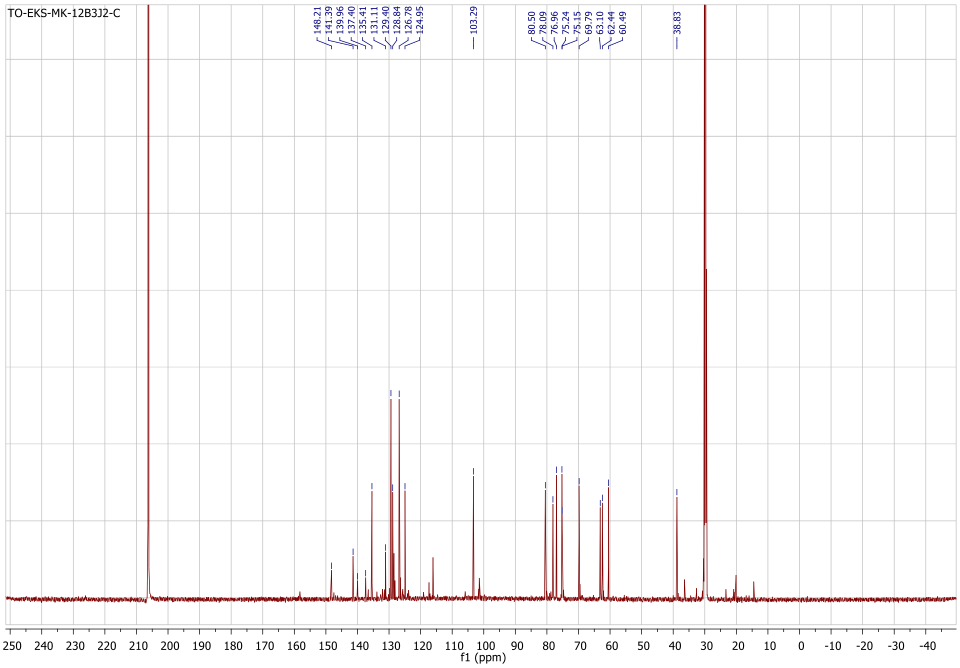

Figure S61. ^{13}C NMR spectrum of 8-nitroflavan-4-ol 6-methylene-*O*-*β*-D-(4”-*O*-methyl)-glucopyranoside **(5b)**

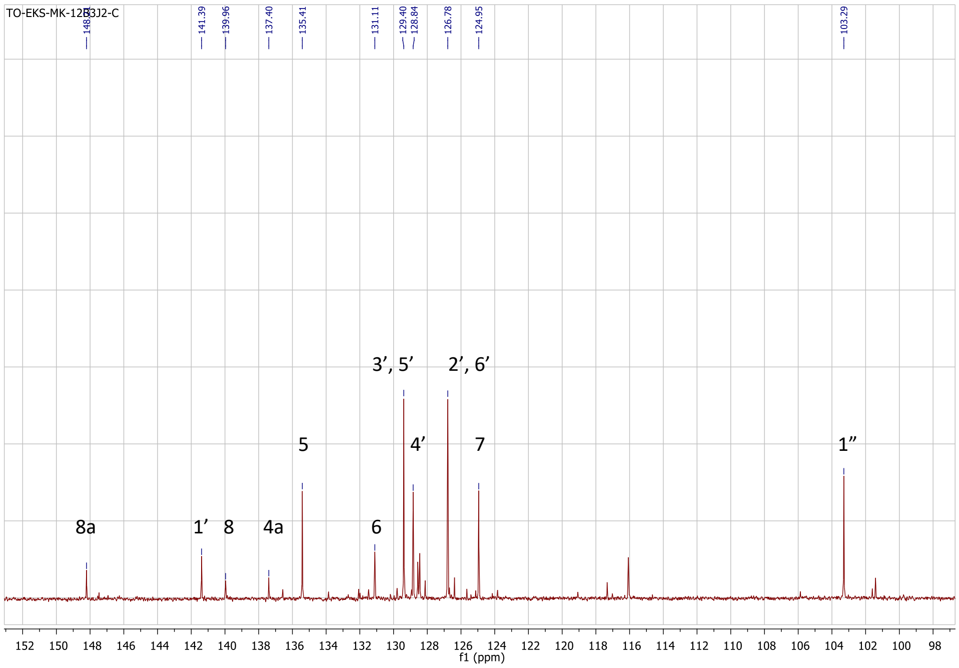

Figure S62. ^{13}C NMR spectrum expansion of 8-nitroflavan-4-ol 6-methylene-*O*-*β*-D-(4”-*O*-methyl)-glucopyranoside **(5b)**

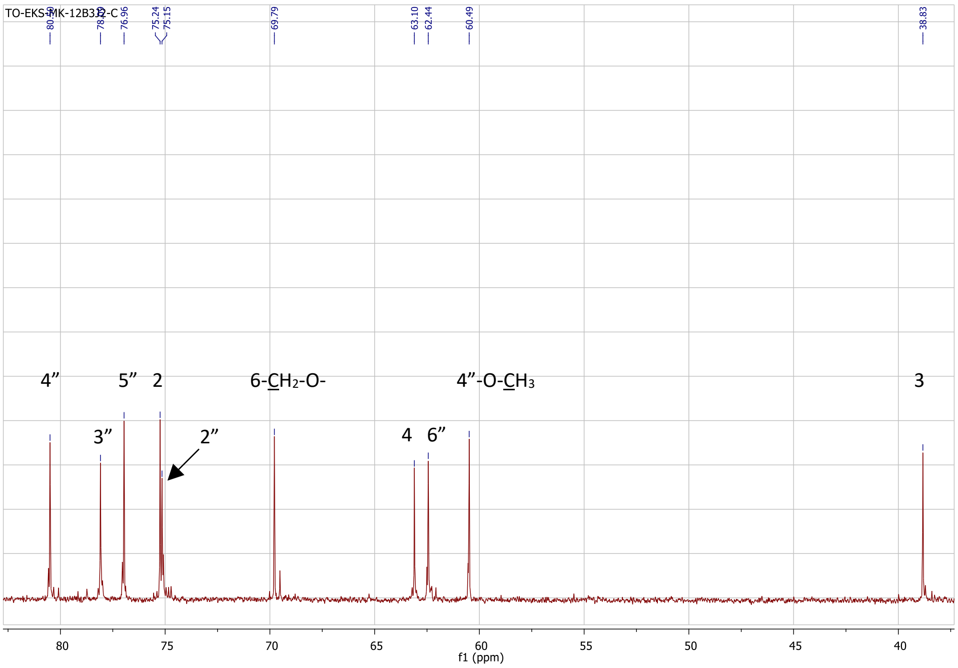

Figure S63. ^{13}C NMR spectrum expansion of 8-nitroflavan-4-ol 6-methylene-*O*-*β*-D-(4"-*O*-methyl)-glucopyranoside **(5b)**

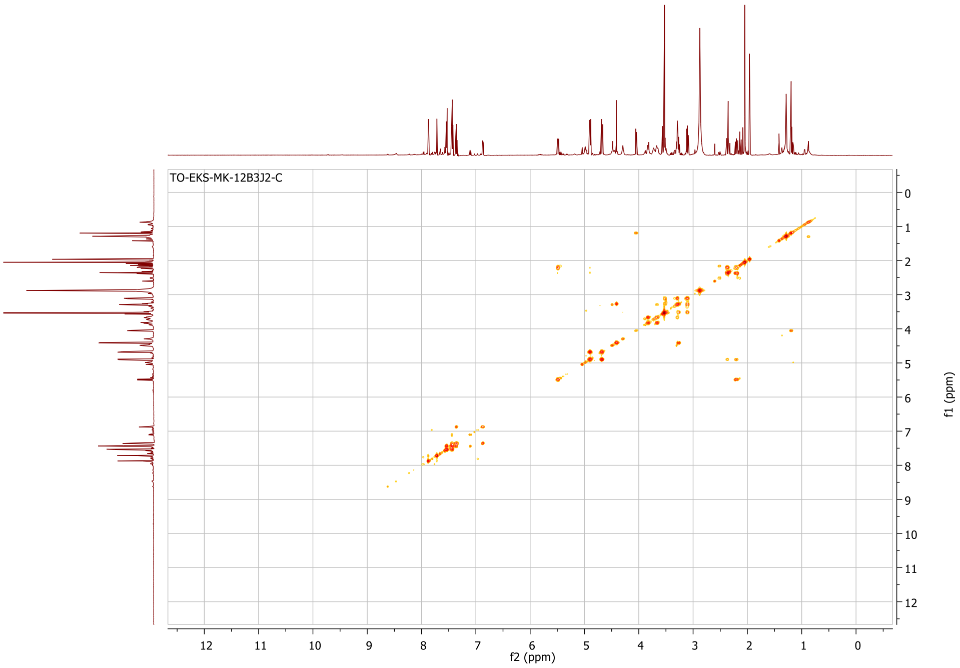

Figure S64. COSY contour map – ^{1}H x ^{1}H of 8-nitroflavan-4-ol 6-methylene-*O*-*β*-D-(4"-*O*-methyl)-glucopyranoside **(5b)**

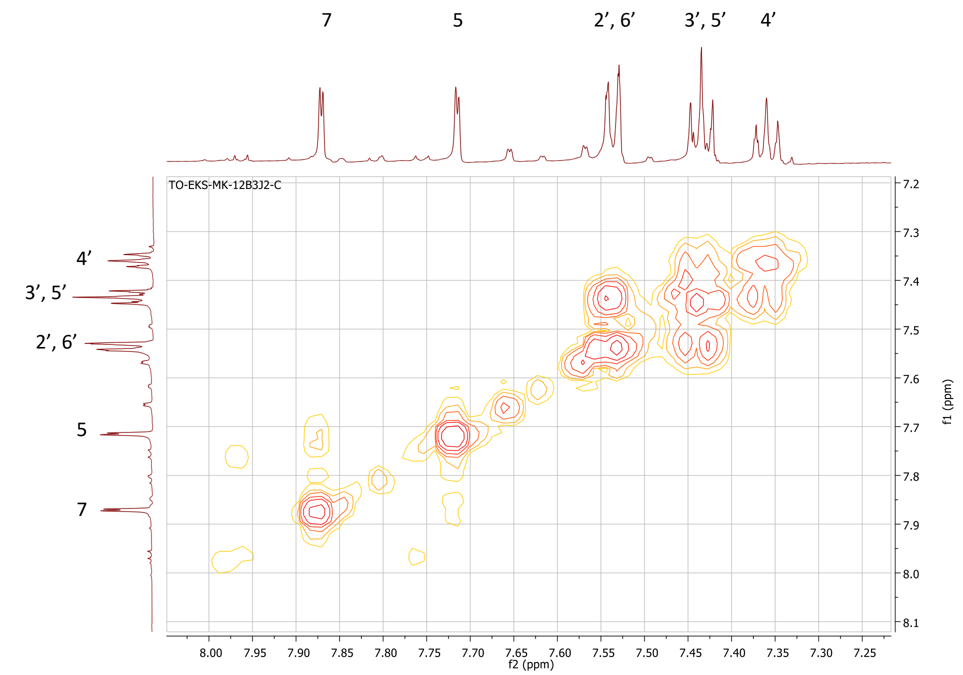

Figure S65. COSY contour map – ^{1}H x ^{1}H expansion of 8-nitroflavan-4-ol 6-methylene-*O*-*β*-D-(4”-*O*-methyl)-glucopyranoside **(5b)**

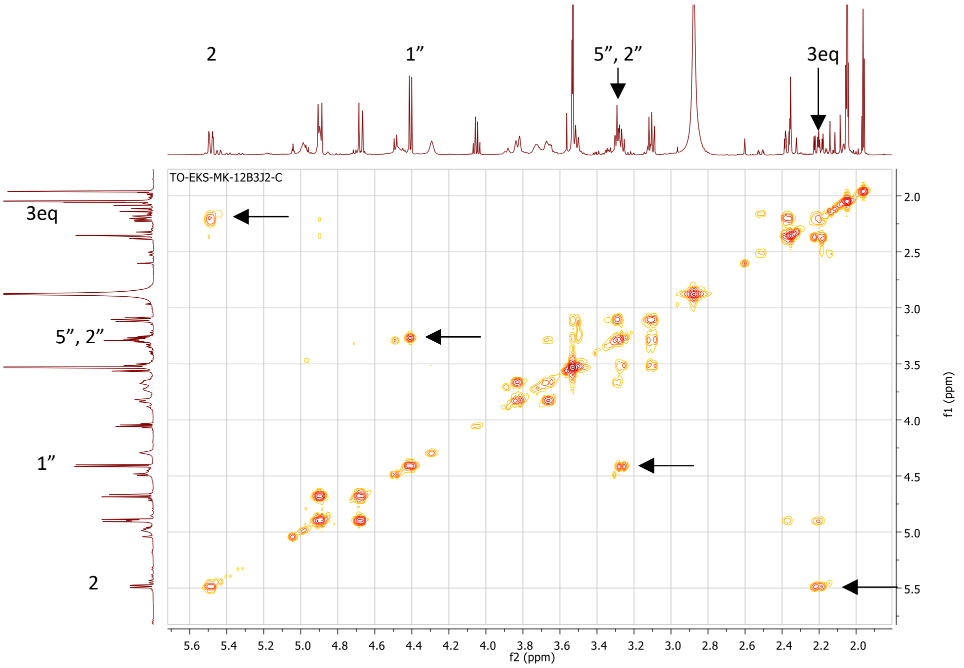

Figure S66. COSY contour map – ^{1}H x ^{1}H expansion of 8-nitroflavan-4-ol 6-methylene-*O*-*β*-D-(4”-*O*-methyl)-glucopyranoside **(5b)**

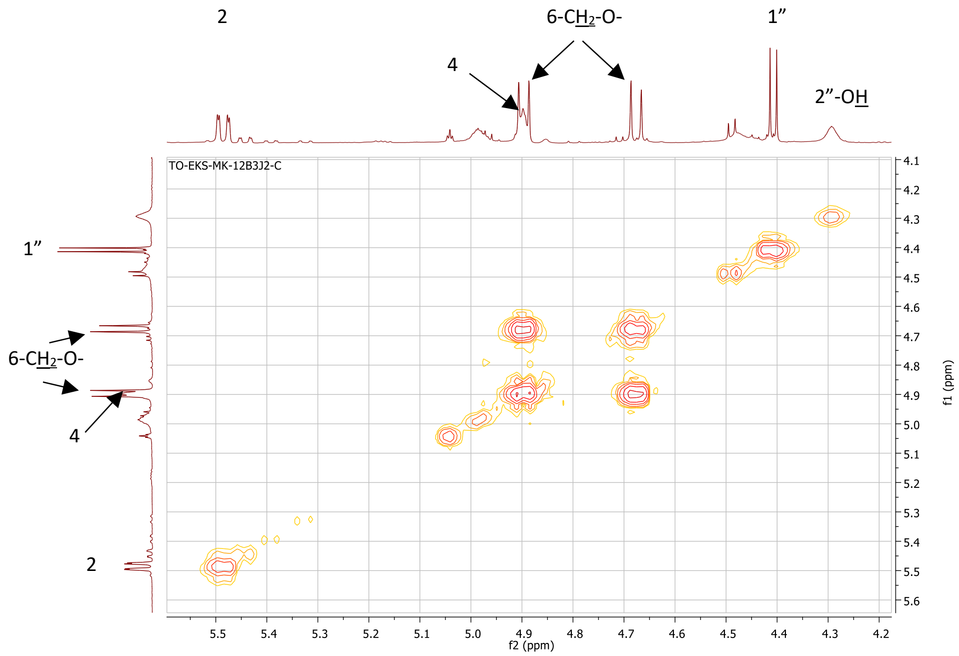

Figure S67. COSY contour map – ^{1}H x ^{1}H expansion of 8-nitroflavan-4-ol 6-methylene-*O*-*β*-D-(4”-*O*-methyl)-glucopyranoside **(5b)**

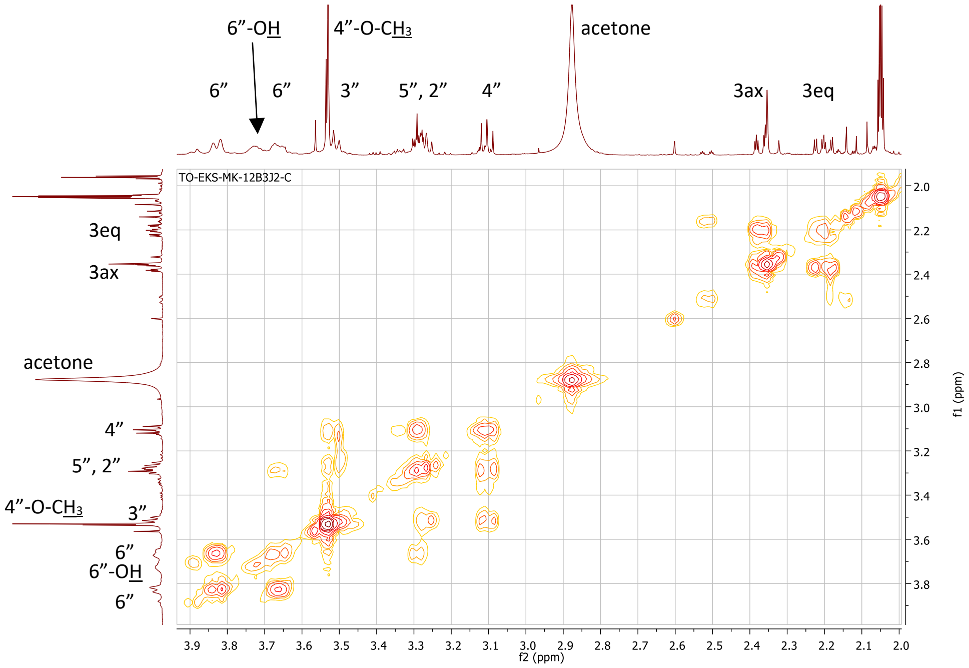

Figure S68. COSY contour map – ^{1}H x ^{1}H expansion of 8-nitroflavan-4-ol 6-methylene-*O*-*β*-D-(4”-*O*-methyl)-glucopyranoside **(5b)**

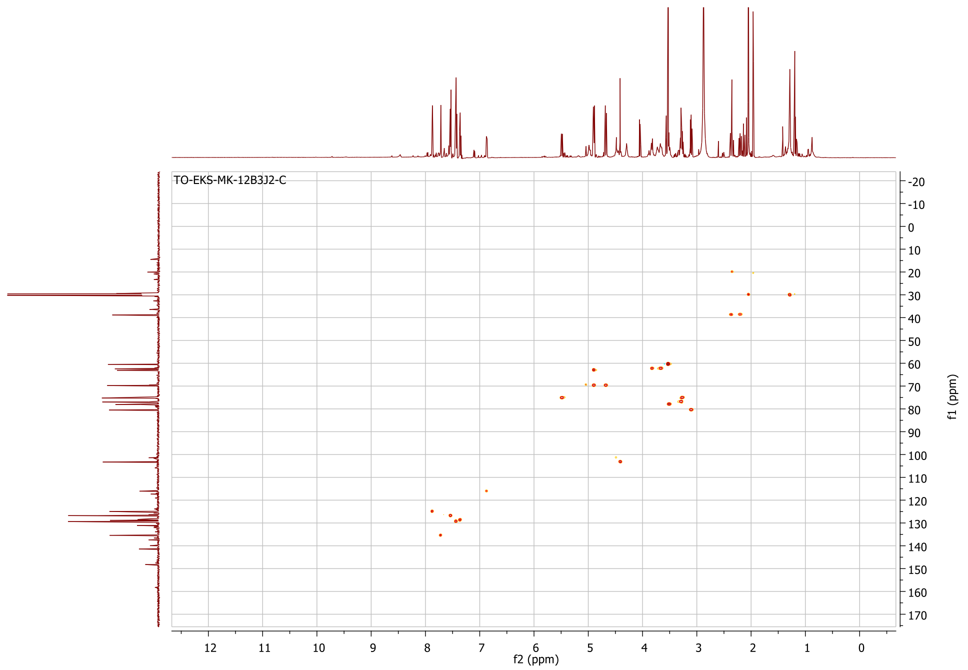

Figure S69. HSQC contour map – ^{1}H x ^{13}C of 8-nitroflavan-4-ol 6-methylene-*O*-*β*-D-(4”-*O*-methyl)-glucopyranoside **(5b)**

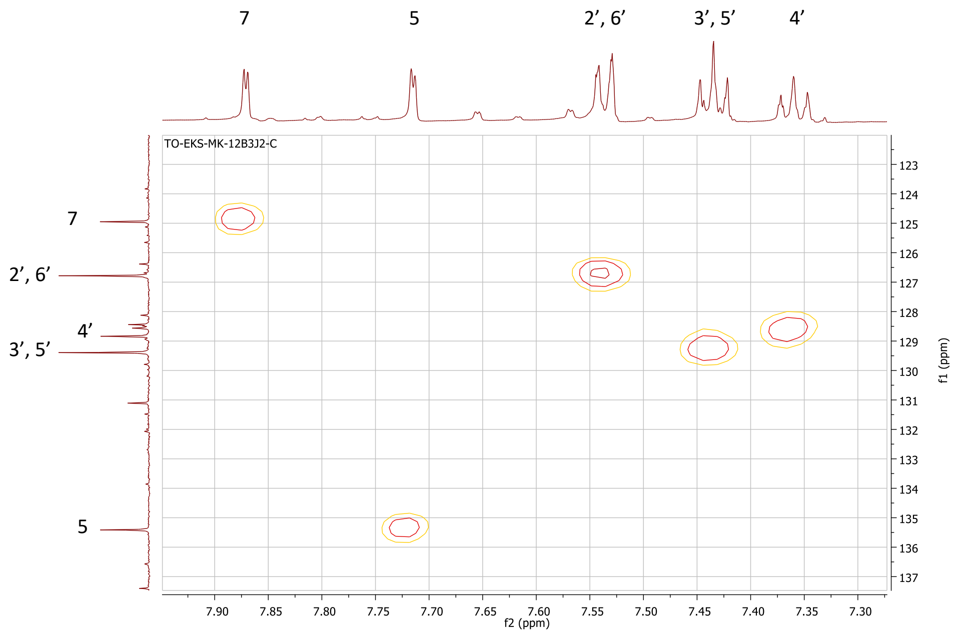

Figure S70. HSQC contour map – ^{1}H x ^{13}C expansion of 8-nitroflavan-4-ol 6-methylene-*O*-*β*-D-(4”-*O*-methyl)-glucopyranoside **(5b)**

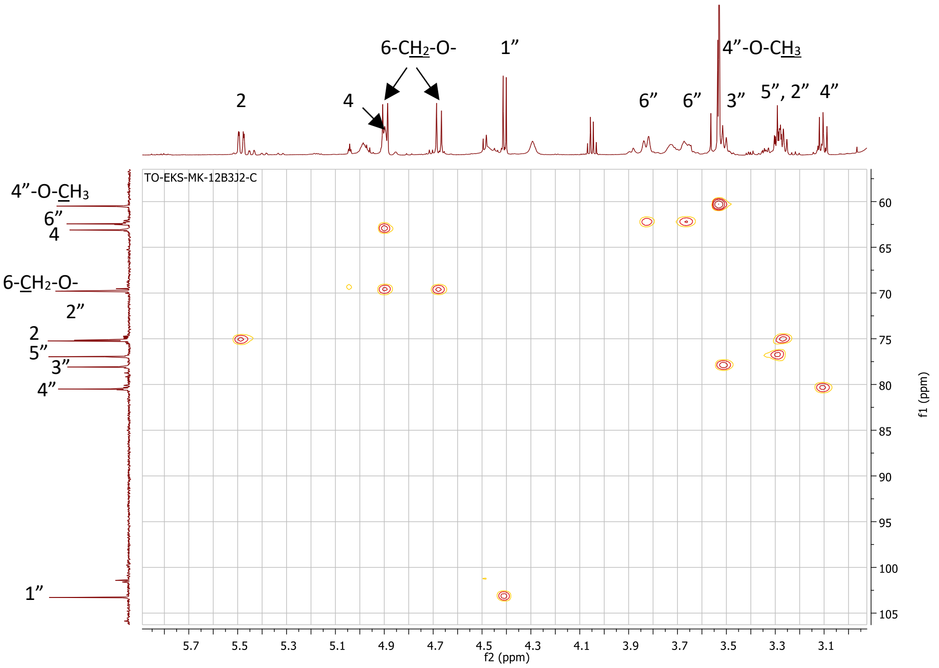

Figure S71. HSQC contour map – ^{1}H x ^{13}C expansion of 8-nitroflavan-4-ol 6-methylene-*O*-*β*-D-(4”-*O*-methyl)-glucopyranoside **(5b)**

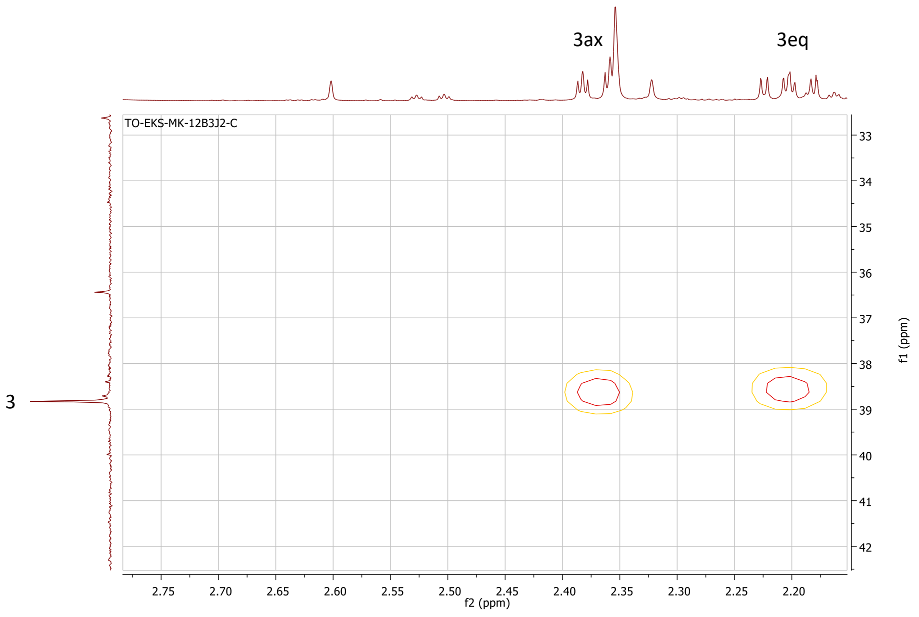

Figure S72. HSQC contour map – ^{1}H x ^{13}C expansion of 8-nitroflavan-4-ol 6-methylene-*O*-*β*-D-(4”-*O*-methyl)-glucopyranoside **(5b)**

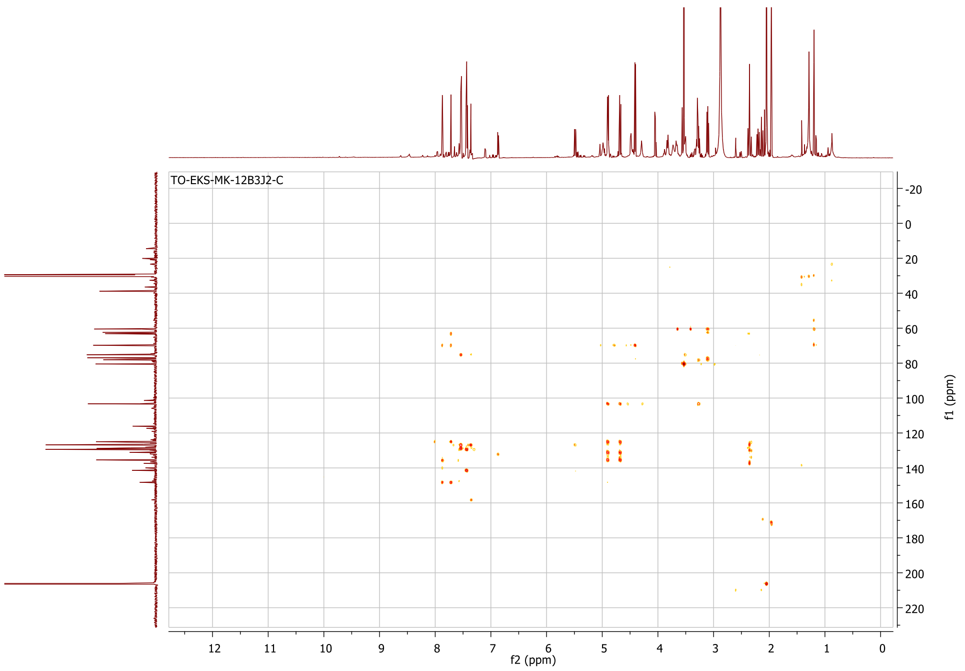

Figure S73. HMBC contour map – ^{1}H x ^{13}C of 8-nitroflavan-4-ol 6-methylene-*O*-*β*-D-(4''-*O*-methyl)-glucopyranoside **(5b)**

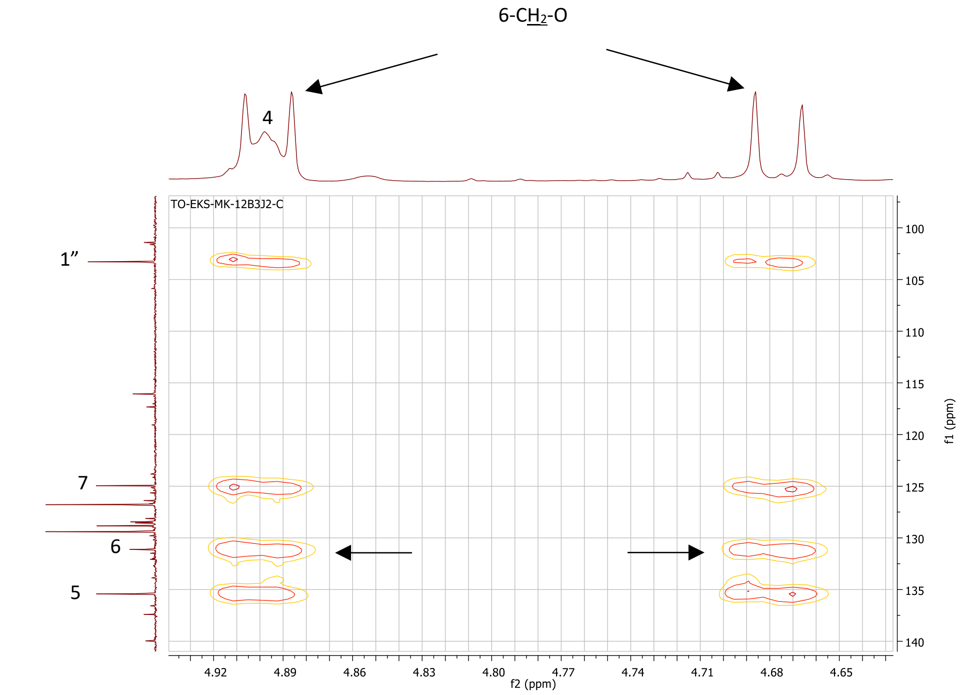

Figure S74. HMBC contour map – ^{1}H x ^{13}C expansion of 8-nitroflavan-4-ol 6-methylene-*O*-*β*-D-(4''-*O*-methyl)-glucopyranoside **(5b)**

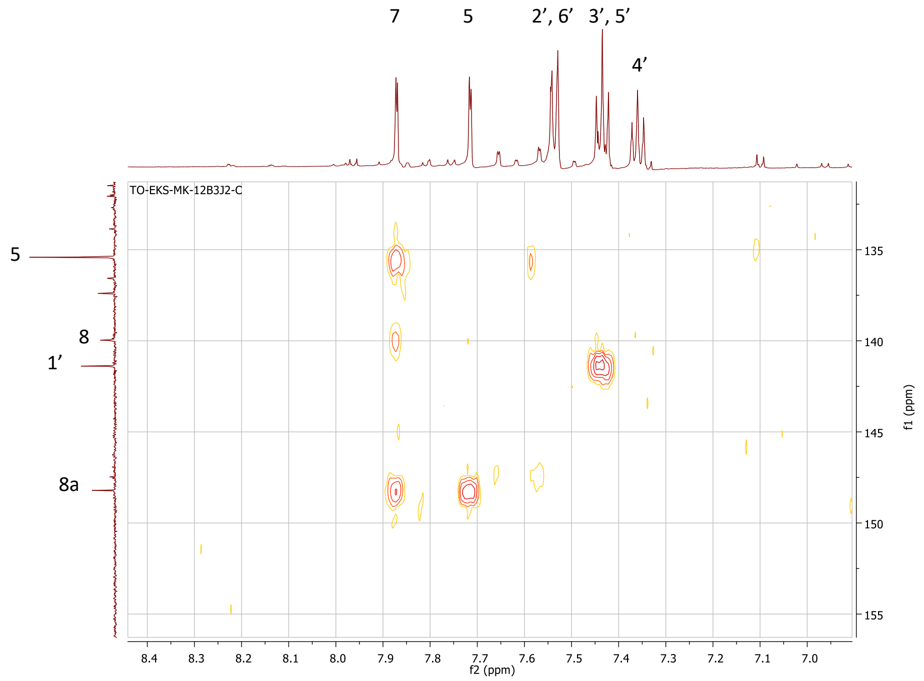

Figure S75. HMBC contour map – ^{1}H x ^{13}C expansion of 8-nitroflavan-4-ol 6-methylene-*O*-*β*-D-(4”-*O*-methyl)-glucopyranoside **(5b)**

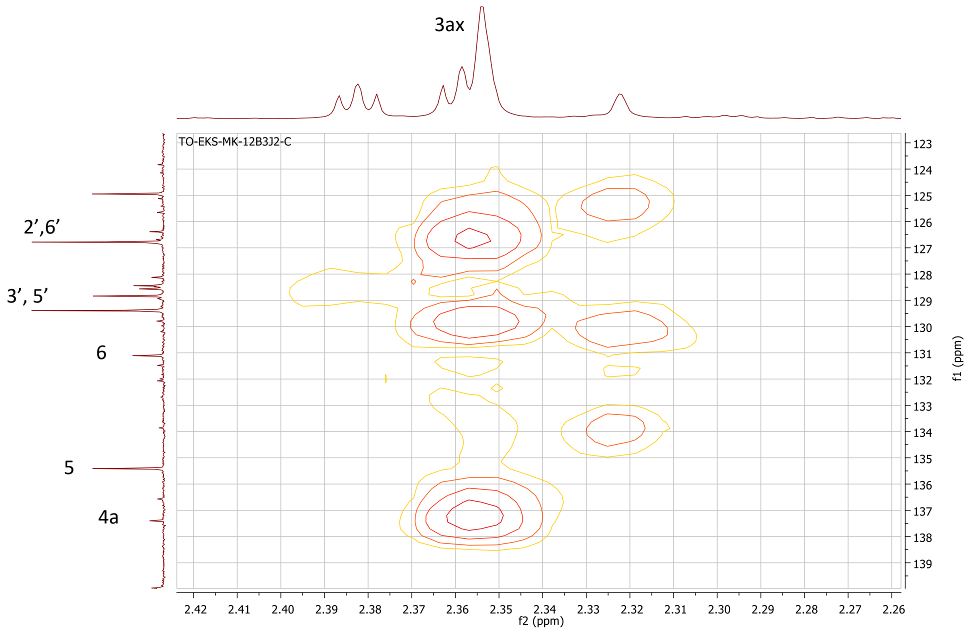

Figure S76. HMBC contour map – ^{1}H x ^{13}C expansion of 8-nitroflavan-4-ol 6-methylene-*O*-*β*-D-(4”-*O*-methyl)-glucopyranoside **(5b)**

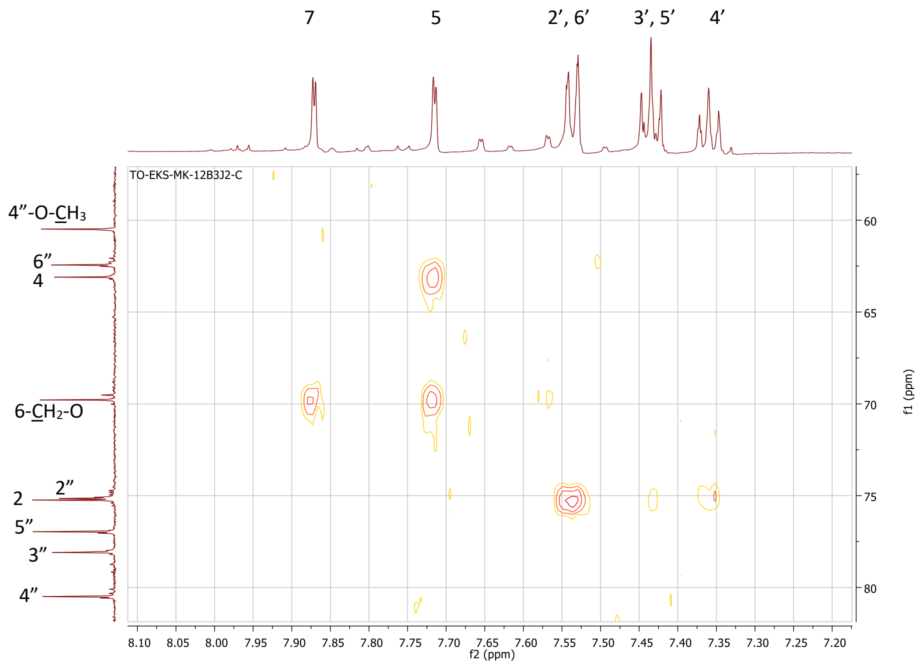

Figure S77. HMBC contour map – ^{1}H x ^{13}C expansion of 8-nitroflavan-4-ol 6-methylene-*O*-*β*-D-(4”-*O*-methyl)-glucopyranoside **(5b)**

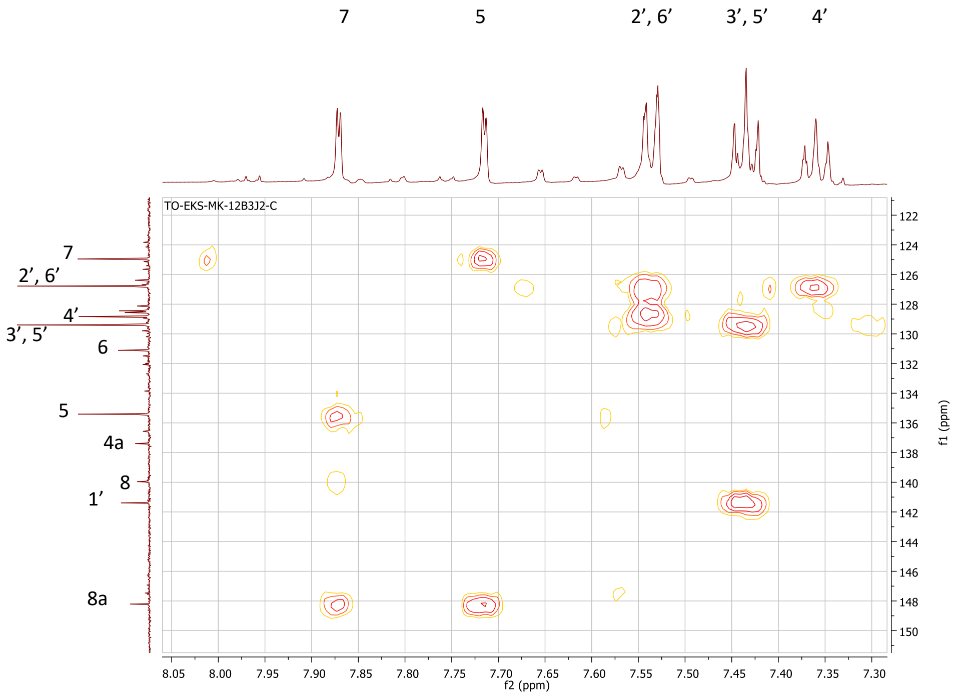

Figure S78. HMBC contour map – ^{1}H x ^{13}C expansion of 8-nitroflavan-4-ol 6-methylene-*O*-*β*-D-(4”-*O*-methyl)-glucopyranoside **(5b)**

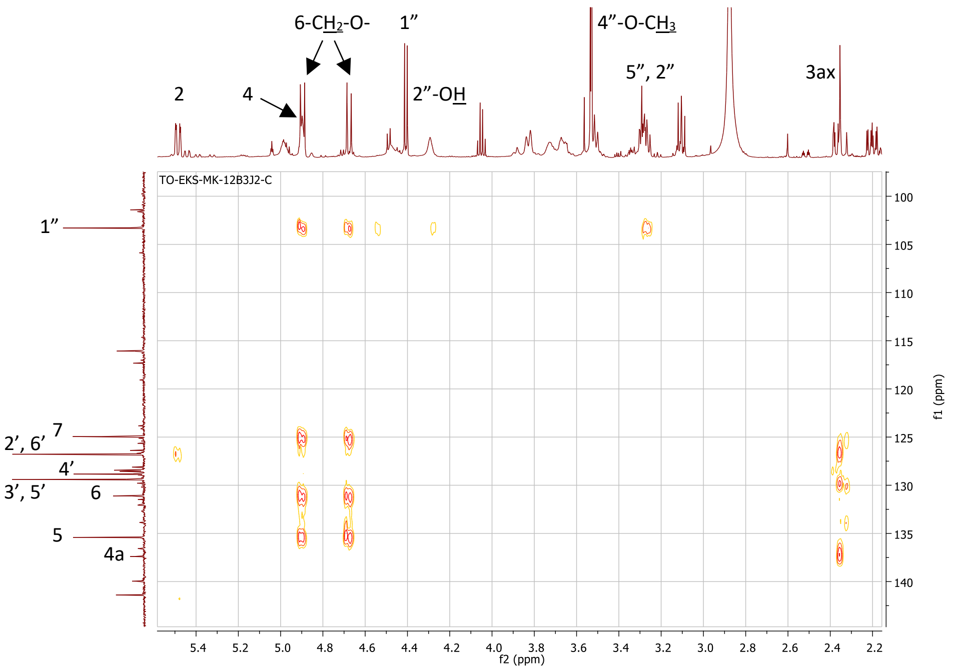

Figure S79. HMBC contour map – ^{1}H x ^{13}C expansion of 8-nitroflavan-4-ol 6-methylene-*O*-*β*-D-(4"-*O*-methyl)-glucopyranoside **(5b)**

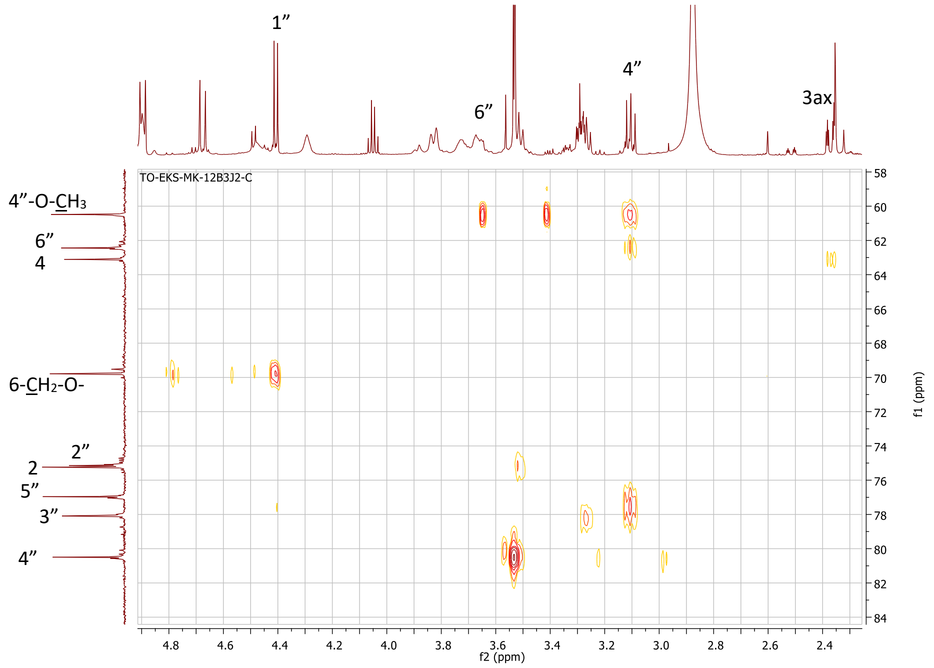

Figure S80. HMBC contour map – ^{1}H x ^{13}C expansion of 8-nitroflavan-4-ol 6-methylene-*O*-*β*-D-(4"-*O*-methyl)-glucopyranoside **(5b)**

Molecular Formula: $C_{16}H_{11}NO_4$
Formula Weight: 281.263
Ionization mode: positive
Precursor $[M + H]^+$: 282.069
Collision energy (CE): -25.0

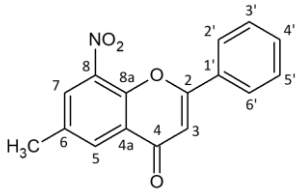

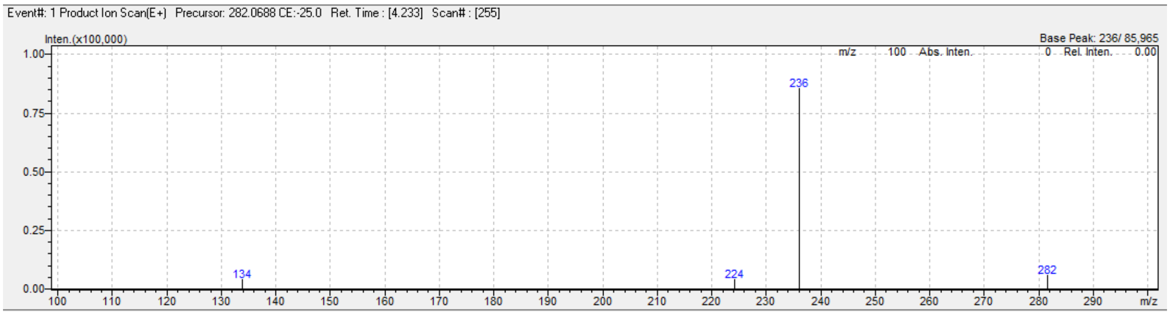

Figure S81. MS analysis of 6-methyl-8-nitroflavone **(6)**

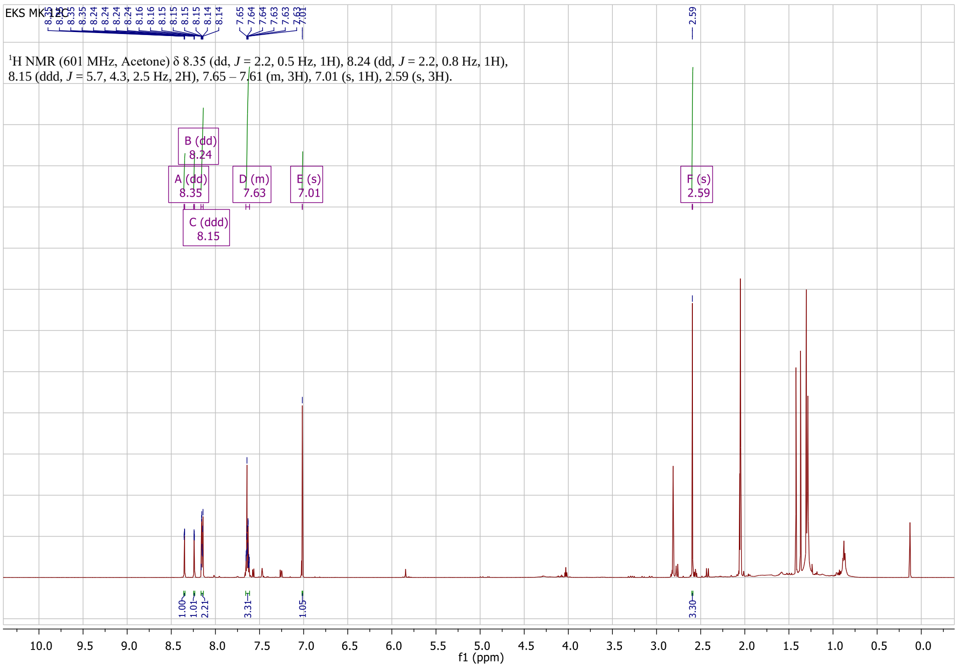

Figure S82. ^{1}H NMR spectrum of 6-methyl-8-nitroflavone **(6)**

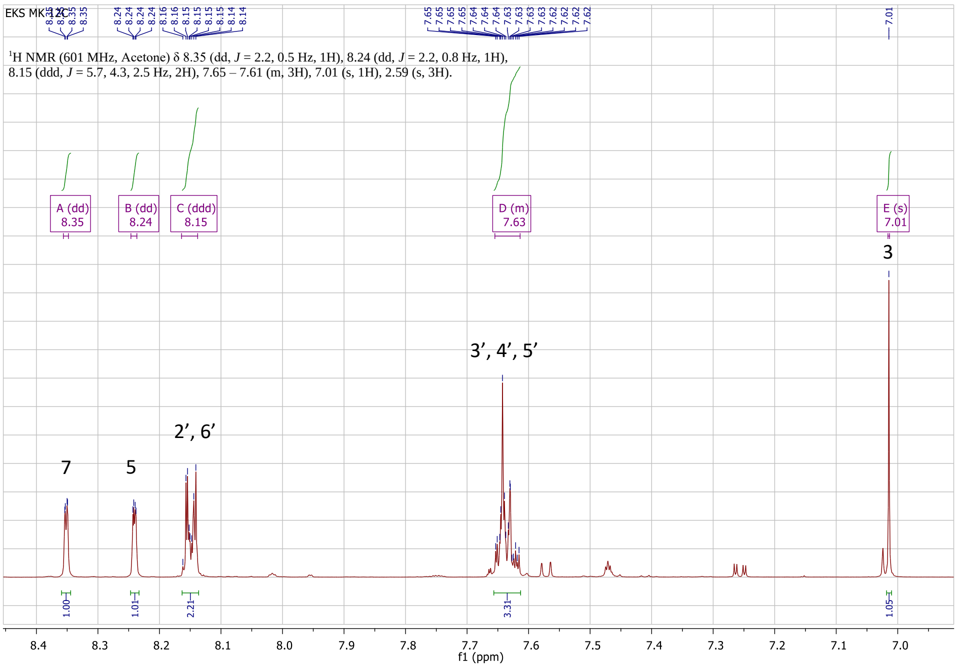

Figure S83. ^{1}H NMR spectrum expansion of 6-methyl-8-nitroflavone **(6)**

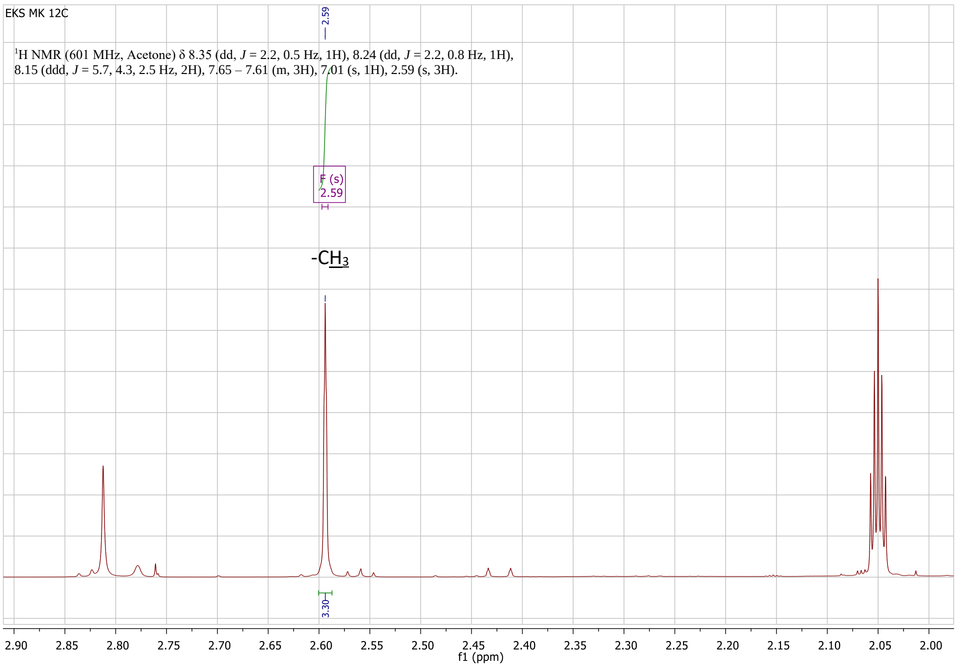

Figure S84. ^{1}H NMR spectrum expansion of 6-methyl-8-nitroflavone **(6)**

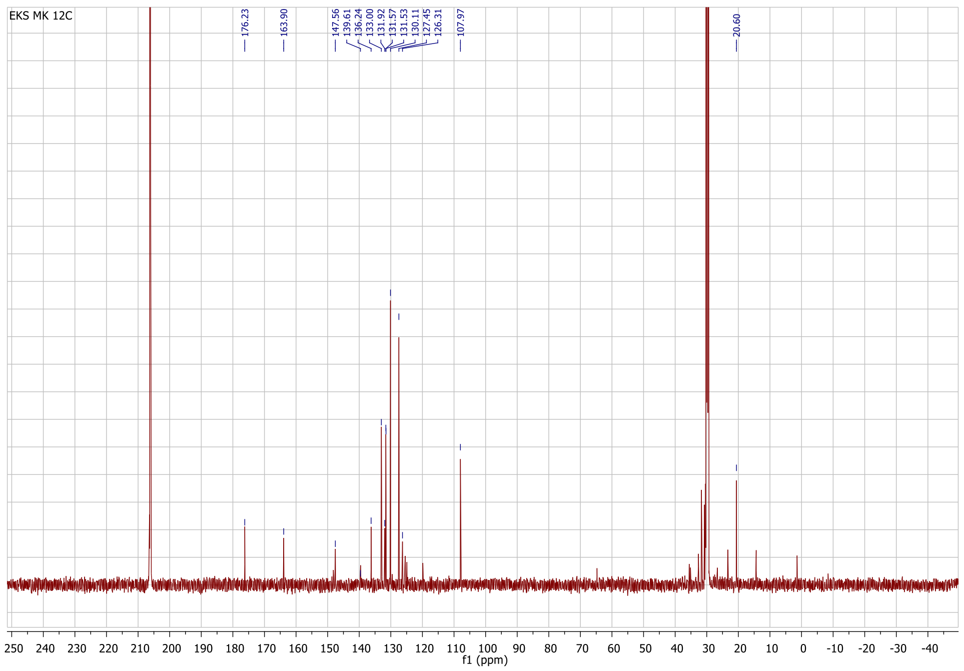

Figure S85. ^{13}C NMR spectrum of 6-methyl-8-nitroflavone **(6)**

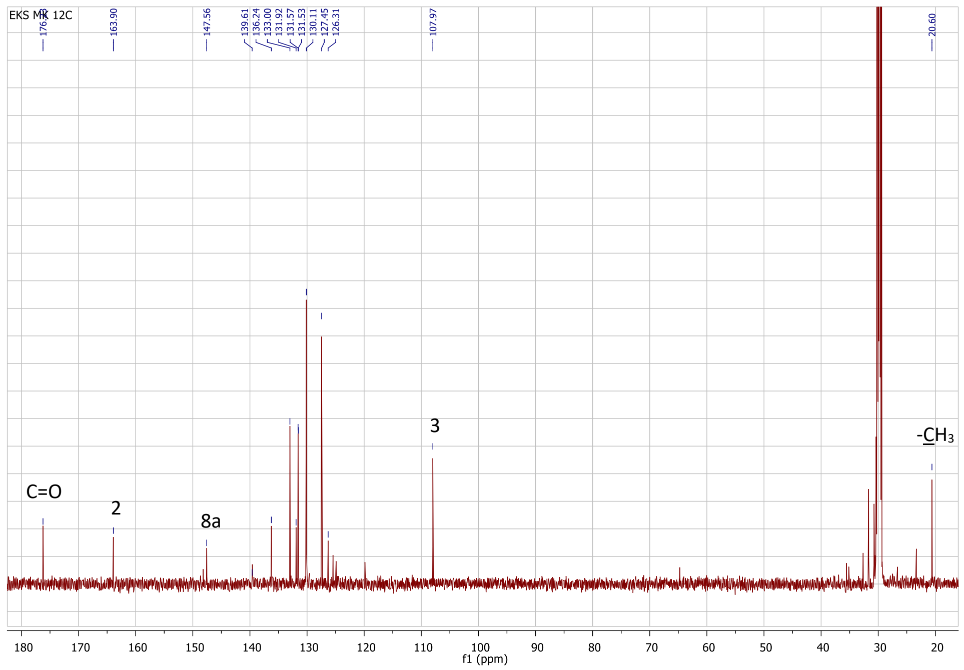

Figure S86. ^{13}C NMR spectrum expansion of 6-methyl-8-nitroflavone **(6)**

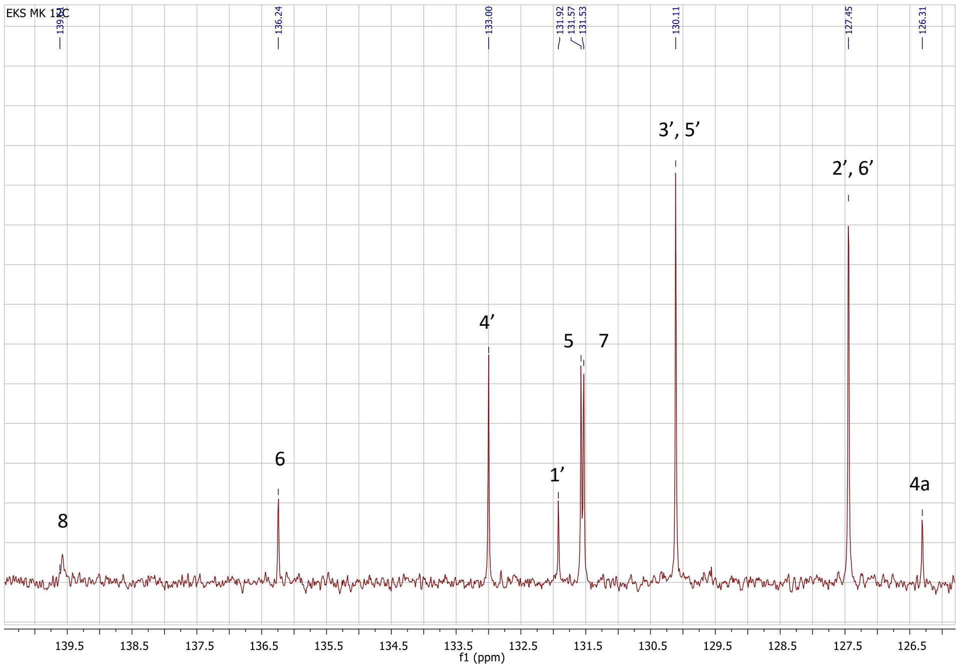

Figure S87. ^{13}C NMR spectrum expansion of 6-methyl-8-nitroflavone **(6)**

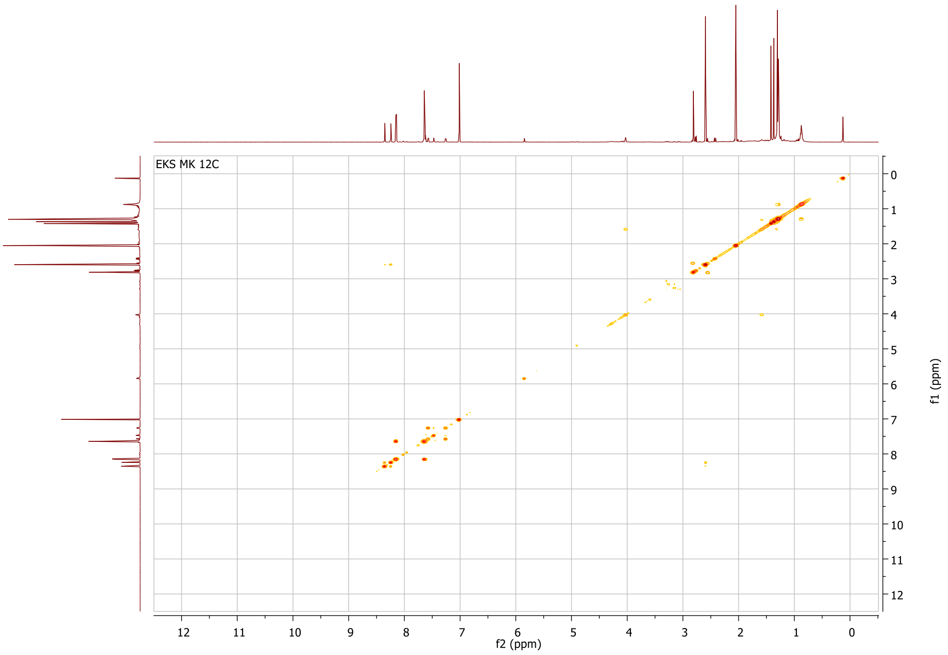

Figure S88. COSY contour map – ^{1}H x ^{1}H of 6-methyl-8-nitroflavone **(6)**

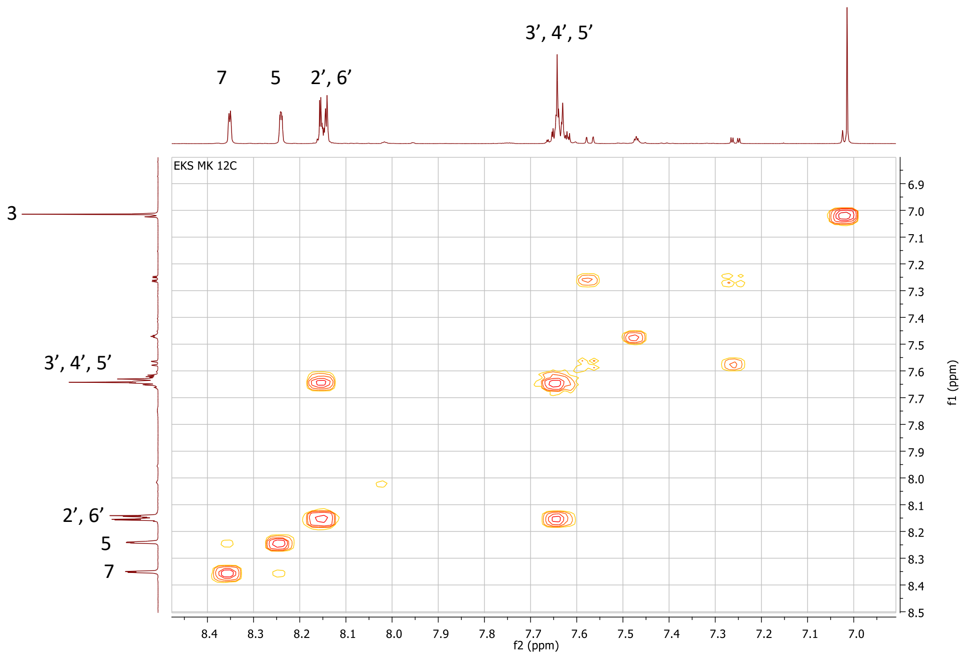

Figure S89. COSY contour map – ^{1}H x ^{1}H expansion of 6-methyl-8-nitroflavone **(6)**

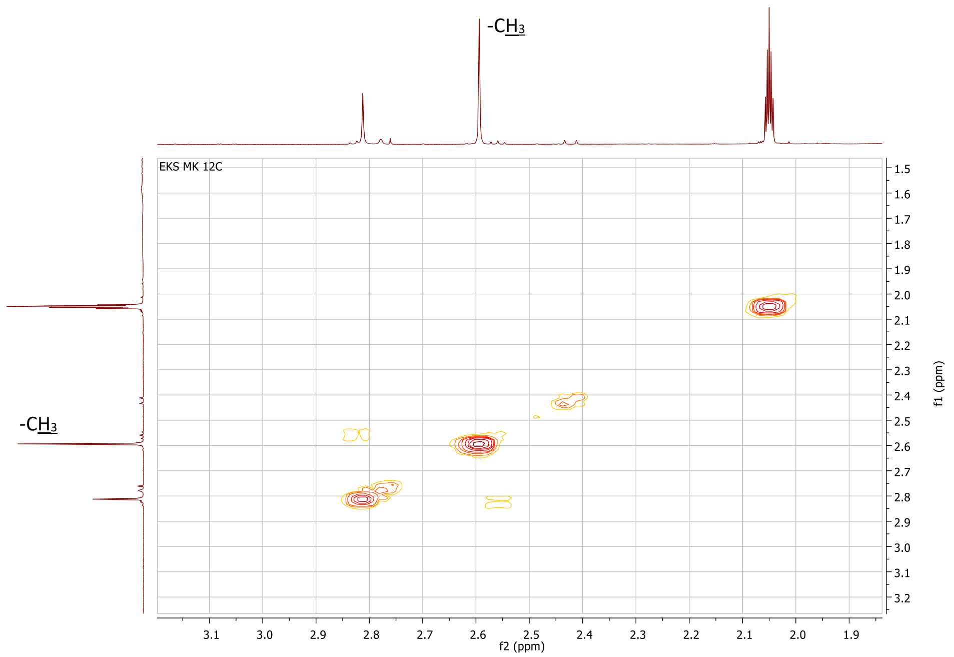

Figure S90. COSY contour map – ^{1}H x ^{1}H expansion of 6-methyl-8-nitroflavone **(6)**

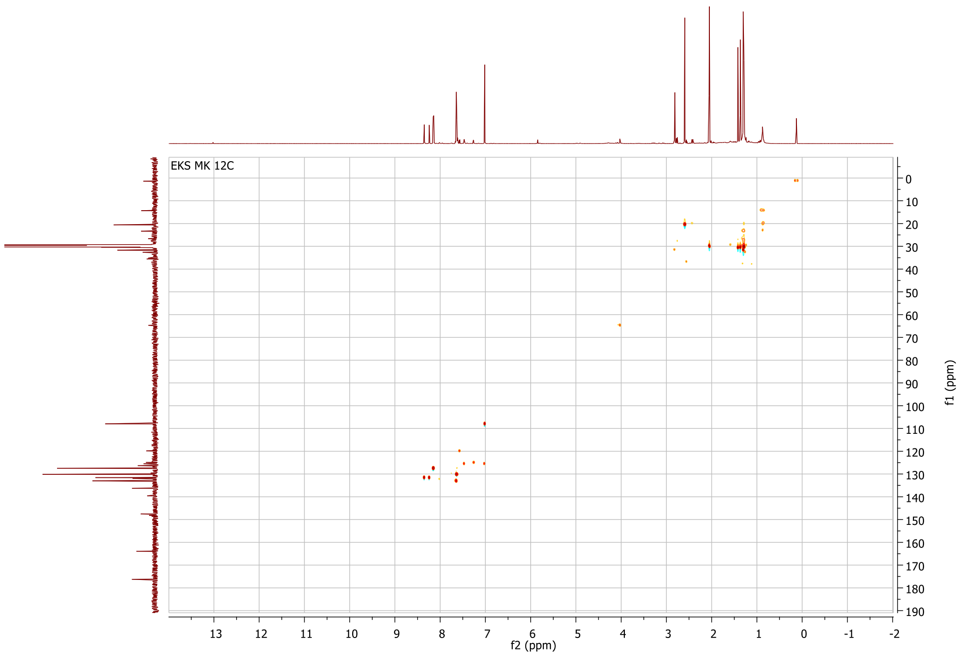

Figure S91. HSQC contour map – ^{1}H x ^{13}C of 6-methyl-8-nitroflavone **(6)**

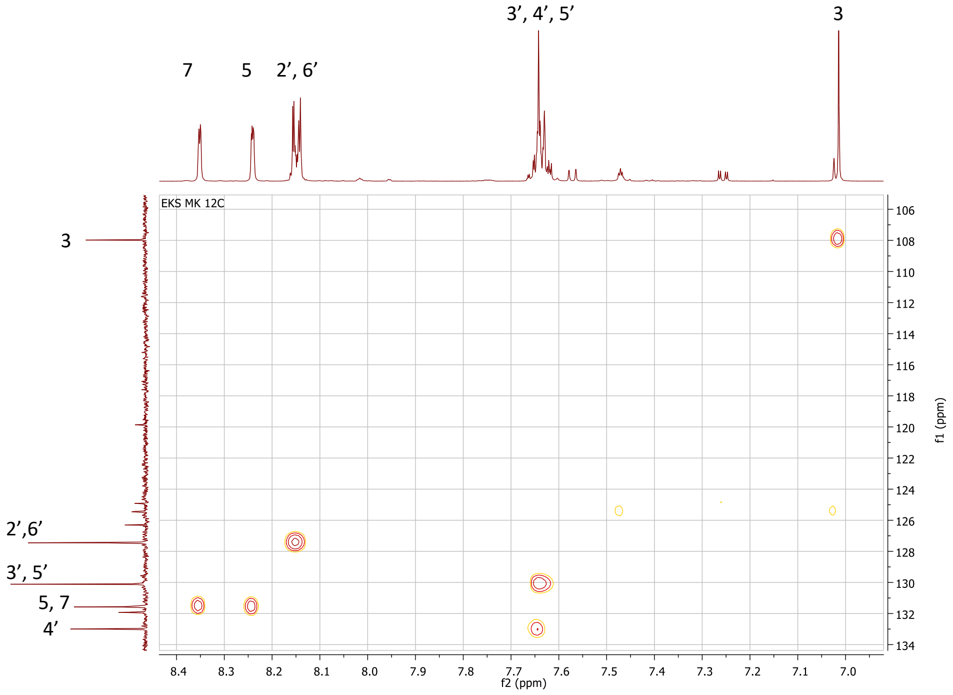

Figure S92. HSQC contour map – ^{1}H x ^{13}C expansion of 6-methyl-8-nitroflavone **(6)**

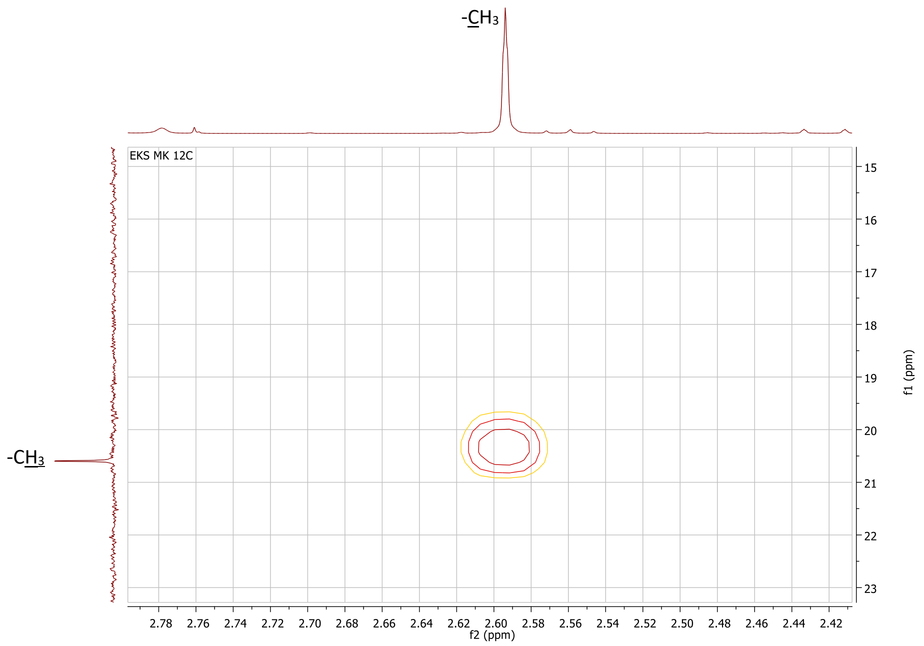

Figure S93. HSQC contour map – ^{1}H x ^{13}C expansion of 6-methyl-8-nitroflavone **(6)**

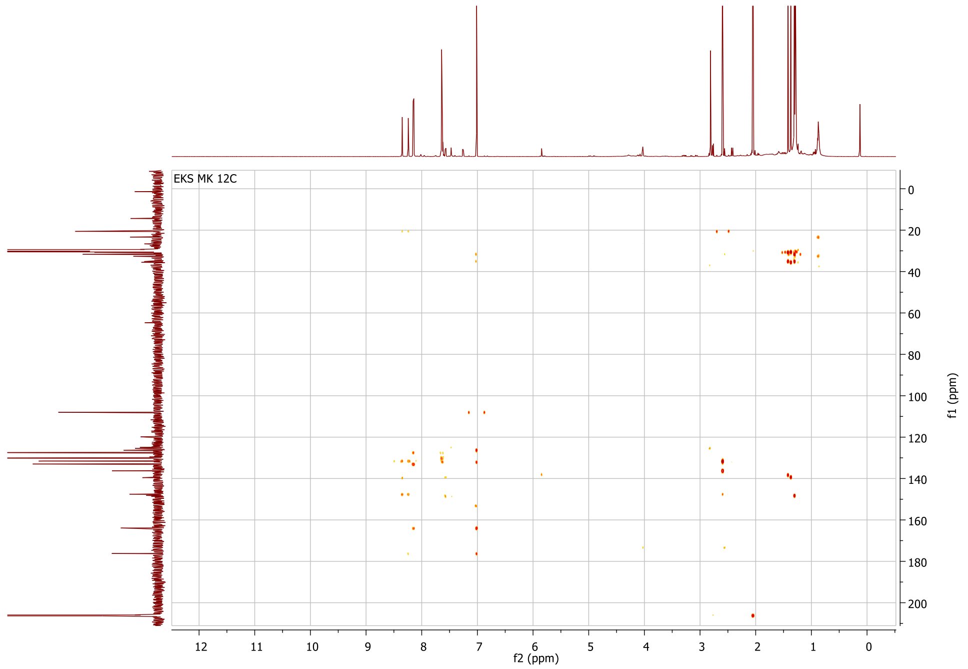

Figure S94. HMBC contour map – ^{1}H x ^{13}C of 6-methyl-8-nitroflavone **(6)**

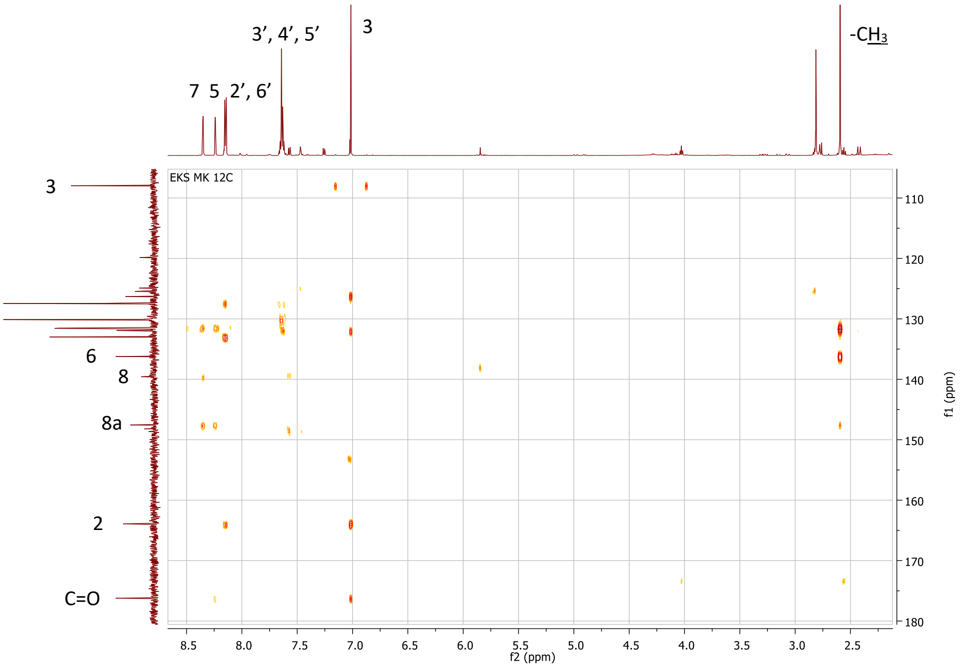

Figure S95. HMBC contour map – ^{1}H x ^{13}C expansion of 6-methyl-8-nitroflavone **(6)**

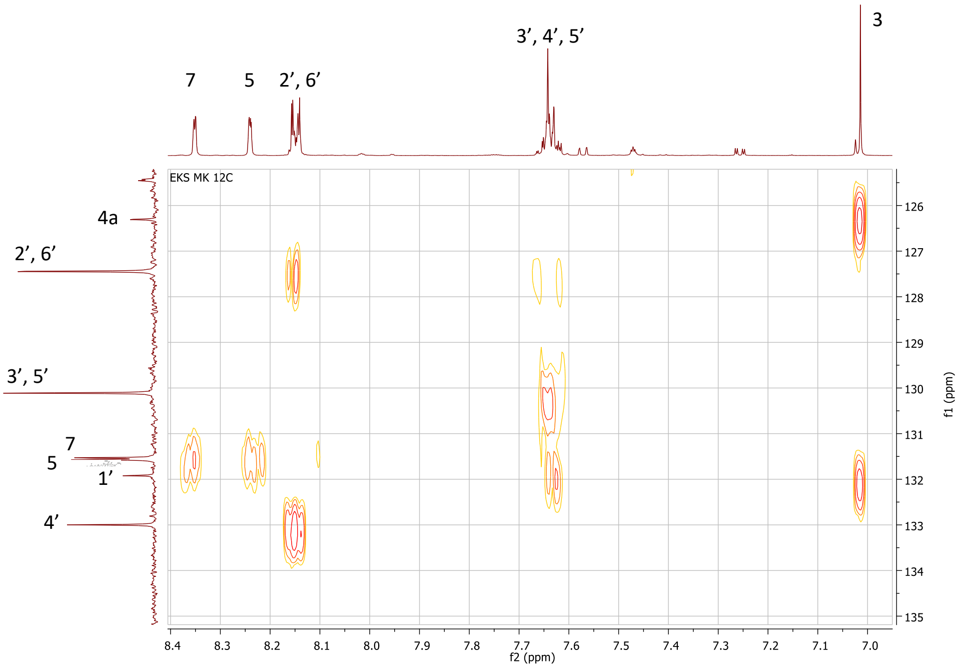

Figure S96. HMBC contour map – ^{1}H x ^{13}C expansion of 6-methyl-8-nitroflavone **(6)**

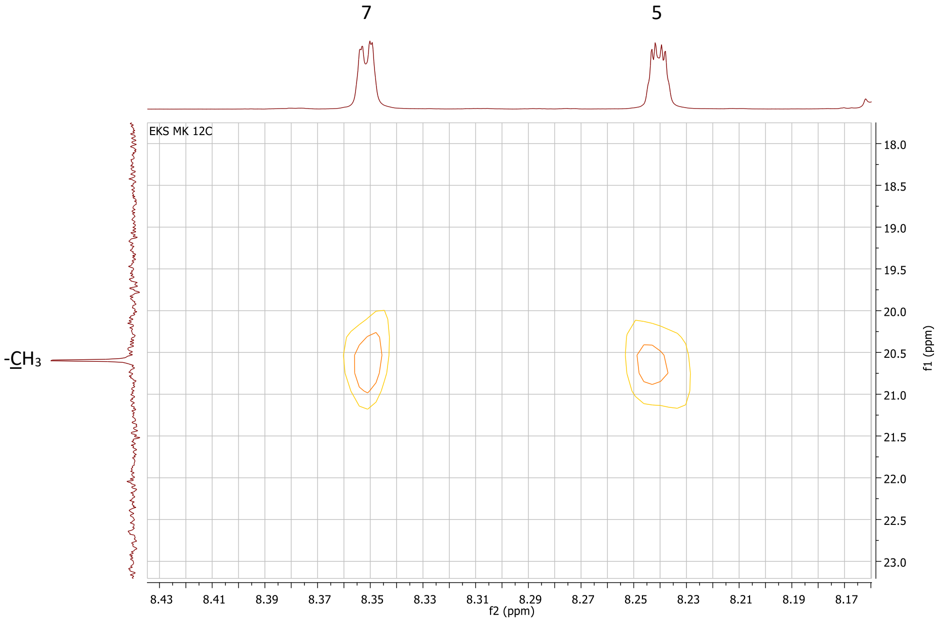

Figure S97. HMBC contour map – ^{1}H x ^{13}C expansion of 6-methyl-8-nitroflavone **(6)**

Molecular Formula: $C_{23}H_{23}NO_{10}$
Formula Weight: 473.429
Ionization mode: positive
Precursor $[M + H]^{+}$: 474.132
Collision energy (CE): -15.0

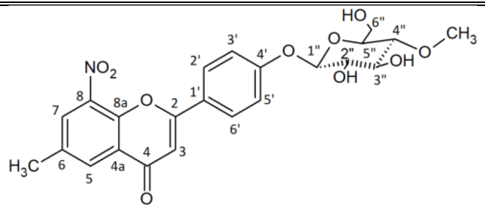

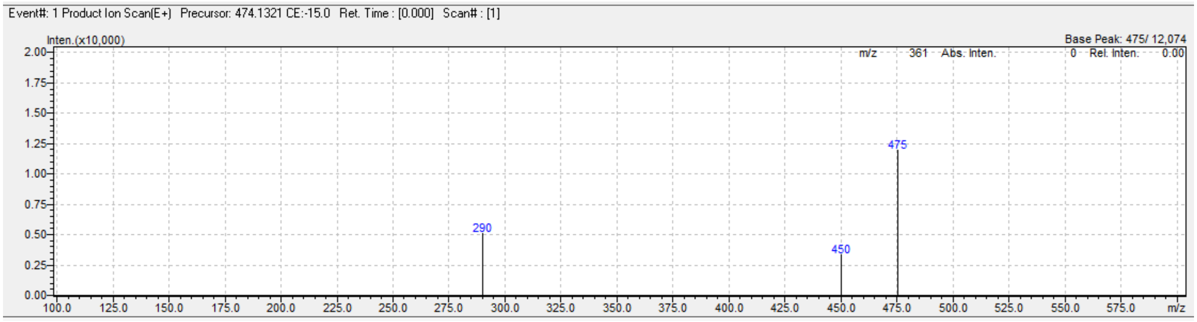

Figure S98. MS analysis of 6-methyl-8-nitroflavone 4’-*O*-*β*-D-(4”-*O*-methyl)-glucopyranoside **(6a)**

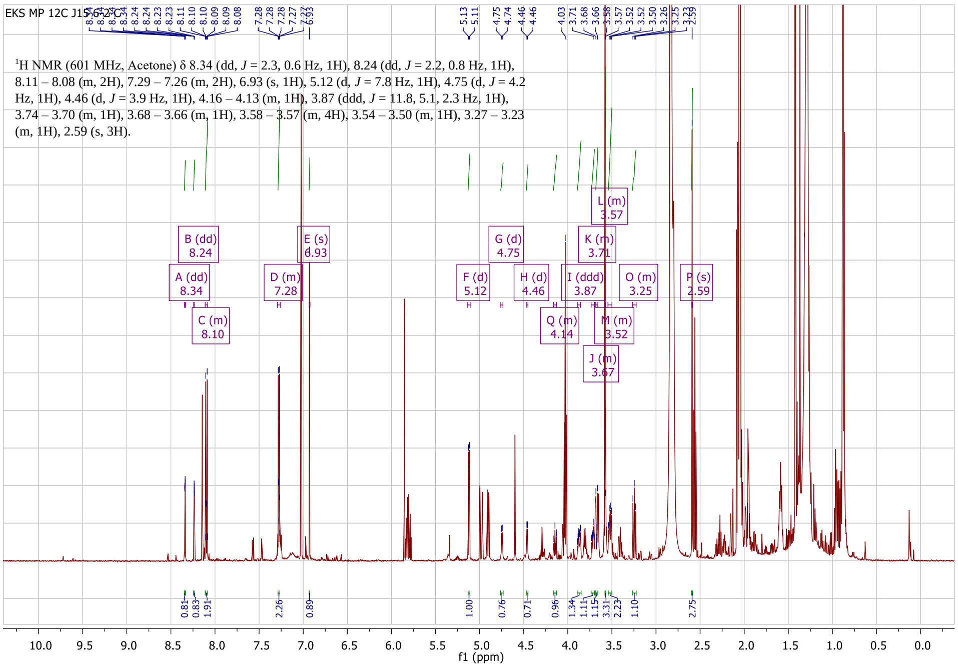

Figure S99. ^{1}H NMR spectrum of 6-methyl-8-nitroflavone 4'-*O*-*β*-D-(4''-*O*-methyl)-glucopyranoside **(6a)**

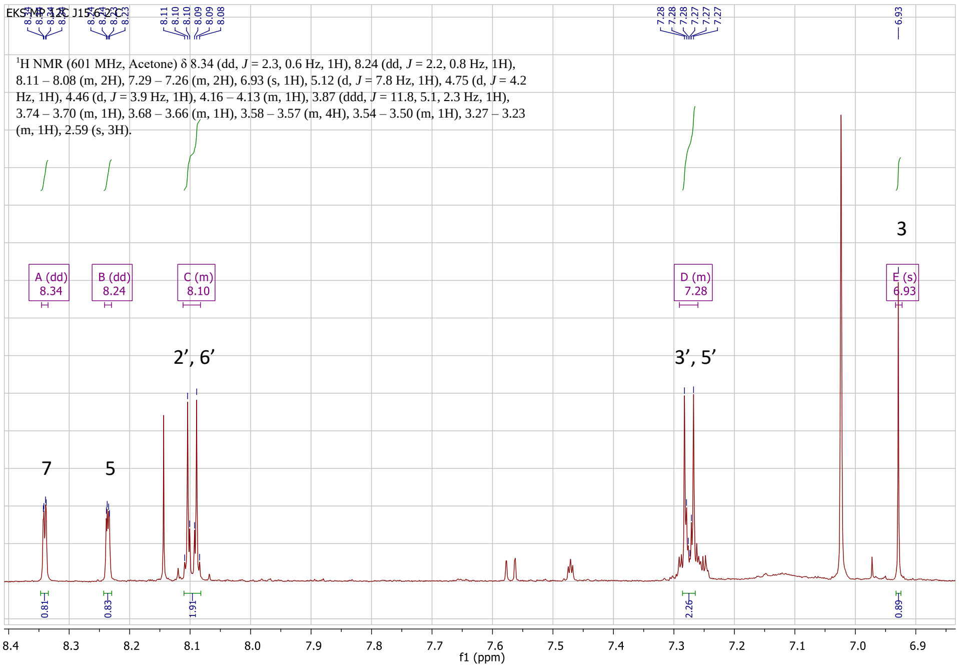

Figure S100. ^{1}H NMR spectrum expansion of 6-methyl-8-nitroflavone 4'-*O*-*β*-D-(4''-*O*-methyl)-glucopyranoside **(6a)**

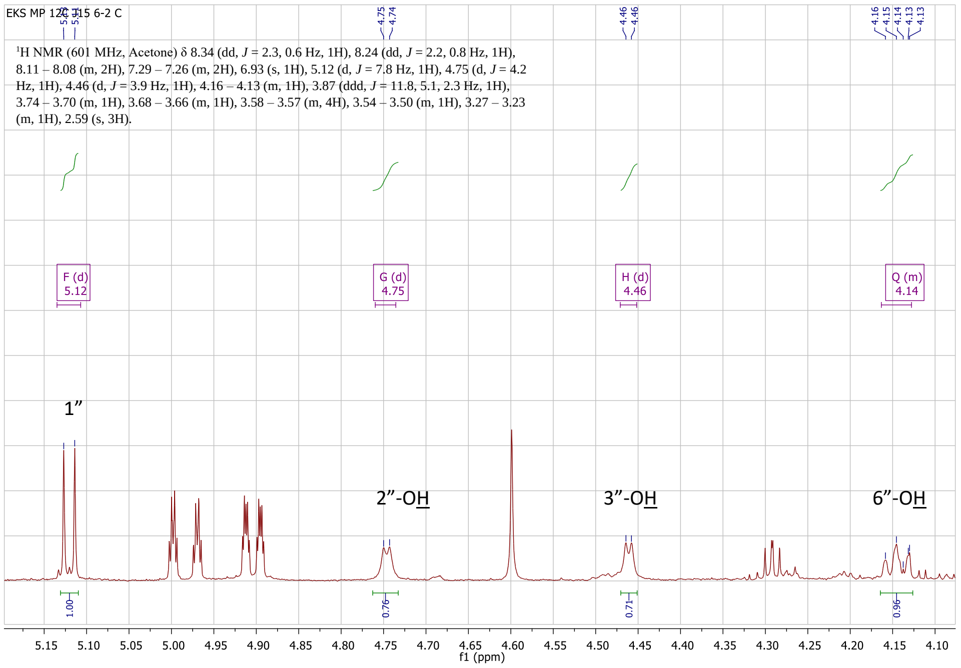

Figure S101. ^{1}H NMR spectrum expansion of 6-methyl-8-nitroflavone 4’-*O*-*β*-D-(4”-*O*-methyl)-glucopyranoside **(6a)**

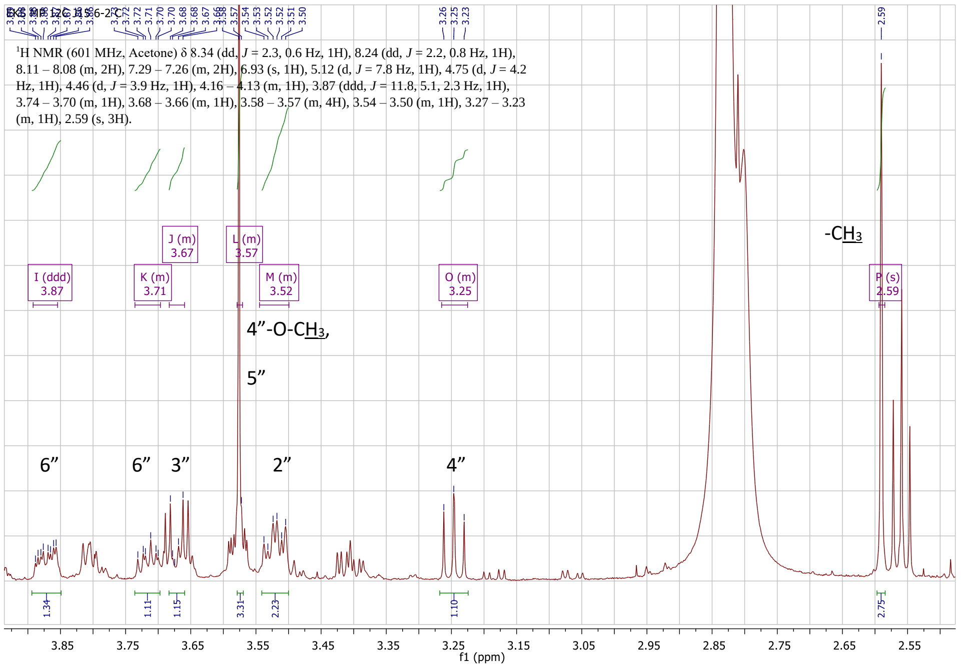

Figure S102. ^{1}H NMR spectrum expansion of 6-methyl-8-nitroflavone 4’-*O*-*β*-D-(4”-*O*-methyl)-glucopyranoside **(6a)**

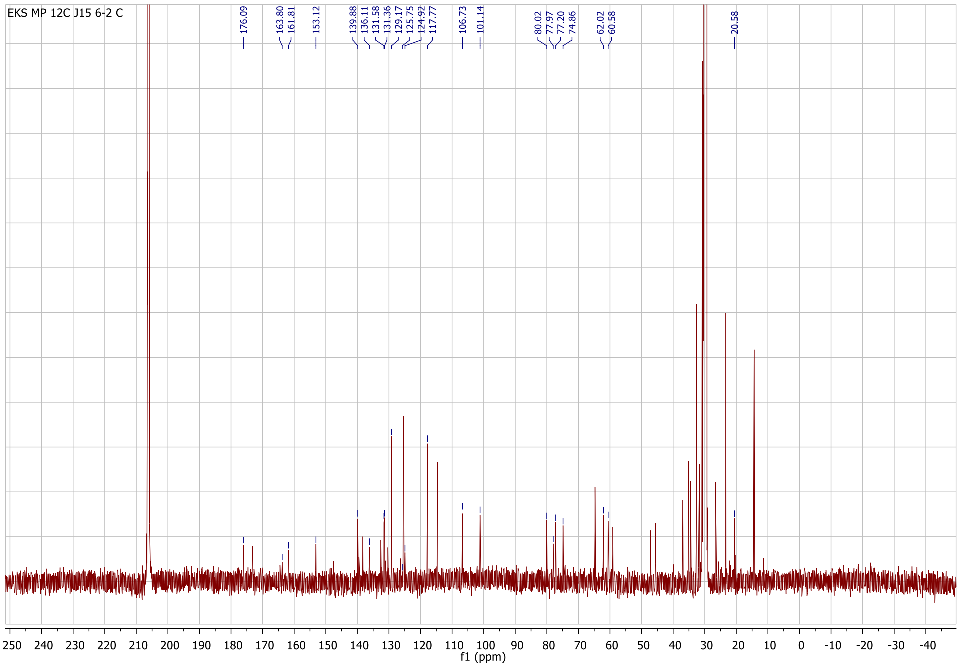

Figure S103. ^{13}C NMR spectrum of 6-methyl-8-nitroflavone 4′-*O*-*β*-D-(4″-*O*-methyl)-glucopyranoside **(6a)**

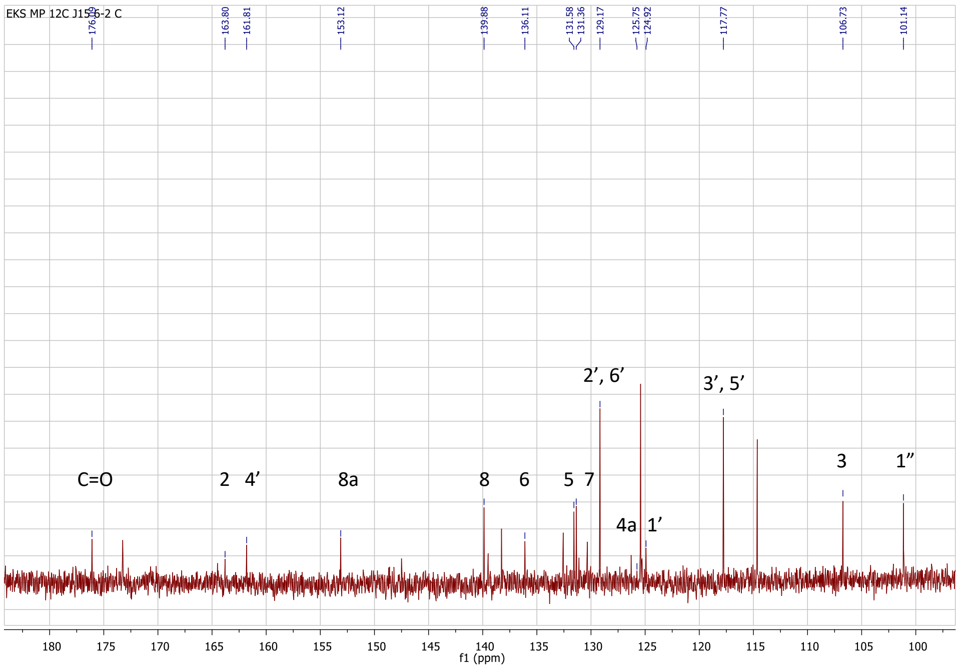

Figure S104. ^{13}C NMR spectrum expansion of 6-methyl-8-nitroflavone 4′-*O*-*β*-D-(4″-*O*-methyl)-glucopyranoside **(6a)**

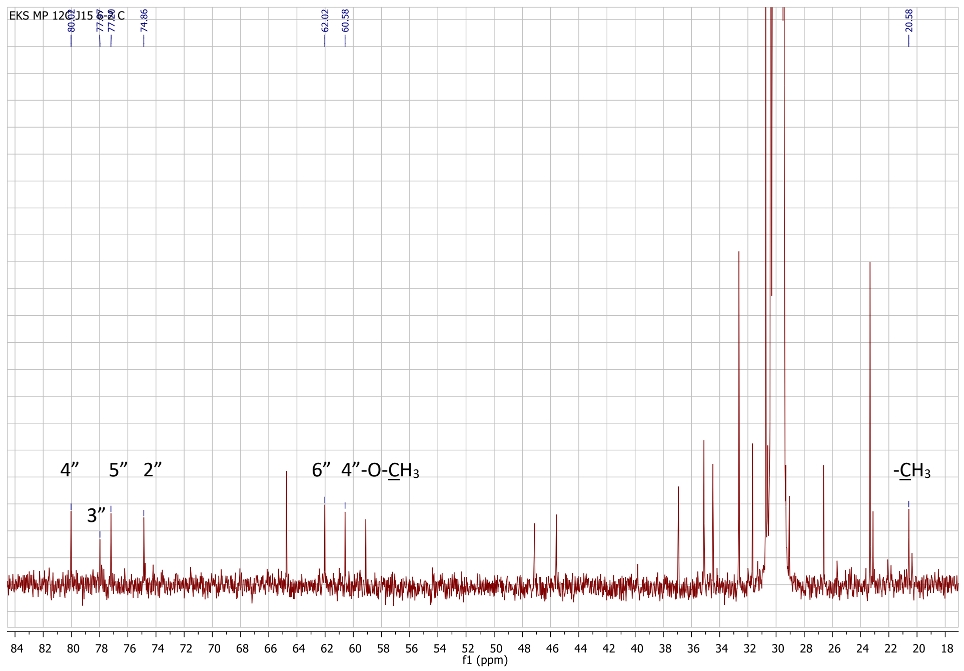

Figure S105. ^{13}C NMR spectrum expansion of 6-methyl-8-nitroflavone 4’-*O*-*β*-D-(4”-*O*-methyl)-glucopyranoside **(6a)**

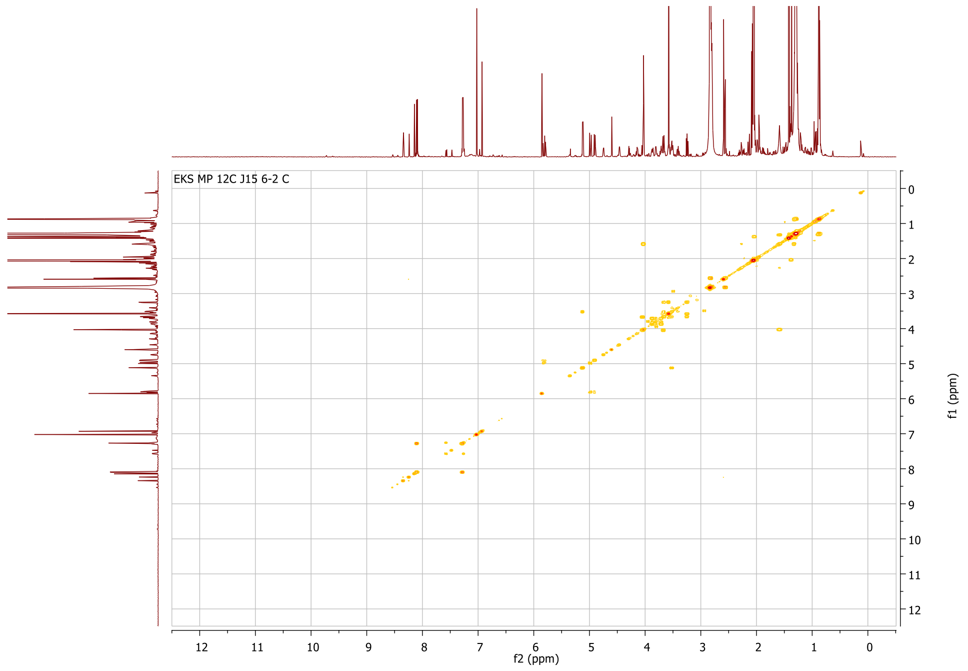

Figure S106. COSY contour map – ^{1}H x ^{1}H of 6-methyl-8-nitroflavone 4’-*O*-*β*-D-(4”-*O*-methyl)-glucopyranoside **(6a)**

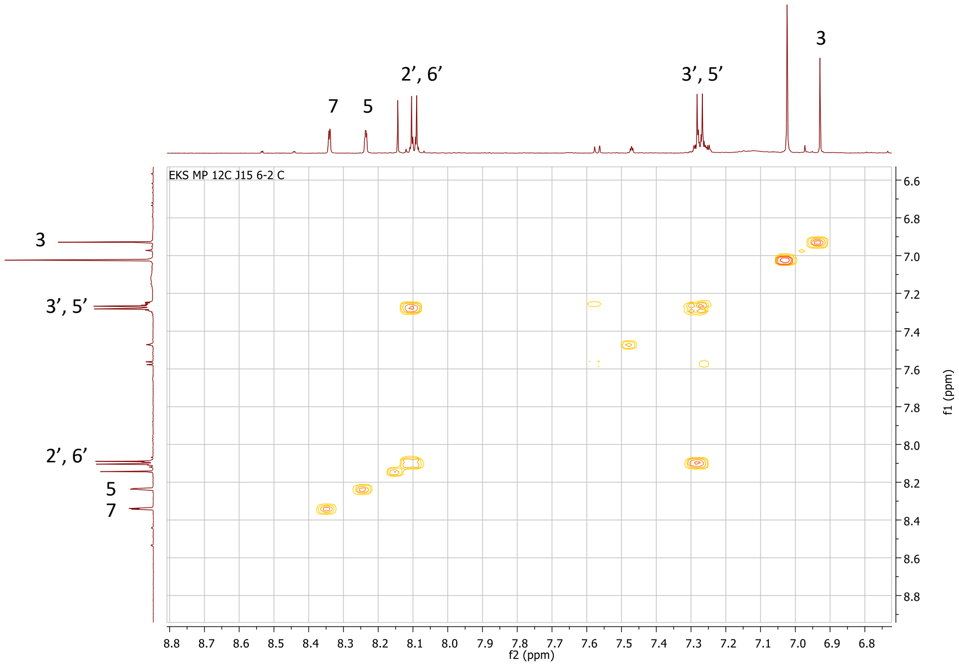

Figure S107. COSY contour map – ^{1}H x ^{1}H expansion of 6-methyl-8-nitroflavone 4’-*O*-*β*-D-(4”-*O*-methyl)-glucopyranoside **(6a)**

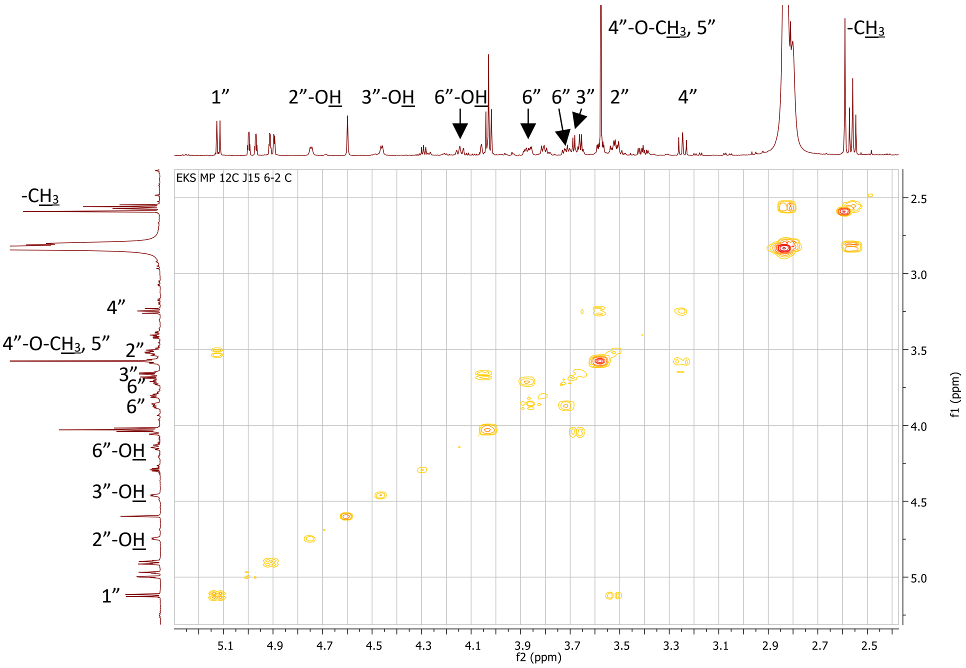

Figure S108. COSY contour map – ^{1}H x ^{1}H expansion of 6-methyl-8-nitroflavone 4’-*O*-*β*-D-(4”-*O*-methyl)-glucopyranoside **(6a)**

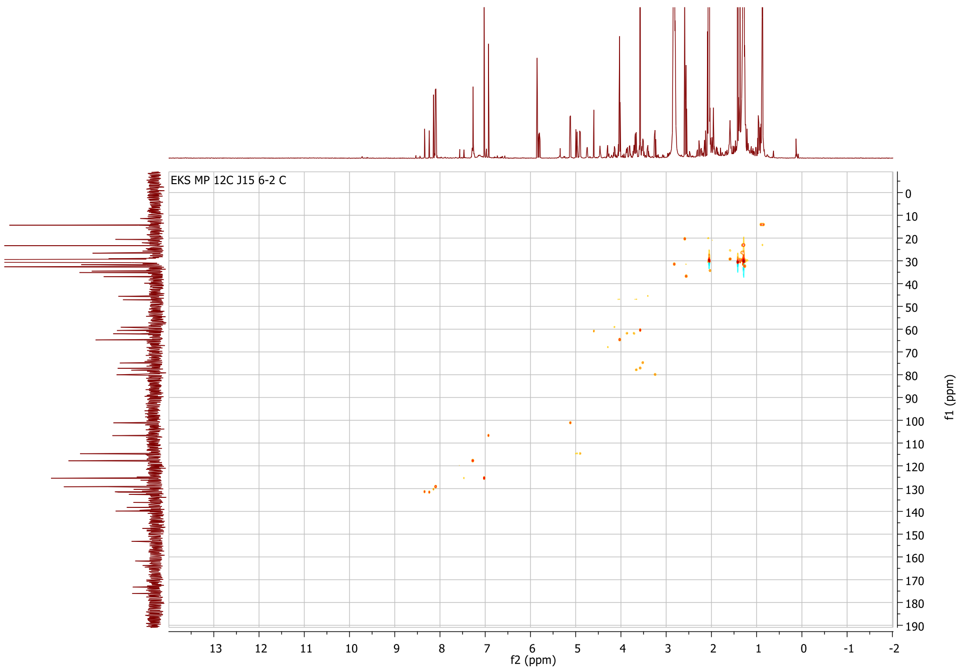

Figure S109. HSQC contour map – ^{1}H x ^{13}C of 6-methyl-8-nitroflavone 4’-*O*-*β*-D-(4”-*O*-methyl)-glucopyranoside **(6a)**

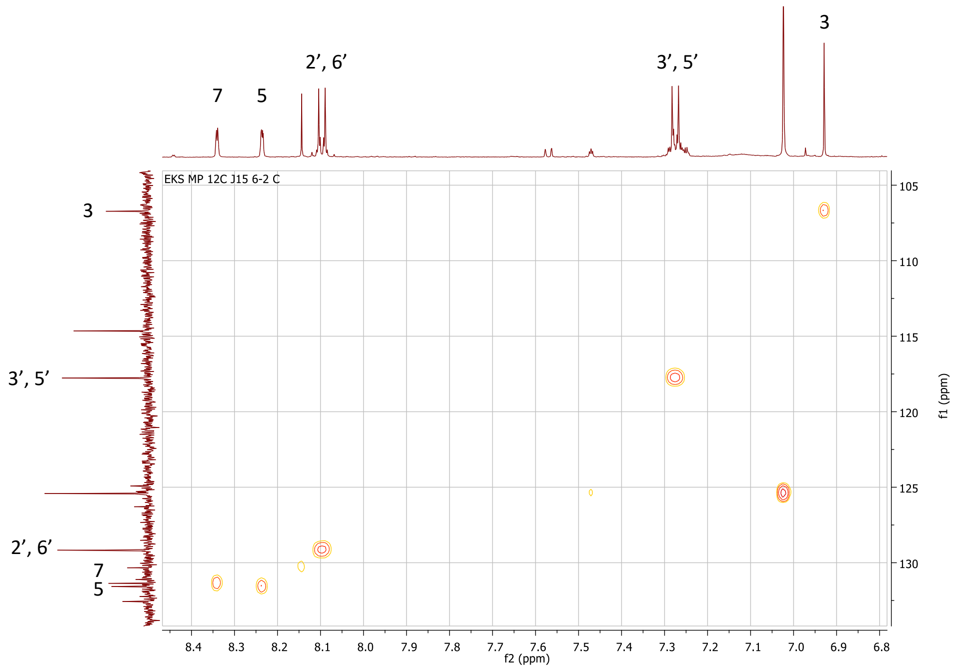

Figure S110. HSQC contour map – ^{1}H x ^{13}C expansion of 6-methyl-8-nitroflavone 4’-*O*-*β*-D-(4”-*O*-methyl)-glucopyranoside **(6a)**

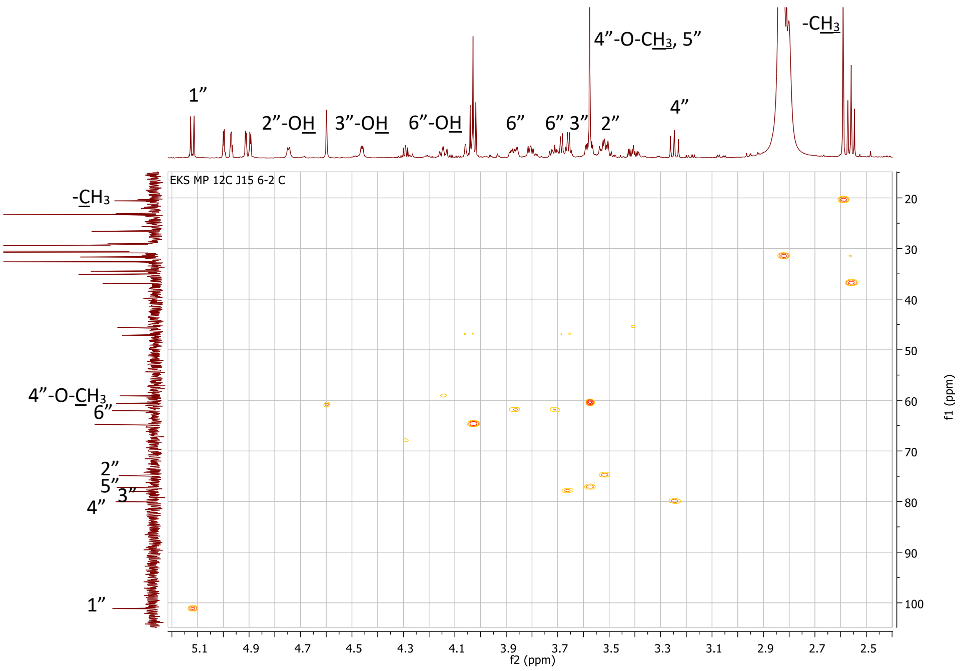

Figure S111. HSQC contour map – ^{1}H x ^{13}C expansion of 6-methyl-8-nitroflavone 4’-*O*-*β*-D-(4”-*O*-methyl)-glucopyranoside **(6a)**

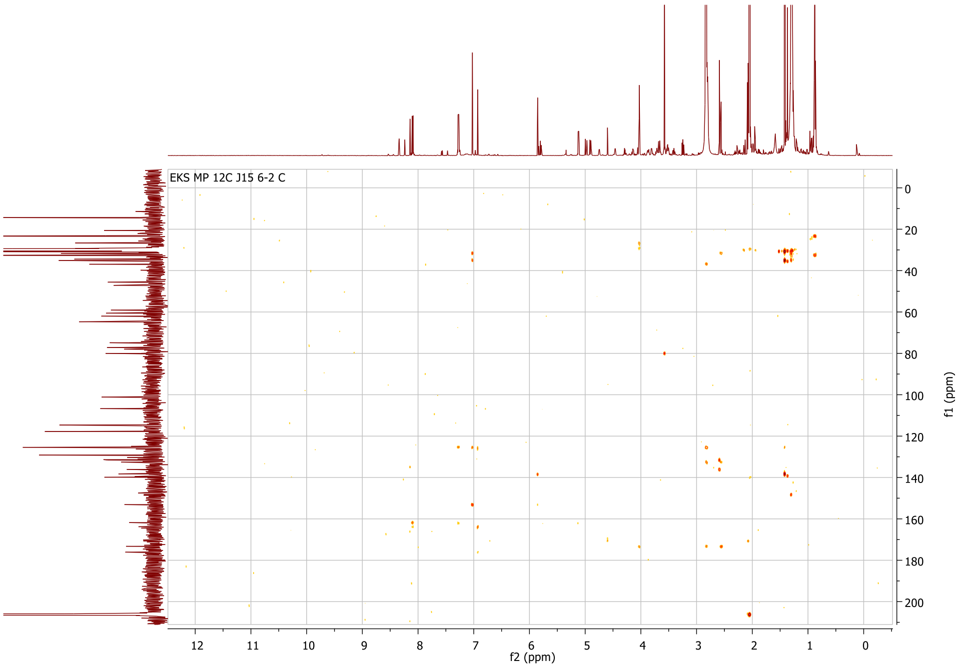

Figure S112. HMBC contour map – ^{1}H x ^{13}C of 6-methyl-8-nitroflavone 4’-*O*-*β*-D-(4”-*O*-methyl)-glucopyranoside **(6a)**

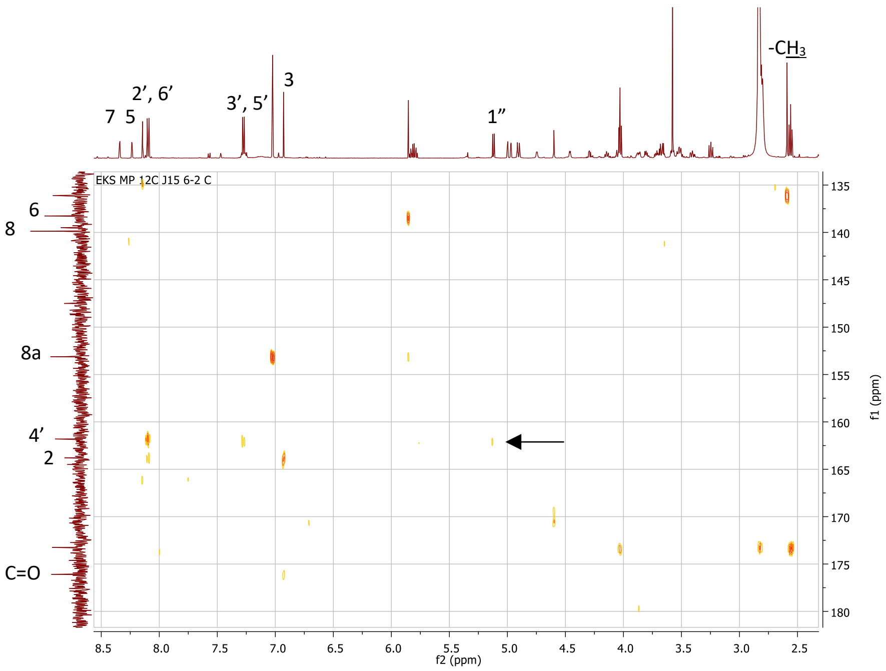

Figure S113. HMBC contour map – ^{1}H x ^{13}C expansion of 6-methyl-8-nitroflavone 4'-*O*-*β*-D-(4"-*O*-methyl)-glucopyranoside **(6a)**

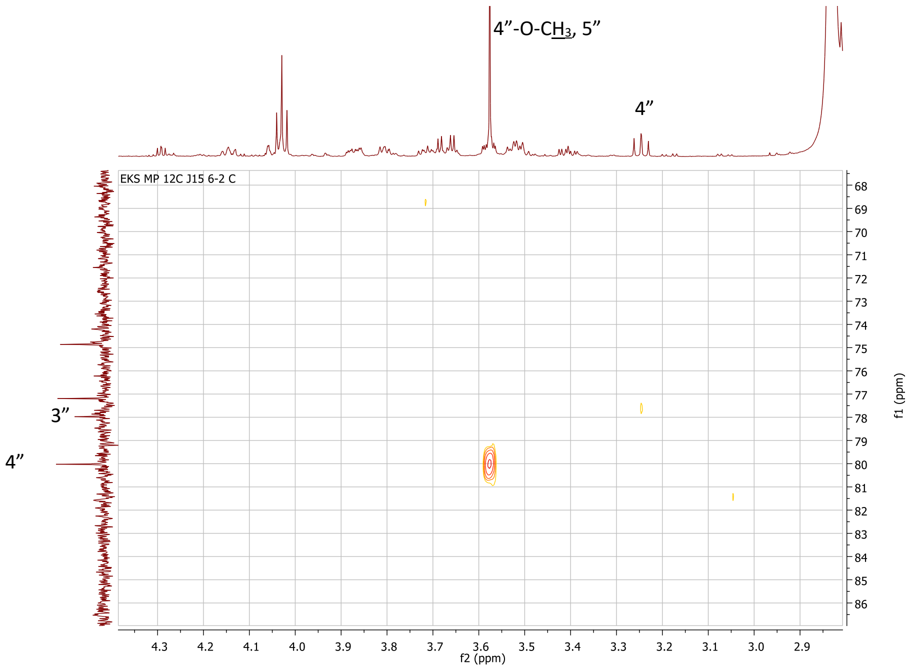

Figure S114. HMBC contour map – ^{1}H x ^{13}C expansion of 6-methyl-8-nitroflavone 4'-*O*-*β*-D-(4"-*O*-methyl)-glucopyranoside **(6a)**

Molecular Formula: $C_{15}H_{10}BrClO_2$
Formula Weight: 337.596
Ionization mode: positive
Precursor $[M + H]^+$: 336.955
Collision energy (CE): -15.0

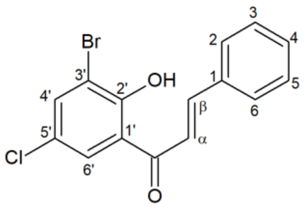

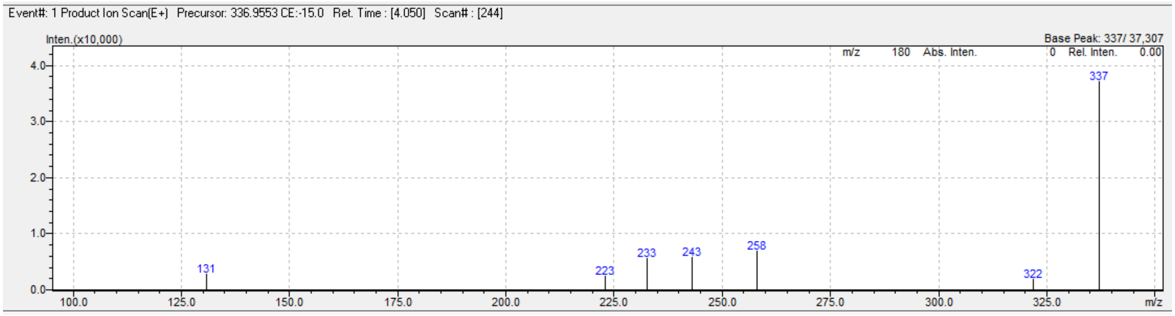

Figure S115. MS analysis of 3'-bromo-5'-chloro-2'-hydroxychalcone **(7)**

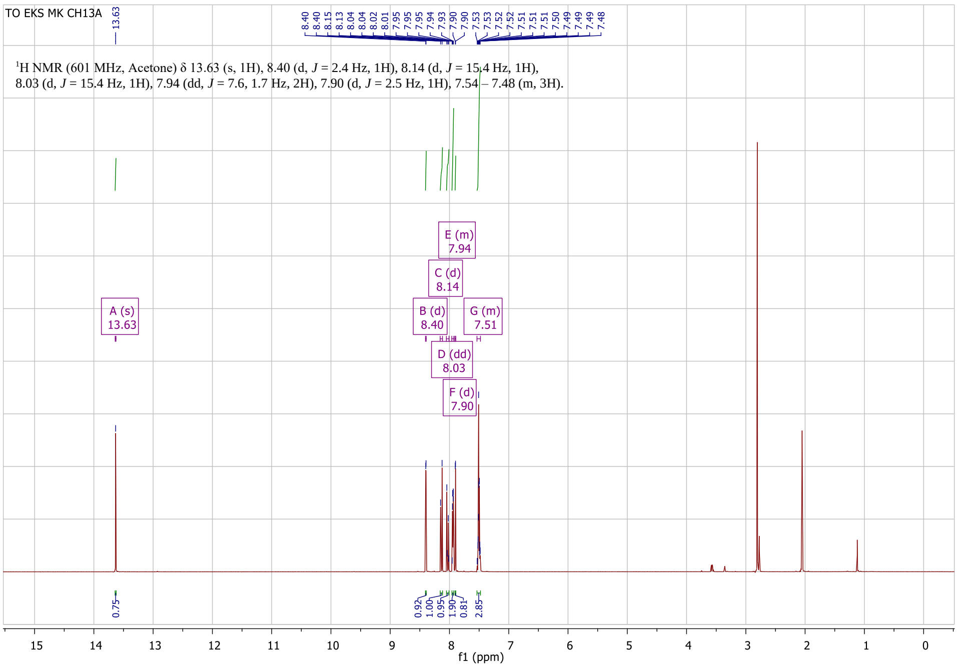

Figure S116. ^{1}H NMR spectrum of 3'-bromo-5'-chloro-2'-hydroxychalcone **(7)**

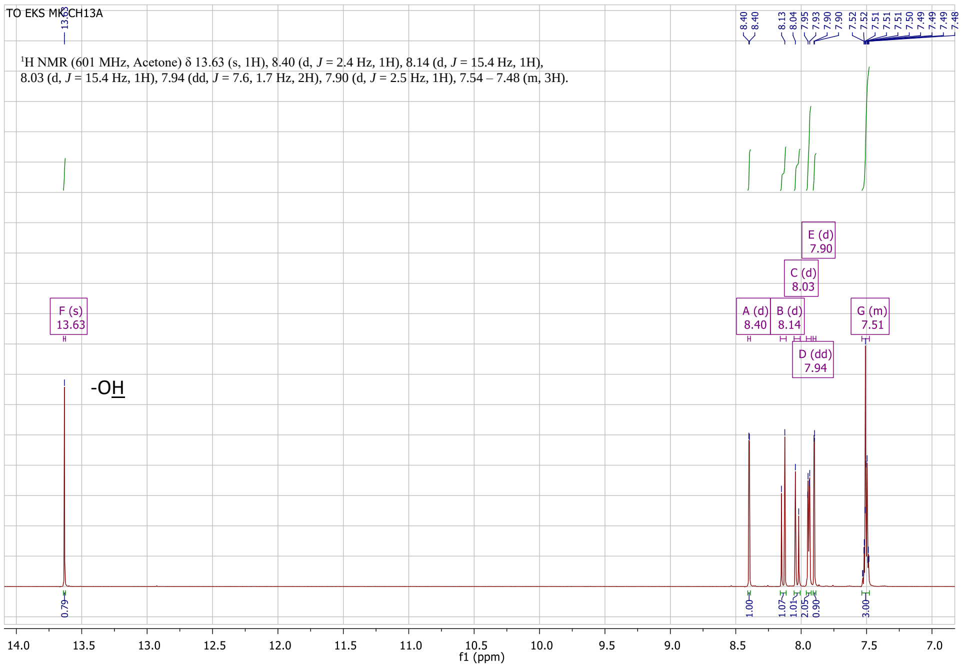

Figure S117. ^{1}H NMR spectrum expansion of 3’-bromo-5’-chloro-2’-hydroxychalcone **(7)**

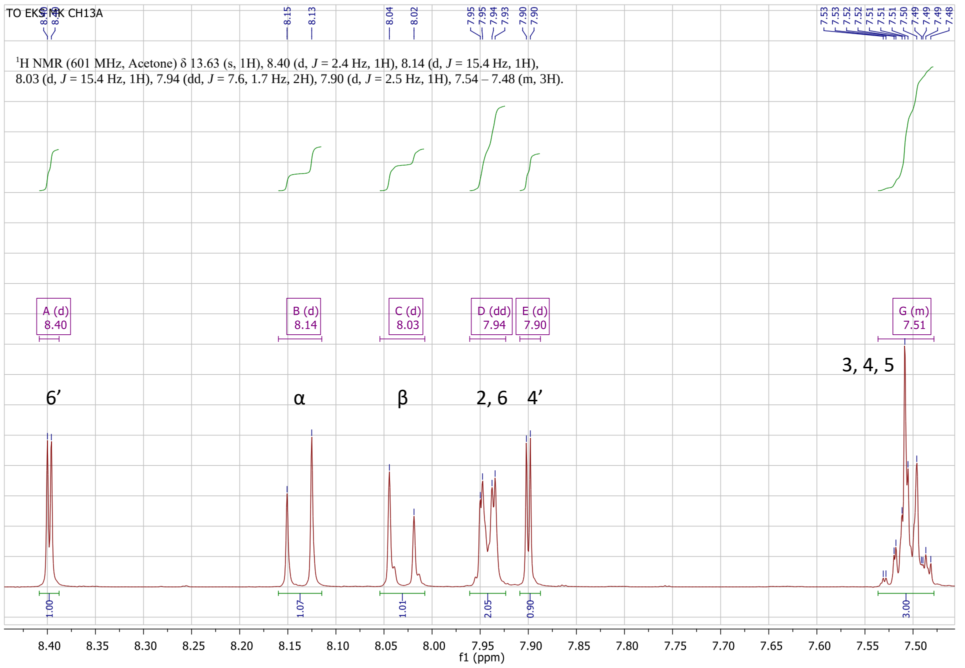

Figure S118. ^{1}H NMR spectrum expansion of 3’-bromo-5’-chloro-2’-hydroxychalcone **(7)**

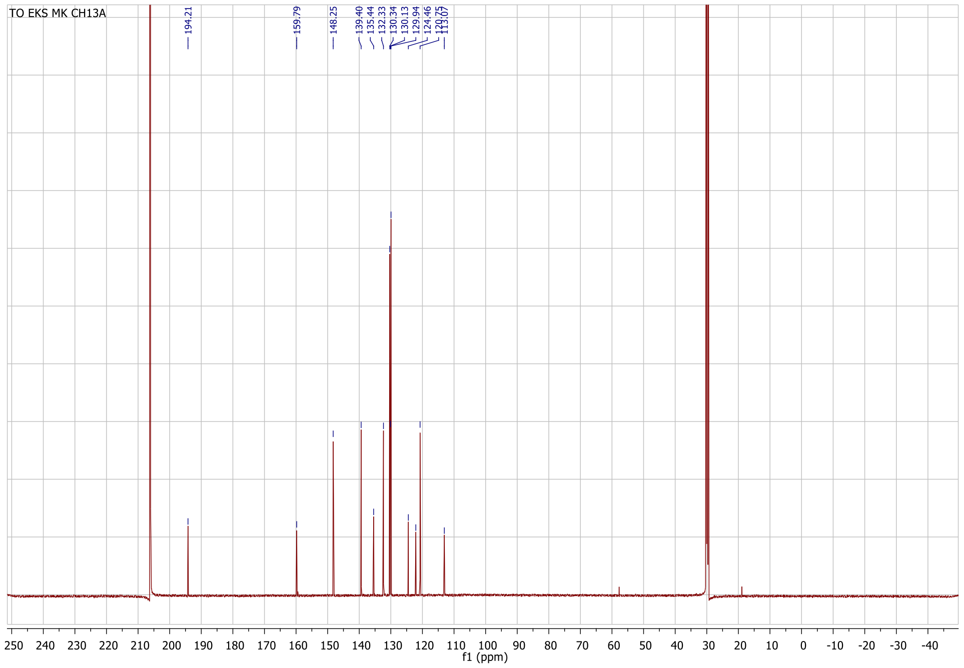

Figure S119. ^{13}C NMR spectrum of 3'-bromo-5'-chloro-2'-hydroxychalcone **(7)**

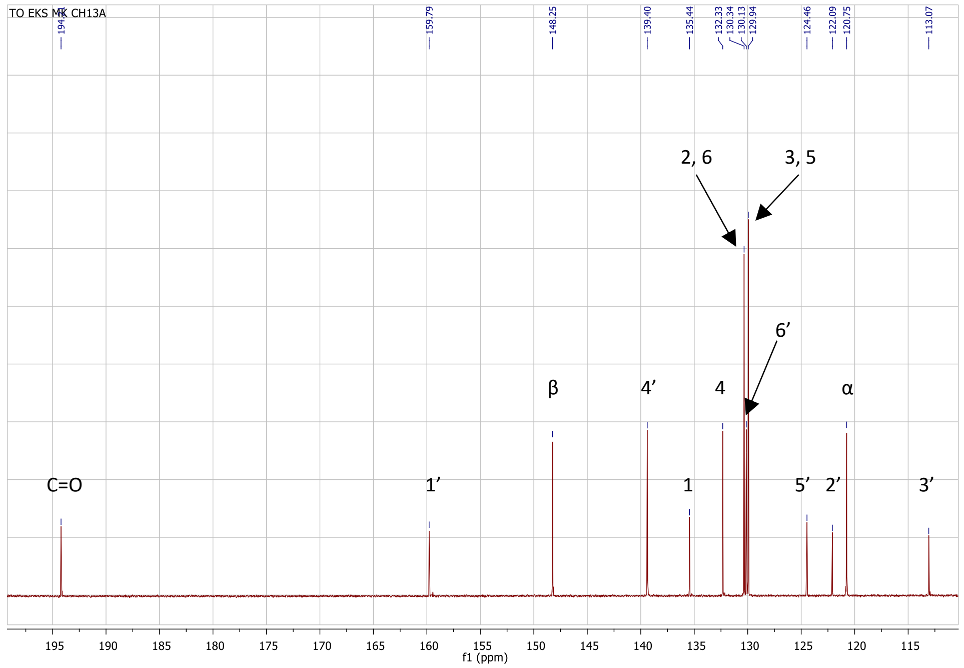

Figure S120. ^{13}C NMR spectrum expansion of 3'-bromo-5'-chloro-2'-hydroxychalcone **(7)**

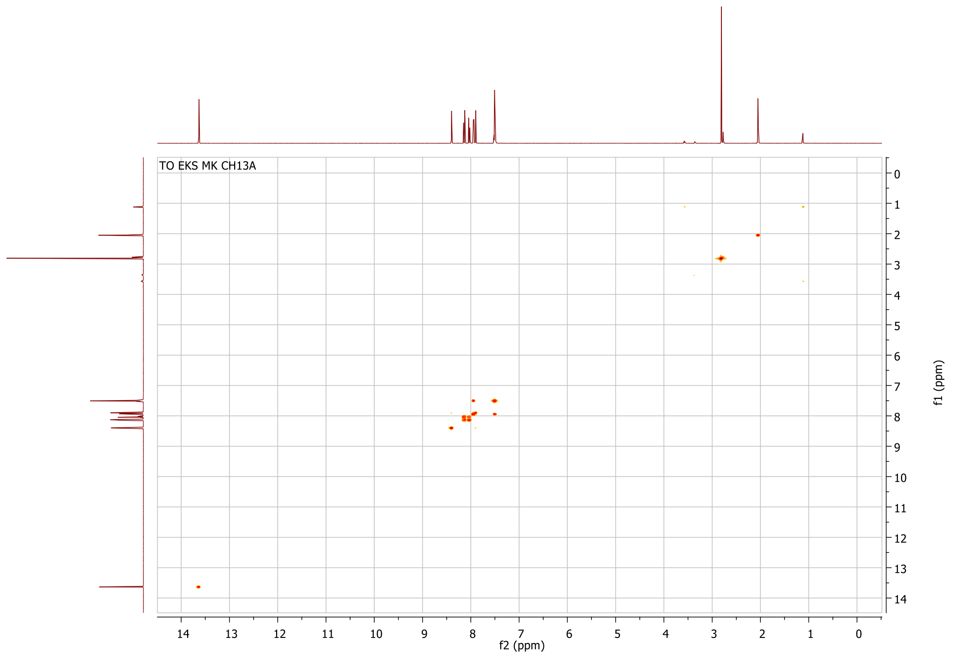

Figure S121. COSY contour map – ^1H x ^1H of 3'-bromo-5'-chloro-2'-hydroxychalcone **(7)**

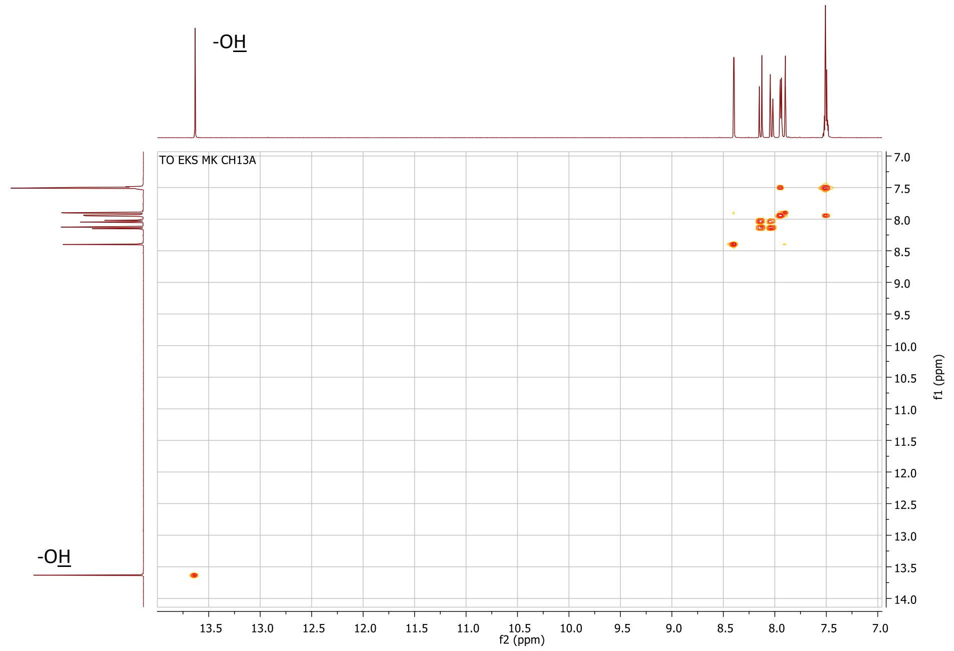

Figure S122. COSY contour map – ^1H x ^1H expansion of 3'-bromo-5'-chloro-2'-hydroxychalcone **(7)**

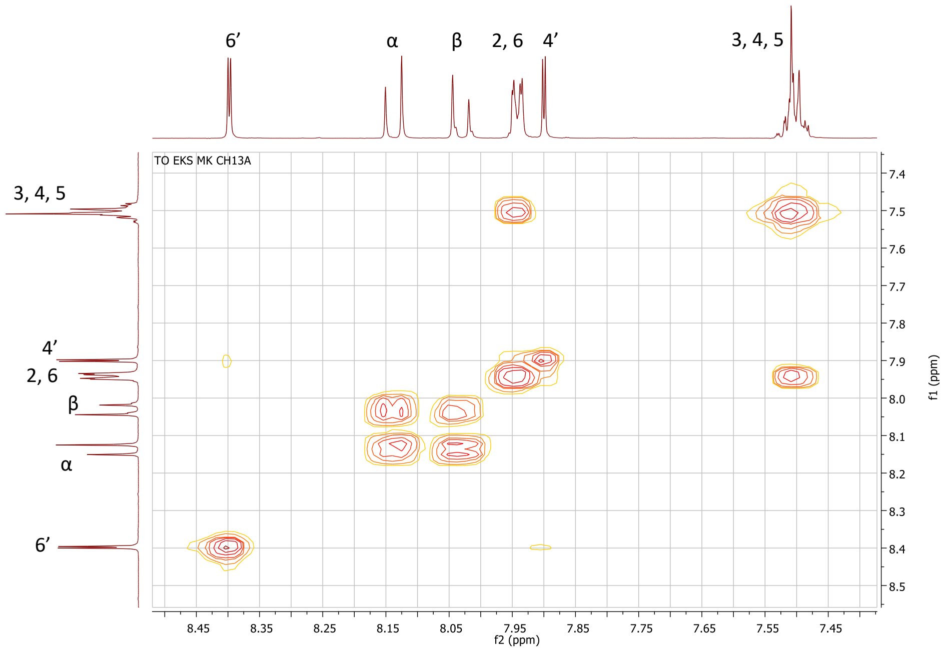

Figure S123. COSY contour map – ^1H x ^1H expansion of 3’-bromo-5’-chloro-2’-hydroxychalcone **(7)**

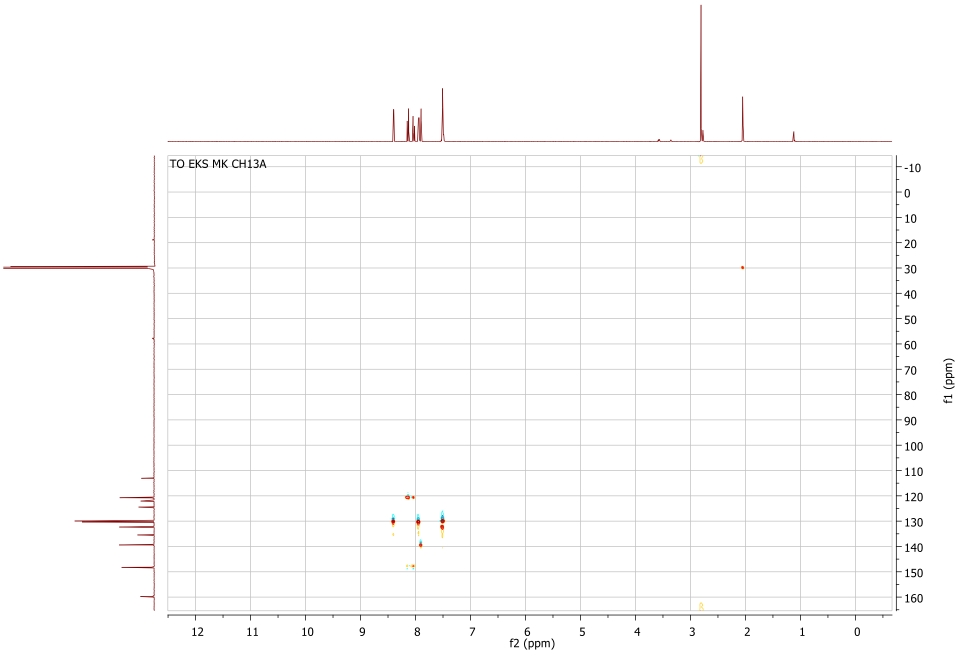

Figure S 124. HSQC contour map – ^1H x ^{13}C of 3’-bromo-5’-chloro-2’-hydroxychalcone **(7)**

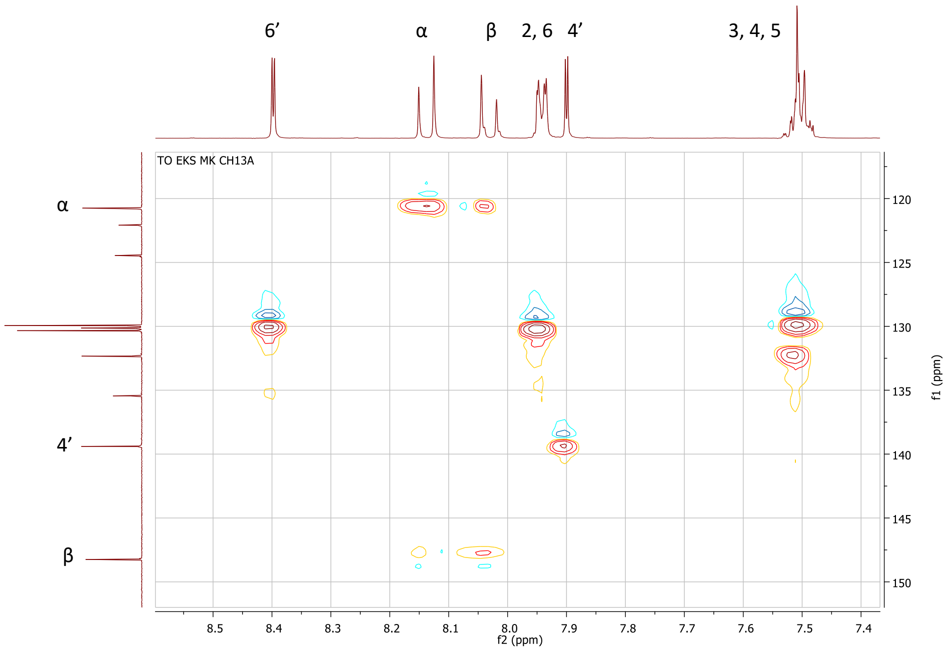

Figure S125. HSQC contour map – ^{1}H x ^{13}C expansion of 3’-bromo-5’-chloro-2’-hydroxychalcone **(7)**

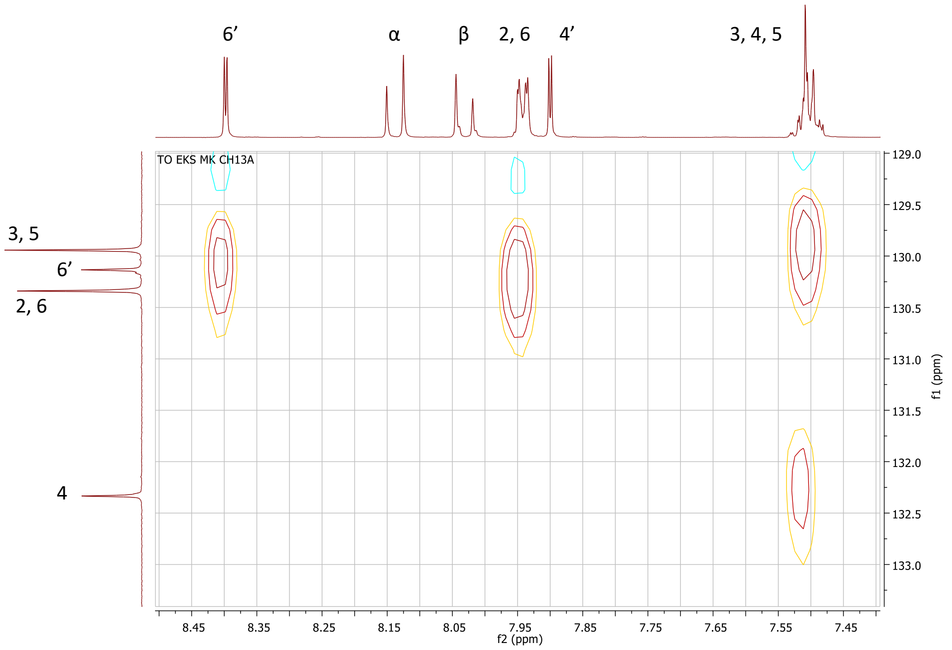

Figure S126. HSQC contour map – ^{1}H x ^{13}C expansion of 3’-bromo-5’-chloro-2’-hydroxychalcone **(7)**

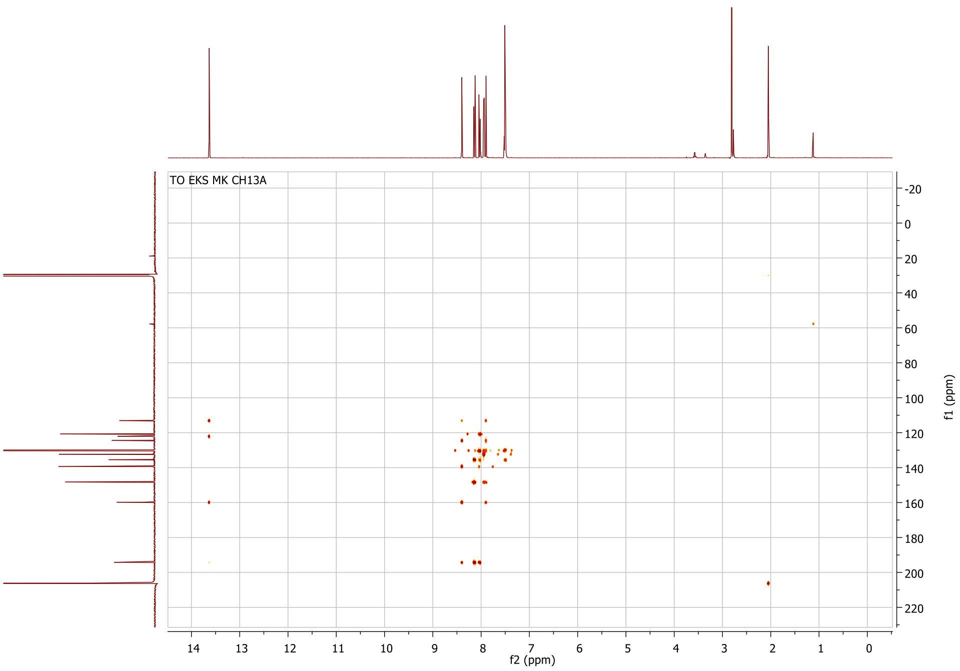

Figure S127. HMBC contour map – ^{1}H x ^{13}C of 3’-bromo-5’-chloro-2’-hydroxychalcone **(7)**

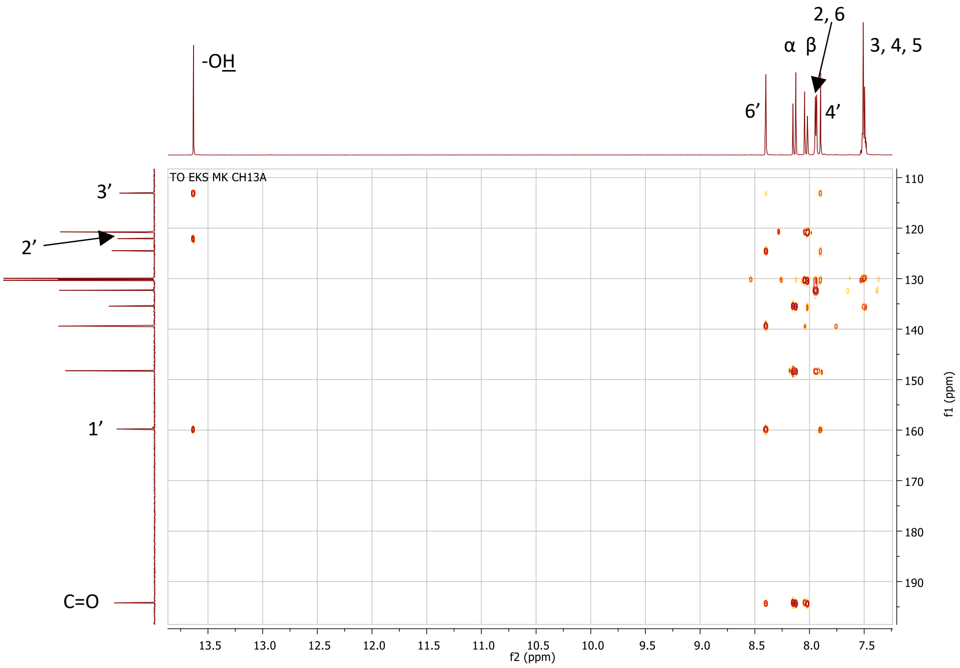

Figure S128. HMBC contour map – ^{1}H x ^{13}C expansion of 3’-bromo-5’-chloro-2’-hydroxychalcone **(7)**

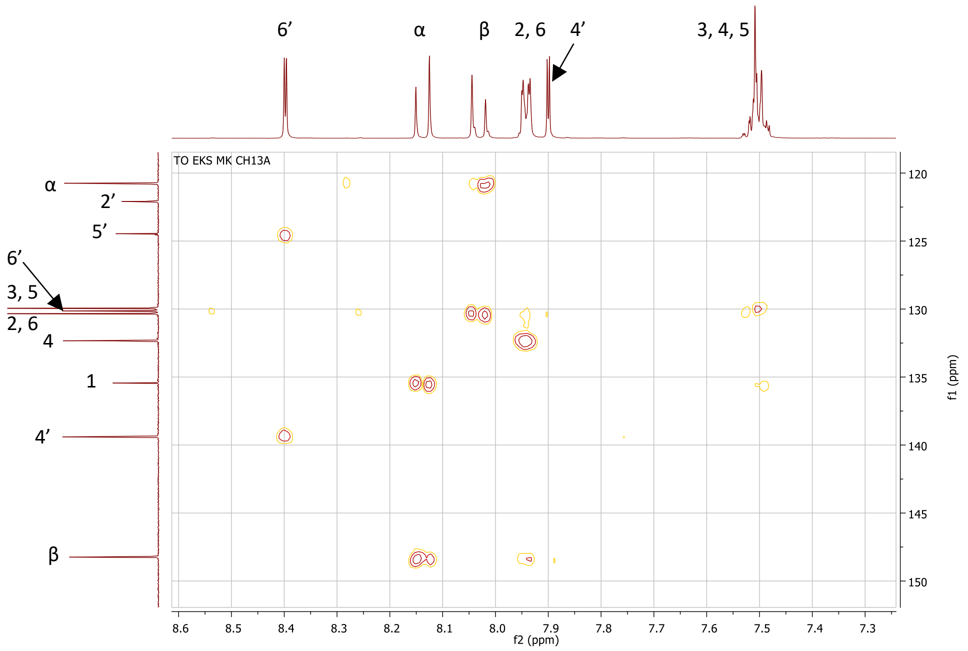

Figure S129. HMBC contour map – ^{1}H x ^{13}C expansion of 3’-bromo-5’-chloro-2’-hydroxychalcone **(7)**

Molecular Formula: $C_{22}H_{22}BrClO_8$
Formula Weight: 529.762
Ionization mode: positive
Precursor $[M + Na]^{+}$: 550.917
Collision energy (CE): -35.0

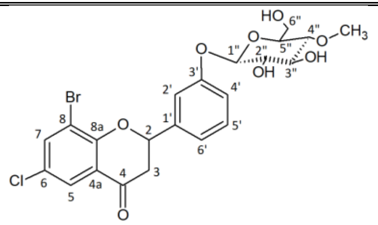

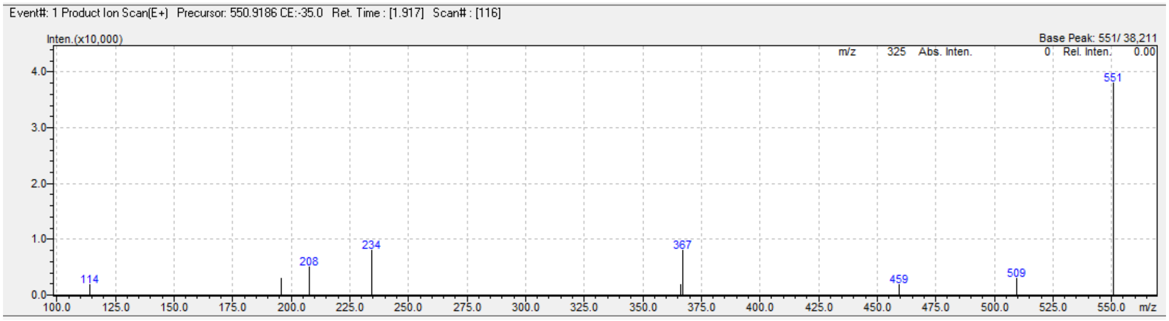

Figure S130. MS analysis of 8-bromo-6-chloroflavanone 3’-*O*-*β*-D-(4”-*O*-methyl)-glucopyranoside **(7a)**

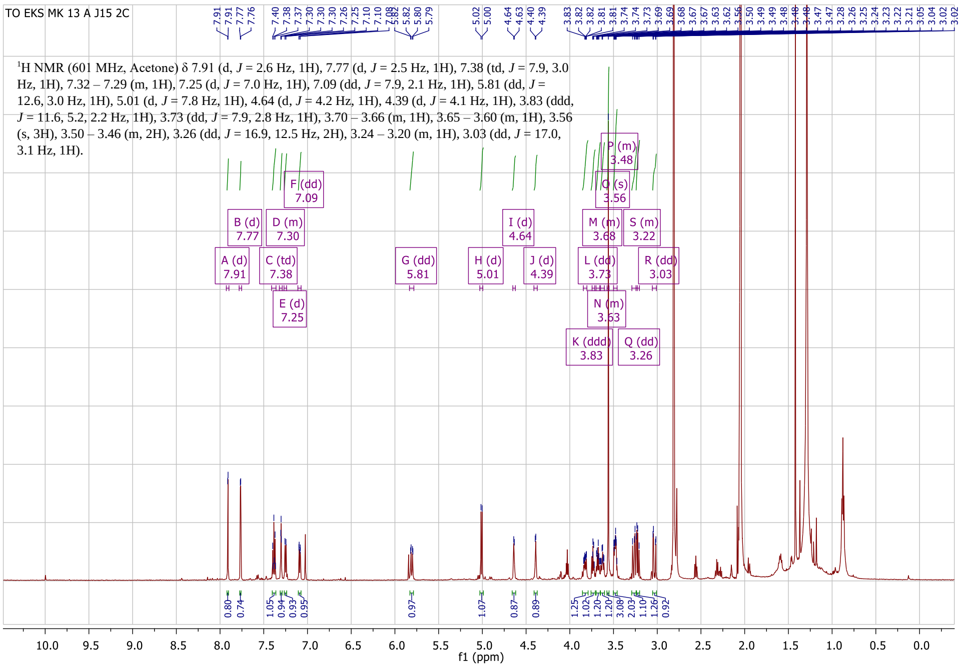

Figure S131. ^{1}H NMR spectrum of 8-bromo-6-chloroflavanone 3'-*O*-*β*-D-(4"-*O*-methyl)-glucopyranoside **(7a)**

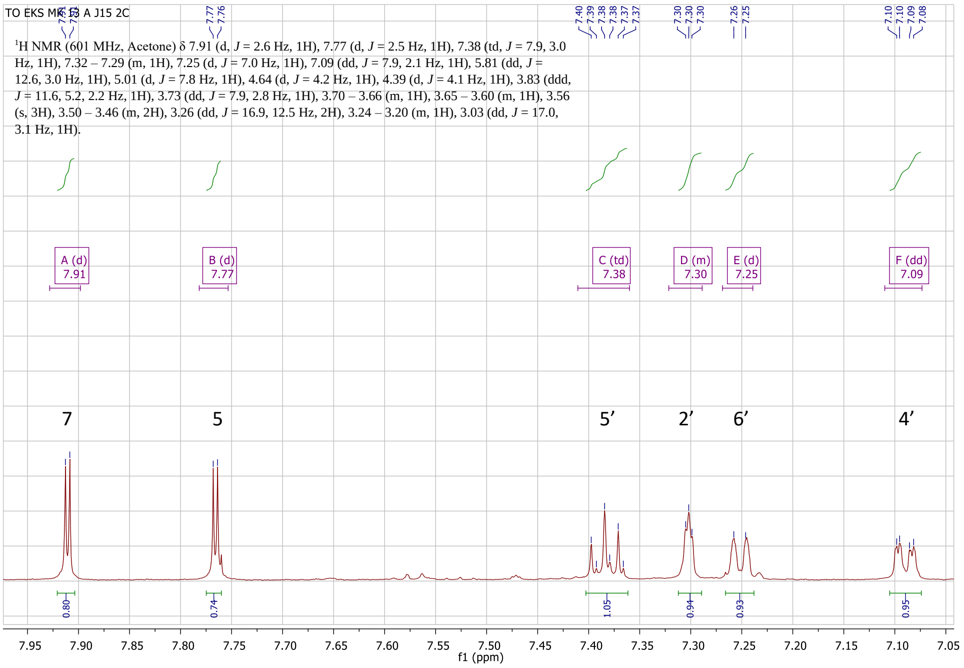

Figure S132. ^{1}H NMR spectrum expansion of 8-bromo-6-chloroflavanone 3'-*O*-*β*-D-(4"-*O*-methyl)-glucopyranoside **(7a)**

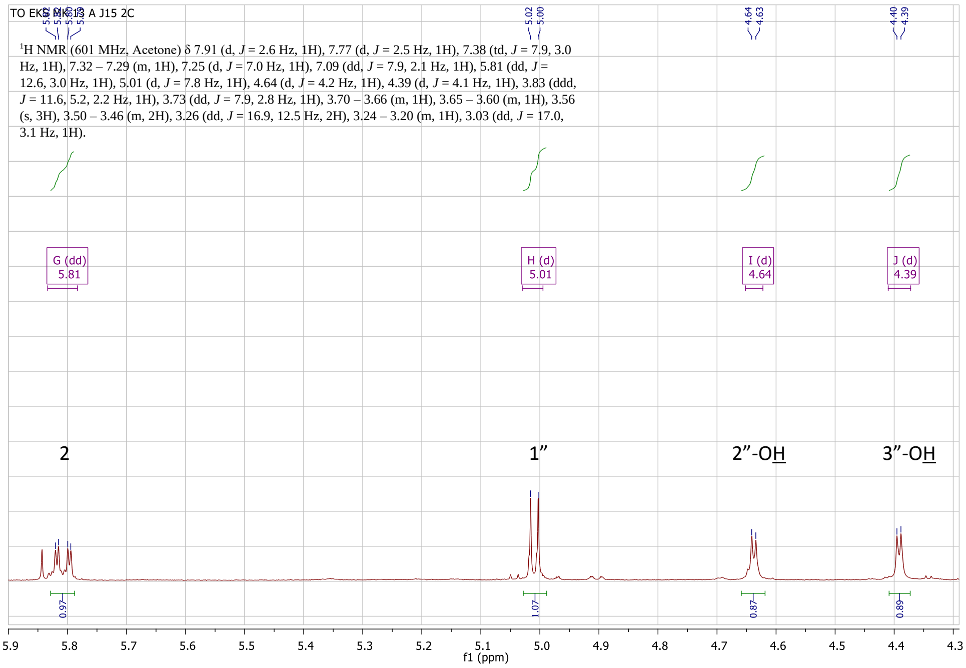

Figure S133. ^{1}H NMR spectrum expansion of 8-bromo-6-chloroflavanone 3’-*O*-*β*-D-(4”-*O*-methyl)-glucopyranoside **(7a)**

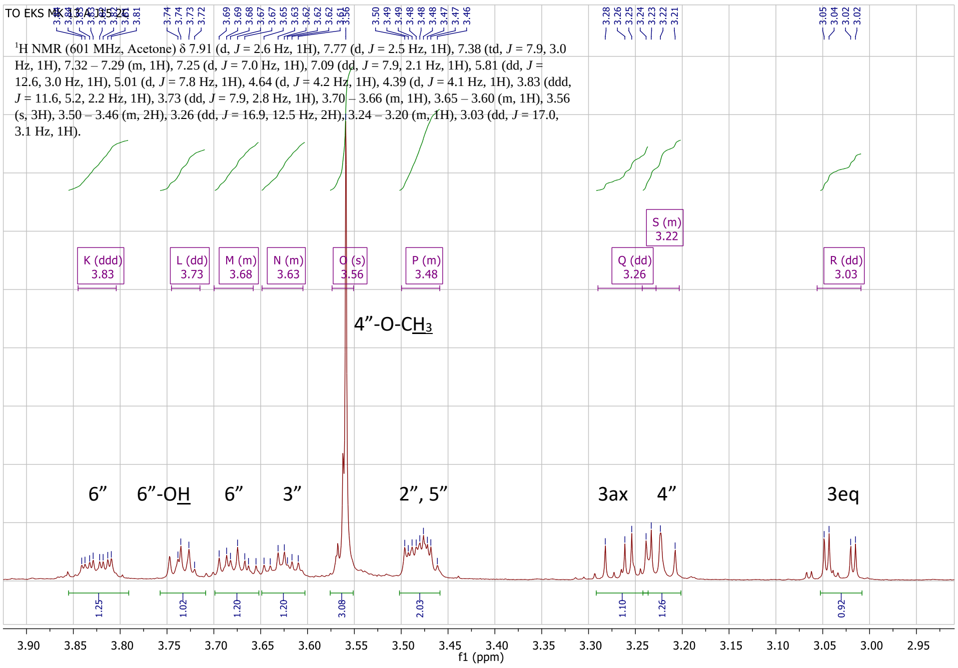

Figure S134. ^{1}H NMR spectrum expansion of 8-bromo-6-chloroflavanone 3’-*O*-*β*-D-(4”-*O*-methyl)-glucopyranoside **(7a)**

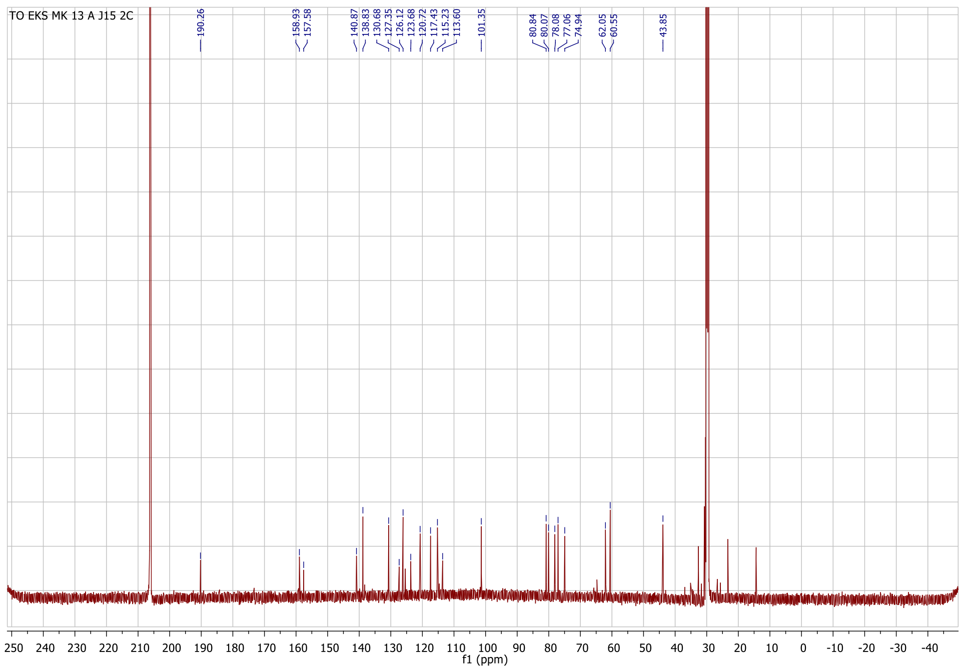

Figure S135. ^{13}C NMR spectrum of 8-bromo-6-chloroflavanone 3'-*O*-*β*-D-(4''-*O*-methyl)-glucopyranoside **(7a)**

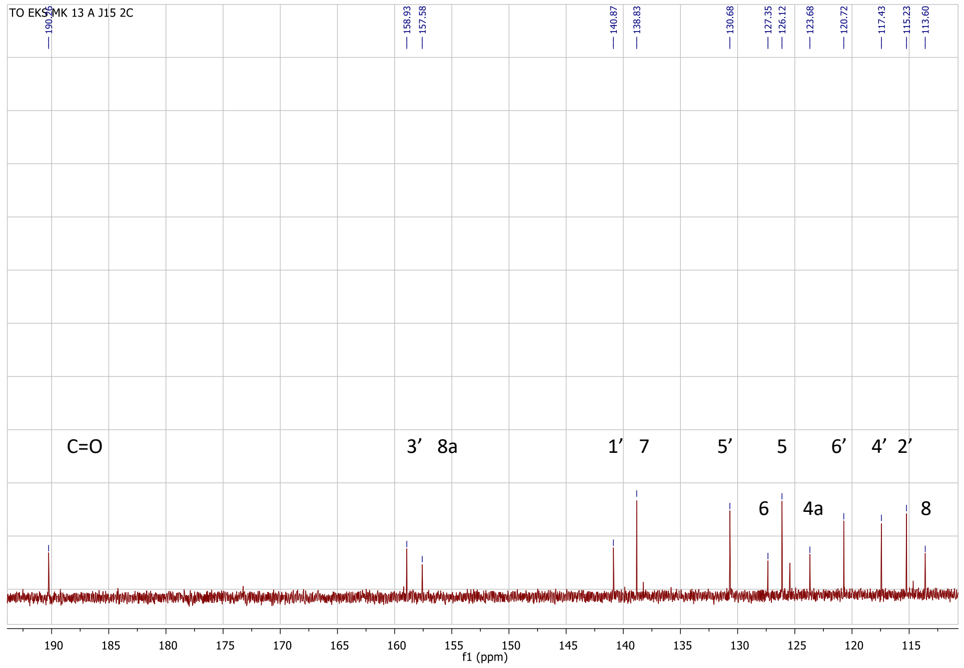

Figure S136. ^{13}C NMR spectrum expansion of 8-bromo-6-chloroflavanone 3'-*O*-*β*-D-(4''-*O*-methyl)-glucopyranoside **(7a)**

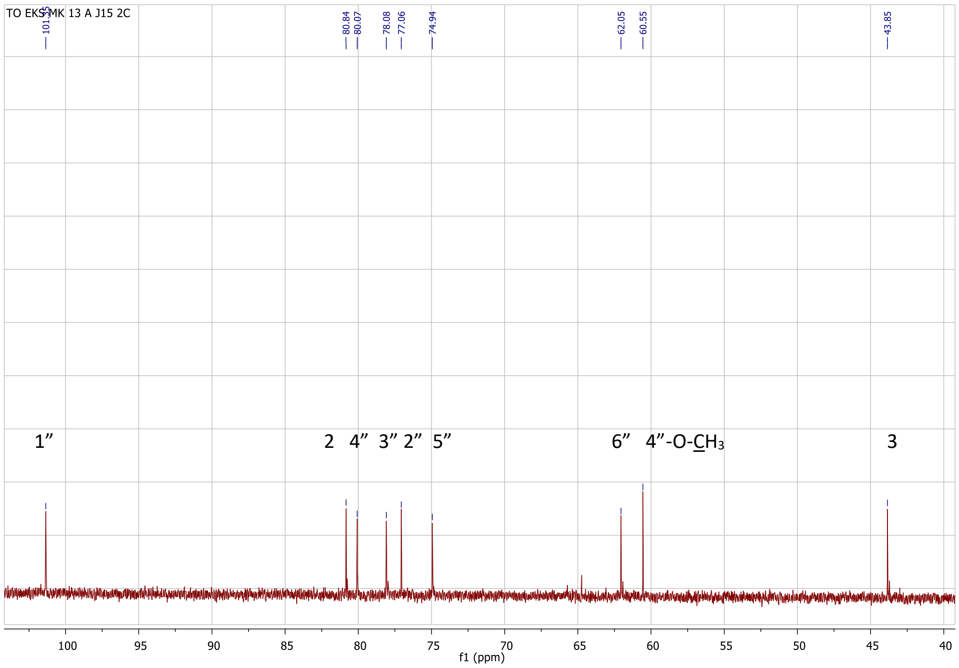

Figure S137. ^{13}C NMR spectrum expansion of 8-bromo-6-chloroflavanone 3’-*O*-*β*-D-(4”-*O*-methyl)-glucopyranoside **(7a)**

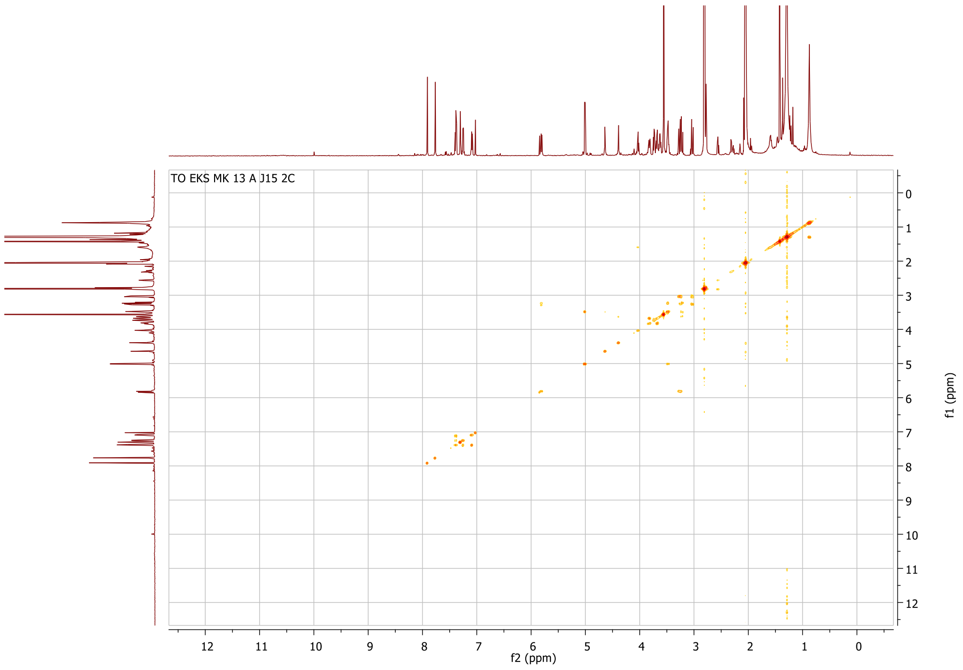

Figure S138. COSY contour map – ^{1}H x ^{1}H of 8-bromo-6-chloroflavanone 3’-*O*-*β*-D-(4”-*O*-methyl)-glucopyranoside **(7a)**

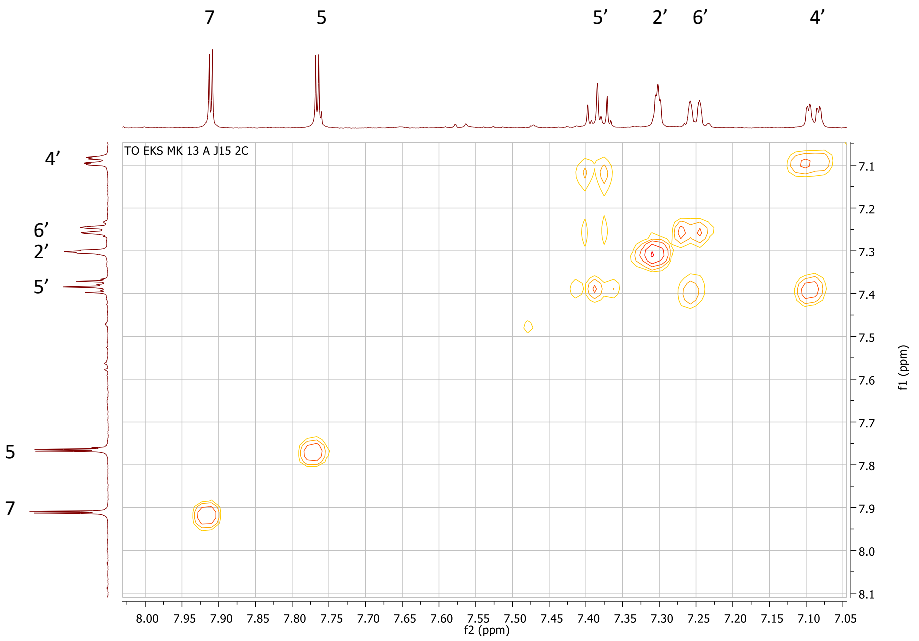

Figure S139. COSY contour map – ^{1}H x ^{1}H expansion of 8-bromo-6-chloroflavanone 3’-*O*-*β*-D-(4”-*O*-methyl)-glucopyranoside **(7a)**

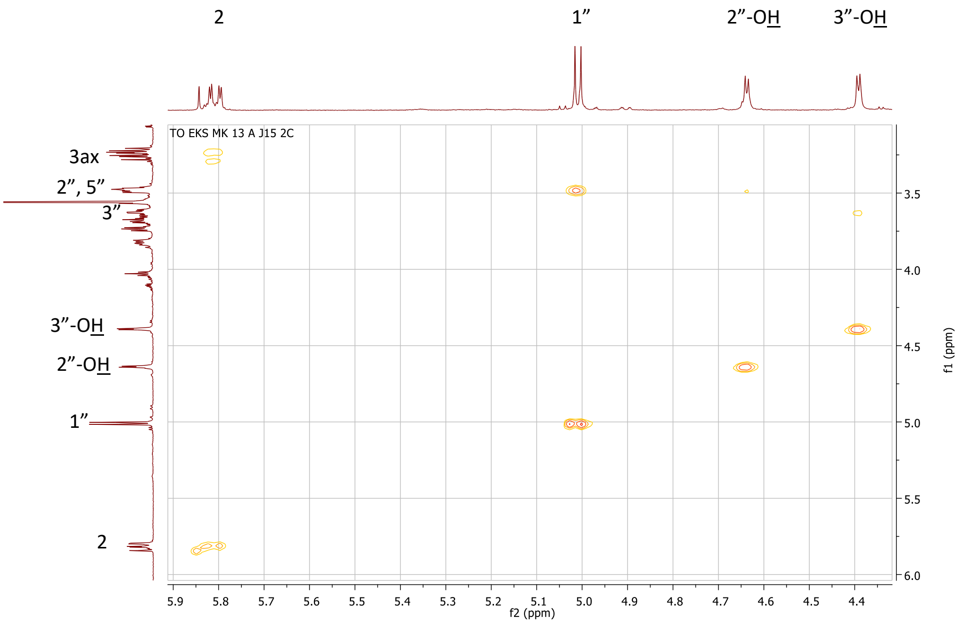

Figure S140. COSY contour map – ^{1}H x ^{1}H expansion of 8-bromo-6-chloroflavanone 3’-*O*-*β*-D-(4”-*O*-methyl)-glucopyranoside **(7a)**

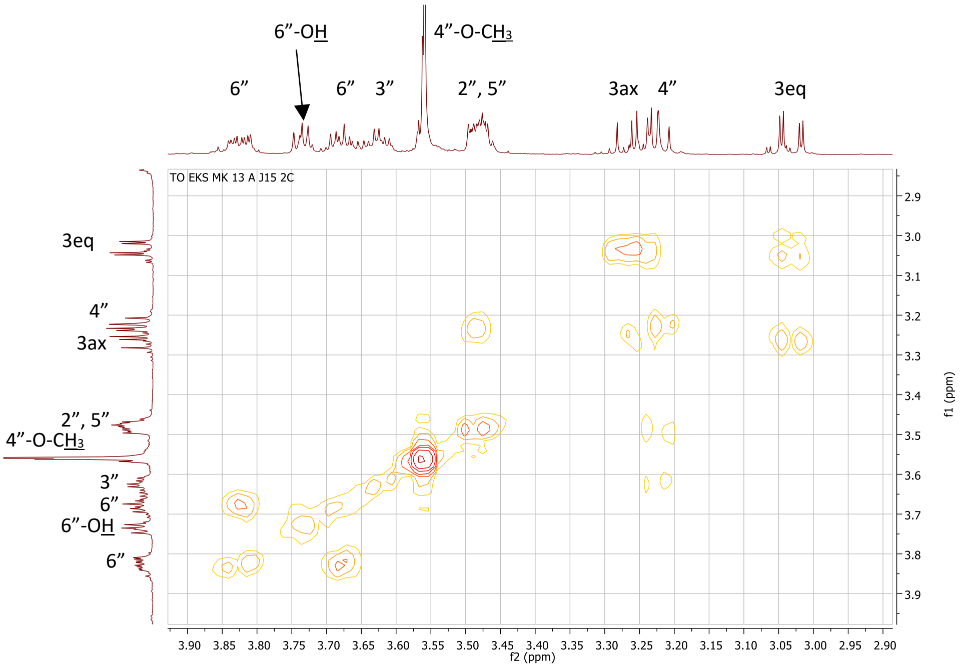

Figure S141. COSY contour map – ^{1}H x ^{1}H expansion of 8-bromo-6-chloroflavanone 3'-*O*-*β*-D-(4"-*O*-methyl)-glucopyranoside **(7a)**

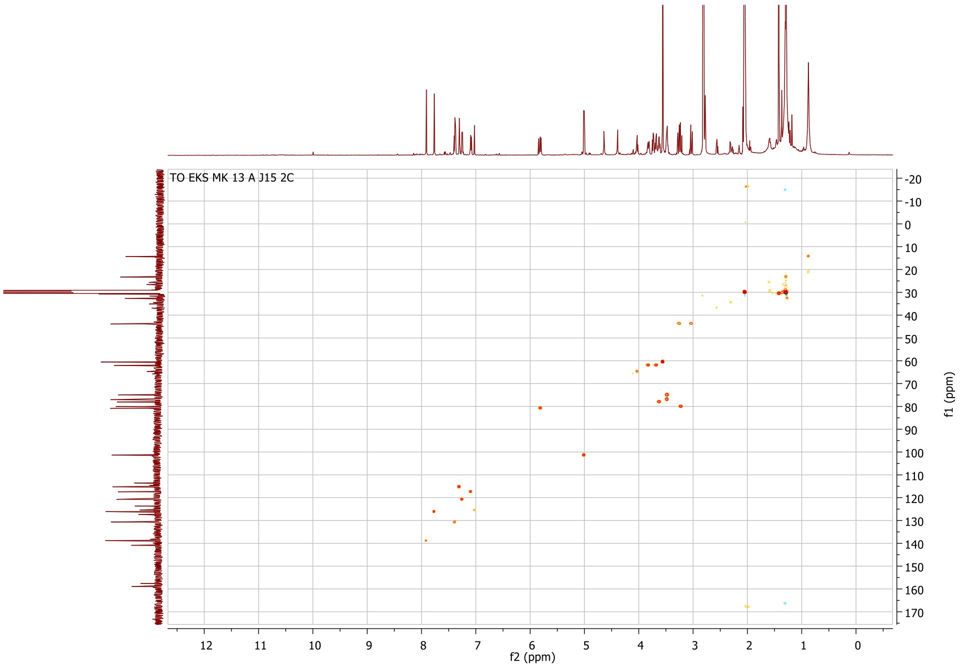

Figure S142. HSQC contour map – ^{1}H x ^{13}C of 8-bromo-6-chloroflavanone 3'-*O*-*β*-D-(4"-*O*-methyl)-glucopyranoside **(7a)**

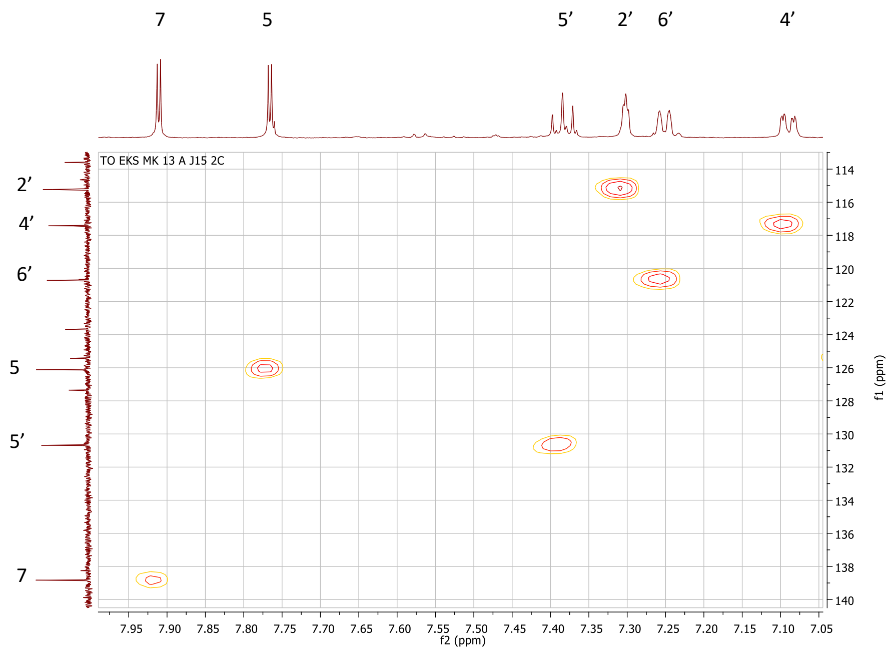

Figure S143. HSQC contour map – ^{1}H x ^{13}C expansion of 8-bromo-6-chloroflavanone 3’-*O*-*β*-D-(4”-*O*-methyl)-glucopyranoside **(7a)**

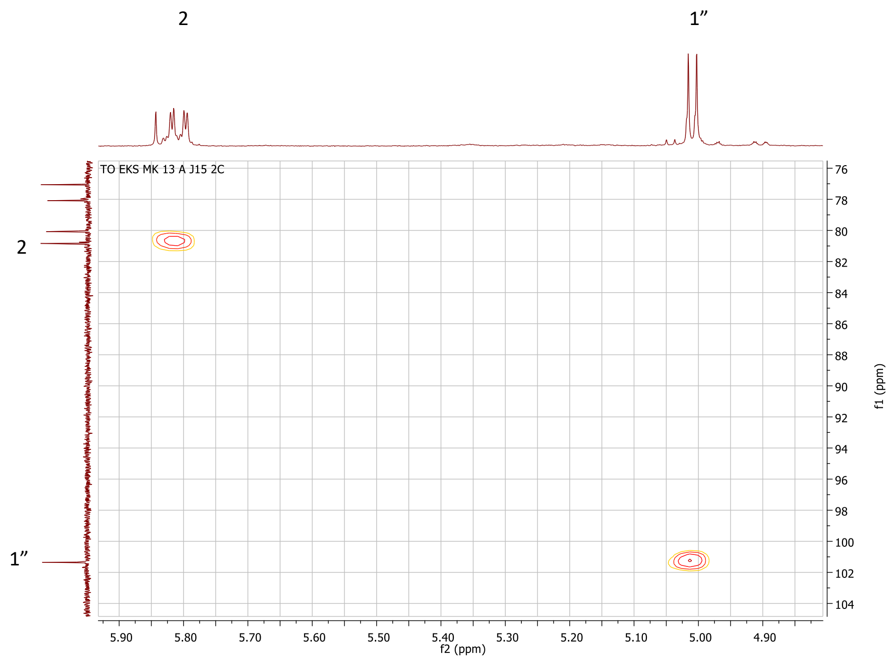

Figure S144. HSQC contour map – ^{1}H x ^{13}C expansion of 8-bromo-6-chloroflavanone 3’-*O*-*β*-D-(4”-*O*-methyl)-glucopyranoside **(7a)**

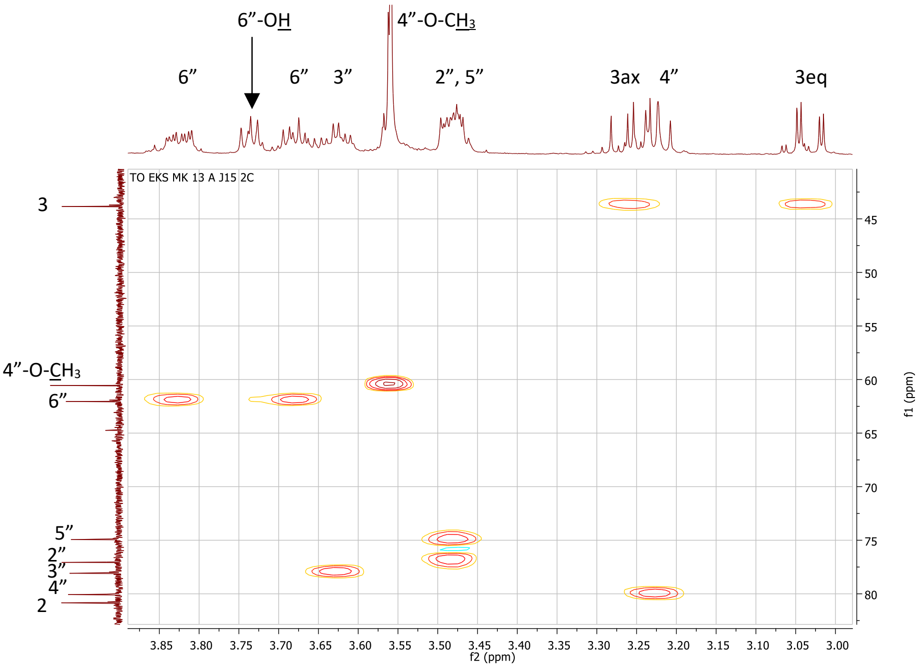

Figure S145. HSQC contour map – ^{1}H x ^{13}C expansion of 8-bromo-6-chloroflavanone 3’-*O*-*β*-D-(4”-*O*-methyl)-glucopyranoside **(7a)**

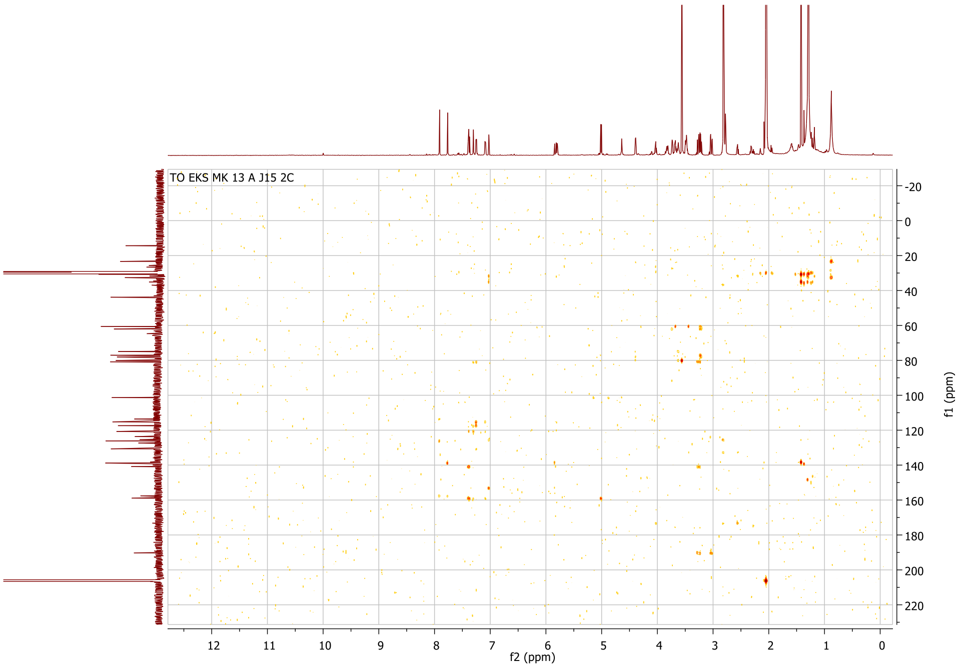

Figure S146. HMBC contour map – ^{1}H x ^{13}C of 8-bromo-6-chloroflavanone 3’-*O*-*β*-D-(4”-*O*-methyl)-glucopyranoside **(7a)**

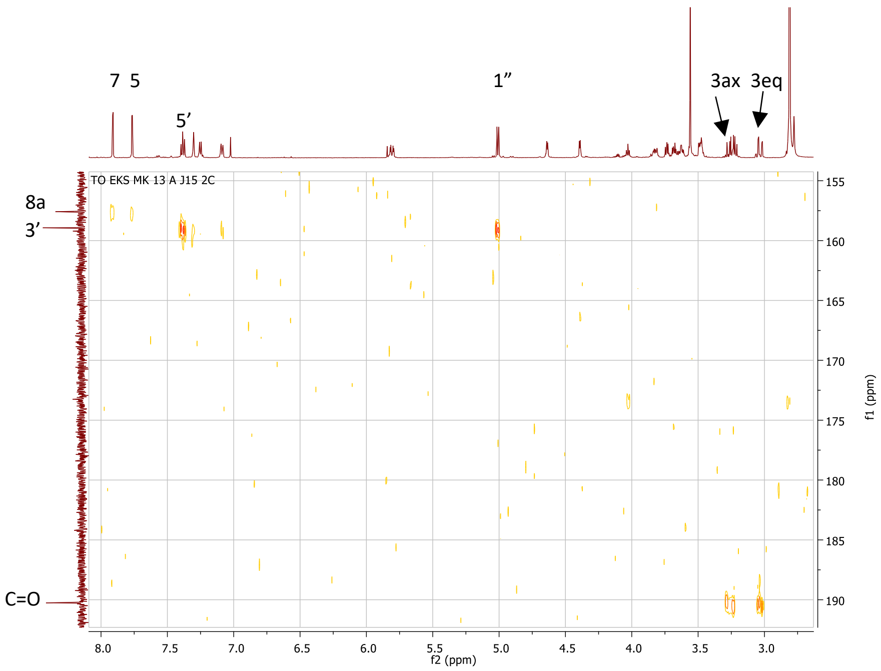

Figure S147. HMBC contour map – ^{1}H x ^{13}C expansion of 8-bromo-6-chloroflavanone 3’-*O*-*β*-D-(4”-*O*-methyl)-glucopyranoside **(7a)**

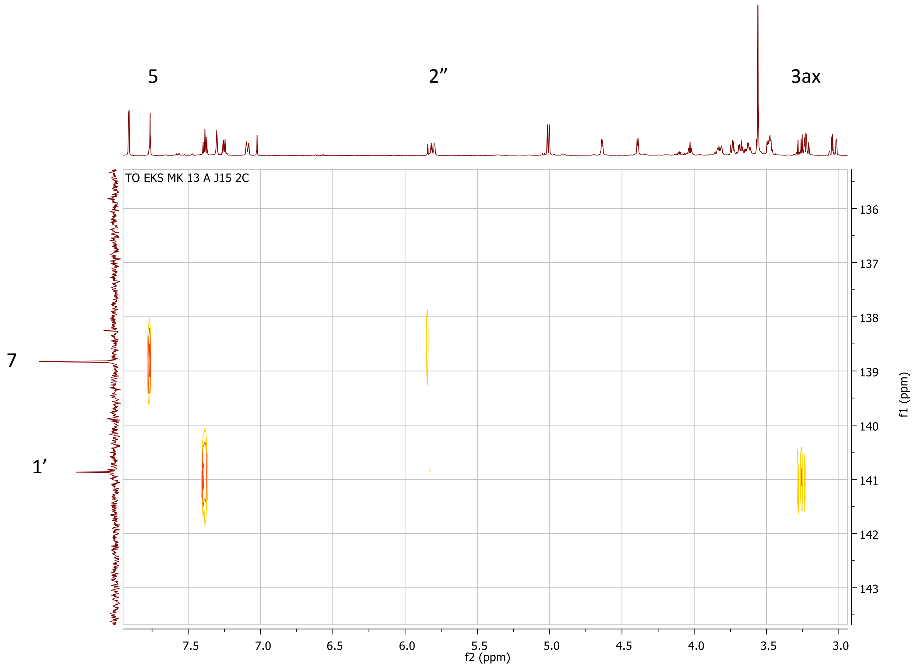

Figure S148. HMBC contour map – ^{1}H x ^{13}C expansion of 8-bromo-6-chloroflavanone 3’-*O*-*β*-D-(4”-*O*-methyl)-glucopyranoside **(7a)**

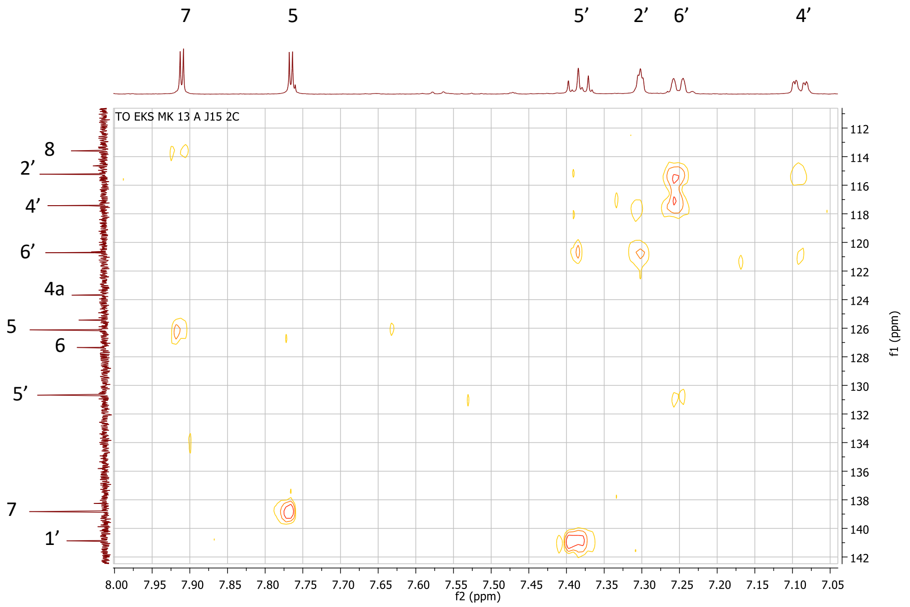

Figure S149. HMBC contour map – $^{1}H \times {}^{13}C$ expansion of 8-bromo-6-chloroflavanone 3’-*O*-*β*-D-(4”-*O*-methyl)-glucopyranoside **(7a)**

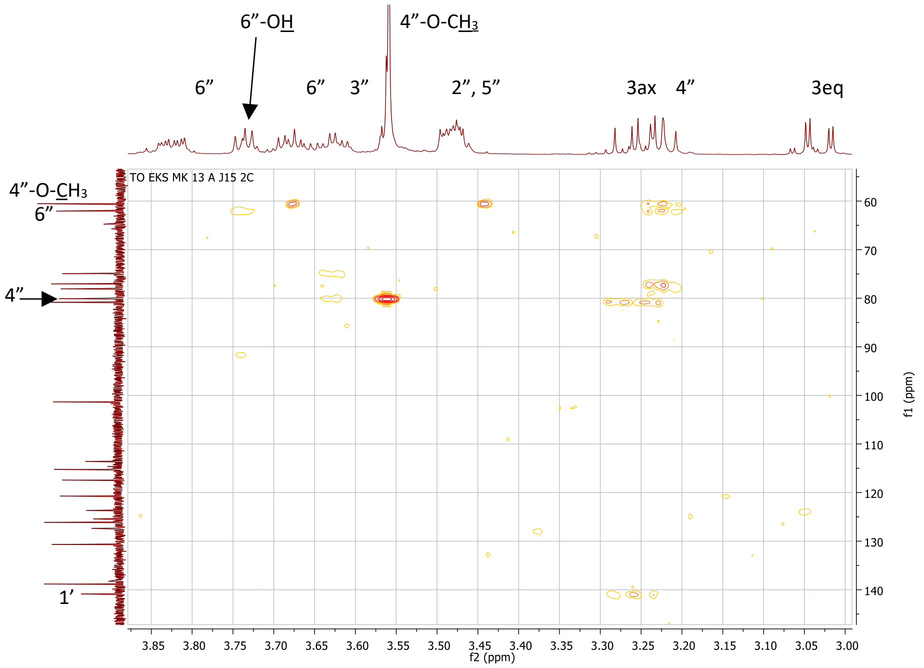

Figure S150. HMBC contour map – $^{1}H \times {}^{13}C$ expansion of 8-bromo-6-chloroflavanone 3’-*O*-*β*-D-(4”-*O*-methyl)-glucopyranoside **(7a)**

Molecular Formula: $C_{15}H_{10}BrClO_2$
Formula Weight: 337.596
Ionization mode: positive
Precursor $[M + H]^+$: 336.955
Collision energy (CE): -15.0

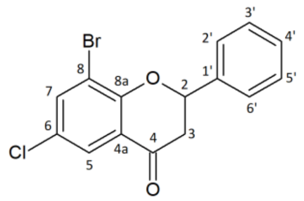

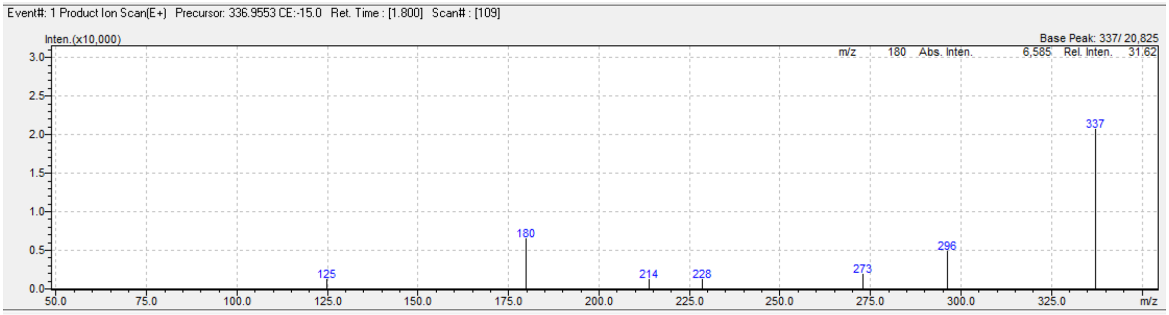

Figure S151. MS analysis of 8-bromo-6-chloroflavanone **(8)**

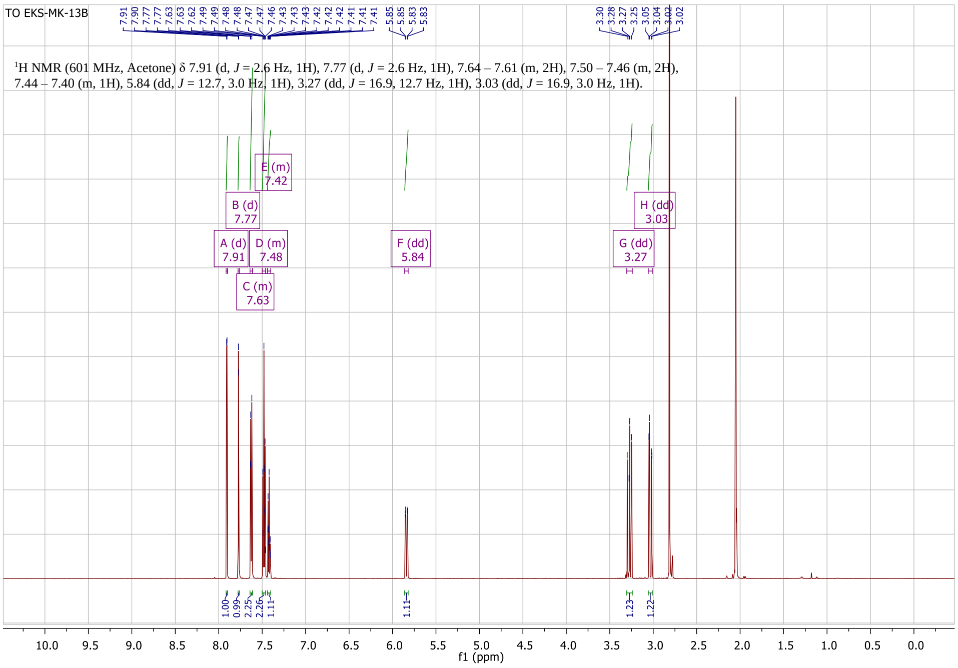

Figure S152. ^{1}H NMR spectrum of 8-bromo-6-chloroflavanone **(8)**

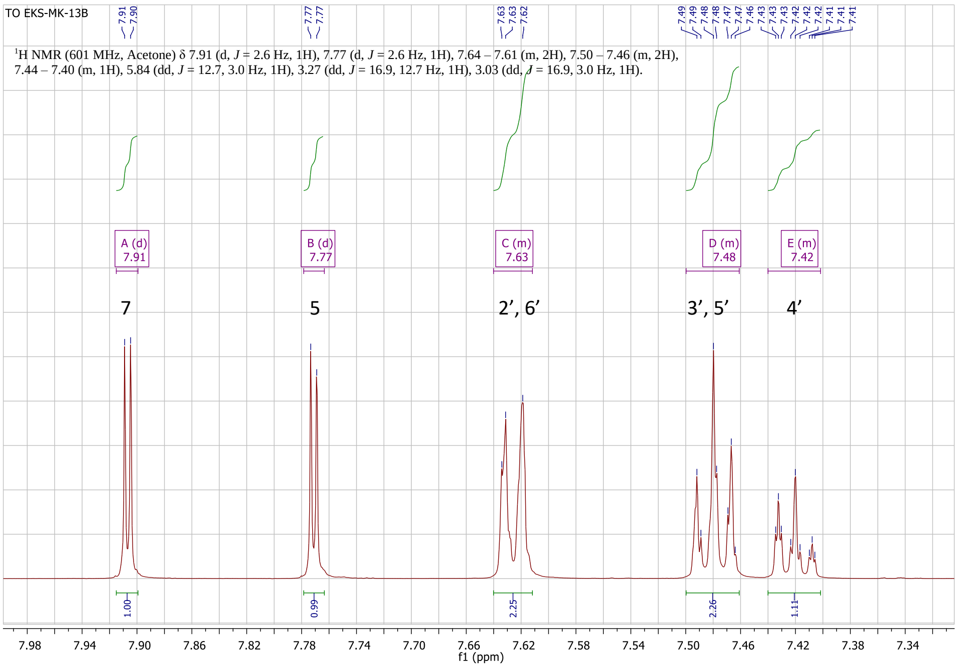

Figure S153. ^{1}H NMR spectrum expansion of 8-bromo-6-chloroflavanone **(8)**

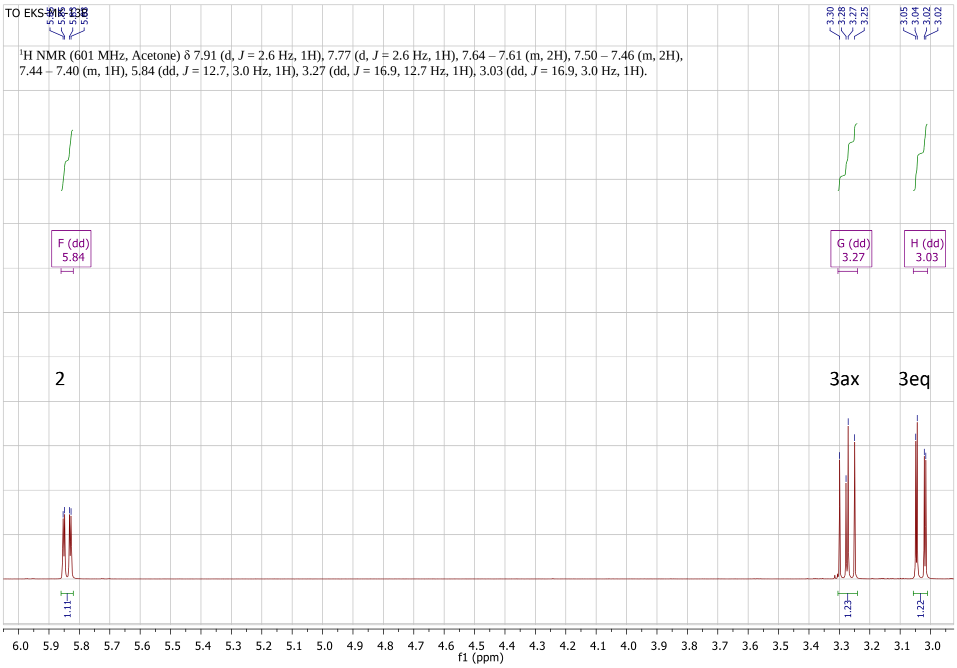

Figure S154. ^{1}H NMR spectrum expansion of 8-bromo-6-chloroflavanone **(8)**

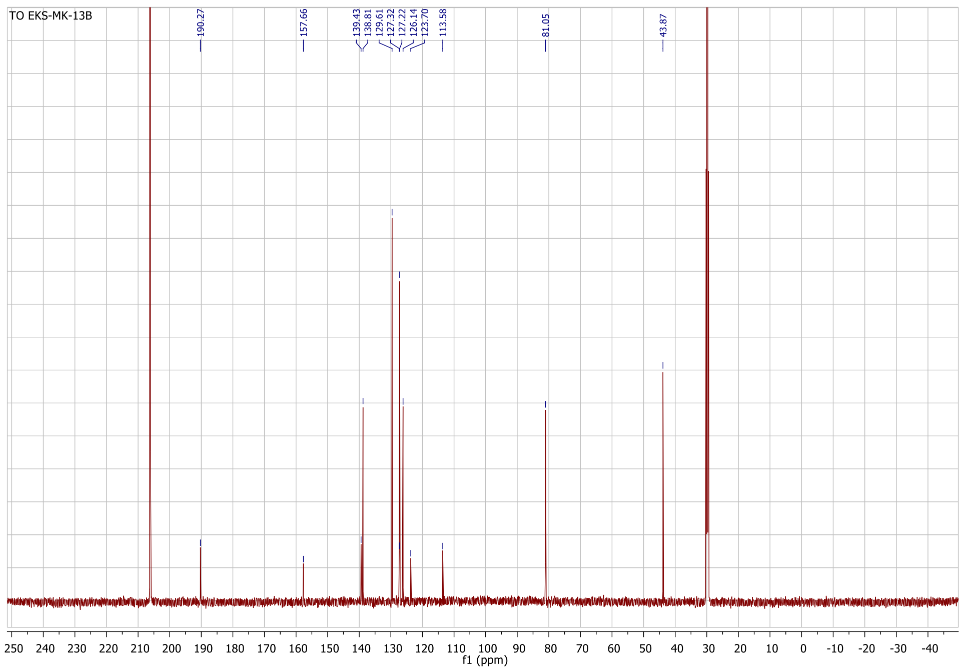

Figure S155. ^{13}C NMR spectrum of 8-bromo-6-chloroflavanone **(8)**

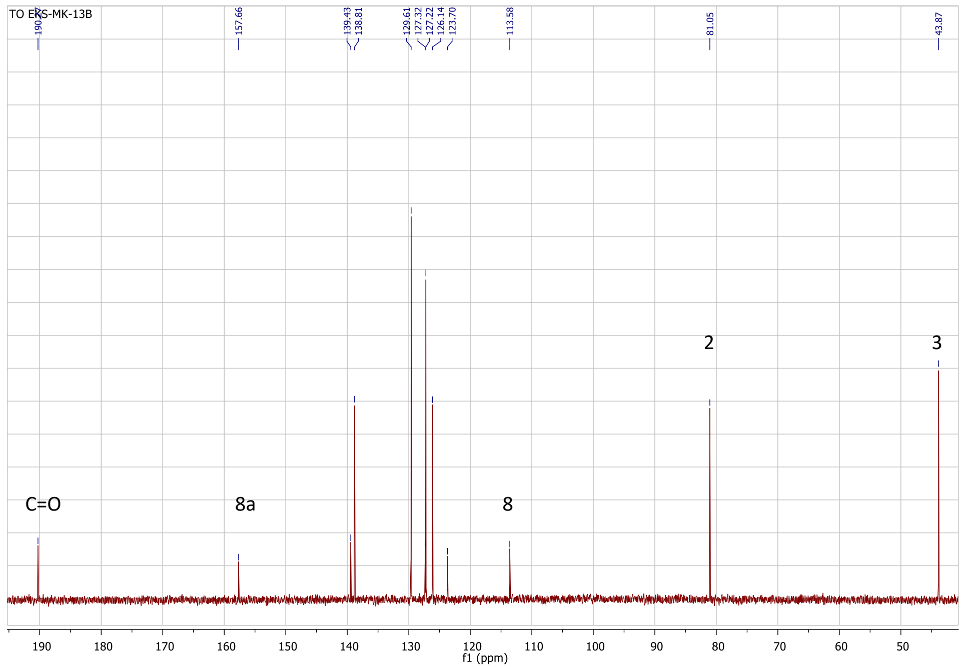

Figure S156. ^{13}C NMR spectrum expansion of 8-bromo-6-chloroflavanone **(8)**

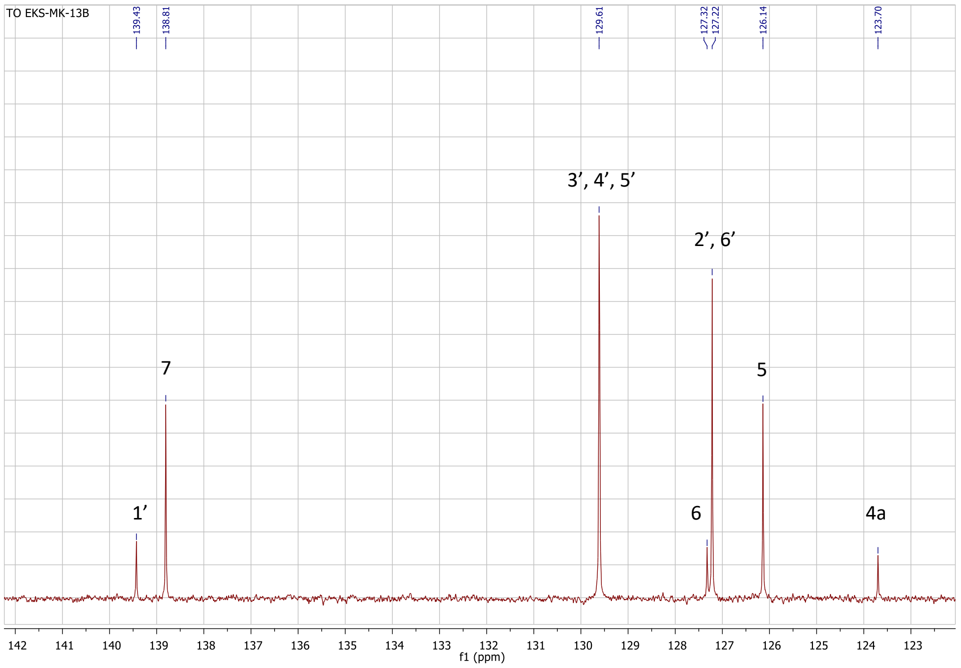

Figure S157. ^{13}C NMR spectrum expansion of 8-bromo-6-chloroflavanone **(8)**

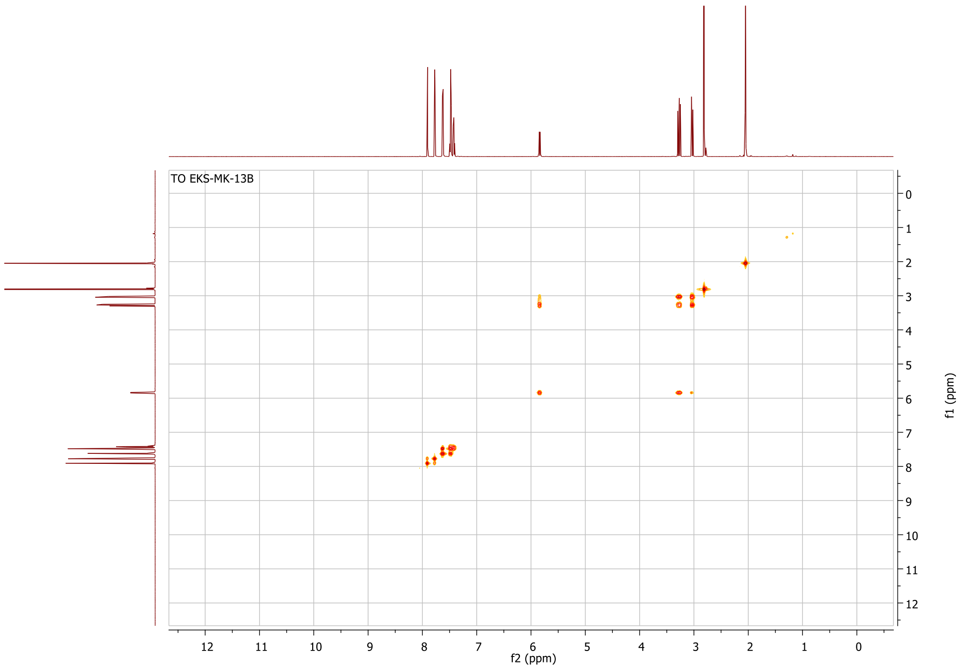

Figure S158. COSY contour map – ^{1}H x ^{1}H of 8-bromo-6-chloroflavanone **(8)**

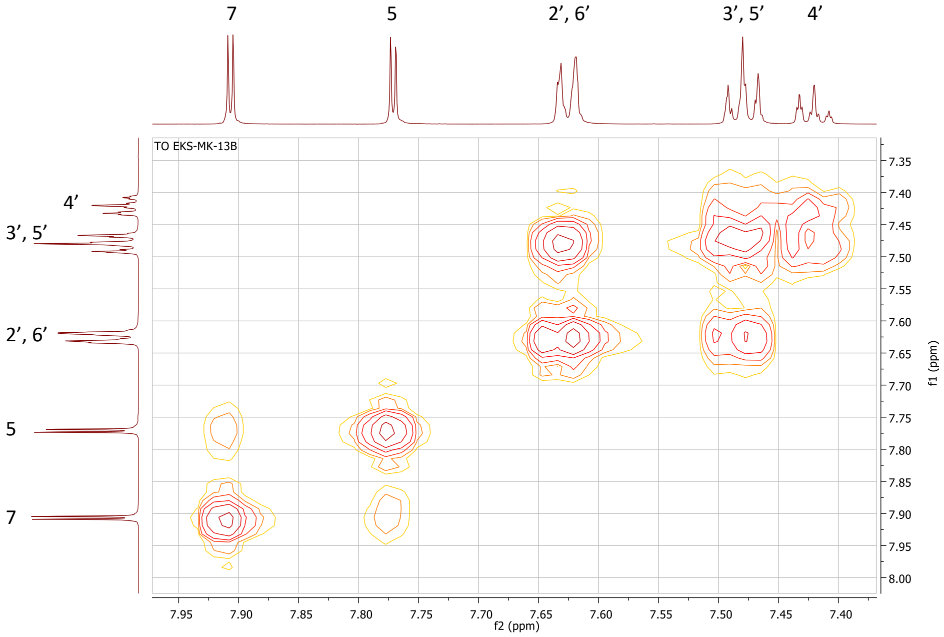

Figure S159. COSY contour map – ^{1}H x ^{1}H expansion of 8-bromo-6-chloroflavanone **(8)**

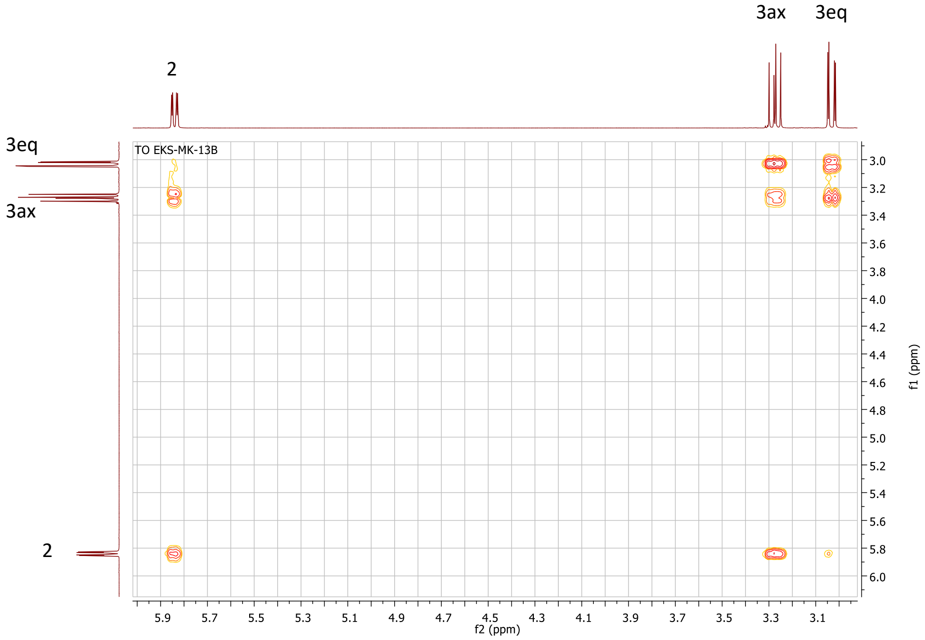

Figure S160. COSY contour map – ^{1}H x ^{1}H expansion of 8-bromo-6-chloroflavanone **(8)**

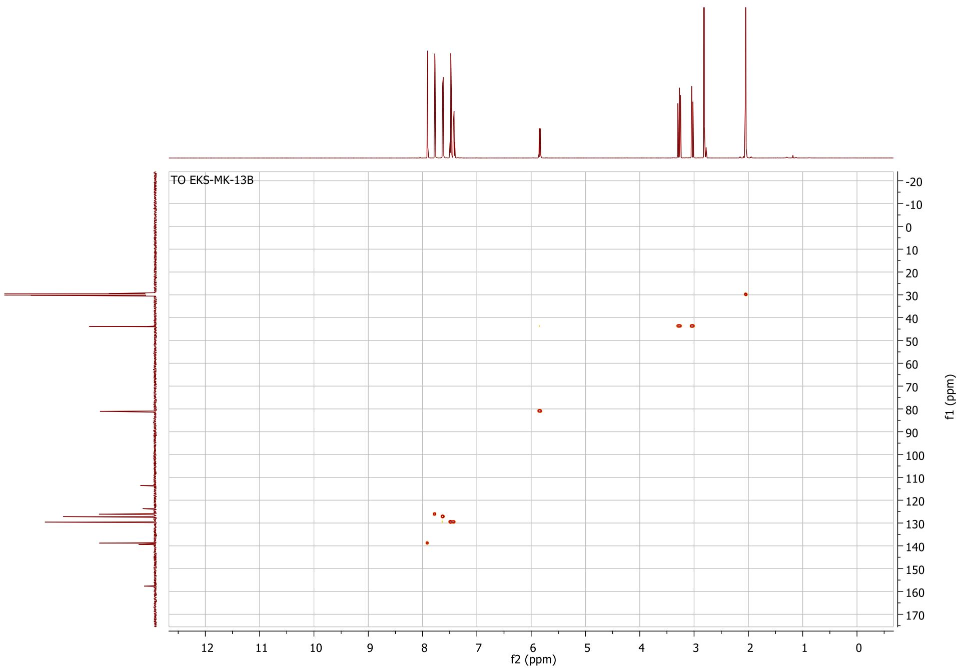

Figure S161. HSQC contour map – ^{1}H x ^{13}C of 8-bromo-6-chloroflavanone **(8)**

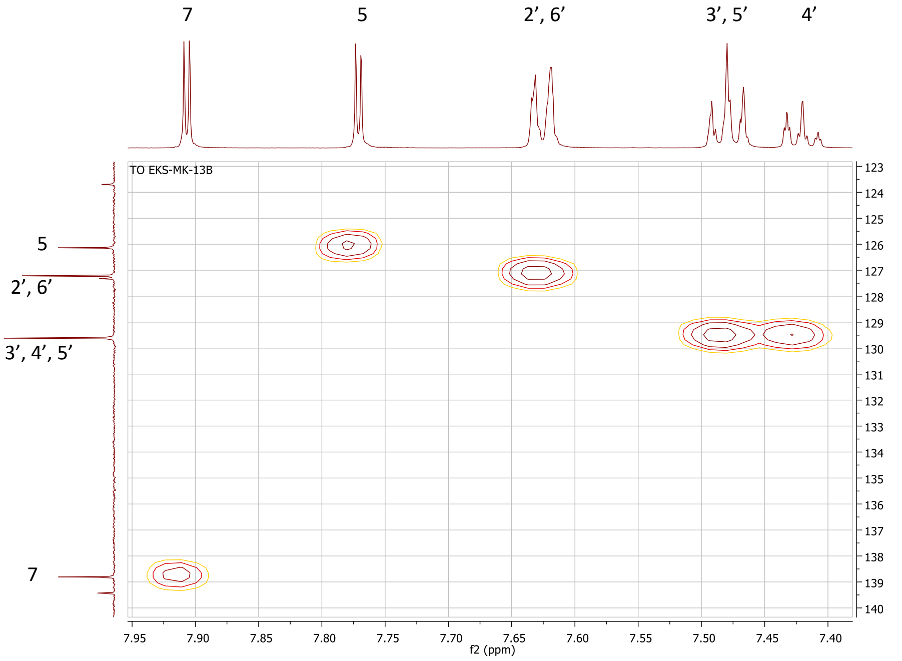

Figure S162. HSQC contour map – ^{1}H x ^{13}C expansion of 8-bromo-6-chloroflavanone **(8)**

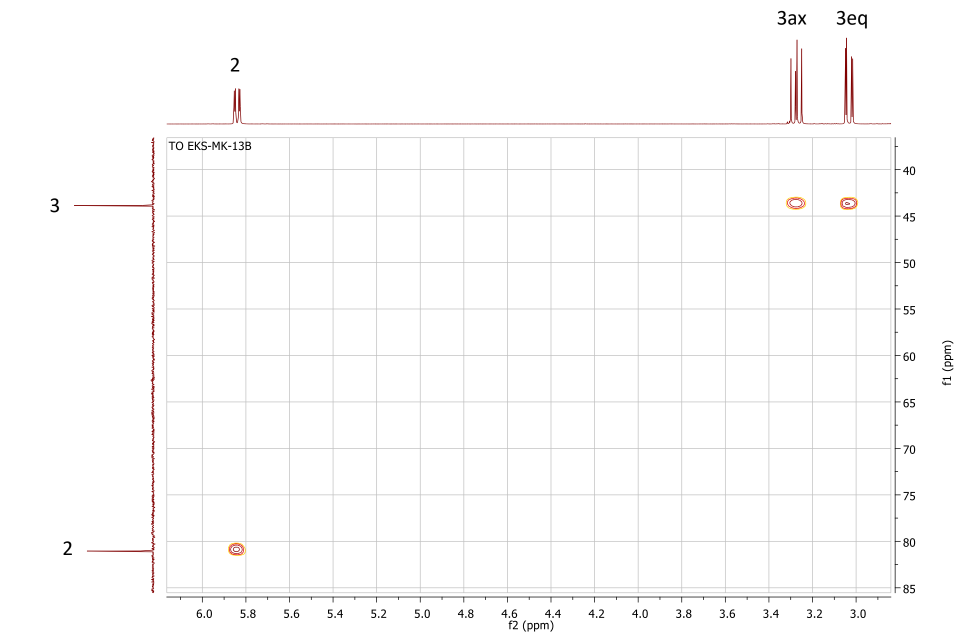

Figure S163. HSQC contour map – ^{1}H x ^{13}C expansion of 8-bromo-6-chloroflavanone **(8)**

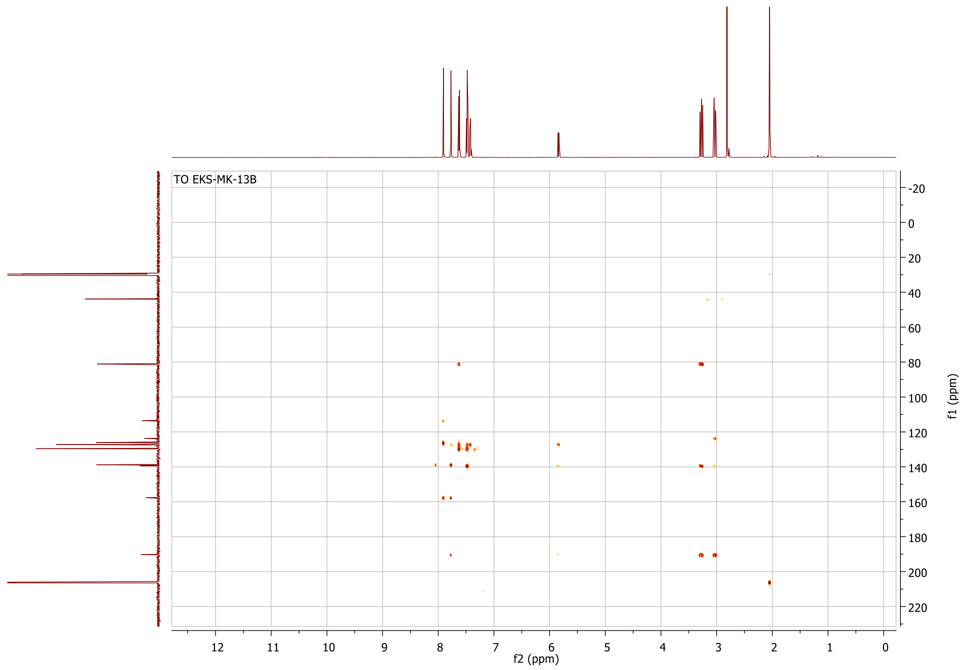

Figure S164. HMBC contour map – ^{1}H x ^{13}C of 8-bromo-6-chloroflavanone **(8)**

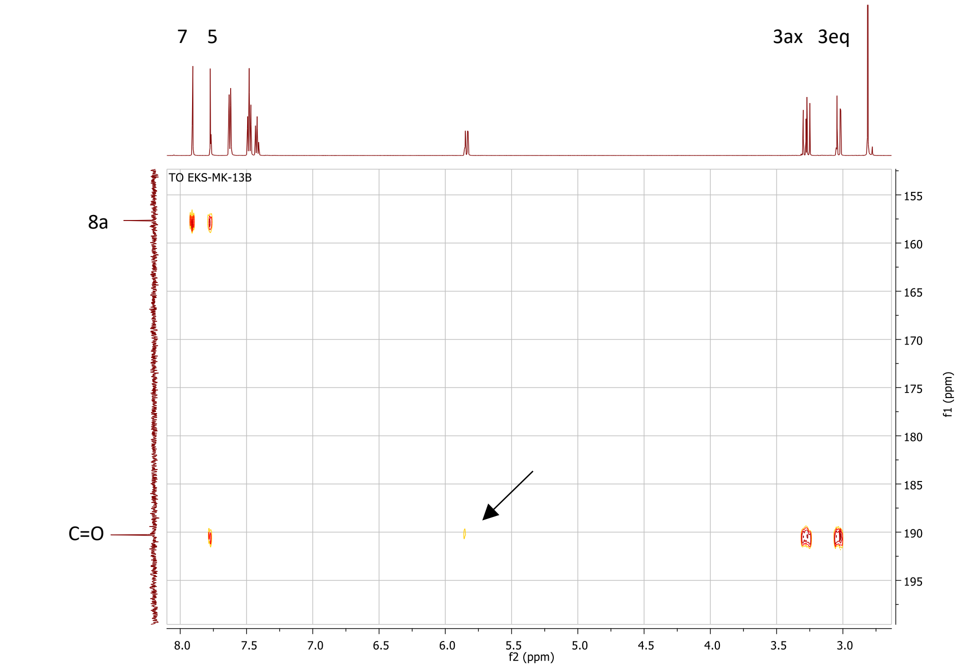

Figure S165. HMBC contour map – $^{1}H \times {}^{13}C$ expansion of 8-bromo-6-chloroflavanone **(8)**

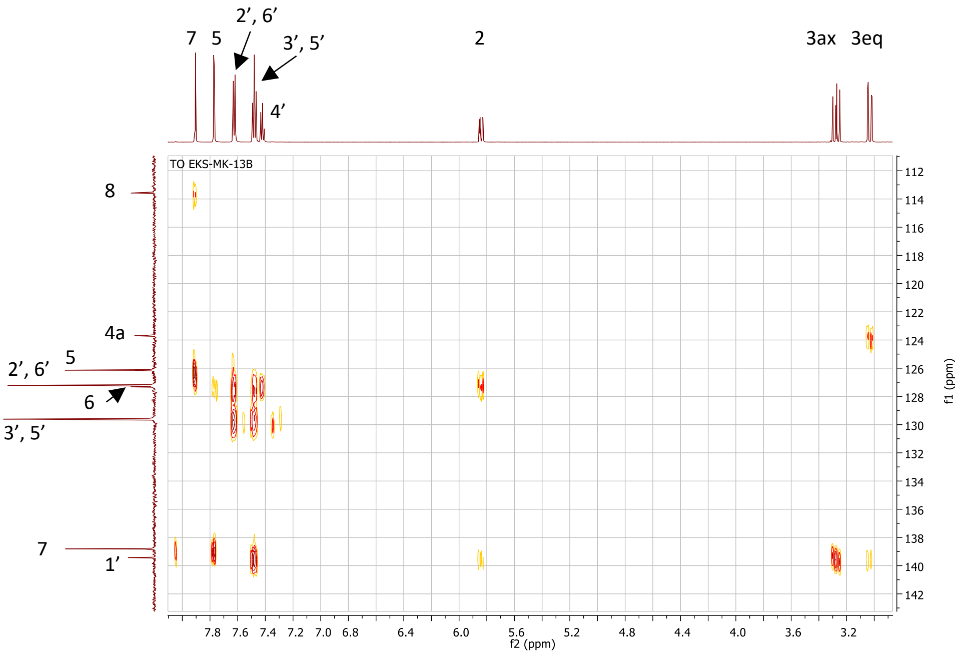

Figure S166. HMBC contour map – $^{1}H \times {}^{13}C$ expansion of 8-bromo-6-chloroflavanone **(8)**

Molecular Formula: $C_{22}H_{24}BrClO_8$
Formula Weight: 531.778
Ionization mode: positive
Precursor $[M + H]^+$: 553.014
Collision energy (CE): -25.0

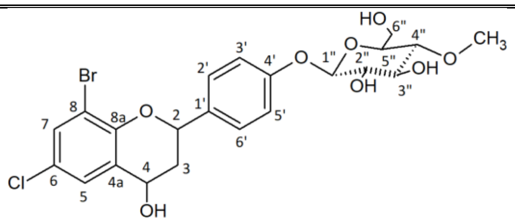

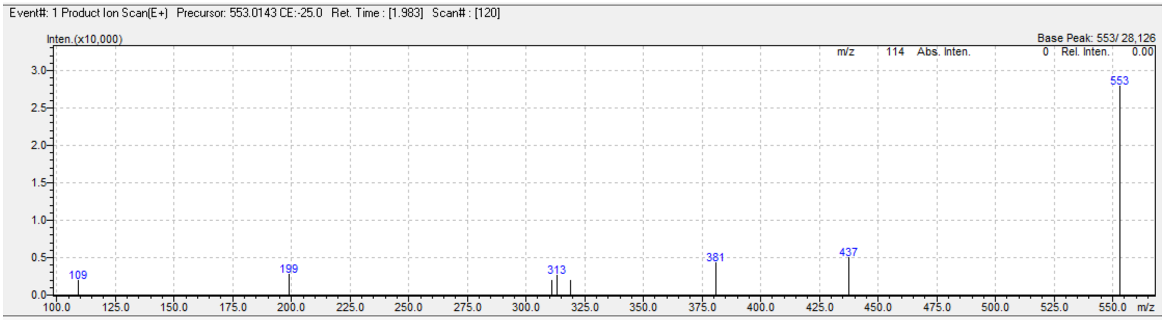

Figure S167. MS analysis of 8-bromo-6-chloroflavan-4-ol 4’-*O*-*β*-D-(4”-*O*-methyl)-glucopyranoside **(8a)**

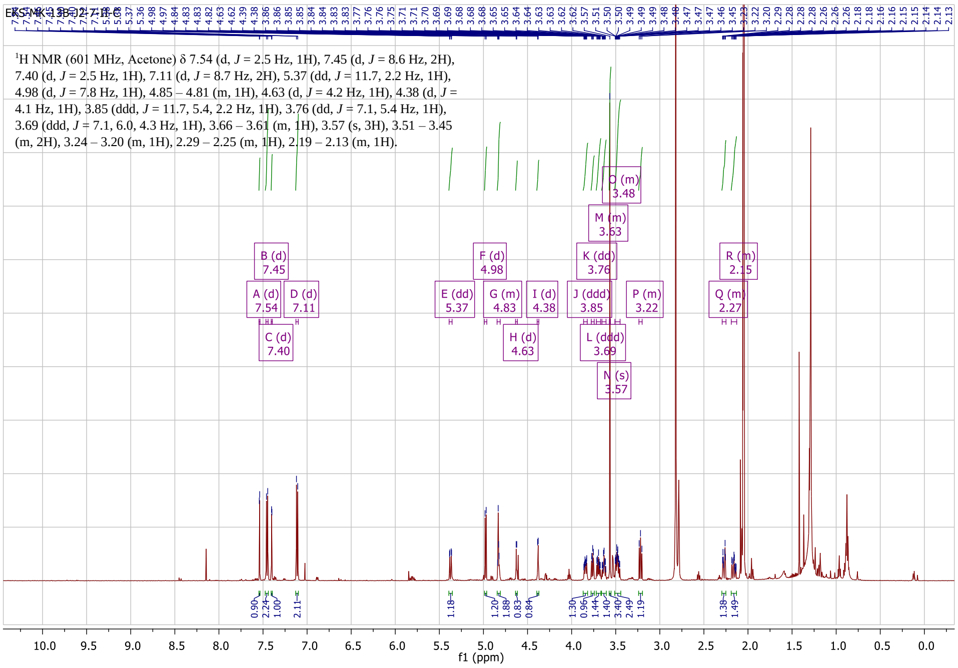

Figure S168. ^{1}H NMR spectrum of 8-bromo-6-chloroflavan-4-ol 4’-*O*-*β*-D-(4”-*O*-methyl)-glucopyranoside **(8a)**

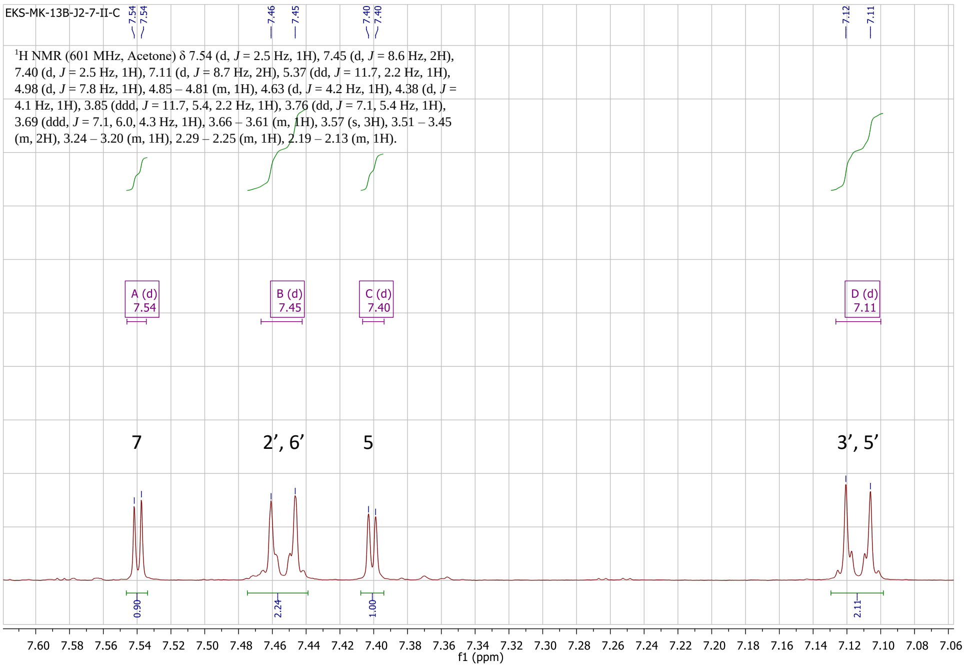

Figure S169. ^{1}H NMR spectrum expansion of 8-bromo-6-chloroflavan-4-ol 4'-*O*-*β*-D-(4''-*O*-methyl)-glucopyranoside **(8a)**

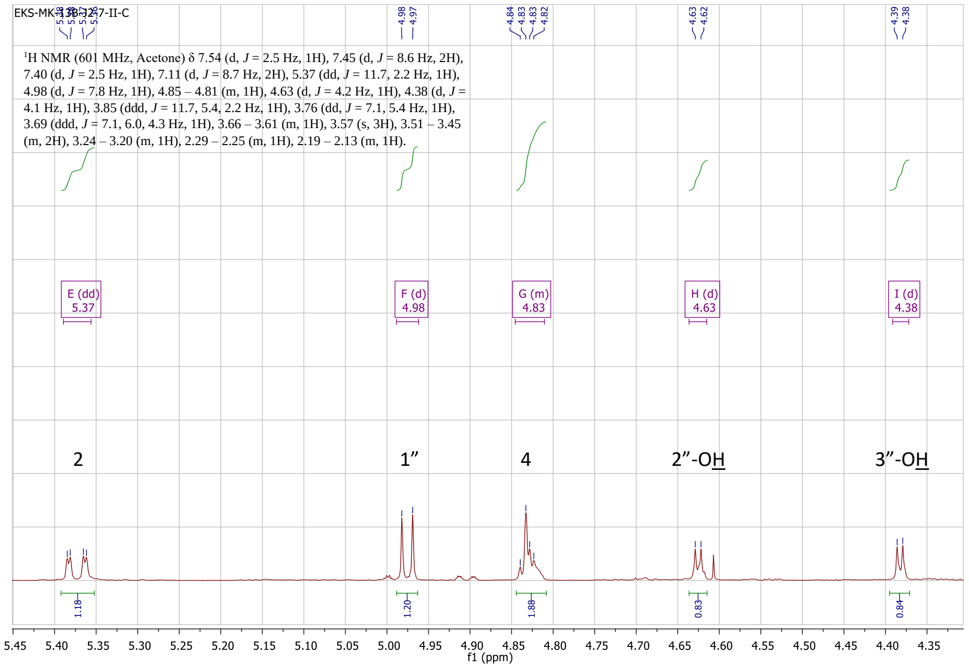

Figure S170. ^{1}H NMR spectrum expansion of 8-bromo-6-chloroflavan-4-ol 4'-*O*-*β*-D-(4''-*O*-methyl)-glucopyranoside **(8a)**

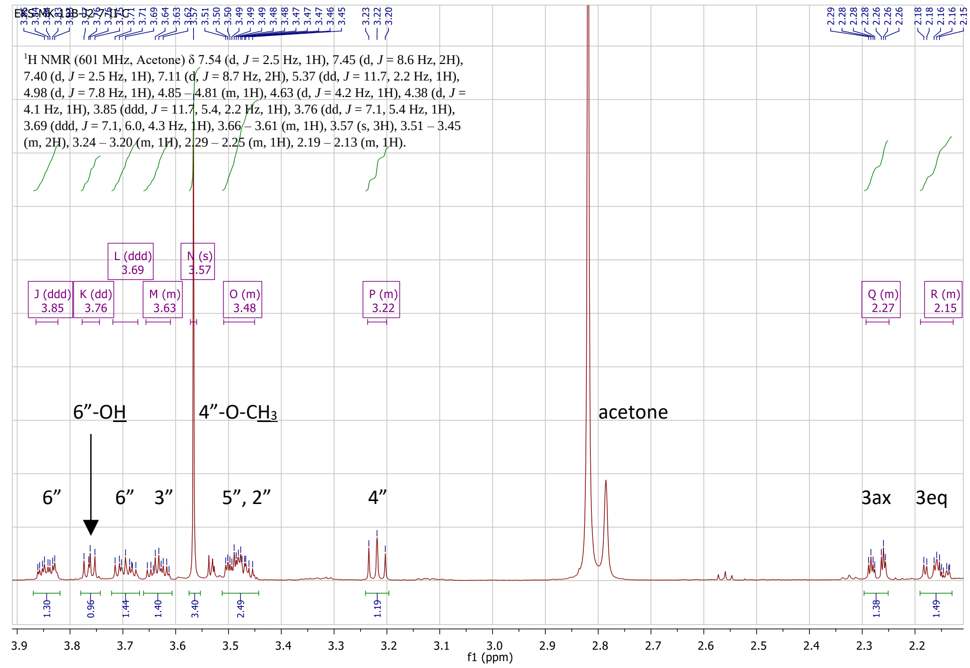

Figure S171. ^{1}H NMR spectrum expansion of 8-bromo-6-chloroflavan-4-ol 4'-*O*-*β*-D-(4''-*O*-methyl)-glucopyranoside **(8a)**

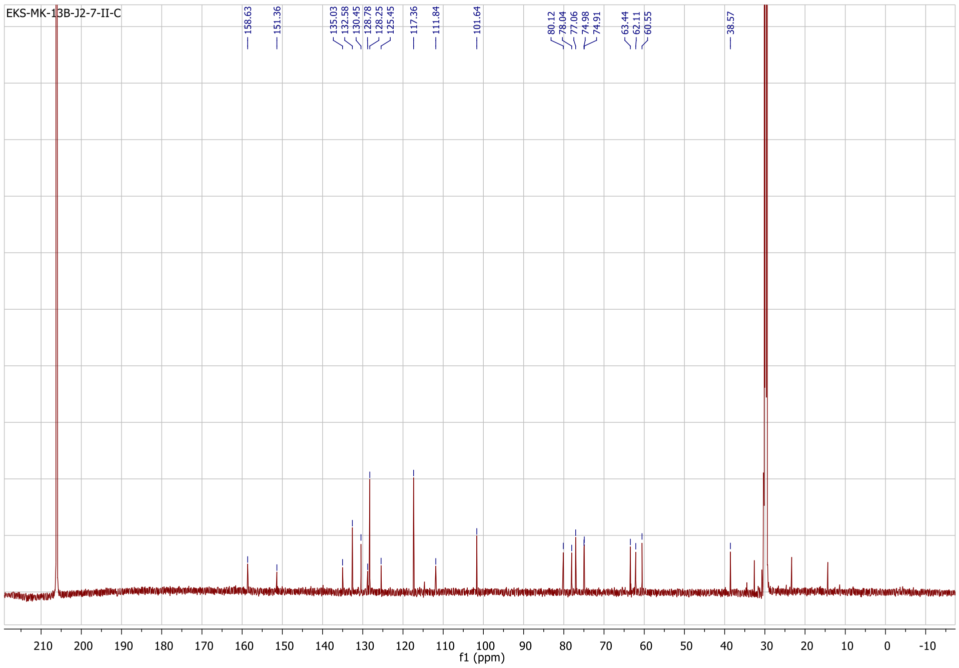

Figure S172. ^{13}C NMR spectrum of 8-bromo-6-chloroflavan-4-ol 4’-*O*-*β*-D-(4”-*O*-methyl)-glucopyranoside **(8a)**

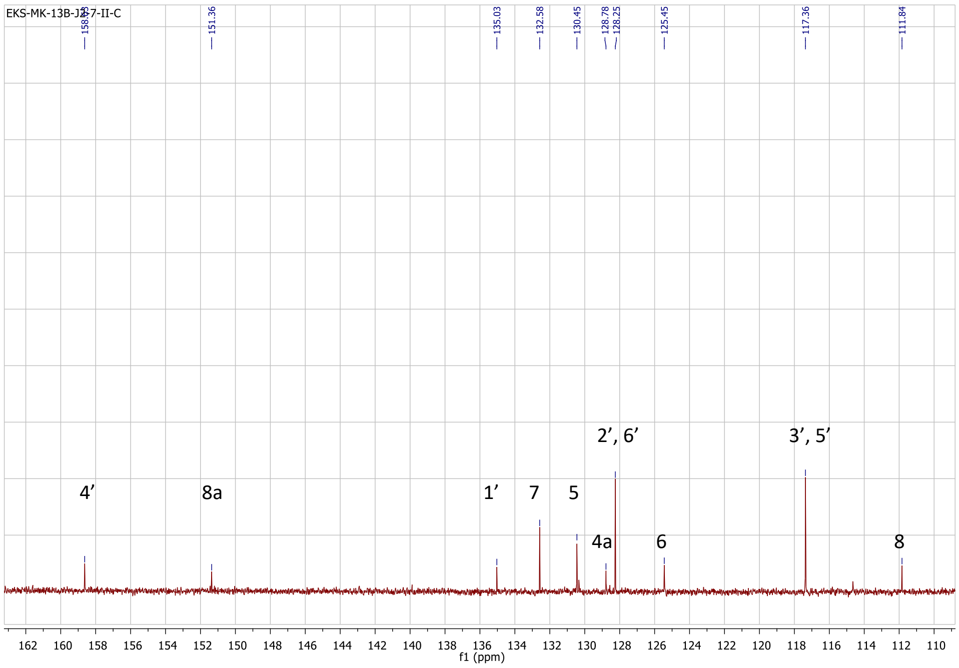

Figure S173. ^{13}C NMR spectrum expansion of 8-bromo-6-chloroflavan-4-ol 4’-*O*-*β*-D-(4”-*O*-methyl)-glucopyranoside **(8a)**

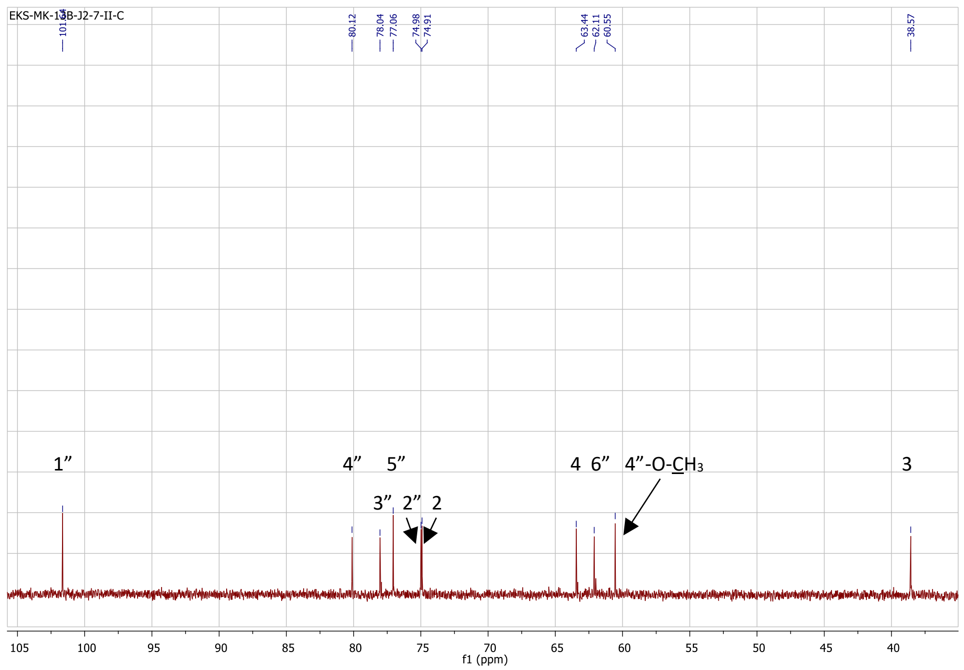

Figure S174. ^{13}C NMR spectrum expansion of 8-bromo-6-chloroflavan-4-ol 4’-*O*-*β*-D-(4”-*O*-methyl)-glucopyranoside **(8a)**

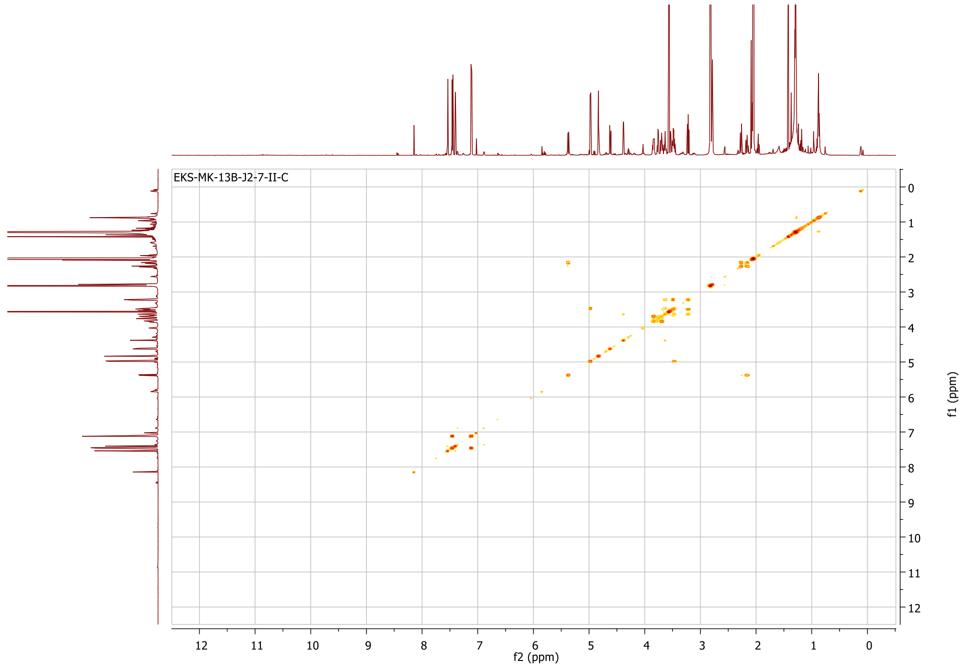

Figure S175. COSY contour map – ^{1}H x ^{1}H of 8-bromo-6-chloroflavan-4-ol 4’-*O*-*β*-D-(4”-*O*-methyl)-glucopyranoside **(8a)**

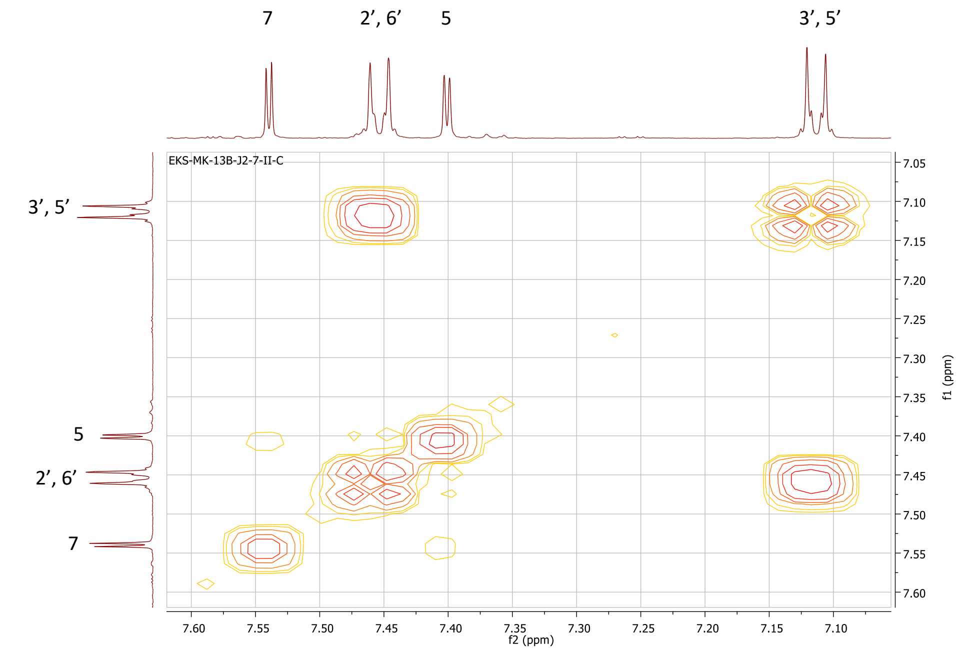

Figure S176. COSY contour map – ^{1}H x ^{1}H expansion of 8-bromo-6-chloroflavan-4-ol 4’-*O*-*β*-D-(4”-*O*-methyl)-glucopyranoside **(8a)**

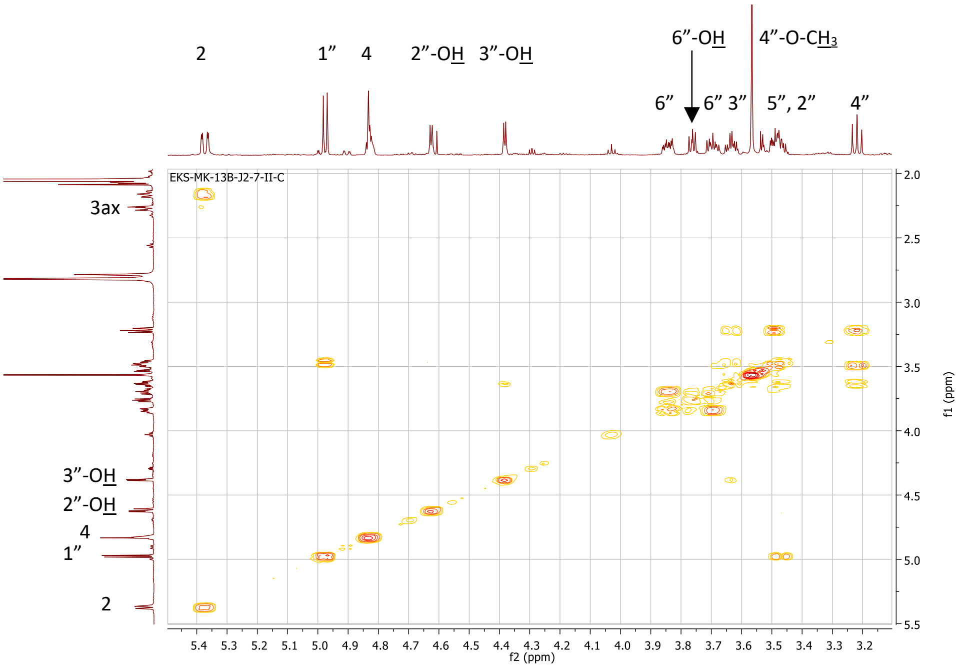

Figure S177. COSY contour map – ^{1}H x ^{1}H expansion of 8-bromo-6-chloroflavan-4-ol 4’-*O*-*β*-D-(4”-*O*-methyl)-glucopyranoside **(8a)**

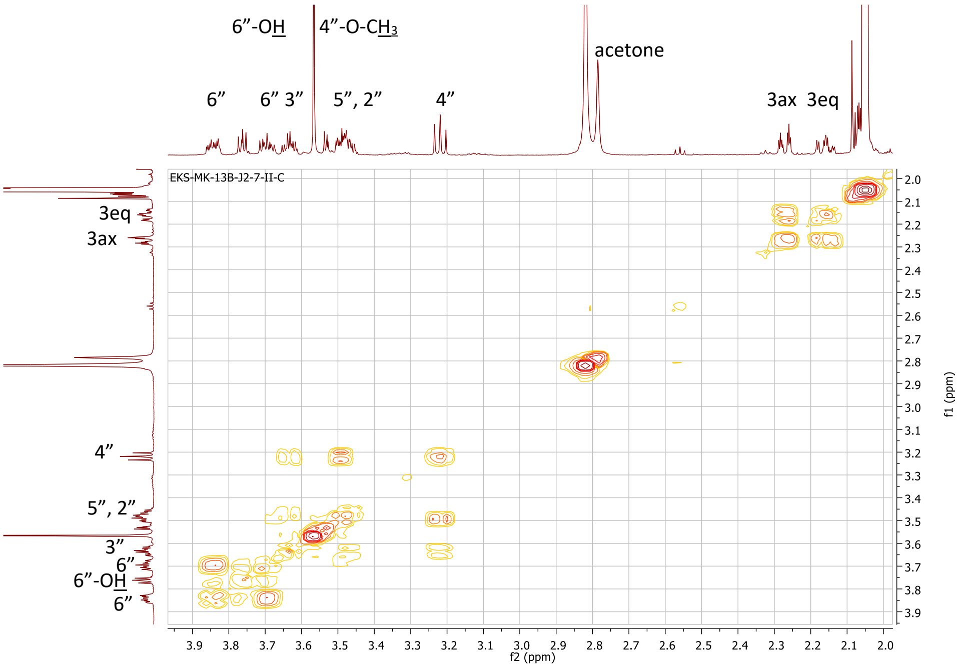

Figure S178. COSY contour map – ^{1}H x ^{1}H expansion of 8-bromo-6-chloroflavan-4-ol 4’-*O*-*β*-D-(4”-*O*-methyl)-glucopyranoside **(8a)**

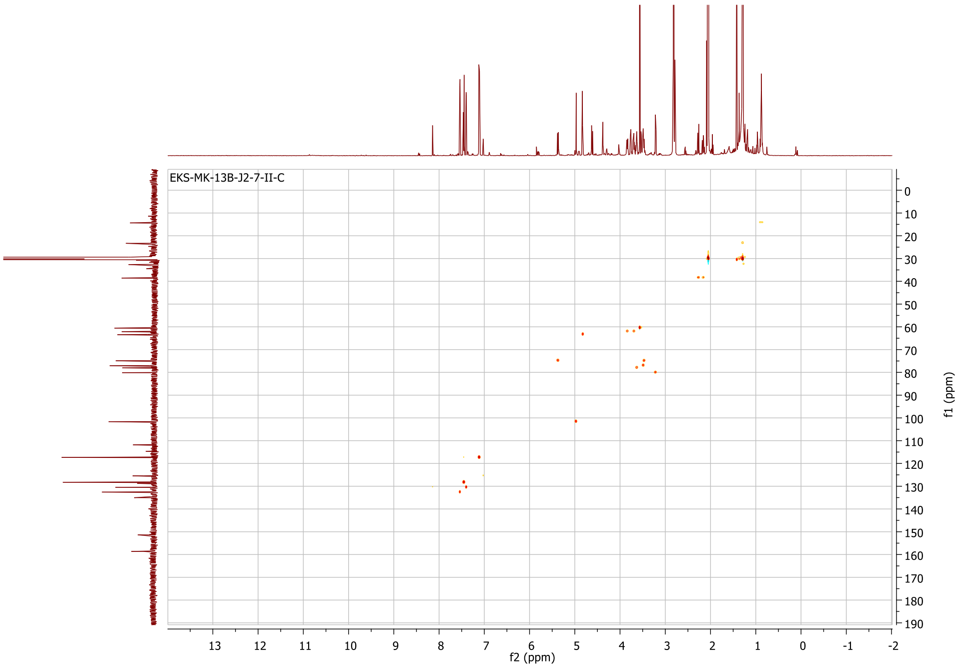

Figure S179. HSQC contour map – ^{1}H x ^{13}C of 8-bromo-6-chloroflavan-4-ol 4’-*O*-*β*-D-(4”-*O*-methyl)-glucopyranoside **(8a)**

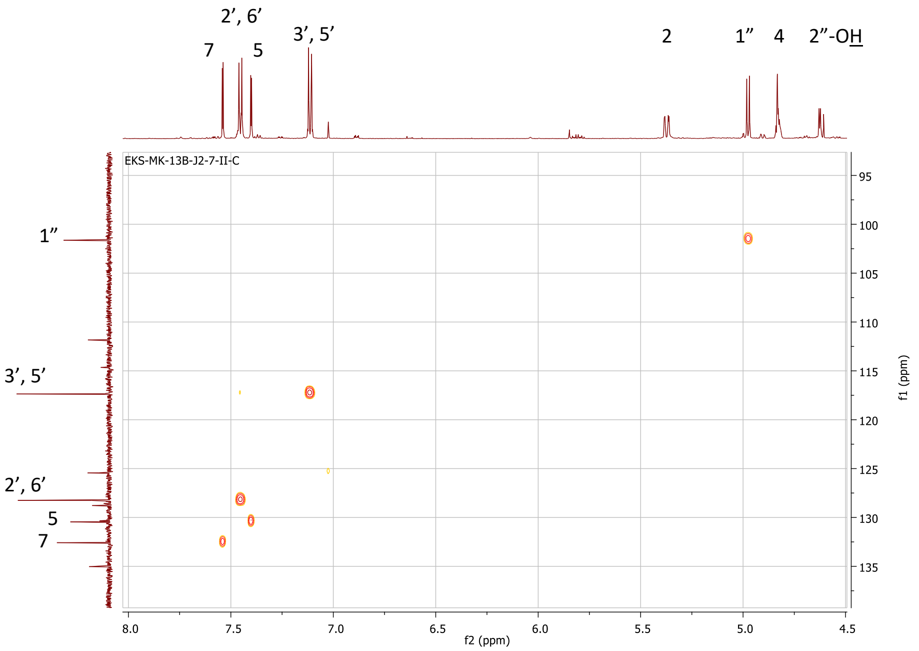

Figure S180. HSQC contour map – ^{1}H x ^{13}C expansion of 8-bromo-6-chloroflavan-4-ol 4'-*O*-*β*-D-(4"-*O*-methyl)-glucopyranoside **(8a)**

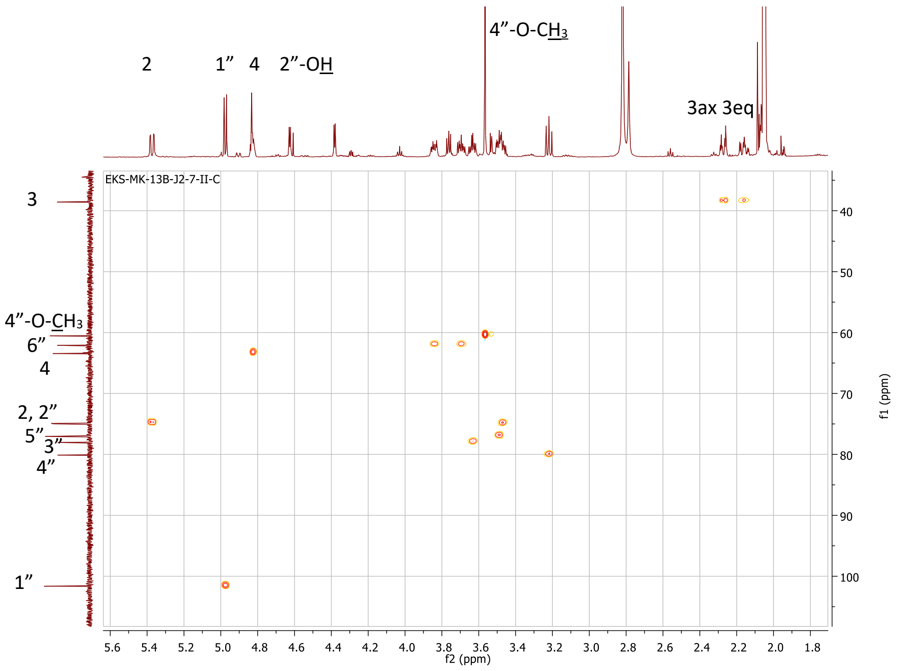

Figure S181. HSQC contour map – ^{1}H x ^{13}C expansion of 8-bromo-6-chloroflavan-4-ol 4'-*O*-*β*-D-(4"-*O*-methyl)-glucopyranoside **(8a)**

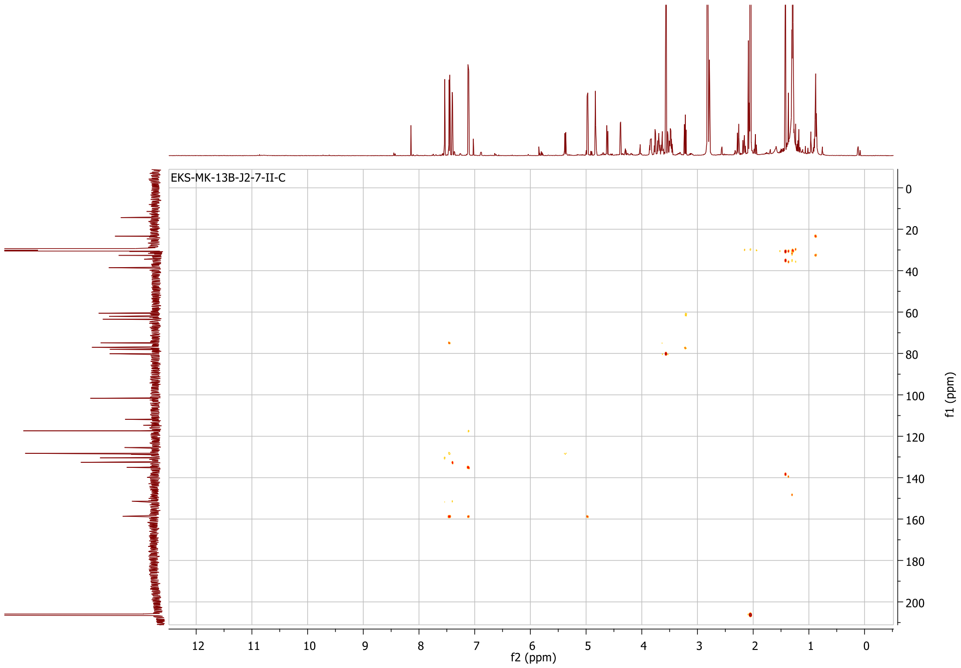

Figure S182. HMBC contour map – ^{1}H x ^{13}C of 8-bromo-6-chloroflavan-4-ol 4'-*O*-*β*-D-(4"-*O*-methyl)-glucopyranoside **(8a)**

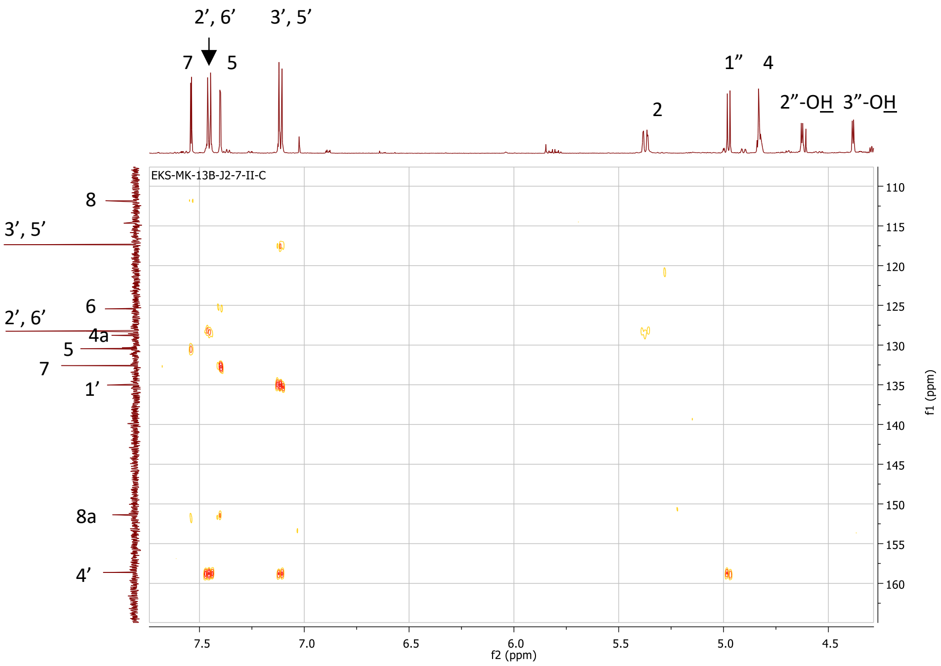

Figure S183. HMBC contour map – ^{1}H x ^{13}C expansion of 8-bromo-6-chloroflavan-4-ol 4'-*O*-*β*-D-(4"-*O*-methyl)-glucopyranoside **(8a)**

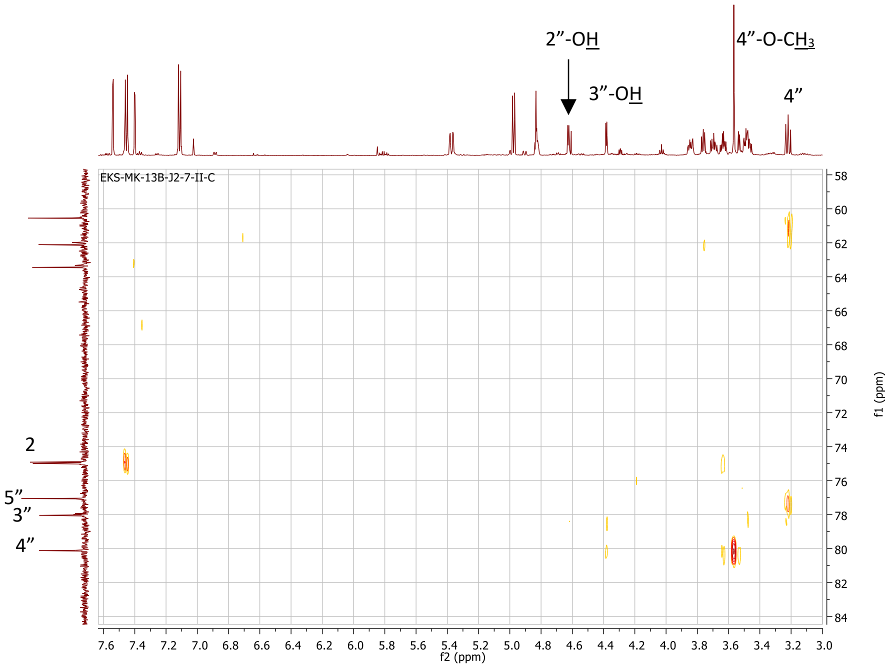

Figure S184. HMBC contour map – ^{1}H x ^{13}C expansion of 8-bromo-6-chloroflavan-4-ol 4’-*O*-*β*-D-(4”-*O*-methyl)-glucopyranoside **(8a)**

Molecular Formula: $C_{15}H_8BrClO_2$
Formula Weight: 335.579
Ionization mode: positive
Precursor $[M + H]^+$: 334.939
Collision energy (CE): -35.0

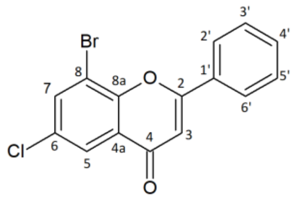

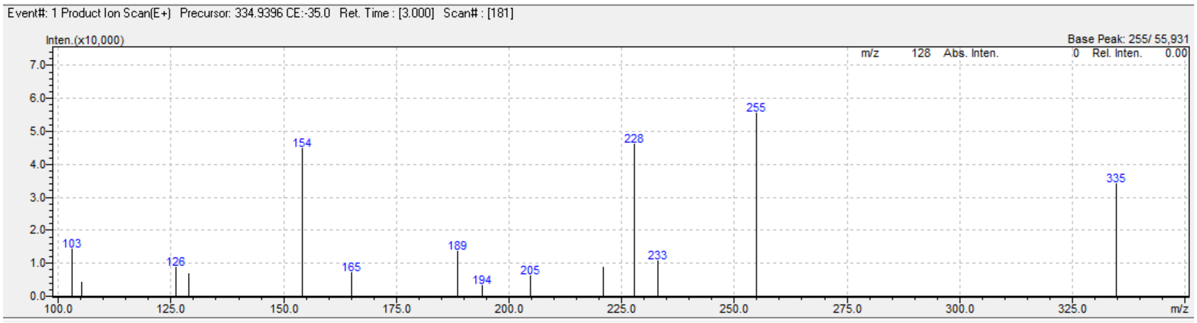

Figure S185. MS analysis of 8-bromo-6-chloroflavone **(9)**

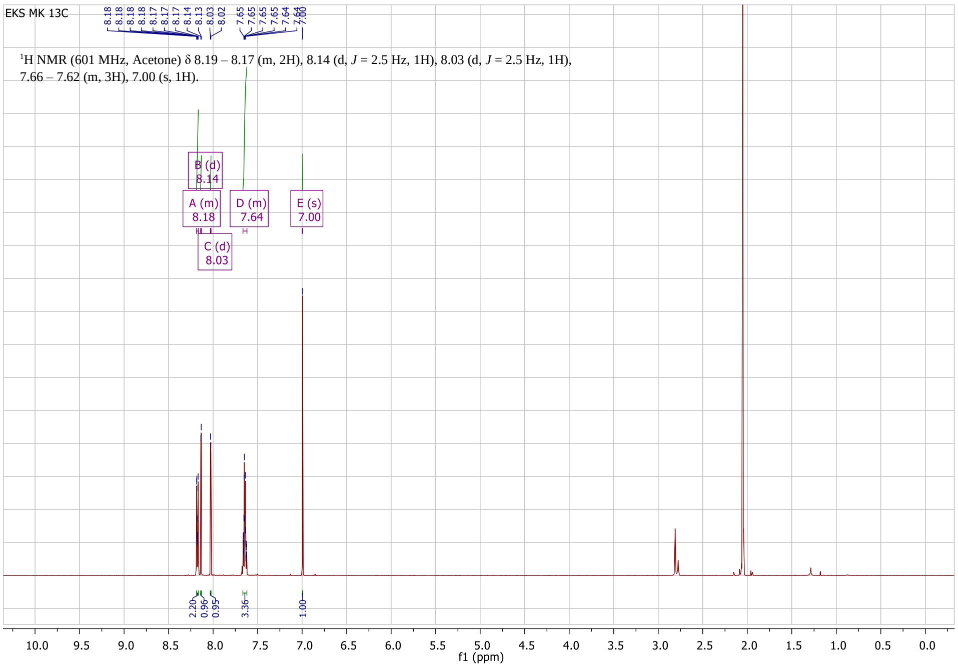

Figure S186. ^{1}H NMR spectrum of 8-bromo-6-chloroflavone **(9)**

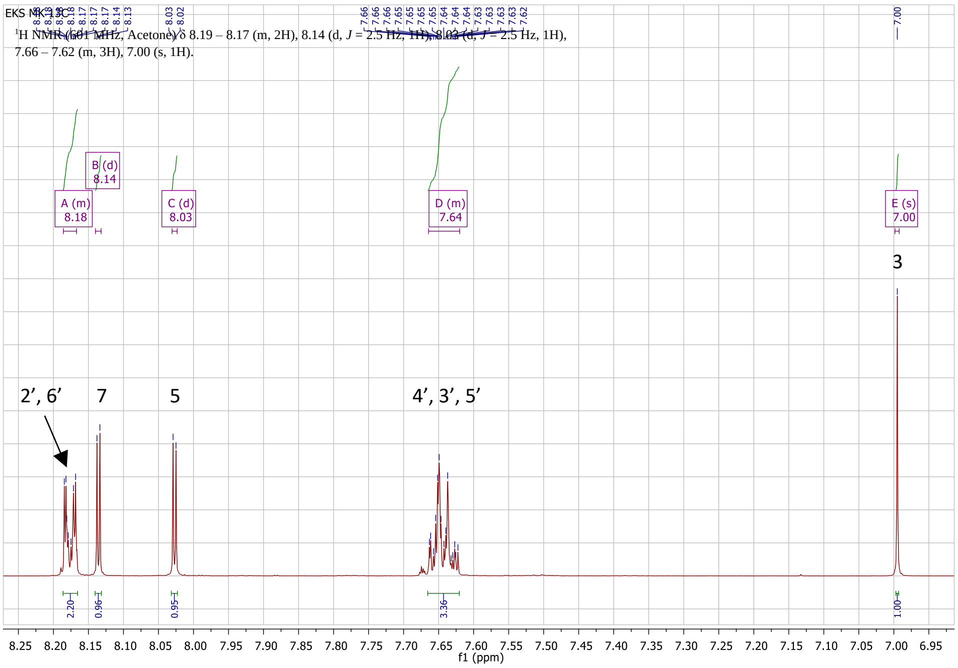

Figure S187. ^{1}H NMR spectrum expansion of 8-bromo-6-chloroflavone **(9)**

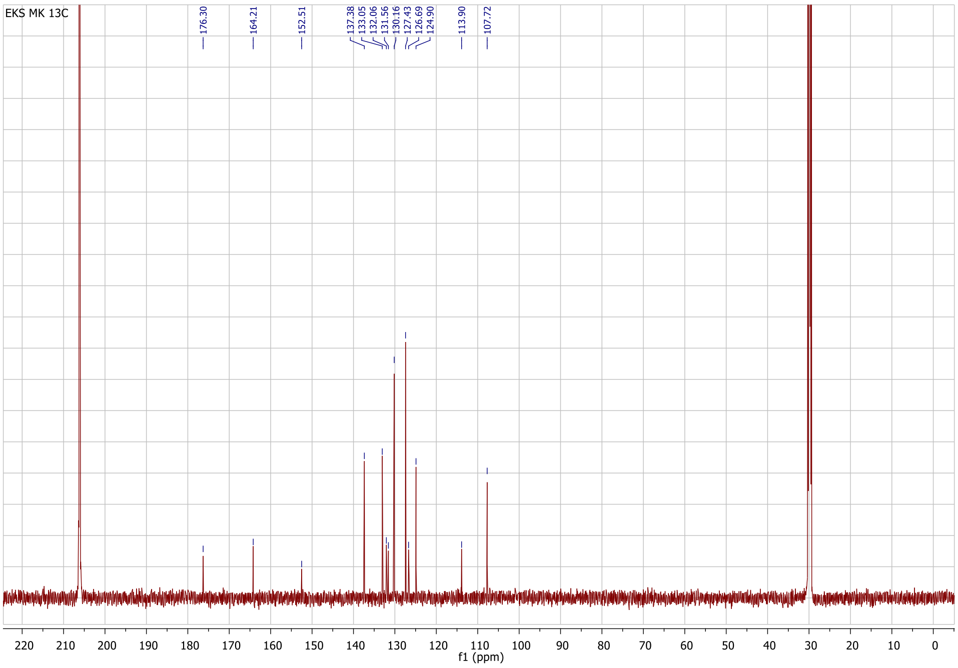

Figure S188. ^{13}C NMR spectrum of 8-bromo-6-chloroflavone **(9)**

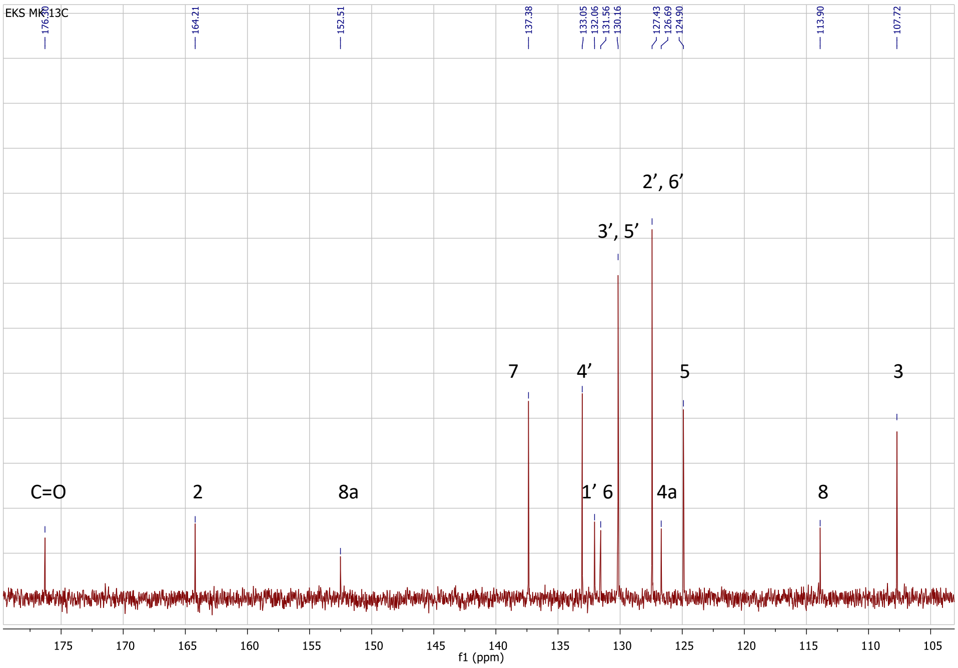

Figure S 189. ^{13}C NMR spectrum expansion of 8-bromo-6-chloroflavone **(9)**

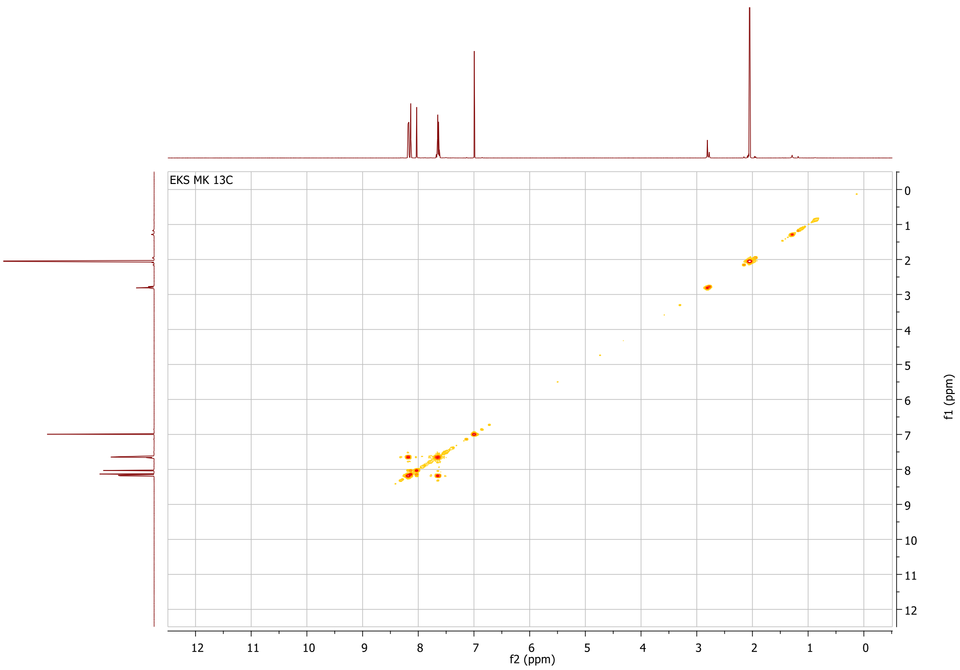

Figure S190. COSY contour map – ^{1}H x ^{1}H of 8-bromo-6-chloroflavone **(9)**

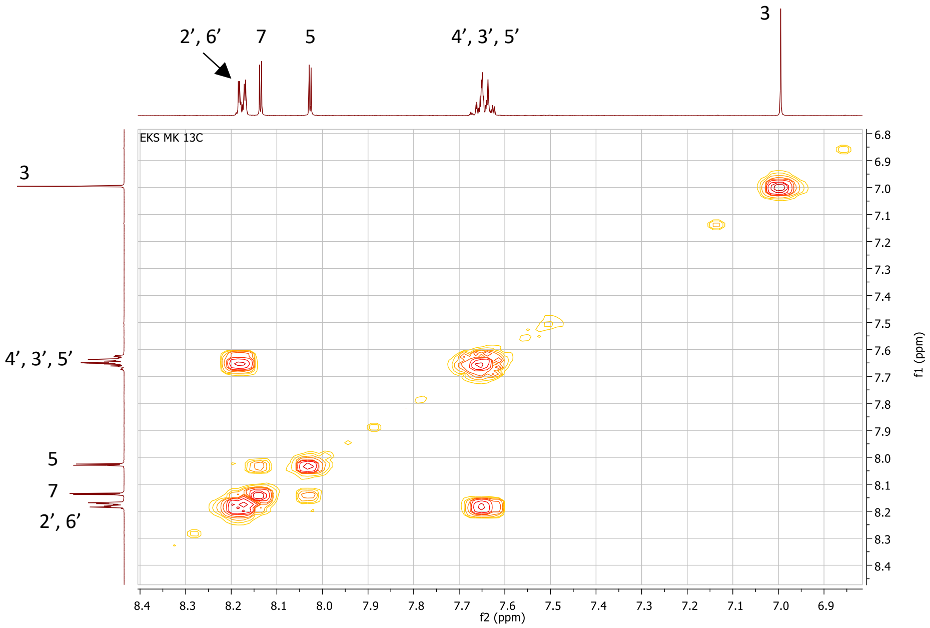

Figure S191. COSY contour map – ^{1}H x ^{1}H expansion of 8-bromo-6-chloroflavone **(9)**

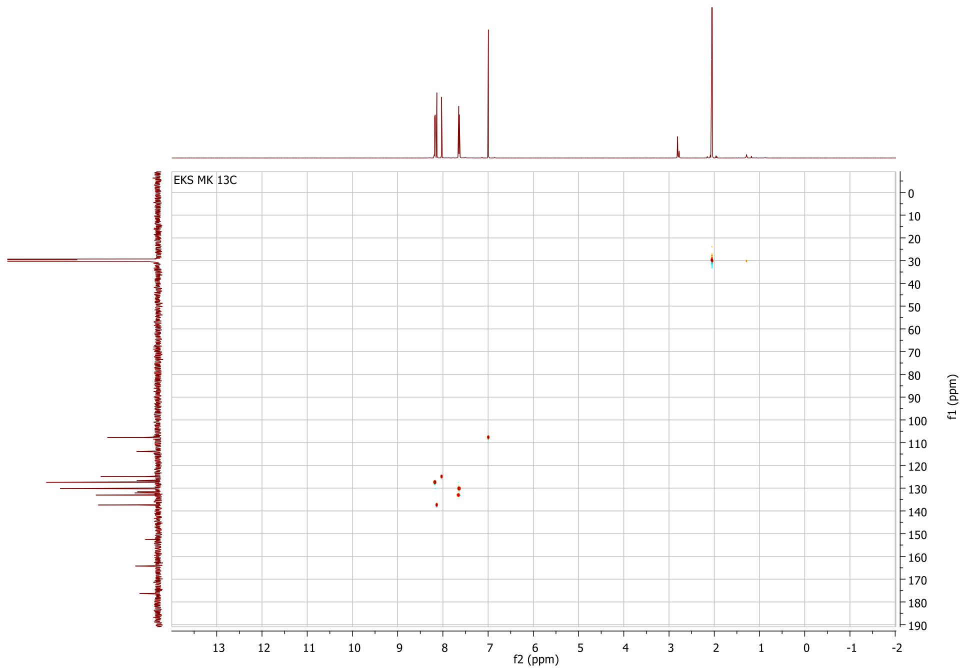

Figure S192. HSQC contour map – ^{1}H x ^{13}C of 8-bromo-6-chloroflavone **(9)**

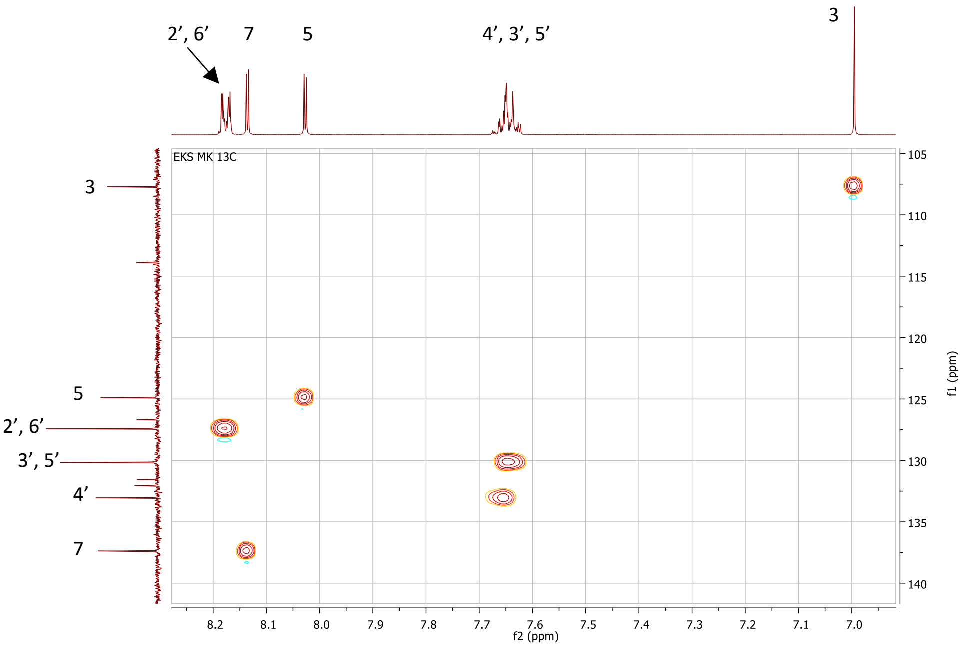

Figure S193. HSQC contour map – ^{1}H x ^{13}C expansion of 8-bromo-6-chloroflavone **(9)**

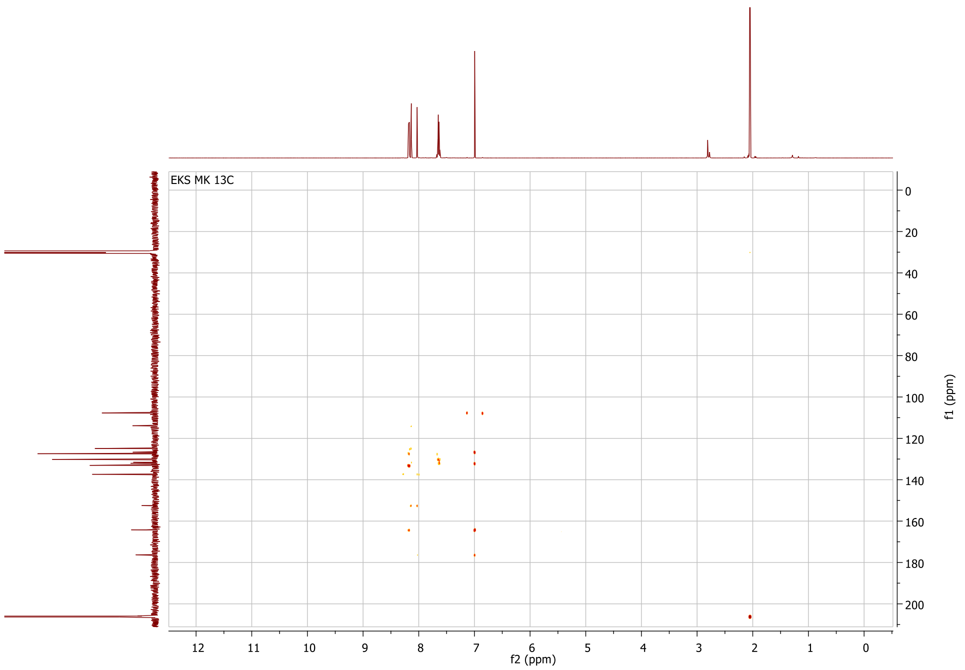

Figure S194. HMBC contour map – ^{1}H x ^{13}C of 8-bromo-6-chloroflavone **(9)**

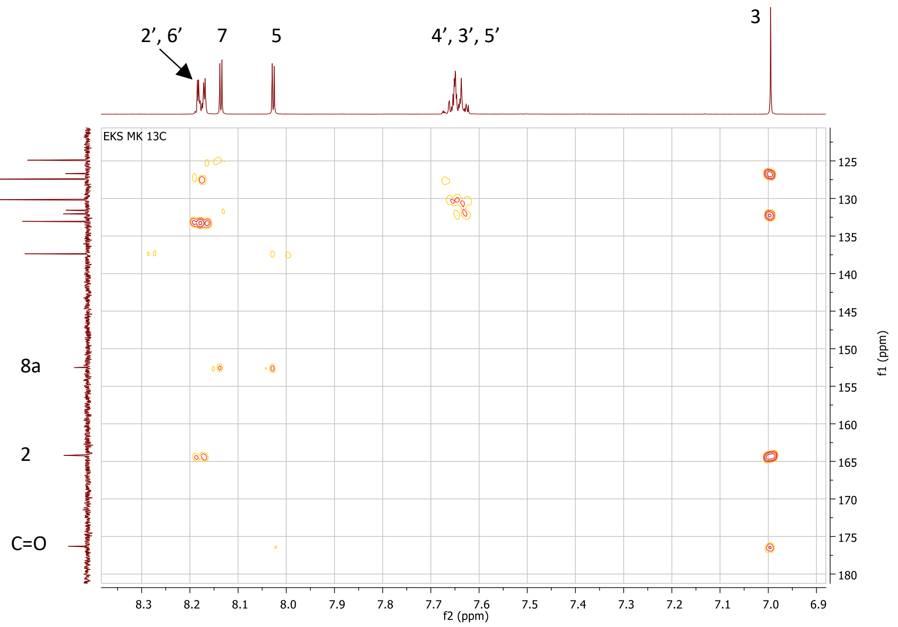

Figure S195. HMBC contour map – ^{1}H x ^{13}C expansion of 8-bromo-6-chloroflavone **(9)**

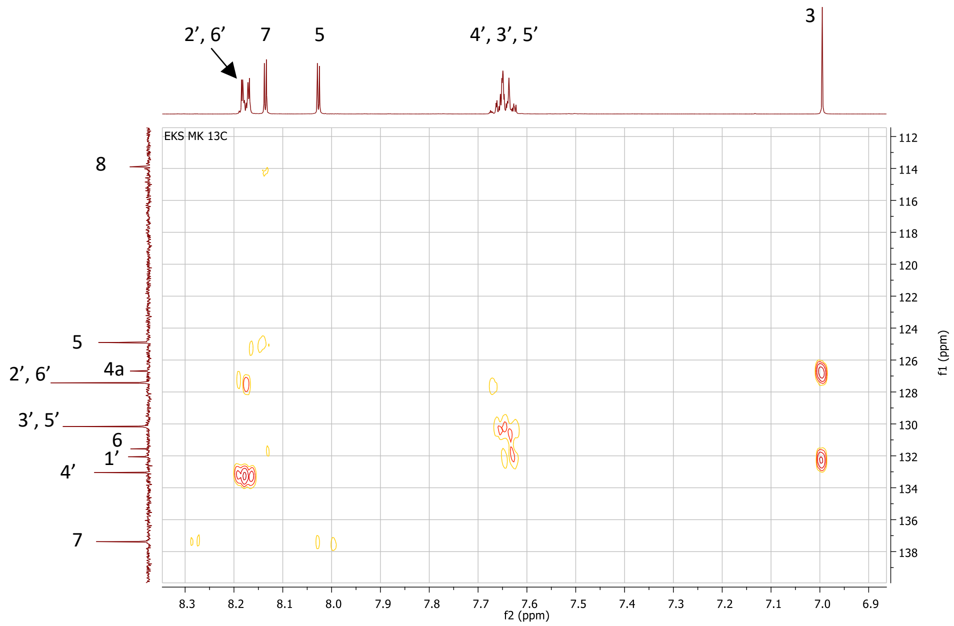

Figure S196. HMBC contour map – ^{1}H x ^{13}C expansion of 8-bromo-6-chloroflavone **(9)**

Molecular Formula: $C_{22}H_{20}BrClO_8$
Formula Weight: 527.746
Ionization mode: positive
Precursor $[M + H]^{+}$: 527.003
Collision energy (CE): -15.0

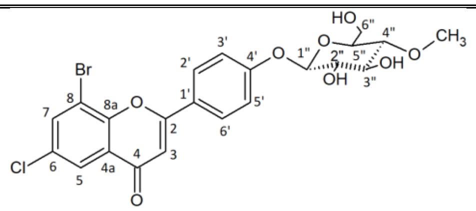

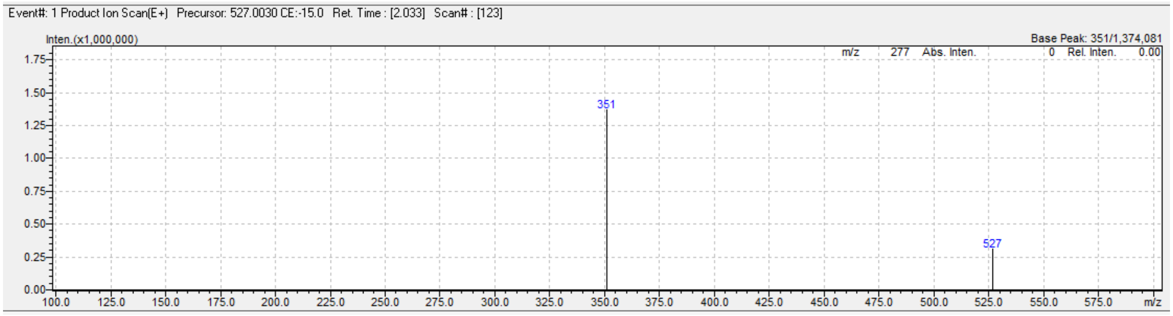

Figure S197. MS analysis of 8-bromo-6-chloroflavone 4'-*O*-*β*-D-(4''-*O*-methyl)-glucopyranoside **(9a)**

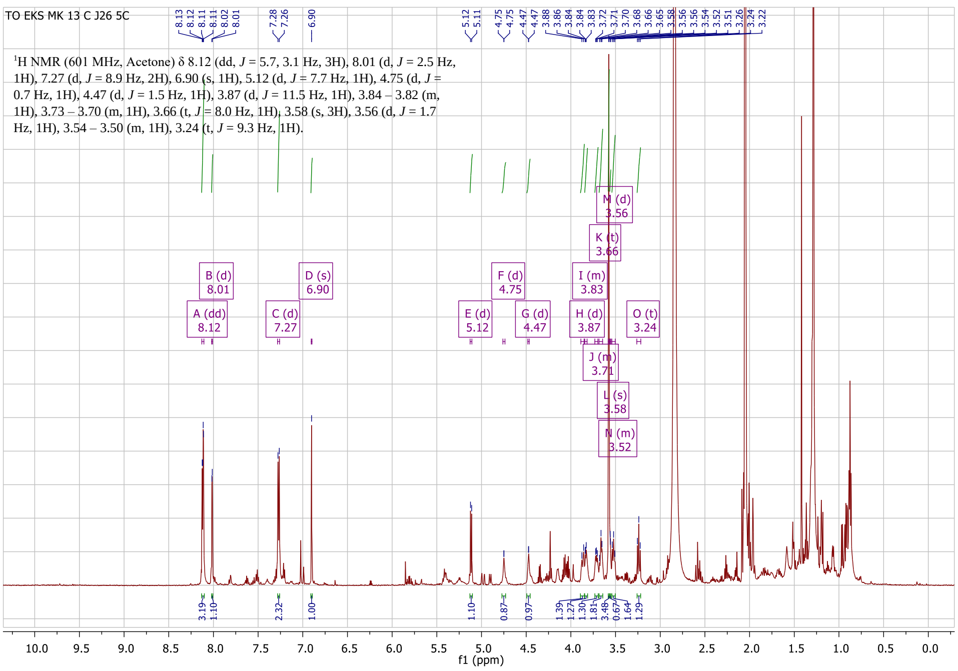

Figure S198. ^{1}H NMR spectrum of 8-bromo-6-chloroflavone 4’-*O*-*β*-D-(4”-*O*-methyl)-glucopyranoside **(9a)**

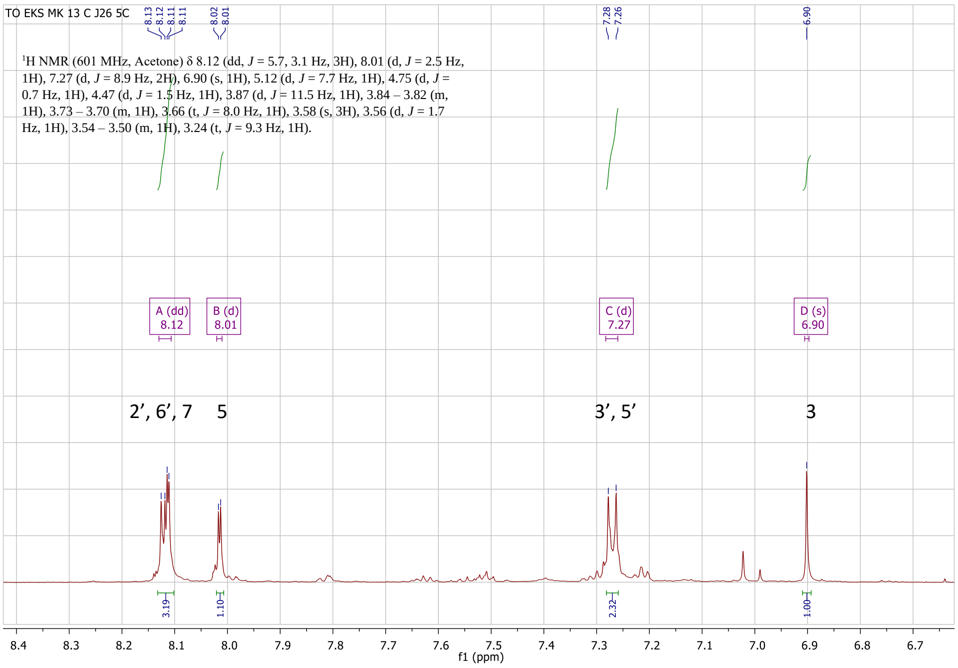

Figure S199. ^{1}H NMR spectrum expansion of 8-bromo-6-chloroflavone 4’-*O*-*β*-D-(4”-*O*-methyl)-glucopyranoside **(9a)**

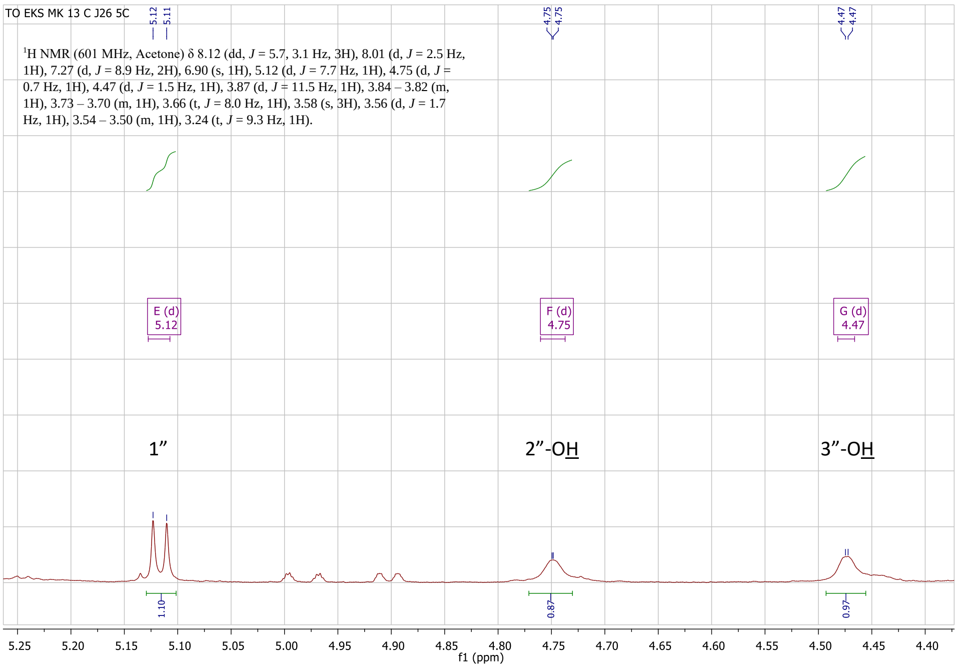

Figure S200. ^{1}H NMR spectrum expansion of 8-bromo-6-chloroflavone 4’-*O*-*β*-D-(4”-*O*-methyl)-glucopyranoside **(9a)**

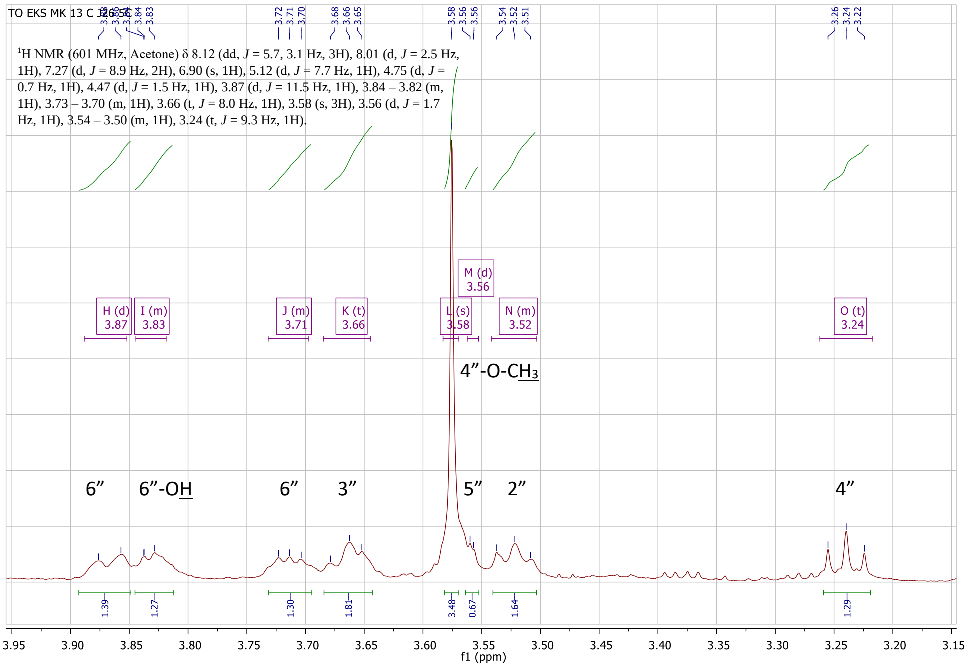

Figure S201. ^{1}H NMR spectrum expansion of 8-bromo-6-chloroflavone 4’-*O*-*β*-D-(4”-*O*-methyl)-glucopyranoside **(9a)**

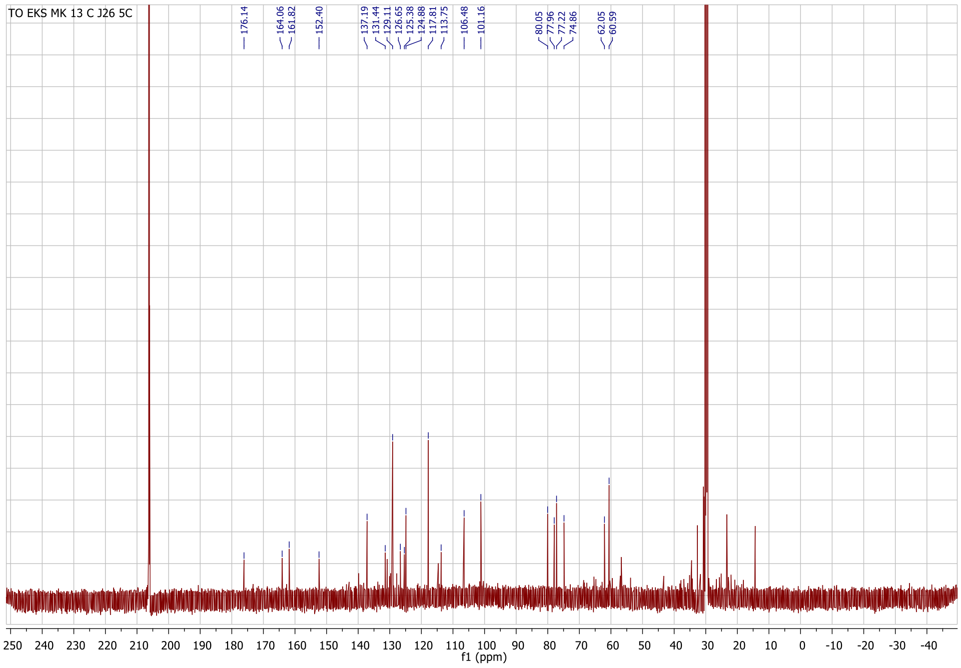

Figure S202. ^{13}C NMR spectrum of 8-bromo-6-chloroflavone 4’-*O*-*β*-D-(4”-*O*-methyl)-glucopyranoside **(9a)**

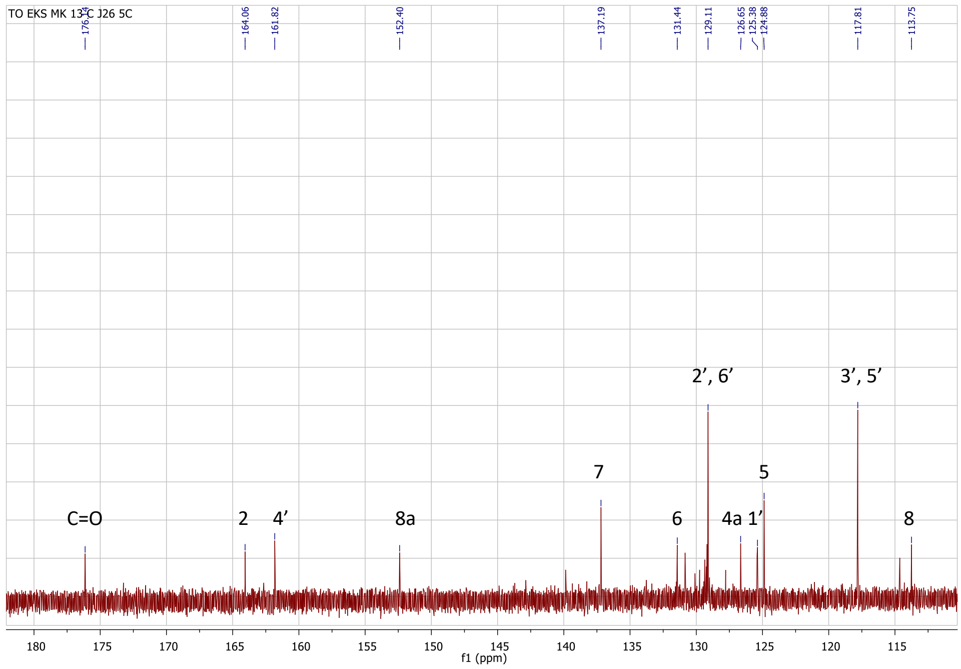

Figure S203. ^{13}C NMR spectrum expansion of 8-bromo-6-chloroflavone 4’-*O*-*β*-D-(4”-*O*-methyl)-glucopyranoside **(9a)**

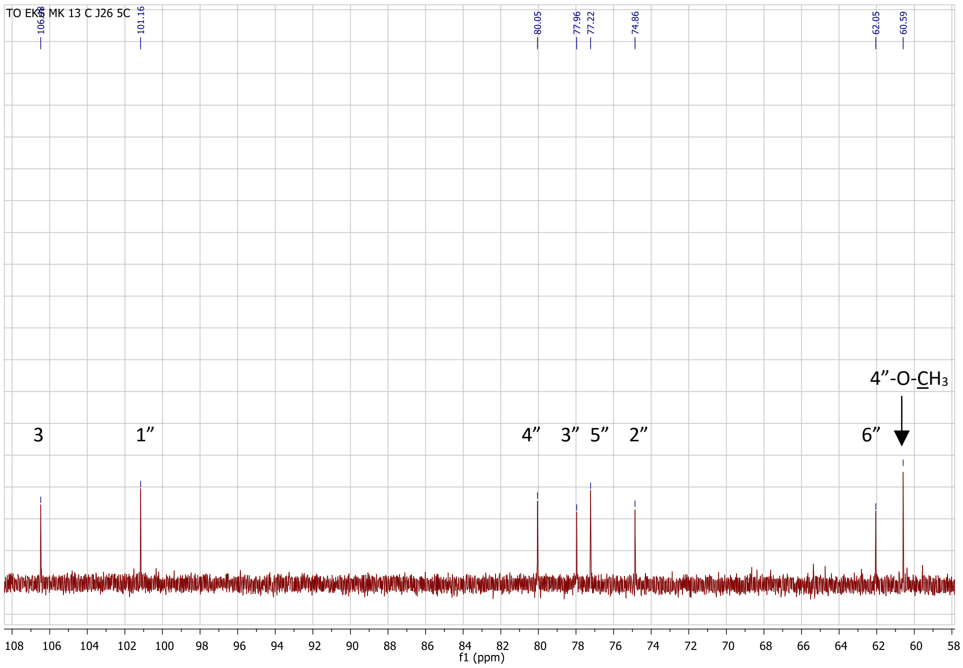

Figure S204. ^{13}C NMR spectrum expansion of 8-bromo-6-chloroflavone 4’-*O*-*β*-D-(4”-*O*-methyl)-glucopyranoside **(9a)**

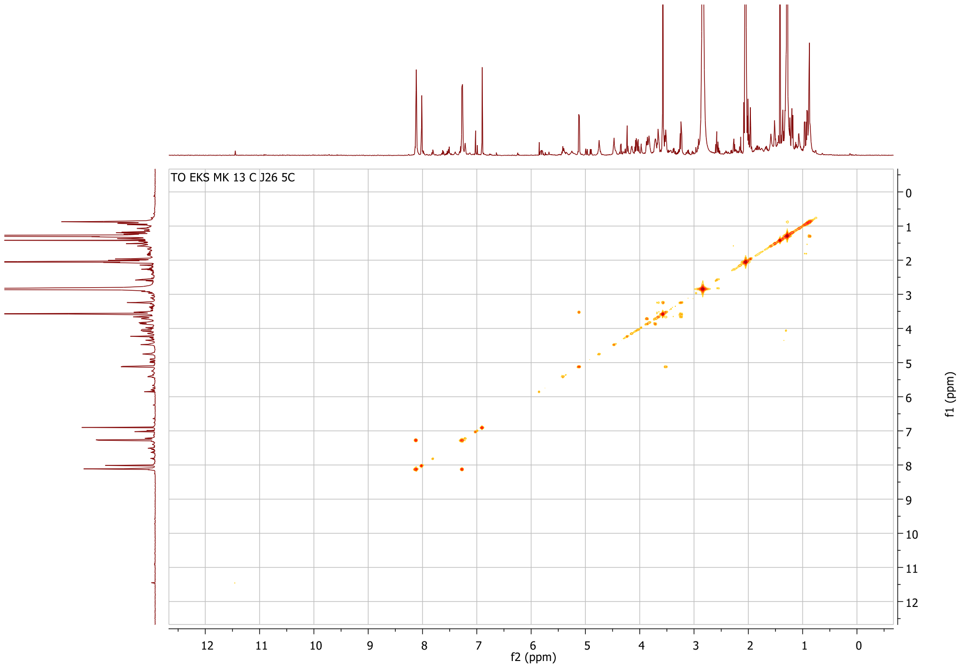

Figure S205. COSY contour map – ^{1}H x ^{1}H of 8-bromo-6-chloroflavone 4’-*O*-*β*-D-(4”-*O*-methyl)-glucopyranoside **(9a)**

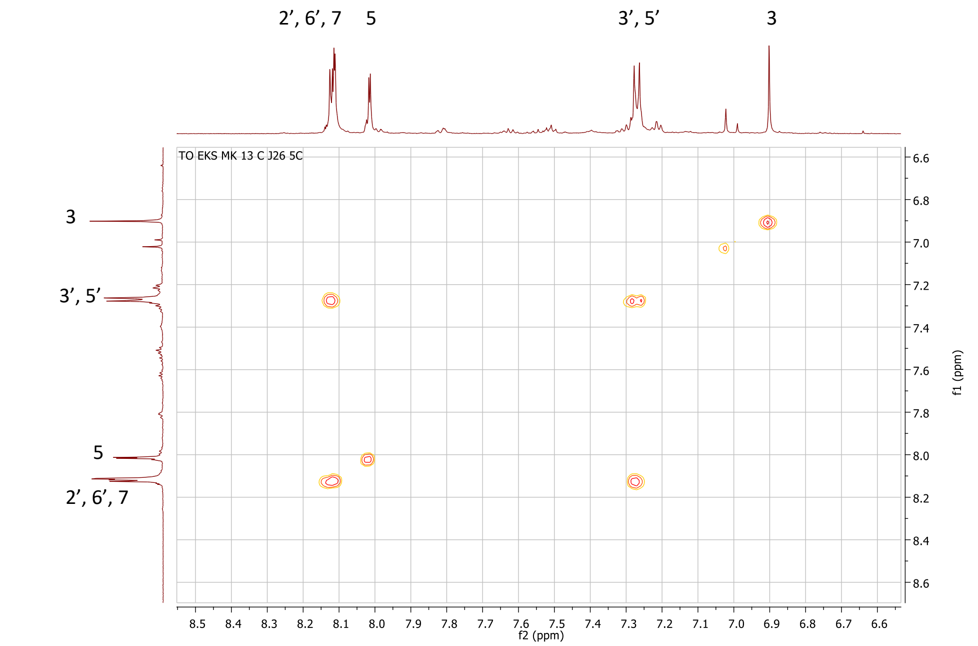

Figure S206. COSY contour map – ^1H x ^1H expansion of 8-bromo-6-chloroflavone 4’-*O*-*β*-D-(4”-*O*-methyl)-glucopyranoside **(9a)**

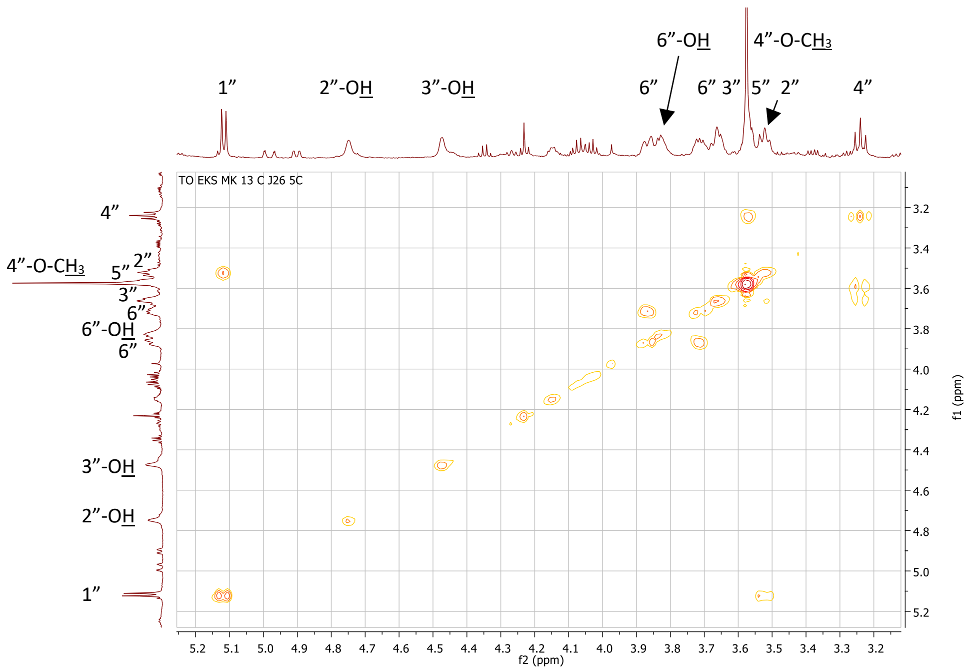

Figure S207. COSY contour map – ^1H x ^1H expansion of 8-bromo-6-chloroflavone 4’-*O*-*β*-D-(4”-*O*-methyl)-glucopyranoside **(9a)**

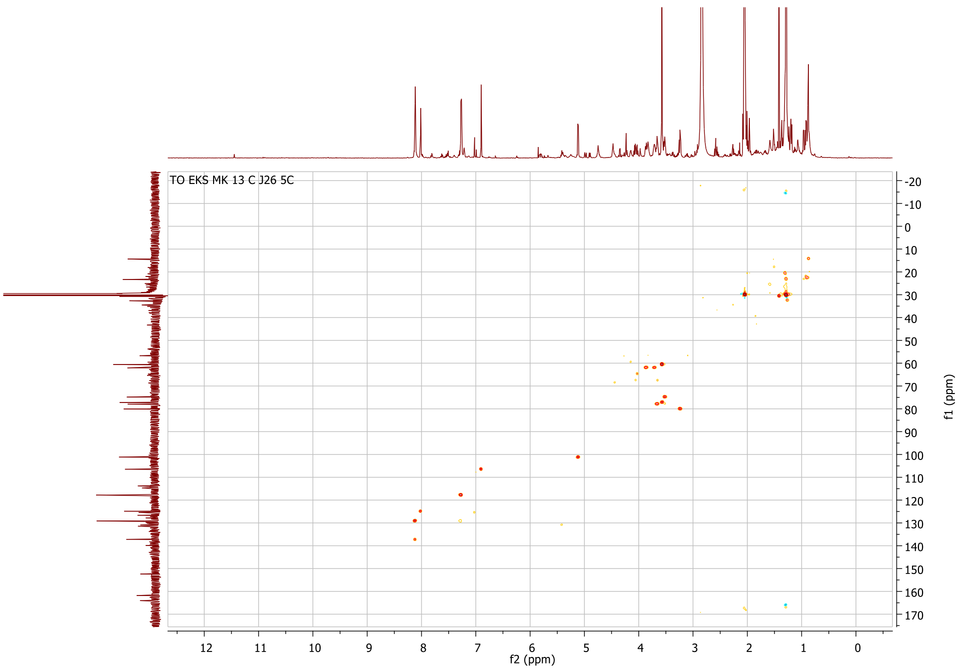

Figure S208. HSQC contour map – ^{1}H x ^{13}C of 8-bromo-6-chloroflavone 4’-*O*-*β*-D-(4”-*O*-methyl)-glucopyranoside **(9a)**

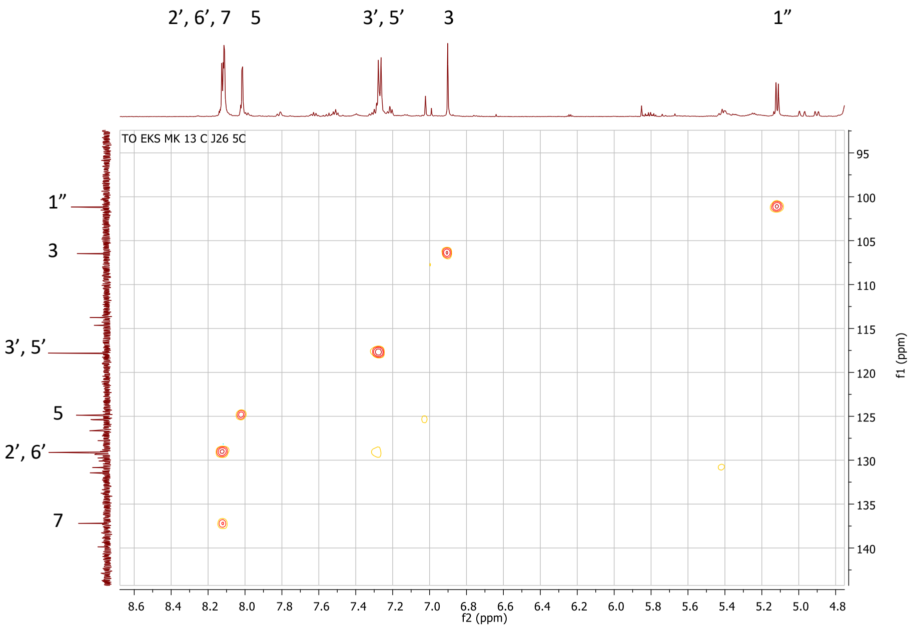

Figure S209. HSQC contour map – ^{1}H x ^{13}C expansion of 8-bromo-6-chloroflavone 4’-*O*-*β*-D-(4”-*O*-methyl)-glucopyranoside **(9a)**

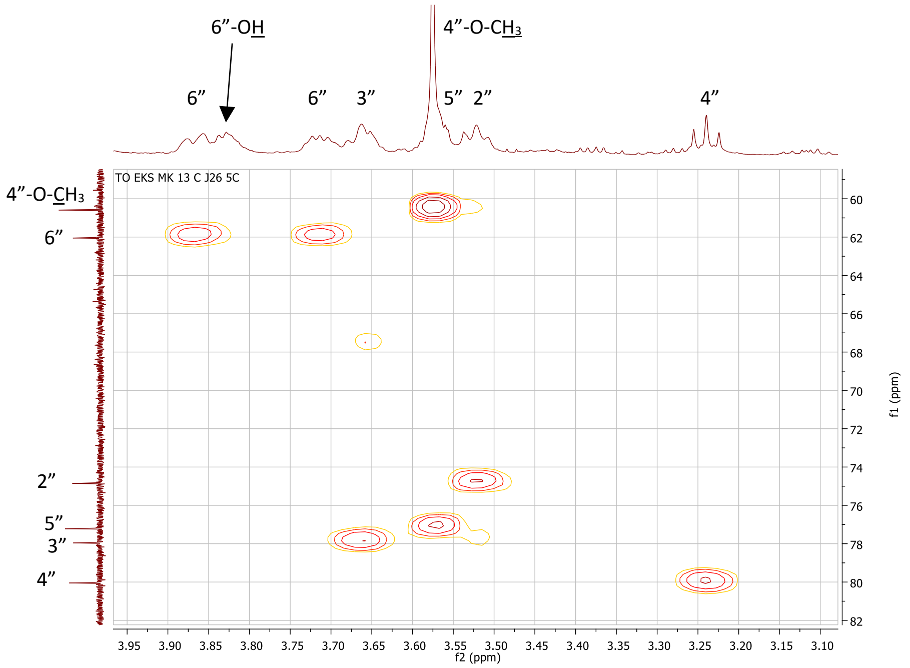

Figure S210. HSQC contour map – ^{1}H x ^{13}C expansion of 8-bromo-6-chloroflavone 4’-*O*-*β*-D-(4”-*O*-methyl)-glucopyranoside **(9a)**

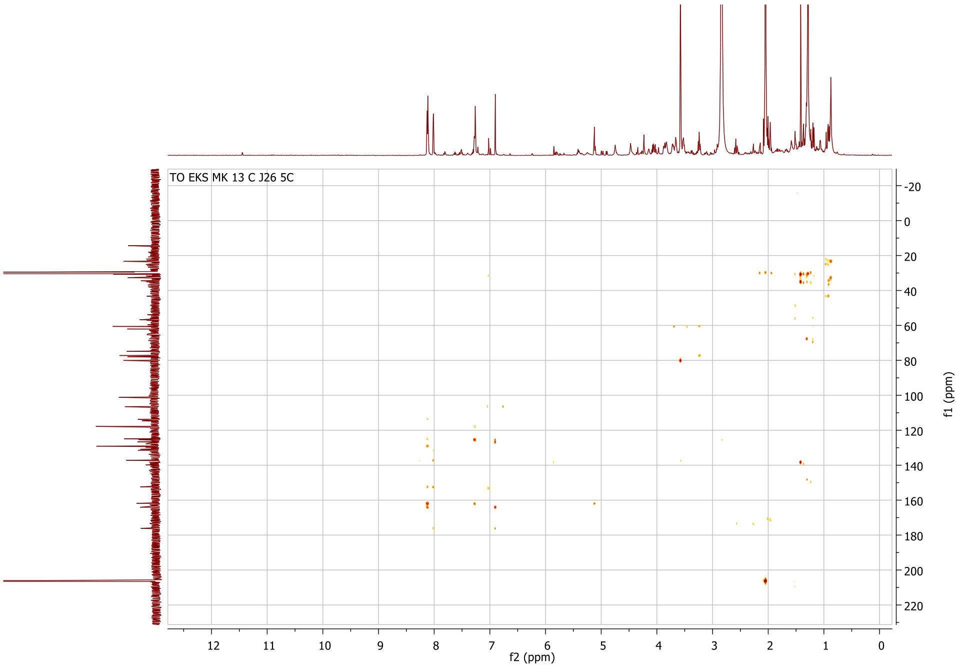

Figure S211. HMBC contour map – ^{1}H x ^{13}C of 8-bromo-6-chloroflavone 4’-*O*-*β*-D-(4”-*O*-methyl)-glucopyranoside **(9a)**

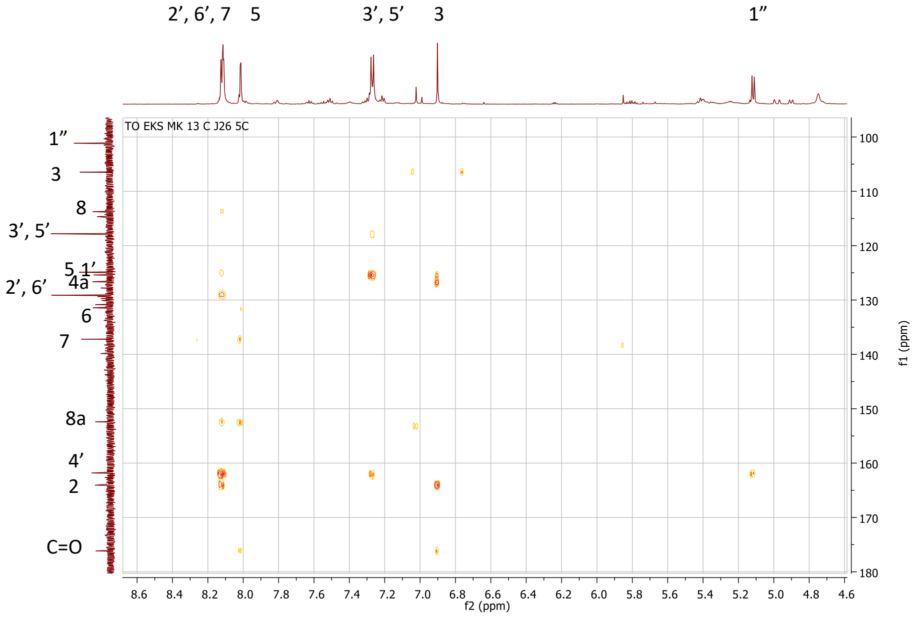

Figure S212. HMBC contour map – ^{1}H x ^{13}C expansion of 8-bromo-6-chloroflavone 4'-*O*-*β*-D-(4"-*O*-methyl)-glucopyranoside **(9a)**

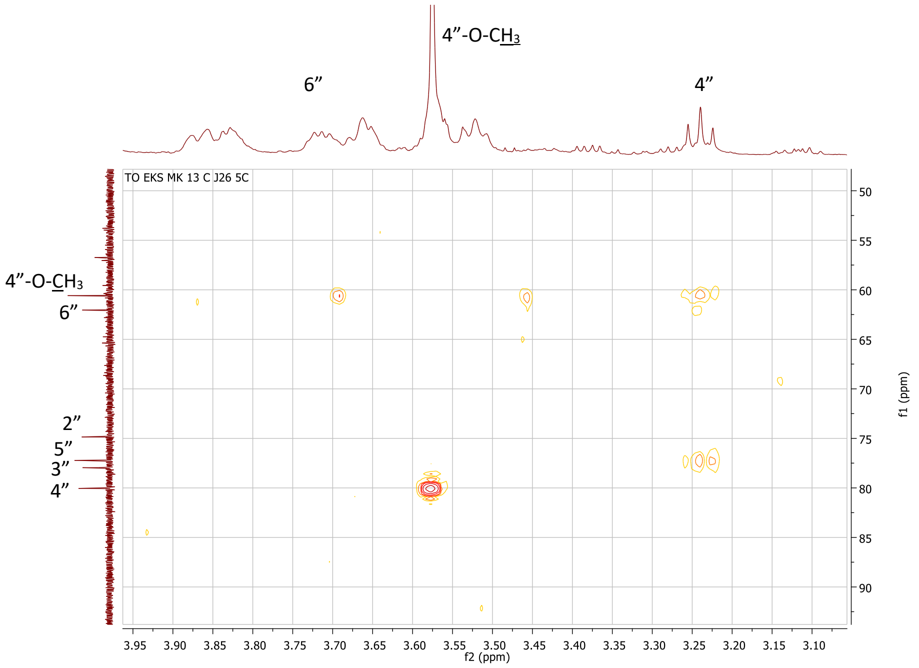

Figure S213. HMBC contour map – ^{1}H x ^{13}C expansion of 8-bromo-6-chloroflavone 4'-*O*-*β*-D-(4"-*O*-methyl)-glucopyranoside **(9a)**
